# Anthraquinone Derivatives and Other Aromatic Compounds from Marine Fungus *Asteromyces cruciatus* KMM 4696 and Their Effects against *Staphylococcus aureus*

**DOI:** 10.3390/md21080431

**Published:** 2023-07-29

**Authors:** Olesya I. Zhuravleva, Ekaterina A. Chingizova, Galina K. Oleinikova, Sofya S. Starnovskaya, Alexandr S. Antonov, Natalia N. Kirichuk, Alexander S. Menshov, Roman S. Popov, Natalya Yu. Kim, Dmitrii V. Berdyshev, Artur R. Chingizov, Alexandra S. Kuzmich, Irina V. Guzhova, Anton N. Yurchenko, Ekaterina A. Yurchenko

**Affiliations:** 1 G.B. Elyakov Pacific Institute of Bioorganic Chemistry, Far Eastern Branch of the Russian Academy of Sciences, Prospect 100-Letiya Vladivostoka, 159, Vladivostok 690022, Russia; zhuravleva.oi@dvfu.ru (O.I.Z.); martyyas@mail.ru (E.A.C.);; 2 Institute of High Technologies and Advanced Materials, Far Eastern Federal University, 10 Ajax Bay, Russky Island, Vladivostok 690922, Russia; 3 Institute of Cytology Russian Academy of Sciences, Tikhoretskiy Ave. 4, St. Petersburg 194064, Russia; irina.guzhova@incras.ru

**Keywords:** marine-derived fungus, secondary metabolites, anthraquinones, antibiotics, skin infection, HaCaT, sortase A, urease, migration

## Abstract

New anthraquinone derivatives acruciquinones A–C (**1**–**3**), together with ten known metabolites, were isolated from the obligate marine fungus *Asteromyces cruciatus* KMM 4696. Acruciquinone C is the first member of anthraquinone derivatives with a 6/6/5 backbone. The structures of isolated compounds were established based on NMR and MS data. The absolute stereoconfigurations of new acruciquinones A–C were determined using ECD and quantum chemical calculations (TDDFT approach). A plausible biosynthetic pathway of the novel acruciquinone C was proposed. Compounds **1**–**4** and **6**–**13** showed a significant antimicrobial effects against *Staphylococcus aureus* growth, and acruciquinone A (**1**), dendryol B (**4**), coniothyrinone B (**7**), and ω-hydroxypachybasin (**9**) reduced the activity of a key staphylococcal enzyme, sortase A. Moreover, the compounds, excluding **4**, inhibited urease activity. We studied the effects of anthraquinones **1**, **4**, **7**, and **9** and coniothyrinone D (**6**) in an in vitro model of skin infection when HaCaT keratinocytes were cocultivated with *S. aureus*. Anthraquinones significantly reduce the negative impact of *S. aureus* on the viability, migration, and proliferation of infected HaCaT keratinocytes, and acruciquinone A (**1**) revealed the most pronounced effect.

## 1. Introduction

Anthraquinones are usual metabolites for marine fungi. A recent review by Hafez Ghoran and coauthors described 296 specialized metabolites belonging to the anthraquinone class, which were isolated from 28 marine fungal strains from 2000 to 2021 [1]. They are acetate-derivative metabolites originating from a polyketide containing eight C2 units, which generates, in turn, with three aldol condensations, the carbon skeleton of anthraquinones, except for the two carbonyl oxygens of the central ring. The presence in their structure of many different functional groups makes them very active in interaction with various molecular targets and exhibit wide spectrum of biological activities, including anticancer and antibacterial effects [2].

One of the five main causative agents of nosocomial infections, which are united by the abbreviation ESKAPE, is *Staphylococcus aureus* [3]. A decrease in the protective properties of the skin and the body in hospital patients leads to damage to keratinocytes under the influence of *S. aureus* lytic toxins, the destruction of the protective barrier, and the penetration of *S. aureus* into the bloodstream [4]. The global prevalence of bacterial skin diseases in 2019, according to the Global Burden of Disease project, was 14,684.3 cases per 100,000 population [5]. These diseases have rarely been fatal (0.9 cases per 100,000), but the slightest infection can lead to sepsis if the course is unfavorable. There were an estimated 48.9 million cases of sepsis and 11.0 million sepsis-related deaths worldwide in 2017, accounting for 19.7% of all deaths worldwide [6], and the Gram-positive bacterium *S. aureus* is one of the main reasons for this.

Recently, chlorine-containing compounds acrucipentyns A–F were isolated by us from *Asteromyces cruciatus* KMM4696 fungus associated with brown alga *Sargassum pallidum*, and these compounds showed significant antibacterial activity against *Staphylococcus aureus* [7]. The detailed separation of the non-polar part of this fungal extract resulted in the isolation of a number of new and known anthraquinone derivatives. Thus, in this work, we describe the isolation and determination of the structure of these compounds, as well as the study of their antimicrobial properties, including their effects against *Staphylococcus aureus*-infected human HaCaT keratinocytes.

## 2. Results

### 2.1. Isolated Compounds from Asteromyces cruciatus

**Figure 1:**
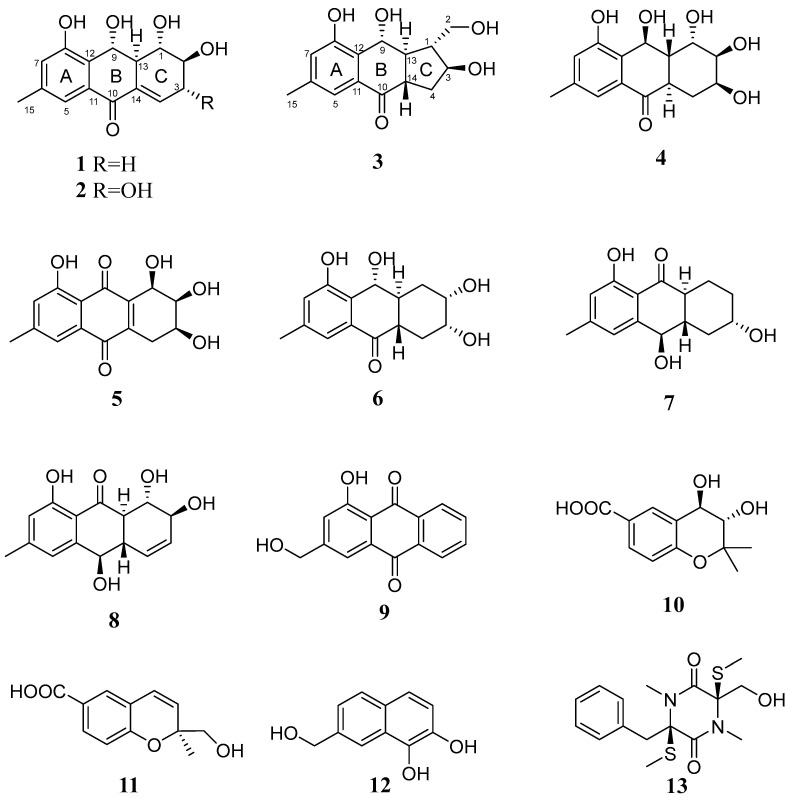
Isolated compounds from *Asteromyces cruciatus*.

As a result of chromatographic separation of the ethyl acetate extract of the culture of the fungus *Asteromyces cruciatus* KMM 4696, new acruciquinones A–C (**1**–**3**), as well as known dendryol B (**4**) [8], pleosporone (**5**) [9], coniothyrinone D (**6**) [10], coniothyrinone B (**7**) [10], rubrumol (**8**) [11], ω-hydroxypachybasin (**9**) [12,13], trans-3,4-dihydroxy-3,4-dihydroanofinic acid (**10**) [14], quadricinctapyran A **(11**) [15], 7-hydroxymethyl-1,2-naphthalenediol (**12**) [16], and gliovictin (**13**) [17] (Figure 1), were isolated. The known compounds (**4**–**13**) were characterized by ^1^H, ^13^C NMR, and HR ESI MS data and identified by comparison with literature data.

### 2.2. Structural Characterization of New Compounds

The molecular formula of **1** was determined as C_15_H_16_O_5_ based on the analysis of the (+)-HRESIMS spectrum (Figure S87) containing the peak of the cationized molecule [M + Na]+ (*m*/*z* 299.0887) and was confirmed by the ^13^C NMR data. In the ^1^H and ^13^C NMR spectra of compound **1** (Table 1, Figures S7–S12) there were signals of a tetrasubstituted benzene ring; an olefinic proton; a methyl; a methylene, and four methine groups, three of which were oxygenated; five *sp*^2^-hybridized quaternary carbons; and one unsaturated keto group.

**Figure 2:**
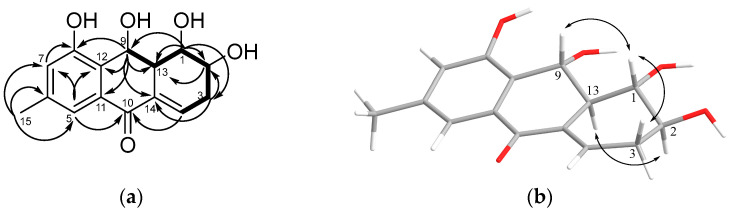
Key ^1^H–^13^C HMBC and ^1^H-^1^H-COSY correlations (**a**) and ROESY correlations (**b**) of **1**.

The HMBC correlations from H-5 to C-7, C-10, C-12, and C-15; from H-7 to C-5, C-8, and C-12; from H3-15 to C-5, C-6, and C-7; and from H-9 to C-8, C-11, C-12, and C-13 (Figure 2a and Figure S11) established the structure of rings A and B and determined the position of the methyl and hydroxyl groups in the tetrasubstituted benzene ring and the hydroxyl and keto groups in ring B. Observed ^1^H-^1^H-COSY interactions (H-9/H-13/H-1/H-2/H2-3/ H-4) and HMBC correlations from H-1 to C-2, C-3, C-9, and C-13; from H-3α to C-1, C-2, C-4, and C-14; and from H-4 to C-10 determined the structure of ring C, its fusion with ring B at C-13/C-14, the position of hydroxyl groups at C-1 and C-2, and the ∆4,14 position of the trisubstituted double bond.

The vicinal coupling constant values (Table 1), as well as the ROESY correlations (Figure 2b and Figure S12) of the H-1/H-3β, H-9, and H-2/H-13 correlations, show that the first three protons in **1** are on the same side of the molecule, while H-2 and H-13 are oriented in the opposite direction.

The molecular formula of compound **2** was determined as C_15_H_16_O_6_ based on the analysis of the (+)-HRESIMS spectrum data containing the peak of the cationized molecule [M + Na]^+^ (*m*/*z* 315.0830) and was confirmed by the ^13^C NMR data. The ^1^H and ^13^C NMR spectra of compound **2** (Table 1, Figures S14–S18) were very similar to those for **1**, with the exception of proton and carbon signals at C-1, C-2, C-3, C-4, and C-14 of the cyclohexene ring. Downfield chemical shifts at C-3 and the presence of an additional methine group in **2** instead of a methylene group in 1 suggested the structure of **2** as a 3-hydroxy derivative of **1**. Observed ^1^H-^1^H COSY interactions (H-13/H-1/H-2/H-3/H-4) proved the position of the hydroxyl groups in compound **2** at C-1, C-2, and C-3 (Figure S17).

**Figure 3:**
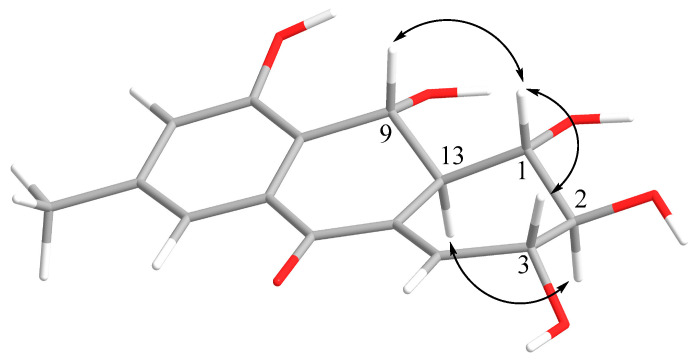
Key ROESY correlations in **2**.

The coupling constant values (Table 1), as well as the ROESY correlations (Figure 3) between H-1, H-3, and H-9 and between H-2 and H-13 showed that the relative structure of **2** was the same as that of **1**.

An analysis of the literature data showed that the NMR spectra of compounds **1** and **2** were close to those for the known anthraquinones, dendryols A and D [8]. However, the values of chemical shifts and coupling constants of vicinal protons at C-1 and C-2 in the spectra of **1** and **2** significantly differed from those for known dendryols. Thus, compounds **1** and **2** are new stereoisomers of known dendryols A and D, respectively, and were named acruciquinones A (**1**) and B (**2**), respectively.

The molecular formula of compound **3** was determined as C_15_H_18_O_5_ based on the analysis of the (+)-HRESIMS spectrum data containing the peak of the cationized molecule [M + Na]^+^ (*m*/*z* 301.1042) and was confirmed by the ^13^C NMR data. The ^1^H and ^13^C NMR spectra of **3** (Table 2, Figures S19–S24) contain signals of a tetrasubstituted benzene ring; a methyl ring; two methylene groups, one of which is oxygenated; five methine groups, two of which are bonded to oxygen; four quaternary *sp*^2^-carbons; and one unsaturated ketone group.

**Figure 4:**
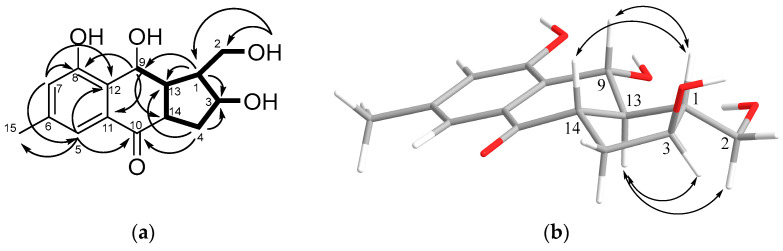
Key ^1^H–^13^C HMBC and ^1^H–^1^H COSY correlations (**a**) and ROESY correlations (**b**) of **3**.

HMBC correlations from H-5 (δ_H_ 7.25) to C-6 (δ_C_ 139.7), C-7 (δ_C_ 122.7), C-10 (δ_C_ 197.8), C-12 (δ_C_ 127.8), and C-15 (δ_C_ 21.0); from H-7 (δ_H_ 6.86) to C-5 (δ_C_ 118.9), C-8 (δ_C_ 158.3), C-12, and C-15; from H_3_-15 (δ_H_ 2.31) to C-5, C-6, and C-7; and from H-9 (δ_H_ 5.07) to C-8, C-11 (δ_C_ 134.7), C-12, C-13 (δ_C_ 54.7), and C-14 (δ_C_ 51.4) (Figure 4a and Figure S23) establish that the structure of rings A and B are the same as those for compounds **1** and **2**.

The observed ^1^H-^1^H COSY correlations (H-9/H-13/H-1(H_2_-2)/H-3/H_2_-4/H-14) and HMBC correlations from H-1 (δ_H_ 2.14) to C-2 (δ_C_ 62.9), C-3 (δ_C_ 73.1), C-9 (δ_C_ 74.5), and C-13; from H-4α (δ_H_ 1.98) to C-1, C-3, C-10, and C-14; and from H-14 to C-4, C-10, and C-13 revealed the structure of ring C, its fusion with ring B, and the position of hydroxymethyl and hydroxyl groups at C-1 and C-3, respectively (Figure 4a).

The relative configurations of **3** were established based on the ROESY correlations (Figure 4b and Figure S24): H-1/H-9, H-14, and H-13/H-2a, H-3.

Thus, the structure of compound **3** was determined and named acruciquinone C. It should be noted that acruciquinone C is the first and only representative of anthraquinone derivatives with a 6/6/5 framework.

**Figure 5:**
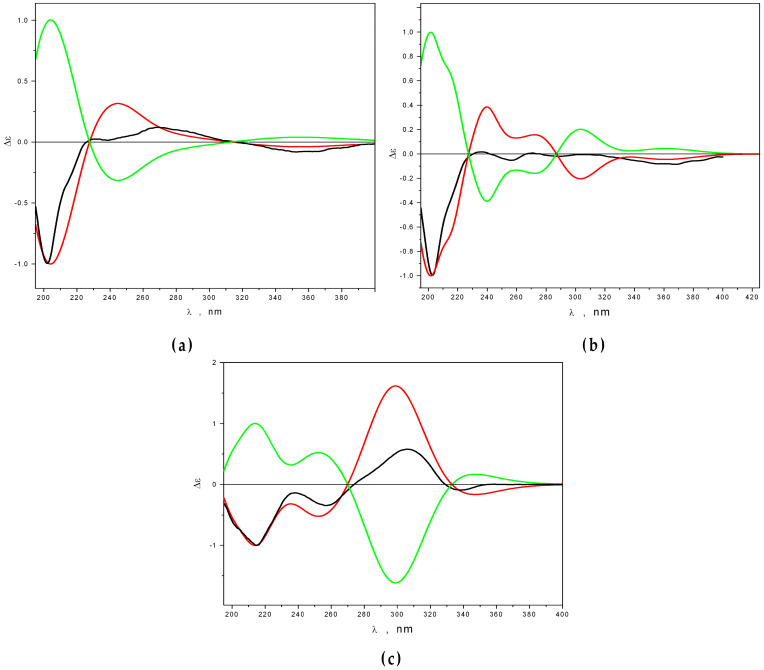
Calculated (red) and experimental (black) CD spectra for compounds **1** (**a**), **2** (**b**), and **3** (**c**). The green color ECD curves were calculated for enantiomers of compounds **1**–**3**.

The absolute configurations of **1**–**3** were determined using an approach based on a comparison of the ECD spectra recorded for these compounds (Figures S2, S4, and S6) with the theoretical spectra calculated for them using the B3LYP exchange–correlation functional and cc-pvTz basis set implemented in GAUSSIAN 16 software (Figure 5) [18].

The best agreement between Δε_exp_ and Δε_calc_ is achieved for **1**–**3** in the case of configurations 1*S*,2*S*,9*R*,13*S*, 1*S*,2*S*,3*R*,9*R*,13*S*, and 1*R*,3*S*,9*R*,13*S*,14*S*, respectively.

### 2.3. Bioactivity of Isolated Compounds

The effects of isolated anthraquinones on *Staphylococcus aureus* growth and the activity of its some enzymes were experimentally investigated. Moreover, the influence of antibacterial compounds on viability, migration, and proliferation of *S. aureus*-treated HaCaT keratinocytes was investigated. Compound **5** was isolated in an insufficient amount (1.0 mg) and was not investigated in any bioactivity tests. Compounds **2**, **3**, and **11** were isolated in very small amounts (0.9 mg, 1.5 mg, and 1.1 mg, respectively), so, only their influence on *S. aureus* growth was investigated.

#### 2.3.1. Antimicrobial Activity

**Figure 6:**
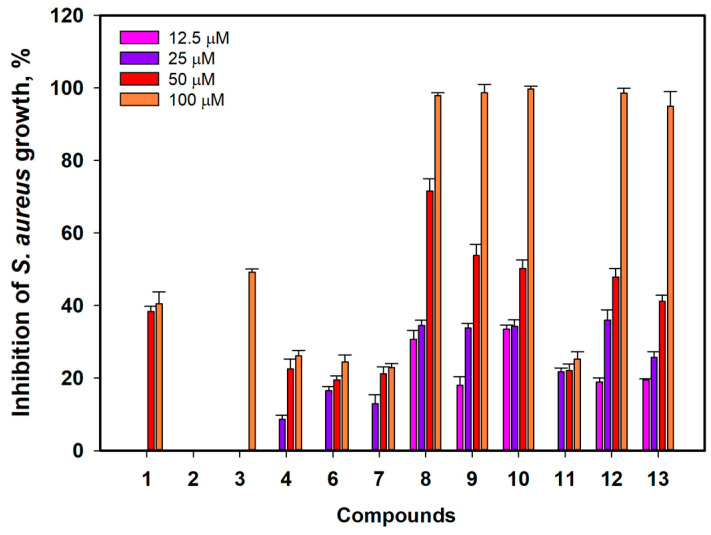
Antimicrobial activity against *Staphylococcus aureus* of compounds **1**–**4** and **6**–**13**. All experiments were carried out in triplicate. The data are presented as a mean ± standard error of mean (SEM).

The antimicrobial activity of compounds **1**–**4** and **6**–**13** against *Staphylococcus aureus* is presented in Figure 6.

Acruciquinone B (**2**) did not show any influence on *S. aureus* growth up to a concentration of 100 µM. Acruciquinone A (**1**) inhibited *S. aureus* growth by 38.4 ± 1.5% and 40.5 ± 3.3% at concentrations of 50 and 100 µM, respectively. Compounds **4**, **6**, **7**, and **11** showed inhibition of *S. aureus* growth near 30% at concentrations up to 100 µM.

The half-maximal concentration (IC_50_) of antistaphylococcal action was calculated for compounds **3**, **8**–**10**, **12**, and **13** (Table 3).

Compounds **8**-**10** showed the best effect on *S. aureus* growth, with calculated IC_50_ values of 35.4, 45.3, and 49.7 µM, respectively. Compounds **12** and **13** were less effective, with IC_50_ values of 52.1 and 58.2 µM, respectively. Acruciquinone C (**3**) had an IC_50_ near 100 µM.

Antimicrobial compounds can influence various aspects of bacterial life, including modification of environmental conditions via urease enzymes [19] or sortase A processing of the bacterial cell wall [20].

We investigated the effect of compounds **1**, **4**, **6**–**10**, **12**, and **13** on the activity of urease and sortase A from *S. aureus* in cell-free assays.

#### 2.3.2. Influence of Some Isolated Compounds on Urease Activity

**Figure 7:**
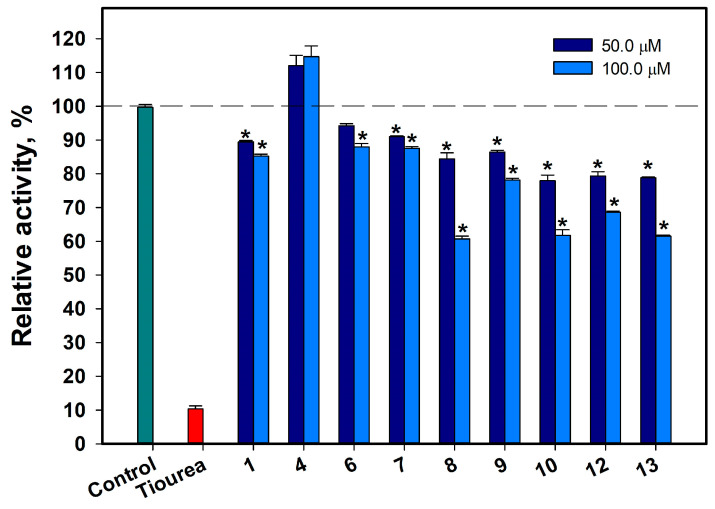
Effects of compounds **1**, **4**, **6**–**10**, and **12**–**13** on urease activity. Thiourea was used as a control. All experiments were carried out in triplicate. The data are presented as a mean ± standard error of mean (SEM). * Indicates significant differences between the control (DMSO) and compounds (*p* value ≤ 0.05).

Compounds **1**, **4**, **6**–**10**, **12**, and **13** were investigated as urease inhibitors, and only dendryol B (4) did not inhibit urease activity (Figure 7). The most significant effect was observed for compounds **8**, **10**, and **13**, which, at 100 µM, decreased the urease activity by 39.2%, 38.5%, and 38.3%, respectively, and, at a concentration of 50 µM, inhibited urease activity by 15.3%, 21.9%, and 21.2%, respectively. Compounds **12** and **9**, at 50 µM, decreased the urease activity by 20.3% and 13.5% and, at 100 µM, decreased urease activity by 31.6% and 21.8%, respectively. New acruciquinone A (**1**) decreased the urease activity by 10.7% and 14.6% at concentrations of 50 µM and 100 µM, respectively, and compounds **6** and **7** showed similar effects.

#### 2.3.3. Influence of Some Isolated Compounds on Sortase A Activity

**Figure 8:**
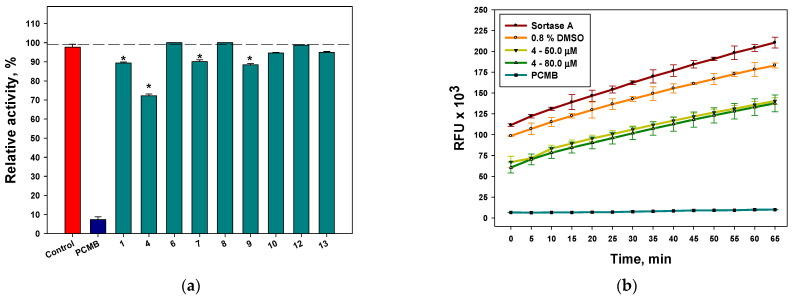
The effects of compounds **1**, **4**, **6**–**10**, and **12**–**13** on sortase A activity after 10 min of incubation (**a**) and time-dependent graph of inhibitory effect of dendryol B (**4**) (**b**). 4-(Hydroxymercuri)benzoic acid (PCMB) was used as a control. All experiments were carried out in triplicate. The data are presented as a mean ± standard error of mean (SEM). * Indicates significant differences between the control (DMSO 0.8%) and compounds (*p* value ≤ 0.05).

The effects of the investigated compounds on sortase A activity are presented in Figure 8a. Dendryol B (**4**) showed the most significant inhibitory effect on sortase A activity. It inhibited sortase A activity at concentrations of 50 µM and 80 µM by 27.6% and 32.1%, respectively, and its effect was stable during all periods of observation (Figure 8b).

Compounds **1**, **7**, and **9** had similar effects on sortase A activity, with significant inhibition of 14.7%, 6.3%, and 14.7%, respectively, at a concentration of 80 µM. The minimal effects of **10** and **13**, as well as those of **6** and **8**, were not statistically significant.

To detect the key structural moieties of anthraquinone derivatives for their inhibitory effect on sortase A, the molecular docking of compounds **1**, **4**, **5**, and **7**–**9** with sortase A was evaluated using fast online service SwissDock.

In the apo structure of sortase A (PDB ID 1T2P), a V-shaped pocket is formed by the β4, β7, and β8 strands on one side of the β barrel, together with three surrounding loops. The left side of the pocket is a hydrophobic tunnel formed by Ala92, Ala104, Ala118, Val161, Pro163, Val166, Val 168, Ile182, Val193, Trp194, Ile199, and Val201, along with two putative catalytic residues: Cys184 and Arg197 [21]. The right side of the pocket consists of several polar residues: Glu105, Asn114, Ser116, and Thr180. Earlier, the anthraquinone dimer skyrin N1287 was found as a sortase A inhibitor, and its complex with sortase A (PDB ID 1T2P) was investigated by molecular docking features. It was reported that skyrin, similar to curcumin, forms a hydrogen-bonding interaction or salt bridge with the guanidinium moiety of Arg197. N1287 and curcumin form extensive interactions with residues in the hydrophobic tunnel. In particular, the aromatic moiety from N1287 forms a cation-π interaction with Arg197. N1287 also forms hydrogen-bonding interactions with polar residues on the right side of the pocket, such as Asn114 and Ser116 [22].

In our calculations, the most active sortase A inhibitor, dendryol B (**4**), can form a pose (∆G −6.8640547 kkal/mol) with the hydrogen-bonding interactions between Arg197 and its 9-OH, Glu105 and 3-OH, Asn114 and keto-group C-10, and Gly167 and 8-OH. Moreover, hydrophobic interactions between **4** and Val168, Ile199, and Leu169 were detected. In the other side, the stable pose (∆G −6.800687) forms the hydrogen-binding interaction between Glu105 and 1-OH and the hydrophobic interactions between Cys184 and Me-15; Ala92 and C-7, H-7, Trp194, and Me-15; Ile182 and keto group C-10; and Ala118 and C-15 of **4**.

Acruciquinone A (**1**) can form complex ∆G −6.5611596 with hydrogen-bonding interactions between Arg197 and 2-OH of **1** and Ala92 and 1-OH, as well as with the hydrophobic interactions with Ala92, Gly192 (C-2, H-2), Ala104 (keto-group C-10), Ile182 (C-7 and H-7), Thr93 (1-OH), and Trp194 (H-1). Another pose was calculated (∆G −6.747401) with the hydrogen-bonding interactions with Arg197 (keto-group C-10) and the hydrophobic interactions with Cys184 (Me-15), Trp194 (Me-15), Ala104 (C-5 and C-6), and Leu169 (2-OH).

**Figure 9:**
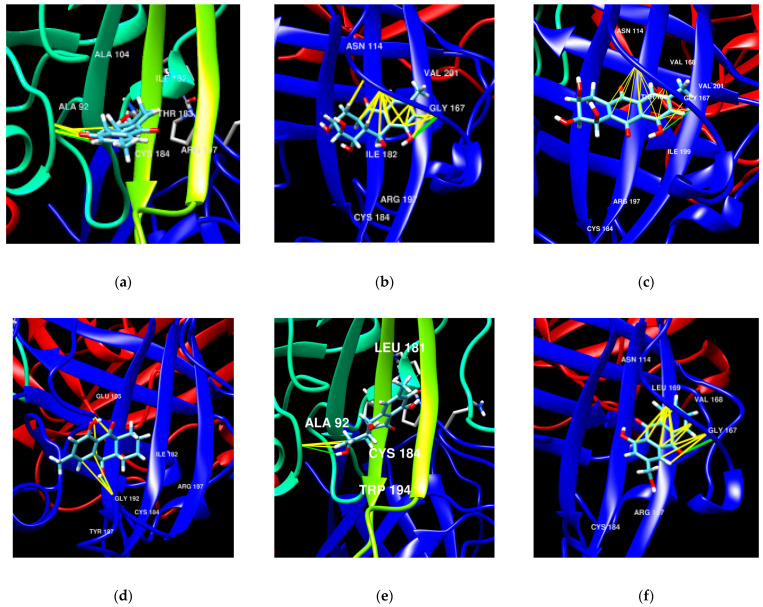
**Figure 9**. The molecular docking poses of some anthraquinones with sortase A (PDB ID 1T2P): **1** (**a**), **4** (**b**), **5** (**c**), **7** (**d**), **8** (**e**), and **9** (**f**).

Therefore, we can assume that the main differences in the structures of compounds **4** and **1** that influence their complexes with sortase A are the stereochemistry of the 9-OH group: the β orientation of 9-OH provides the opportunity to form a maximum number of interactions if both 9-OH and C(=O)-10 with sortase A (Figure 9).

Coniothyrinone B (**7**) can form complex ∆G −7.046784 with the hydrogen-bonding interactions with Arg197 and Gly192 and the hydrophobic interactions with Ile182, Trp194, Tyr187, Ala104, Gly192, and Thr93. Another complex (∆G −6.411958) has hydrogen-bonding interactions with Glu105 and hydrophobic interactions with Cys184, Trp194, Ala92, Leu97, and Ile182.

ω-Hydroxypachybasin (**9**) can form complex ∆G −6.769604 with the hydrogen-bonding interactions with Arg197, Asn114, and Gly167 and the hydrophobic interactions with Val168, Thr180, Ile199, Val166, Val201, and Gly167. The complex consists of hydrophobic interactions with Cys184 (as well as Ala92, Trp194, Thr93, and Ala104), whereas ∆G -6.2245307 does not have hydrogen-bonding interactions.

Rubrumol (**8**), which did not inhibit sortase A activity, can form complex ∆G −6.9308696 with the hydrogen-bonding interactions with Ala92 and the hydrophobic interactions with Cys184, Ala92, Trp194, and Ile182. Another complex (∆G −6.237157) has hydrogen-bonding interactions with Glu105 and Ala92 and hydrophobic interactions with Cys184, Ala92, and Ile182.

Therefore, compounds **1**, **4**, **7**, and **9**, which inhibited the activity of sortase A in a SensoLyte 520 Sortase A Activity Assay, may form the interactions with Arg197. No poses with the interactions with Arg197 were predicted for compound **8**. This observation confirms the conclusion about the significance of building with Arg197 for inhibition of sortase A’ activity by anthraquinones.

Pleosporone (**5**), which was not investigated in a SensoLyte 520 Sortase A Activity Assay, can form complex ∆G −6.9511595 with the hydrogen-bonding interactions with Arg197 (keto-group C-9), Asn114 (keto-group C-10), and Gly167 (8-OH) and the hydrophobic interactions with Val 166 (H-7), Val168 (aromatic ring A, C-9, C-10), Val201 (Me-15), Ile199 (C-6, C-7), Thr180 (C-5, C-10, C-11), and Gly167 (8-OH). Another calculated complex (∆G −6.2182164) has hydrogen-bonding interactions with Ala92 (1-OH, 2-OH) and hydrophobic interactions with Cys184 (3-OH), Ile182 (3-OH, H-4b, C-4), Ala92 (1-OH), and Trp194 (2-OH).

A comparison of these poses with complexes of **4** allows us to assume that pleosporone (**5**) may also act as an inhibitor of sortase A activity.

#### 2.3.4. Effects of Compounds on HaCaT Keratinocytes Infected with *Staphylococcus aureus*

Thus, the investigated secondary metabolites of *Asteromyces cruciatus* KMM4696 fungus can inhibit sortase A, especially urease enzyme activities, and affect *S. aureus* growth. However, it is advisable to study the effects of these anthraquinone derivatives in a co-culture of *S. aureus* with human cells before confidently talking about their real antibacterial potential. Therefore, the protective influence of compounds **1**, **4**, **7**, and **9** at a concentration of 10 μM on human HaCaT keratinocyte cells infected with *S. aureus* was experimentally investigated. Compound **6** did not show a significant effect on sortase A activity and had a small influence on urease activity and *S. aureus* growth; therefore, it was selected for in vitro investigation for comparison of its effect with that of **7**.

**Figure 10:**
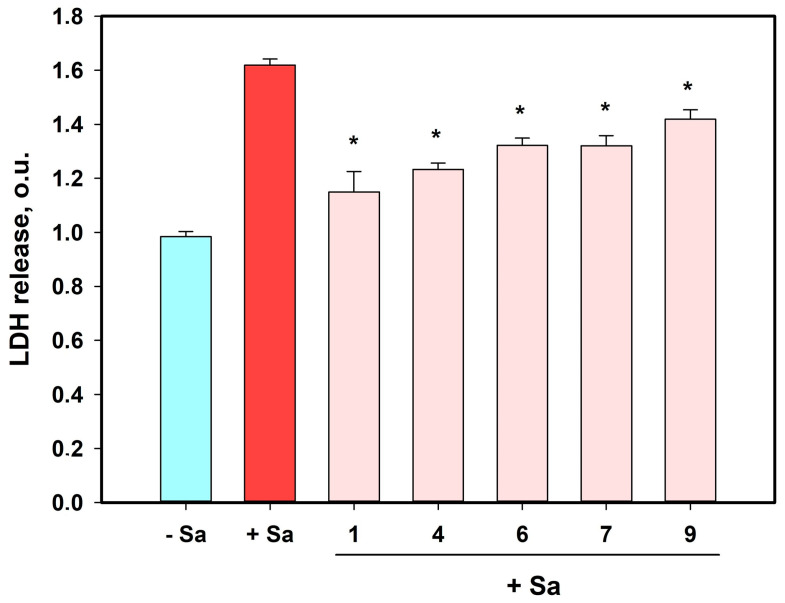
The effects of compounds **1**, **4**, **6**, **7**, and **9** on LDH release from HaCaT cells after infection with *S. aureus* (Sa) for 48 h. All compounds were used at a concentration of 10 µM. The experiments were carried out in triplicate. * Indicates statistically significant differences between S. aureus-infected cells and *S. aureus*-infected cells treated with compounds (*p* < 0.05).

*S. aureus* produces a number of lysing molecules causing the disruption of mammalian cells, so the release of lactate dehydrogenase (LDH) is used for detection of infected cell viability [23]. The effect of compounds **1**, **4**, **6**–**10**, **12**, and **13** on the LDH release from *S. aureus*-infected HaCaT cells is presented in Figure 10.

The incubation of HaCaT cells with *S. aureus* induced an increase in LDH release of 64.4%. All compounds investigated at a concentration of 10 µM showed significant effects on LDH release from staphylococci-infected HaCaT cells. After 48 h of coincubation, compounds **1**, **4**, **6**, **7**, and **9** caused statistically significant diminishments in LDH release from these cells of 29.4%, 23.8%, 18.3%, 18.4%, and 12.3%, respectively.

The effects of compounds **1**, **4**, **6**, **7**, and **9** on the proliferation of *S. aureus*-infected HaCaT cells were investigated using CFDA SE vital fluorescent dye and the flow cytometry technique described in [24]. The CFDA SE covalent builds with intracellular cytoplasm components, and its quantity (and intensity of fluorescence, respectively) in the cell decreases equivalent to the number of past divisions.

**Figure 11:**
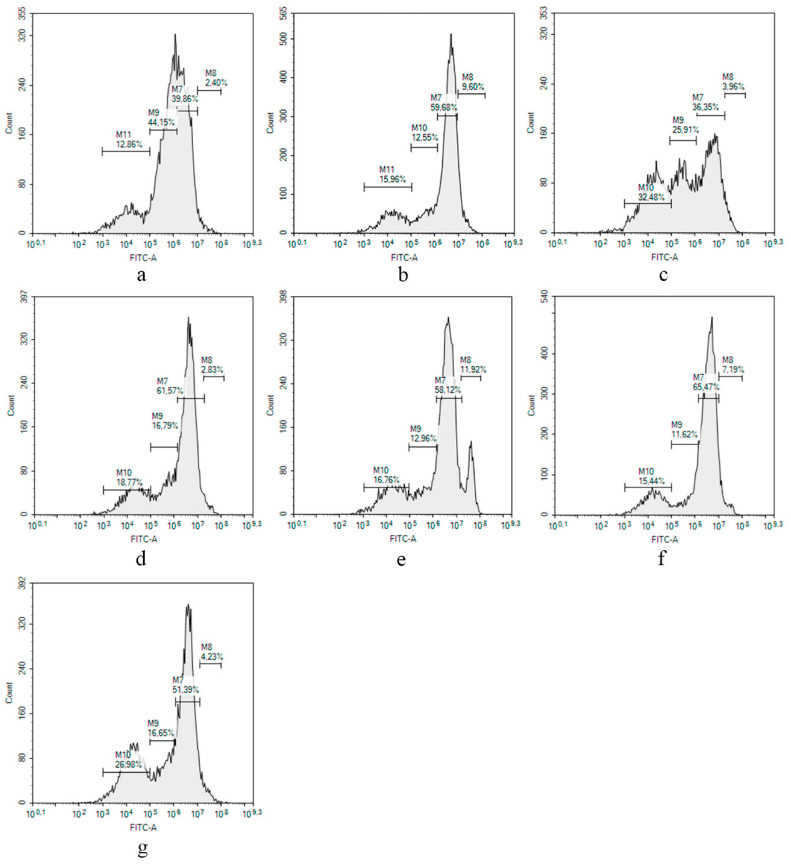
The proliferative profiles of non-treated HaCaT cells (**a**), *S. aureus*-infected HaCaT cells (**b**), and infected cells treated with compounds **1** (**c**), **4** (**d**), **6** (**e**), **7** (**f**), and **9** (**g**). All compounds were used at a concentration of 10 µM. The experiments were carried out in triplicate. The most representative picture for each case is presented.

The analysis of obtained flow cytometry data resulted in the detection of four HaCaT cell subpopulations (Figure 11a), and *S. aureus* infection significantly changed the ratio between them (Figure 11b). The percentage of each subpopulation is presented in Table 4.

The most noticeable change as a result of a staphylococcal infection was a change in the ratio between division 1 and division 2, which indicates a slowdown in HaCaT proliferation. Compounds **4**, **6**, and **7** did not show any observed changes in the picture (Figure 11d–f). Compound **9** induced a significant decrease in the amount of the cells in division 1 and an increase in the amount of cells in division 3. The most significant influence on infected HaCaT cells was observed for compound **1** (Figure 11c), which greatly increased the number of the cells in division 3 in comparison with infected and non-infected HaCaT cells.

**Figure 12:**
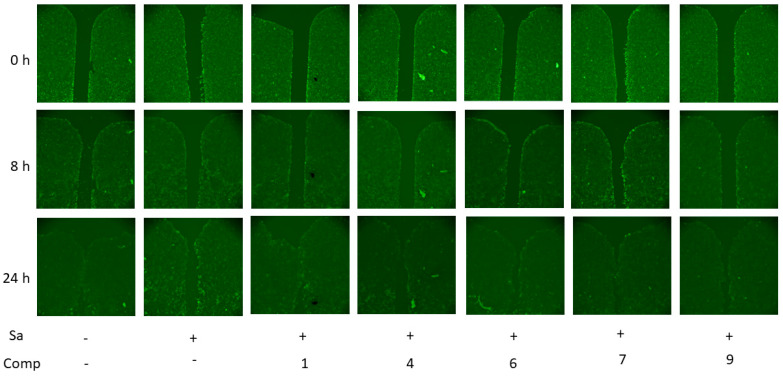
The effects of compounds **1**, **4**, **6**, **7**, and **9** on migration of *S. aureus* (Sa)-infected HaCaT cells. All compounds were used at a concentration of 10 µM. The experiments were carried out in triplicate. The most representative picture for each case is presented.

Finally, the effects of compounds **1**, **4**, **6**, **7**, and **9** on the migration of *S. aureus*-infected HaCaT cells were investigated (Figure 12). Manufacturing devices from Ibidi® were used for the creation of a cell-free zone in a monolayer of HaCaT cells stained with CFDA SE fluorescent dye, after which the *S. aureus* suspension and compounds were added and the cell migration to this cell-free zone was monitored by a fluorescent microscope for 24 h.

The first differences in cell position were detected after 8 h of observation, and full fusion of the cell-free zone in the non-infected cell layer was observed after 24 h. *S. aureus* infection inhibits fusion of this cell-free area, which was observed after 24 h. All investigated compounds improved migration of the *S. aureus*-infected cells in a cell-free zone. Complete confluence, similar to control cells, was observed for compound **1**, and compounds **6**, **9**, and especially **7** caused almost complete cell overgrowth of the cell-free zone. Compound **4** surprisingly showed the most incomplete fusion of the cell-free zone, but its positive effect was noticeable.

## 3. Discussion

### 3.1. Secondary Metabolites of Asteromyces cruciatus KMM4696

**Figure 13:**
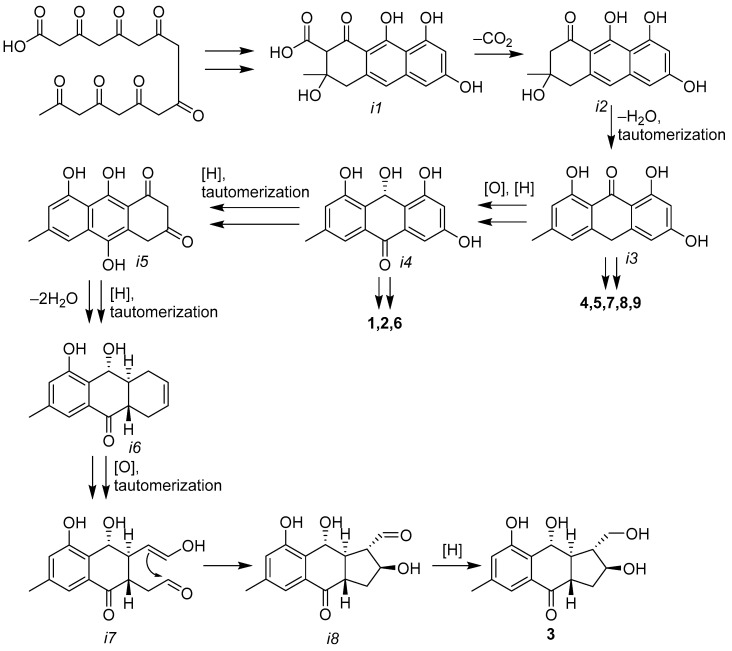
Plausible biogenetic pathway of acruciquinone C (**3**).

A biogenesis pathway for the framework of the novel acruciquinone C (**3**) has been proposed (Figure 13). It is obvious that the first steps of acruciquinone C biosynthesis are common to most fungal anthraquinones originating from the octaketide precursor [25]. The dehydration and tautomerization of intermediate *i2* result in anthrone *i3*, which is a plausible direct precursor of compounds **4**, **5**, and **7**–**9**. *i3* can also be sequentially oxidized and reduced to *i4*, from which compounds **1**, **2**, and **6** are most likely formed. Moreover, *i4* probably undergoes several reductions and tautomerizations, which, via diketone *i5*, lead to intermediate *i6* with monoene ring C [26]. Further oxidative cleavage of the double bond and tautomerization lead to *i7*, which, as a result of aldol condensation, turns into a direct precursor of acruciquinone C (**3**) with a 6/6/5 skeleton. Compound 3 is formed as a result of the reduction of aldehyde in *i8*.

Naphthalene derivative 12 was previously reported only as a synthetic compound [16]. This compound is undoubtedly a cyclization product of the linear hexaketide precursor.

Gliovictin (**13**), a diketopiperazine isolated from terrestrial fungi of the genera *Helminthosporium* and *Penicillium*, has been isolated from culture broths of the marine deuteromycete *Asteromyces cruciatus* [17].

It was previously shown that strain *A. cruciatus* KMM 4696 can produce the first chlorine-contained monocyclic cyclohexanols containing a 3-methylbutenynyl unit that obviously originated from a tetraketide precursor [7]. Benzopyranes **10** and **11** obviously originated from the same precursor. Thus, the *A. cruciatus* KMM 4696 fungal strain is a promising producer of structurally unique polyketides.

### 3.2. Biological Activity of Isolated Anthraquinone Derivatives

In our work, known dendryol B (**4**), rubrumol (**8**), trans-3,4-dihydroxy-3,4-dihydroanofinic acid (**10**), quadricinctapyran A (**11**), and gliovictin (**13**) were found as agents against *S. aureus* for the first time.

Dendryol B (**4**) was previously isolated from a weed pathogenic fungus, *Dendryphiella* sp., and caused necrotic events on barnyardgrass leaves [8]. Rubrumol (**8**) was assessed for cytotoxic activities against A549, MDA-MB-231, PANC-1, and HepG2 human cancer cell lines but displayed no significant cytotoxic activities. However, the authors showed the significant effect of **8** on the relaxation activity of topoisomerase I [11]. *Trans*-3,4-dihydroxy-3,4-dihydroanofinic acid (**10**) exhibited potent acetylcholinesterase-inhibitory activity [27]. The antimicrobial activity for quadricinctapyran A (**11**), which was not previously detected up to a concentration of 256 μg/mL [15], but the inhibition of *S. aureus* bacterial growth in microplates was estimated by visual observation only. The activity of gliovictin (**13**) against *Escherichia coli* and *Bacillus megaterium* was not observed [28], but it was tested in agar diffusion assays, which are subject to some limitations. In the present work, the antistaphylococcal activity of the compounds was tested using liquid broth titration with spectrophotometric detection, which can be crucial for detection of the effects of compounds.

Coniothyrinone D (**6**) and coniothyrinone B (**7**) were previously isolated from the culture of an endophytic fungus. *Coniothyrium* sp. They were studied as antimicrobials by the diffusion agar method and, their effects against Gram-positive *B. megaterium* were greater than their effects against Gram-negative *E. coli* [10]. The hydroxylated derivatives of coniothyrinone B (**7**), 8-hydroxyconiothyrinone B, 8,11-dihydroxyconiothyrinone B, 4R,8-dihydroxyconiothyrinone B, and 4S,8-dihydroxyconiothyrinone B, from marine algicolous fungus *Talaromyces islandicus* EN-501 showed pronounced activity against *S. aureus* EMBLC-2 growth [29]. Antistaphylococcal activity of ω-hydroxypachybasin (**9**) was reported when this compound was isolated from the plant *Ceratotheca triloba* [30].

In our work, we not only studied the influence of coniothyrinones B (**6**) and D (**7**) and ω-hydroxypachybasin (**9**) on *S. aureus* growth in detail but also their effects on sortase A and urease activity, as well as their potential for skin infection treatment for the first time.

### 3.3. Perspectives of Isolated Anthraquinones for the Treatment of Skin Infections

HaCaT keratinocytes cocultured with *S. aureus* are widely used in vitro models for antibiotic discovery, despite some limitations [23]. Our previously reported results showed that *S. aureus* infection caused HaCaT keratinocyte damage and cell cycle arrest in the G0/G1 phase [31] and resulted in inhibition of cell proliferation and migration, as observed in this work. The studied anthraquinones protect HaCaT cells from *S. aureus*-caused damage because a decrease in the LDH release from treated cells was detected. Moreover, one of the significant anthraquinones changes the proliferation profile and migration of *S. aureus*-infected HaCaT cells.

The various aspects of bacteria’s vital activity are the targets for antibiotics. Bactericidal antibiotics were targeted at a diverse set of biomolecules for inhibition to achieve cell death, including DNA topoisomerases (quinolones ciprofloxacin, levofloxacin, and gemifloxacin), RNA polymerase (rifamycin), penicillin-binding proteins, transglycosylases and peptidoglycan building blocks (*β*-lactam penicillins, carbapenems, cephalosporins, glycopeptides, vancomycin, fosfomycin, and daptomycin), and ribosomes (macrolides, lincosamides, streptogramins, and others) [32]. But these strategies have led to the emergence of resistant bacterial strains, which has become one of the major global public health problems [33].

Therefore, new strategies including inhibition of bacterial sortase A or urease activities have led to the discovery of new drugs to which developing resistance will be less possible. The sortase A enzyme was named an “ideal target” for the development of new anti-infective drugs [34] because it plays a significant role in the pathogenesis of Gram-positive bacteria. Sortase A is a bacterial cell membrane enzyme that anchors crucial virulence factors to the cell wall surface [35], and numerous studies have aimed to find new sortase A inhibitors [22,36]. The urease enzyme is able to do so by virtue of its ability to catalyze the conversion of urea into ammonia, thereby allowing bacterial colonies to live in acidic conditions. To date, according to Hameed and coauthors, only one commercial urease inhibitor, Lithostat (acetohydroxamic acid), is available, but it has a number of limitations [37]. Currently, urease inhibitors are considered mainly as potential leaders in urinary tract infections. However, a number of works indicate the promise of this approach for skin staphylococcal infections [19].

Our data point to the great importance of the structure of anthraquinones for the inhibition of sortase A activity. β-Orientation of the 9-OH group in the structure of dendryol B (**4**) makes its interaction with residues in the binding site the most effective.

In the case of urease inhibition, the differences between the action of all the studied anthraquinones are insignificant, which does not allow us to discuss their structure–activity relationship. The highest activity was found for an alkaloid, i.e., gliovictin (**13**). Recently, a large number of sulfur- and nitrogen-containing compounds have been described as urease inhibitors [38]. Obviously, it is precisely the thiodiketopiperazine moiety of gliovictin that makes it interesting for further study against *Helicobacter pylori* and other urease-producing bacteria.

However, the effect on bacterial growth or enzyme activities does not yet mean that substances will be active in real infections, since an infection model is a more complex and multicomponent system. In this regard, the study of the effects of promising compounds in in vitro infection models can lead to unexpected results, as we see here. In our experiments, dendryol B (**4**) exhibited the greatest inhibition of sortase A activity, with a weak effect on *S. aureus* growth, but its effects in coculture experiments were not so great. In contrast, acruciquinone A (**1**) showed a weak (yet noticeable) inhibition of sortase A and urease activity and a moderate effect on *S. aureus*, but this new metabolite from *Asteromyces cruciatus* was the most effective against *S. aureus*-caused HaCaT cell damage and in a skin wound model. ω-Hydroxypachybasin (**9**) exhibited the most significant effect against *S. aureus* growth and a weak inhibition of urease and sortase A activities but showed the least pronounced protection against HaCaT damage, as well as coniothyrinones D (**6**) and B (**7**).

Thus, the protection of *S. aureus*-infected HaCaT keratinocytes by acruciciquinone A (**1**) is due to both its direct antibacterial action and the effect on the keratinocytes themselves.

## 4. Materials and Methods

### 4.1. General Experimental Procedures

Optical rotations were measured on a Perkin-Elmer 343 polarimeter (Perkin Elmer, Waltham, MA, USA). UV spectra were recorded on a Shimadzu UV-1601PC spectrometer (Shimadzu Corporation, Kyoto, Japan) in methanol. CD spectra were measured with a Chirascan-Plus CD spectrometer (Leatherhead, UK) in methanol. NMR spectra were recorded in CDCl_3_, acetone-*d_6_*, and DMSO-*d_6_* on a Bruker DPX-300 (Bruker BioSpin GmbH, Rheinstetten, Germany), a Bruker Avance III-500 (Bruker BioSpin GmbH, Rheinstetten, Germany), and a Bruker Avance III-700 (Bruker BioSpin GmbH, Rheinstetten, Germany) spectrometer. A calibration of NMR spectra was carried out using the residual solvent signals (7.26/77.16 for CDCl_3_ and 2.05/29.84 for acetone-d_6_ according to [39]). HRESIMS spectra were measured on a Maxis impact mass spectrometer (Bruker Daltonics GmbH, Rheinstetten, Germany). Microscopic examination and photography of fungal cultures were performed with an Olympus CX41 microscope equipped with an Olympus SC30 digital camera. Detailed examination of the ornamentation of the fungal conidia was performed using an EVO 40 scanning electron microscope (SEM).

Low-pressure liquid column chromatography was performed using silica gel (50/100 μm, Imid Ltd., Krasnodar, Russia) and Gel ODS-A (12 nm, S—75 um, YMC Co., Ishikawa, Japan). Plates precoated with silica gel (5–17 μm, 4.5 cm × 6.0 cm, Imid Ltd., Russia) and silica gel 60 RP-18 F_254_S (20 cm × 20 cm, Merck KGaA, Darmstadt, Germany) were used for thin-layer chromatography. Preparative HPLC was carried out on an Agilent 1100 chromatograph (Agilent Technologies, Santa Clara, CA, USA) with an Agilent 1100 refractometer (Agilent Technologies, Santa Clara, CA, USA) and a Shimadzu LC-20 chromatograph (Shimadzu USA Manufacturing, Canby, OR, USA) with a Shimadzu RID-20A refractometer (Shimadzu Corporation, Kyoto, Japan) using YMC ODS-AM (YMC Co., Ishikawa, Japan) (5 µm, 10 mm × 250 mm), YMC ODS-AM (YMC Co., Ishikawa, Japan) (5 µm, 4.6 mm × 250 mm), and Fusion Hydro-RP (Phenomenex, Torrance, CA, USA) (4 μm, 250 mm × 10 mm) columns.

### 4.2. Fungal Strain

The strain of the obligate marine fungus *Asteromyces cruciatus* KMM 4696 was isolated from the surface of the thallus of the brown alga *Sargassum pallidum* (Sea of Japan) and identified using morphological and molecular genetic features [7]. The fungal strain is stored in the Collection of Marine Microorganisms (KMM) of PIBOC FEB RAS (Vladivostok, Russia).

### 4.3. Cultivation of Fungus

*A. cruciatus* fungus was cultured on a rice medium at 22 °C for three weeks in 100 Erlenmeyer flasks (500 mL), each containing 20 g of rice, 20 mg of yeast extract, 10 mg of KH2PO4, and 40 mL of natural seawater from the Marine Experimental Station of PIBOC FEB RAS, Troitsa (Trinity) Bay, Sea of Japan.

### 4.4. Extraction and Isolation

At the end of the incubation period, the mycelium of the *Asteromyces cruciatus* KMM 4696 fungus, together with the medium, was homogenized and extracted with EtOAc (2 L). The obtained extract was concentrated to dryness. The dry residue (7.9 g) was dissolved in a H_2_O−EtOH (4:1) system (200 mL) and extracted successively with *n*-hexane (3 × 0.2 L) and EtOAc (3 × 0.2 L). The ethyl acetate extract was evaporated to dryness (5.3 g) and chromatographed on a silica gel column (3 × 14 cm), which was first eluted with *n*-hexane (200 mL), then with a stepwise gradient of 5% to 50% EtOAc in *n*-hexane (total volume 20 L). Fractions of 250 ml were collected and combined based on TLC data.

The fractions eluted with *n*-hexane-EtOAc (95:5, 80 mg) and *n*-hexane-EtOAc (90:10, 200 mg) were combined and separated on a YMC ODS-A reverse-phase column (1.5 × 5.5 cm), which was eluted with a step gradient from 40% to 80% MeOH in H_2_O (total volume: 1 L) to afford subfractions I and II. Subfraction I (40% MeOH, 146 mg) was separated by reverse-phase HPLC on a YMC ODS-A column, eluting first with MeOH–H_2_O (90:10) to afford two subfractions: I-1 and I-2. Subfraction I-1 was rechromatographed on a YMC ODS-A column eluting with MeOH–H_2_O (55:45) to **13** (4.8 mg). Subfraction I-2 was rechromatographed on an Ultrasfera Si column eluting with *n*-hexane–ethyl acetate (60:40) to **11** (1.1 mg). Subfraction II (60% MeOH, 110 mg) was separated by reverse-phase HPLC on a YMC ODS-AM column eluting with MeOH–H_2_O (80:20), then with MeOH–H_2_O (55:45) to **9** (4 mg) and **12** (21.6 mg).

The fraction of *n*-hexane-EtOAc (80:20, 470 mg) was separated on a Gel ODS-A column (1.5 × 5.5 cm), which was eluted with a step gradient from 40% to 80% MeOH in H_2_O (total volume 1 L) to afford subfraction III. Subfraction III (40% MeOH, 250 mg) was separated by reverse-phase HPLC on a YMC ODS-A column, eluting with MeOH–H_2_O (90:10), then with MeOH–H_2_O (60:40) and MeCN–H_2_O (60:40) to **1** (4.8 mg), **5** (1.0 mg), **8** (2.2 mg), and **10** (6.4 mg).

The *n*-hexane-EtOAc fraction (70:30, 580 mg) was separated on a column with a reverse-phase sorbent YMC ODS-A (1.5 × 5.5 cm), which was eluted with a step gradient from 40% to 100% MeOH in H_2_O (total volume 1 L) to subfractions IV and V. Subfraction IV (40% MeOH, 390 mg) was separated by reverse-phase HPLC on a YMC ODS-A column eluting with MeOH–H_2_O (95:5), then with MeOH–H_2_O (70:30) to afford compounds **3** (1.5 mg), **6** (4.6 mg), and **7** (3 mg). Subfraction V (100% MeOH, 40 mg) was separated by reverse-phase HPLC on a YMC ODS-A column eluting with MeOH-H_2_O (55:45), then with CH_3_CN-H_2_O (25:75) to **2** (0.9 mg) and **4** (2.7 mg).

### 4.5. Spectral Data

Acruciquinone A (**1**): amorphous solids; [α]_D_^20^ −92.0 (*c* 0.1 MeOH); UV (MeOH) *λ*_max_ (log *ε*) 335 (3.14), 285 (3.88), 198 (4.24) nm (see Supplementary Figure S1); CD (*c* 9.6 × 10^−4^, MeOH), λ_max_ (∆ε) 202 (−16.07), 232 (0.35), 269 (1.94), 351 (−1.30) nm (see Supplementary Figure S2); for ^1^H and ^13^C NMR data, see Table 1 and Supplementary Figures S7–S12; HRESIMS *m*/*z* 275.0914 [M − H]^−^ (calcd. for C_15_H_15_O_5_, 275.0925), 299.0887 [M + Na]^+^ (calcd. for C_15_H_16_O_5_Na, 299.0890) (see Figure S87).

Acruciquinone B (**2**): amorphous solids; [α]_D_^20^ −121.4 (*c* 0.07 MeOH); UV (MeOH) *λ*_max_ (log *ε*) 334 ( ), 283 (3.76), 198 (4.09) nm (see Supplementary Figure S3); CD (*c* 9.6 × 10^−4^, MeOH), λ_max_ (∆ε) 203 (−10.73), 256 (−0.56), 288 (−0.21), 366 (−0.91) nm (see Supplementary Figure S4); for ^1^H and ^13^C NMR data, see Table 1 and Supplementary Figures S13–S18; HRESIMS *m*/*z* 291.0882 [M − H]^−^ (calcd. for C_15_H_15_O_6_, 291.0874), 315.0830 [M + Na]^+^ (calcd. for C_15_H_16_O_6_Na, 315.0839) (see Figure S88).

Acruciquinone C (**3**): amorphous solids; [α]_D_^20^ −58.0 (*c* 0.10 MeOH); UV (MeOH) *λ*_max_ (log *ε*) 315 (3.55), 261 (3.86), 213 (4.96) nm (see Supplementary Figure S5); CD (*c* 9.6 × 10^−4^, MeOH), λ_max_ (∆ε) 215 (−10.42), 257 (−3.62), 306 (6.04), 339 (−0.95) nm (see Supplementary Figure S6); for ^1^H and ^13^C NMR data, see Table 2 and Supplementary Figures S19–S24; HRESIMS *m*/*z* 277.1087 [M − H]^−^ (calcd. for C_15_H_17_O_5_, 277.1081), 301.1042 [M + Na]^+^ (calcd. for C_15_H_18_O_5_Na, 301.1046) (see Figure S89).

### 4.6. Quantum Chemical Calculations

The quantum chemical calculations for compounds **1**–**3** in methanol were performed using exchange–correlation functional B3LYP, the polarization continuum model (PCM), and the cc-pvTz basis set implemented in the Gaussian 16 package of programs [18]. Conformations with relative Gibbs free energies in the range of Δ*G_im_* ≤ 5 kcal/mol were chosen for calculations of the UV and ECD spectra at the B3LYP/cc-pvTz_PCM//B3LYP/cc-pvTz_PCM level of theory. The statistical weights of conformations are:

gim=e−ΔGimRT∑ie−ΔGimRT

where *T* = 298.15 K, and the subscript “*m*” denotes conformation, for which *G* is minimal.

Each individual transition from electronic ground state to the *i*-th calculated excited electronic state (1 ≤ *i* ≤ 55) was simulated as a Gauss-type function. The bandwidths taken at 1/e peak heights were chosen to be *σ* = 0.34 eV for **1** and **3** and 0.24 eV for **2**. The UV shifts taken for simulations of spectra are Δ*λ* = 0 nm for **1** and **2** and Δ*λ* = −7 nm for **3**.

The scaled theoretical and experimental ECD spectra were obtained according to the following equation:

Δεscaledλ=ΔελΔελpeak

where the denominator (|Δ*ε*(*λ_peak_*)|) is a modulo of the peak value for the chosen characteristic negative band in corresponding ECD spectra (200 ≤ *λ_peak_* ≤ 220 nm).

### 4.7. Sortase A Activity Inhibition Assay

The enzymatic activity of sortase A from *Staphylococcus aureus* was determined using a SensoLyte 520 Sortase A Activity Assay Kit * Fluorimetric * (AnaSpec AS-72229, AnaSpec, San Jose, CA, USA) in accordance with the manufacturer’s instructions. The compounds were dissolved in DMSO and diluted with reaction buffer to obtain a final concentration of 0.8% DMSO, which did not affect enzyme activity. DMSO at a concentration of 0.8% was used as a control. 4-(Hydroxymercuri)benzoic acid (PCMB) was used as sortase A enzyme activity inhibitor. Fluorescence was measured with a PHERAStar FS plate reader (BMG Labtech, Offenburg, Germany) for 60 min, with a time interval of 5 min. The data were processed with MARS Data Analysis v. 3.01R2 (BMG Labtech, Offenburg, Germany). The results are presented as relative fluorescent units (RFUs) and percentage of the control data and were calculated using STATISTICA 10.0 software.

### 4.8. Urease Inhibition Assay

A reaction mixture consisting of 25 µL enzyme solution (urease from *Canavalia ensiformis*, Sigma, 1U final concentration) and 5 µL of test compounds dissolved in water (10–300.0 µM final concentration) was preincubated at 37 °C for 60 min in 96-well plates. Then, 55 µL of phosphate buffer solution with 100 µM urea was added to each well and incubated at 37 °C for 10 min. The urease-inhibitory activity was estimated by determining ammonia production using the indophenol method. Briefly, 45 µL of phenol reagent (1% *w*/*v* phenol and 0.005% *w*/*v* sodium nitroprusside) and 70 µL of alkali reagent (0.5% *w*/*v* NaOH and 0.1% active chloride NaClO) were added to each well. The absorbance was measured after 50 min at 630 nm using a MultiskanFS microplate reader (Thermo Scientific Inc., Beverly, MA, USA). All reactions were performed in triplicate in a final volume of 200 µL. The pH was maintained at 7.3–7.5 in all assays. DMSO 5% was used as a positive control.

### 4.9. Antimicrobial Activity

The bacterial culture of *Staphylococcus aureus* ATCC 21027 (Collection of Marine Microorganisms PIBOC FEBRAS) was cultured in a Petri dish at 37 °C for 24 h on solid Mueller Hinton broth medium with agar (16.0 g/L).

The assays were performed in 96-well microplates in appropriate Mueller Hinton broth. Each well contained 90 µL of bacterial suspension (10^9^ CFU/mL). Then, 10 µL of a compound diluted at concentrations from 1.5 µM to 100.0 µM using twofold dilution was added (DMSO concentration < 1%). Culture plates were incubated overnight at 37 °C, and the OD_620_ was measured using a MultiskanFS spectrophotometer (Thermo Scientific Inc., Beverly, MA, USA). Gentamicin was used as a positive control at a concentration of 1 mg/mL, and 1% DMSO solution in PBS was used as a negative control. The results were calculated as a percentage of the control data by SigmaPlot 14.0 software.

### 4.10. Cell Line and Culture Conditions

The human HaCaT keratinocyte cell line was kindly provided by Prof. N. Fusenig (Cancer Research Centre, Heidelberg, Germany). All cells had a passage number ≤ 30. The cells were incubated in humidified 5% CO_2_ at 37 °C in DMEM medium (BioloT, St. Petersburg, Russia) containing 10% FBS and 1% penicillin/streptomycin (BioloT, St. Petersburg, Russia).

### 4.11. Cocultivation of HaCaT Cells with S. aureus and Lactate Dehydrigenase Release Test

HaCaT cells at a concentration of 1.5 × 10^4^ cells per well were seeded in 96-well plates for 24 h. Then, a culture medium in each well was changed with *S. aureus* suspension (10^2^ CFU/mL) in full DMEM medium. Fresh DMEM medium without *S. aureus* suspension was added to other wells as needed. The compounds at a concentration of 10 μM were added to wells after 1 h, and HaCaT cells and *S. aureus* were cultured at 37 °C in a humidified atmosphere with 5% (*v*/*v*) CO_2_ for 48 h.

After incubation, the plate was centrifuged at 250× *g* for 10 min, and 50 µL of supernatant from each well was transferred into the corresponding wells of an optically clear 96-well plate. An equal volume of the reaction mixture (50 µL) from an LDH Cytotoxicity Assay Kit (Abcam, Cambridge, UK) was added to each well and incubated for up to 30 min at room temperature. The absorbance of all samples was measured at λ = 450 nm using a Multiskan FC microplate photometer (Thermo Scientific, Waltham, MA, USA) and expressed in optical units (o.u.).

### 4.12. Migration of HaCaT Cells Cocultivated with S. aureus

The silicon 2-well inserts (Ibidi®, Gräfelfing, Germany) were placed in the center of wells in a 24-well plate, and HaCaT cell suspension was added to each well for 24 h. After adhesion, the inserts were removed, and the cells were labeled with (5,6)-carboxyfluorescein succinimidyl ester (CFDA SE) dye (LumiTrace CFDA SE kit, Lumiprobe, Moscow, Russia). CFDA SE stock solution at 5 mM in DMSO was dissolved in PBS for preparation of a 10 µM solution. The cell culture medium was replaced with this CFDA SE solution for 5 min at 37 °C. Then, the cells were washed twice with PBS, and *S. aureus* suspension (10^2^ CFU/mL) in full DMEM medium was added to each well as necessary. The medium without bacteria was added to control wells. The compounds at a concentration of 10 μM were added to wells after 1 h, and HaCaT cells and *S. aureus* were cultured at 37 °C in a humidified atmosphere with 5% (*v*/*v*) CO_2_.

The silicon 2-well inserts from Ibidi® formed cell-free zones and migration of HaCaT cells in these zones were observed using an MBF-10 fluorescent microscope (Lomo Microsystems, St.-Peterburg, RF, Russia) during 30 h of incubation.

### 4.13. Proliferation of HaCaT Cells Cocultivated with S. aureus

The HaCaT cells at a concentration of 1.5 × 10^4^ were seeded in a 12-well plate for 24 h. After adhesion, the cells were strained with (5,6)-carboxyfluorescein succinimidyl ester (CFDA SE) dye (LumiTrace CFDA SE kit, Lumiprobe, Moscow, Russia). CFDA SE stock solution at 5 mM in DMSO was dissolved in PBS for preparation of a 10 µM solution. The cell culture medium was replaced with this CFDA SE solution for 5 min at 37 °C. Then, the cell layer was washed with PBS twice, an *S. aureus* suspension (10^2^ CFU/mL) in full DMEM medium was added to each need well, and after 1 h, the compound at a concentration of 10 µM was added to the wells. The medium without bacterial suspension was added to the control well.

After 48 h of incubation, the cells were washed with PBS twice, scrabbed, and collected in 1.5 mL tubes. The intensity of CFDA fluorescence was analyzed with a NovoCyte flow cytometer (Agilent, Austin, TX, USA).

### 4.14. Molecular Docking

The pdb file of sortase A (PDB ID 1T2P) was obtained from the RCSB Protein Data Bank (https://www.rcsb.org accessed on 25 July 2023) and prepared for docking by the PrepDock package of UCFS Chimera 1.16 software. The chemical structures of ligands were prepared for docking by ChemOffice and checked by the PrepDock package of UCFS Chimera 1.16 software.

Docking was conducted on the SwissDock online server (http://www.swissdock.ch accessed on 25 July 2023) based on EADock DSS docking software [40]. The algorithm implies the generation of many binding modes in the vicinity of all target cavities (blind docking) and estimation of their CHARMM energies via the Chemistry at HARvard Macromolecular Mechanics (CHARMM) algorithm [41] for evaluation of the binding modes with the most favorable energies with FACTS (Fast Analytical Continuum Treatment of Solvation) [42] and, finally, clustering of these binding modes [43].

The predicted building models for each target/ligand pair were visualized and analyzed by UCFS Chimera 1.16 software. Docking parameters such as Gibb’s free energy (ΔG, kcal/mol), FullFitness score (FF, kcal/mol), and hydrogen-bonding (H-bond) and hydrophobic interactions were used for analysis of target/ligand complexes.

### 4.15. Statistical Data Evaluation

All data were obtained in three independent replicates, and calculated values are expressed as a mean ± standard error mean (SEM). Student’s *t*-test was performed using SigmaPlot 14.0 (Systat Software Inc., San Jose, CA, USA) to determine statistical significance. Differences were considered statistically significant at *p* < 0.05.

## 5. Conclusions

The *Asteromyces cruciatus* KMM4696 fungal strain is a promising producer of structurally unique and antibacterial polyketides. New acruciquinone C (**3**) possessed an unprecedented 6/6/5 anthraquinone-derived skeleton. The effect of new acruciquinone A (**1**) and known dendryol B (**4**) on sortase A activity and their weak antimicrobial effects indicate their potential antivirulence properties, with a reduced risk of antimicrobial resistance, made both these compounds very interesting as antivirulence agents. Their effects against *Staphylococcus aureus* in coculture with human HaCaT keratinocytes conditioned inhibition of sortase A and urease activity but did not limit inhibition, which ensures their positive effect on migration and proliferation of infected keratinocytes.

## Figures and Tables

**Table 1:** ^1^H NMR spectroscopic data (acetone-d_6_, *δ* in ppm, *J* in Hz) for **1** and **2**.

Position	1		2	
	δ_C_, mult	δ_H_ (*J* in Hz) ^a^	δ_C_, mult	δ_H_ (*J* in Hz) ^b^
1	78.9, CH	3.99, t (9.0)	77.9, CH	4.09, t (9.5)
2	70.1, CH	3.81, m	77.8, CH	3.61, dd (9.6, 8.4)
3	34.7, CH_2_	α: 2.76, m β: 2.30, m	72.8, CH	4.30, m
4	137.1, CH	6.98, m	139.6, CH	6.85, t (2.7)
5	120.1, CH	7.35, d (1.3)	120.1, CH	7.36, d (1.2)
6	140.0, C	-	140.1, C	-
7	123.3, CH	6.87, d (1.3)	123.6, CH	6.89, d (1.2)
8	157.4, C		157.6, C	
9	75.0, CH	5.37, d (9.8)	74.9, CH	5.39, d (9.9)
10	184.1, C	-	184.3, C	-
11	132.9, C	-	132.8, C	-
12	125.5, C	-	125.5, C	-
13	48.5, CH	2.96, m	48.6, CH	3.02, m
14	132.2, C	-	130.9, C	-
15	21.0, CH_3_	2.30, s	21.0, CH_3_	2.31, s

^a^ Chemical shifts were measured at 500.13 MHz. ^b^ Chemical shifts were measured at 700.13 MHz.

**Table 2:** ^1^H and ^13^C NMR spectroscopic data (δ in ppm, 700.13 /125.75 MHz, acetone-d_6_) for **3**.

Pos.	δ_C_, Mult	δ_H_ (J in Hz)
1	54.4, CH	2.14, m
2	62.9, CH_2_	3.76, t (10.3) 4.13, dd (10.7, 2.8)
3	73.1, CH	4.47, brs
4	35.0, CH_2_	α: 1.98, ddd (14.3, 9.5, 2.9) β: 2.35, m
5	118.9, CH	7.25, s
6	139.7, C	-
7	122.7, CH	6.86, s
8	158.3, C	-
9	74.5, CH	5.07, d (9.4)
10	197.8, C	-
11	134.7, C	-
12	127.8, C	-
13	54.7, CH	2.37, m
14	51.4, CH	2.69, dt (13.6, 9.2)
15	21.0, CH_3_	2.31, s
2-OH	-	5.31, brs
3-OH	-	3.82, d (4.7)
8-OH	-	9.36, brs
9-OH	-	7.31, brs

**Table 3:** The calculated half-maximal (IC_50_) effect of compounds on *S. aureus* growth.

Compound	3	8	9	10	12	13
IC_50_, µM	≥100	35.4 ± 1.5	45.3 ± 3.1	49.7 ± 2.4	52.1 ± 1.3	58.2 ± 1.7

**Table 4:** The effects of compounds on proliferation of *S. aureus*-infected HaCaT cells.

Sample ^1^	Division 0, % of Total Amount	Division 1, % of Total Amount	Division 2, % of Total Amount	Division 3, % of Total Amount
HaCaT cells	3.0 ± 0.8	37.7 ± 3.0	43.2 ± 1.4	15.5 ± 3.7
*S. aureus*	8.7 ± 1.2	58.2 ± 2.0	16.8 ± 6.0	14.9 ± 1.5
**1**	3.6 ± 0.5	34.0 ± 3.3	25.9 ± 0.4	35.5 ± 4.2
**4**	3.1 ± 0.4	63.2 ± 2.3	15.4 ± 1.9	17.6 ± 1.7
**6**	8.6 ± 4.7	60.5 ± 3.4	12.1 ± 1.2	18.1 ± 2.0
**7**	7.2 ± 2.1	65.5 ± 6.4	11.6 ± 1.3	15.4 ± 5.6
**9**	3.4 ± 1.2	46.3 ± 7.2	18.3 ± 6.5	30.2 ± 4.5

^1^ All compounds were used at a concentration of 10 µM. The experiments were carried out in triplicate, and the percentage of each HaCaT cell subpopulation is presented as mean ± standard error of mean.

## Data Availability

The original data presented in the study are included in the article/Supplementary Material; further inquiries can be directed to the corresponding author.

## Supplementary Materials

The following are available online at https://www.mdpi.com/article/10.3390/md21080431/s1, Figures S1–S6: ECD and UV spectra of compounds **1**–**3**; Figures S7–S90: NMR spectra of compounds **1**–**13**; Figures S91–S104: HR ESI MS spectra of compounds **1**–**3** and **6**–**13**.
