# Supplementary material for: Anthraquinone Derivatives and Other Aromatic Compounds from Marine Fungus Asteromyces cruciatus KMM 4696 and Their Effects against Staphylococcus aureus
Source: Mar Drugs. 2023 Jul 29;21(8):431. doi: 10.3390/md21080431 (PMC10455474; doi:10.3390/md21080431)

SUPPLEMENTARY MATERIALS

# Anthraquinone derivatives and other aromatic compounds from marine fungus *Asteromyces cruciatus* KMM 4696 and their effects against *Staphylococcus aureus*

# Content


Figure S1. UV spectrum of **1** ....... 5

Figure S2. CD spectrum of **1** ....... 5

Figure S3. UV spectrum of **2** ....... 6

Figure S4. CD spectrum of **2** ....... 6

Figure S5. UV spectrum of **3** ....... 7

Figure S6. CD spectrum of **3** ....... 7

Figure S7. $^{1}$H NMR spectrum (500 MHz, acetone-d$_6$) of **1** ....... 8

Figure S8. $^{13}$C NMR spectrum (125.75 MHz, acetone-d$_6$) of **1** ....... 9

Figure S9. COSY-45 spectrum (500 MHz, acetone-d$_6$) of **1** ....... 10

Figure S10. HSQC spectrum (500 MHz, acetone-d$_6$) of **1** ....... 11

Figure S11. HMBC spectrum (500 MHz, acetone-d$_6$) of **1** ....... 12

Figure S12. ROESY spectrum (700 MHz, acetone-d$_6$) of **1** ....... 13

Figure S13. $^{1}$H NMR spectrum (500 MHz, acetone-d$_6$) of **2** ....... 14

Figure S14. $^{13}$C NMR spectrum (125.75 MHz, acetone-d$_6$) of **2** ....... 15

Figure S15. DEPT-135 spectrum (125.75 MHz, acetone-d$_6$) of **2** ....... 16

Figure S16. COSY-45spectrum (700 MHz, acetone-d$_6$) of **2** ....... 17

Figure S17. HSQC spectrum (700 MHz, acetone-d$_6$) of **2** ....... 18

Figure S18. HMBC spectrum (700 MHz, acetone-d$_6$) of **2** ....... 19

Figure S19. ROESY spectrum (700 MHz, acetone-d$_6$) of **2** ....... 20

Figure S20. $^{1}$H NMR spectrum (700 MHz, acetone-d$_6$) of **3** ....... 21

Figure S21. $^{13}$C NMR spectrum (75.48 MHz, acetone-d$_6$) of **3** ....... 22

Figure S22. COSY-45 spectrum (700 MHz, acetone-d$_6$) of **3** ....... 23

Figure S23. HSQC spectrum (700 MHz, acetone-d$_6$) of **3** ....... 24

Figure S24. HMBC spectrum (700 MHz, acetone-d$_6$) of **3** ....... 25

Figure S25. ROESY spectrum (700 MHz, acetone-d$_6$) of **3** ....... 27

Figure S26. $^{1}$H spectrum (500 MHz, acetone-d$_6$) of **4** ....... 28

Figure S27. $^{13}$C spectrum (125.77 MHz, acetone-d$_6$) of **4** ....... 29

Figure S28. DEPT-135 NMR spectrum (125.77 MHz, acetone-d$_6$) of **4** ....... 30

Figure S29. COSY-45 spectrum (500 MHz, acetone-d$_6$) of **4** ....... 31

Figure S30. HSQC spectrum (500 MHz, acetone-d$_6$) of **4** ....... 32

Figure S31. HMBC spectrum (500 MHz, acetone-d$_6$) of **4** ....... 33

Figure S32. ROESY spectrum (500 MHz, acetone-d$_6$) of **4** ....... 34

Figure S33. $^{1}$H NMR spectrum (500 MHz, $CDCl_3$) of **5** ....... 35

Figure S34. $^{13}$C NMR spectrum (125.77 MHz, $CDCl_3$) of **5** ....... 36

Figure S35. DEPT-135 spectrum (125.77 MHz, $CDCl_3$) of **5** ....... 37

Figure S36. $^{1}$H NMR spectrum (700 MHz, acetone-d$_6$) of **6** ....... 38

Figure S37. $^{13}$C NMR spectrum (125.77 MHz, acetone-d$_6$) of **6** ....... 39

Figure S38. DEPT-135 spectrum (125.77 MHz, acetone-d$_6$) of **6** ....... 40

Figure S39. COSY-45 spectrum (700 MHz, acetone-$d_6$) of **6**......41
Figure S40. HSQC spectrum (700 MHz, acetone-$d_6$) of **6**......42
Figure S41. HMBC spectrum (700 MHz, acetone-$d_6$) of **6**......43
Figure S42. ROESY spectrum (500 MHz, acetone-$d_6$) of **6**......44
Figure S43. $^1$H NMR spectrum (700 MHz, acetone-$d_6$) of **7**......45
Figure S44. $^{13}$C NMR spectrum (176.04 MHz, acetone-$d_6$) of **7**......46
Figure S45. DEPT-135 spectrum (176.04 MHz, acetone-$d_6$) of **7**......47
Figure S46. COSY-45 spectrum (700 MHz, acetone-$d_6$) of **7**......48
Figure S47. HSQC spectrum (700 MHz, acetone-$d_6$) of **7**......49
Figure S48. HMBC spectrum (700 MHz, acetone-$d_6$) of **7**......50
Figure S49. ROESY spectrum (700 MHz, acetone-$d_6$) of **7**......51
Figure S50. $^1$H NMR spectrum (700 MHz, acetone-$d_6$) of **8**......52
Figure S51. $^{13}$C NMR spectrum (176.04 MHz, acetone-$d_6$) of **8**......53
Figure S52. DEPT-135 spectrum (176.04 MHz, acetone-$d_6$) of **8**......54
Figure S53. COSY-45 spectrum (700 MHz, acetone-$d_6$) of **8**......55
Figure S54. HSQC spectrum (700 MHz, acetone-$d_6$) of **8**......56
Figure S55. HMBC spectrum (700 MHz, acetone-$d_6$) of **8**......57
Figure S56. ROESY spectrum (700 MHz, acetone-$d_6$) of **8**......58
Figure S57. $^1$H NMR spectrum (500 MHz, acetone-$d_6$) of **9**......59
Figure S58. $^{13}$C NMR spectrum (125.77 MHz, acetone-$d_6$) of **9**......60
Figure S59. DEPT-135 spectrum (125.77 MHz, acetone-$d_6$) of **9**......61
Figure S60. COSY-45 spectrum (500 MHz, acetone-$d_6$) of **9**......62
Figure S61. HSQC spectrum (500 MHz, acetone-$d_6$) of **9**......63
Figure S62. HMBC spectrum (500 MHz, acetone-$d_6$) of **9**......64
Figure S63. ROESY spectrum (500 MHz, acetone-$d_6$) of **9**......65
Figure S64. $^1$H NMR spectrum (500 MHz, acetone-$d_6$) of **10**......66
Figure S65. $^{13}$C NMR spectrum (125.77 MHz, acetone-$d_6$) of **10**......67
Figure S66. DEPT-135 spectrum (125.77 MHz, acetone-$d_6$) of **10**......68
Figure S67. COSY-45 spectrum (500 MHz, acetone-$d_6$) of **10**......69
Figure S68. HSQC spectrum (500 MHz, acetone-$d_6$) of **10**......70
Figure S69. HMBC spectrum (500 MHz, acetone-$d_6$) of **10**......71
Figure S70. ROESY spectrum (500 MHz, acetone-$d_6$) of **10**......72
Figure S71. $^1$H NMR spectrum (700 MHz, acetone-$d_6$) of **11**......73
Figure S72. $^{13}$C NMR spectrum (176.04 MHz, acetone-$d_6$) of **11**......74
Figure S73. DEPT-135 NMR spectrum (176.04 MHz, acetone-$d_6$) of **11**......75
Figure S74. COSY-45 spectrum (700 MHz, acetone-$d_6$) of **11**......76
Figure S75. HSQC spectrum (700 MHz, acetone-$d_6$) of **11**......77
Figure S76. HMBC spectrum (700 MHz, acetone-$d_6$) of **11**......78
Figure S77. ROESY spectrum (700 MHz, acetone-$d_6$) of **11**......79

Figure S78. $^1$H NMR spectrum (500 MHz, acetone-$d_6$) of **12** ........ 80
Figure S79. $^{13}$C NMR spectrum (125.77 MHz, acetone-$d_6$) of **12** ........ 81
Figure S80. COSY-45 spectrum (500 MHz, acetone-$d_6$) of **12** ........ 82
Figure S81. HSQC spectrum (500 MHz, acetone-$d_6$) of **12** ........ 83
Figure S82. HMBC spectrum (700 MHz, acetone-$d_6$) of **12** ........ 84
Figure S83. ROESY spectrum (700 MHz, acetone-$d_6$) of **12** ........ 85
Figure S84. $^1$H NMR spectrum (700 MHz, $CDCl_3$) of **13** ........ 86
Figure S85. $^{13}$C NMR spectrum (176.04 MHz, $CDCl_3$) of **13** ........ 87
Figure S86. DEPT-135 NMR spectrum (176.04 MHz, $CDCl_3$) of **13** ........ 88
Figure S87. COSY-45 spectrum (700 MHz, $CDCl_3$) of **13** ........ 89
Figure S88. HSQC spectrum (700 MHz, $CDCl_3$) of **13** ........ 90
Figure S89. HMBC spectrum (700 MHz, $CDCl_3$) of **13** ........ 91
Figure S90. ROESY spectrum (700 MHz, $CDCl_3$) of **13** ........ 92
Figure S91. (+) ESI MS of **1** ........ 93
Figure S92. (-) ESI MS of **1** ........ 93
Figure S93. (+) ESI MS of **2** ........ 94
Figure S94. (-) ESI MS of **2** ........ 94
Figure S95. (+) ESI MS of **3** ........ 95
Figure S96. (-) ESI MS of **3** ........ 95
Figure S97. (+) ESI MS of **6** ........ 96
Figure S98. (+) ESI MS of **7** ........ 97
Figure S99. (+) ESI MS of **8** ........ 98
Figure S100. (-) ESI MS of **9** ........ 99
Figure S101. (-) ESI MS of **10** ........ 100
Figure S102. (-) ESI MS of **11** ........ 101
Figure S103. (+) ESI MS of **12** ........ 101
Figure S104. (+) ESI MS of **13** ........ 102

Figure S1. UV spectrum of **1**

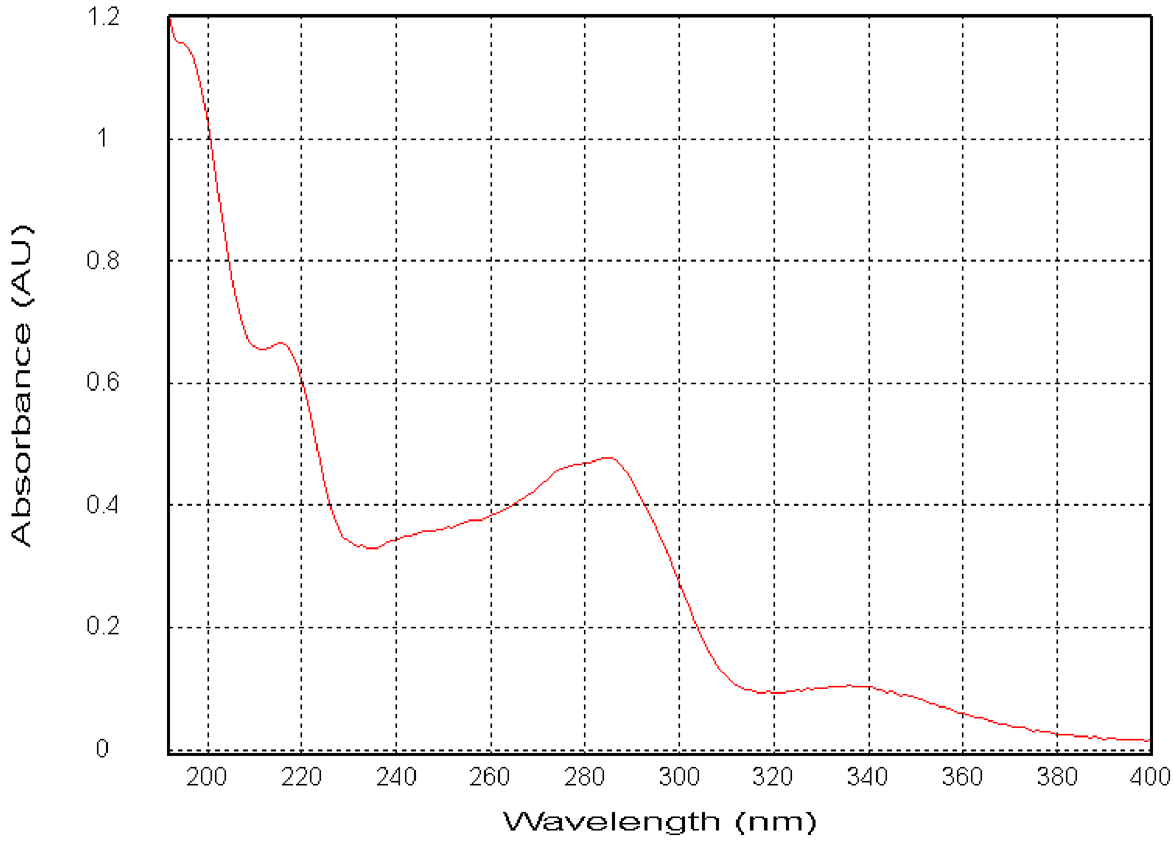


Figure S2. CD spectrum of **1**

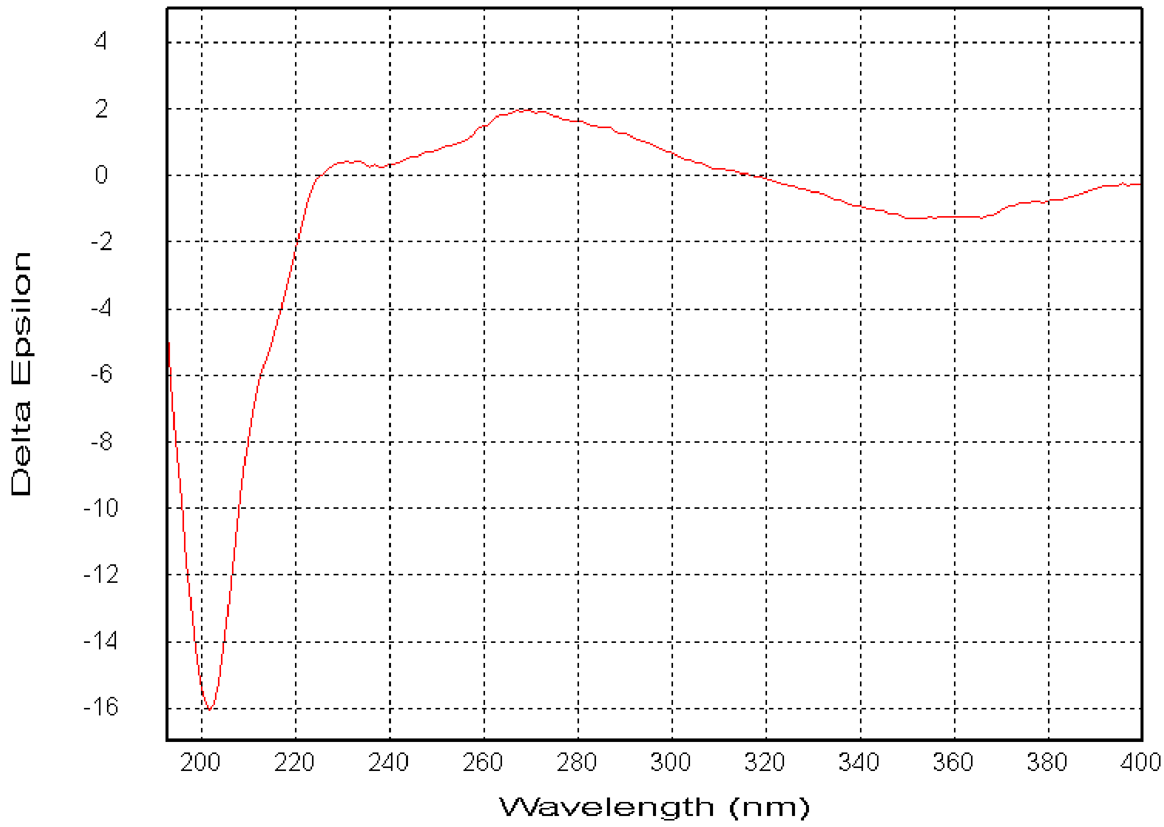

Figure S3. UV spectrum of **2**

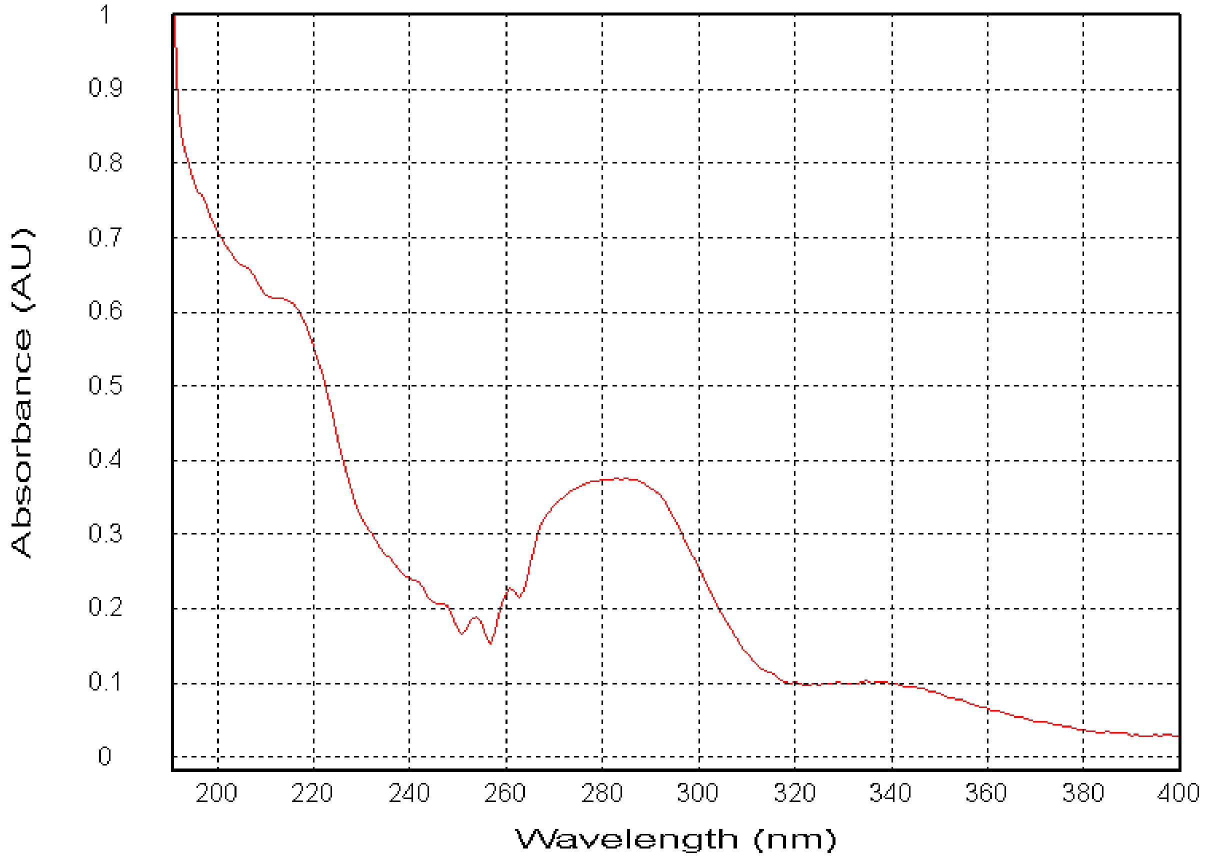


Figure S4. CD spectrum of **2**

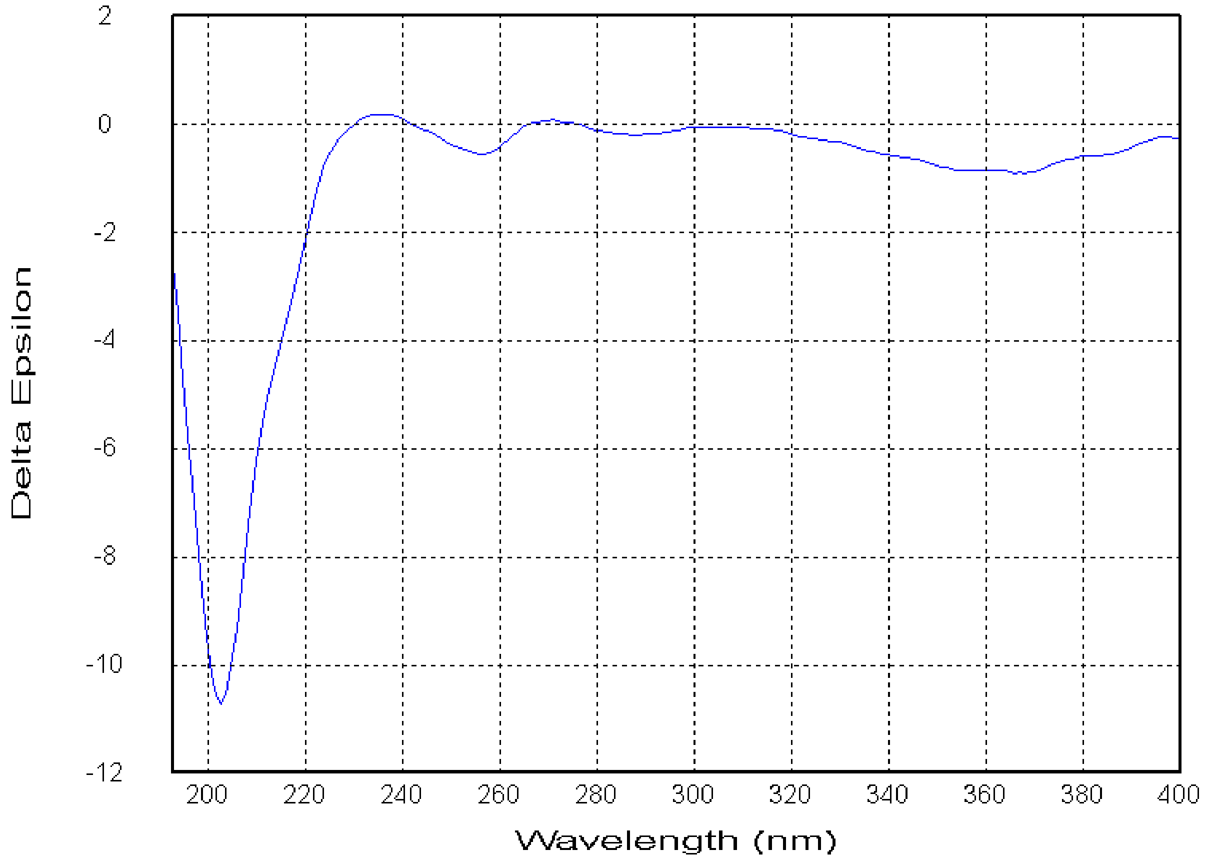

Figure S5. UV spectrum of **3**

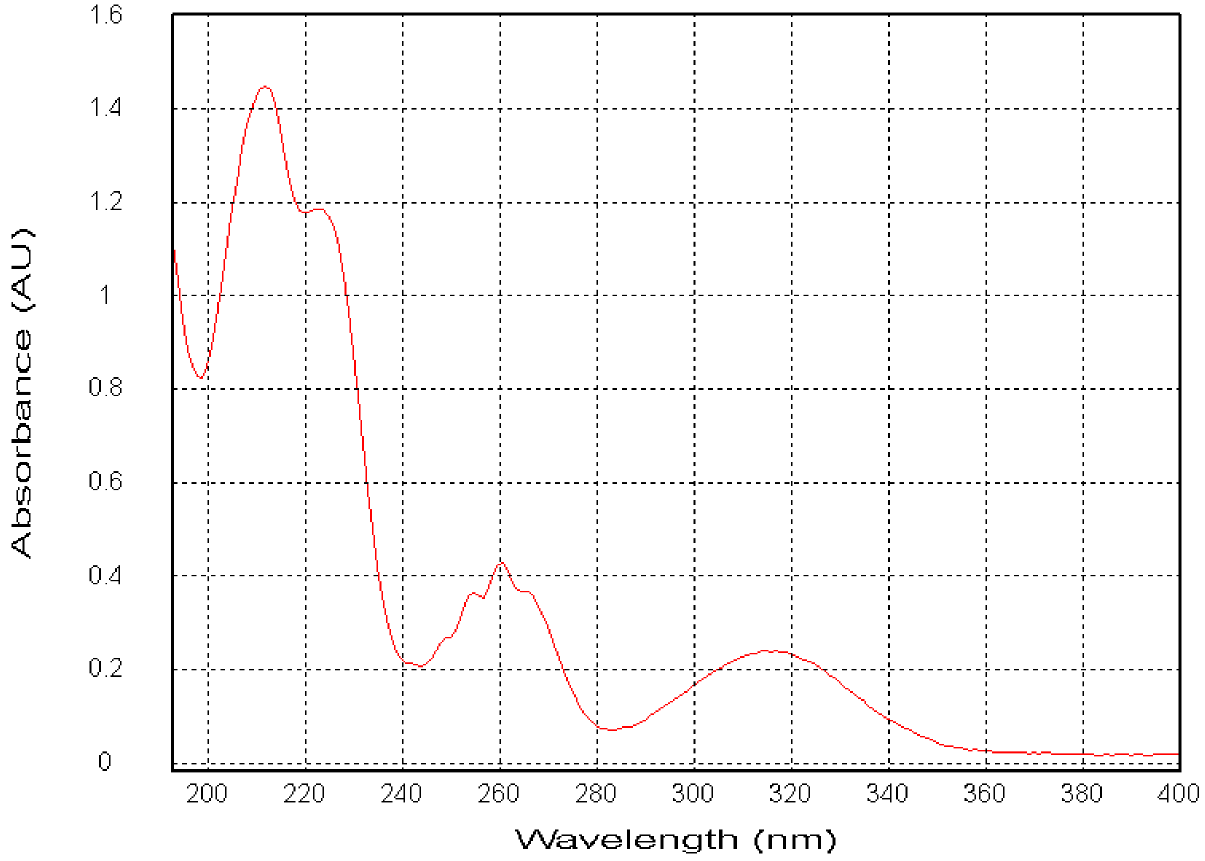


Figure S6. CD spectrum of **3**

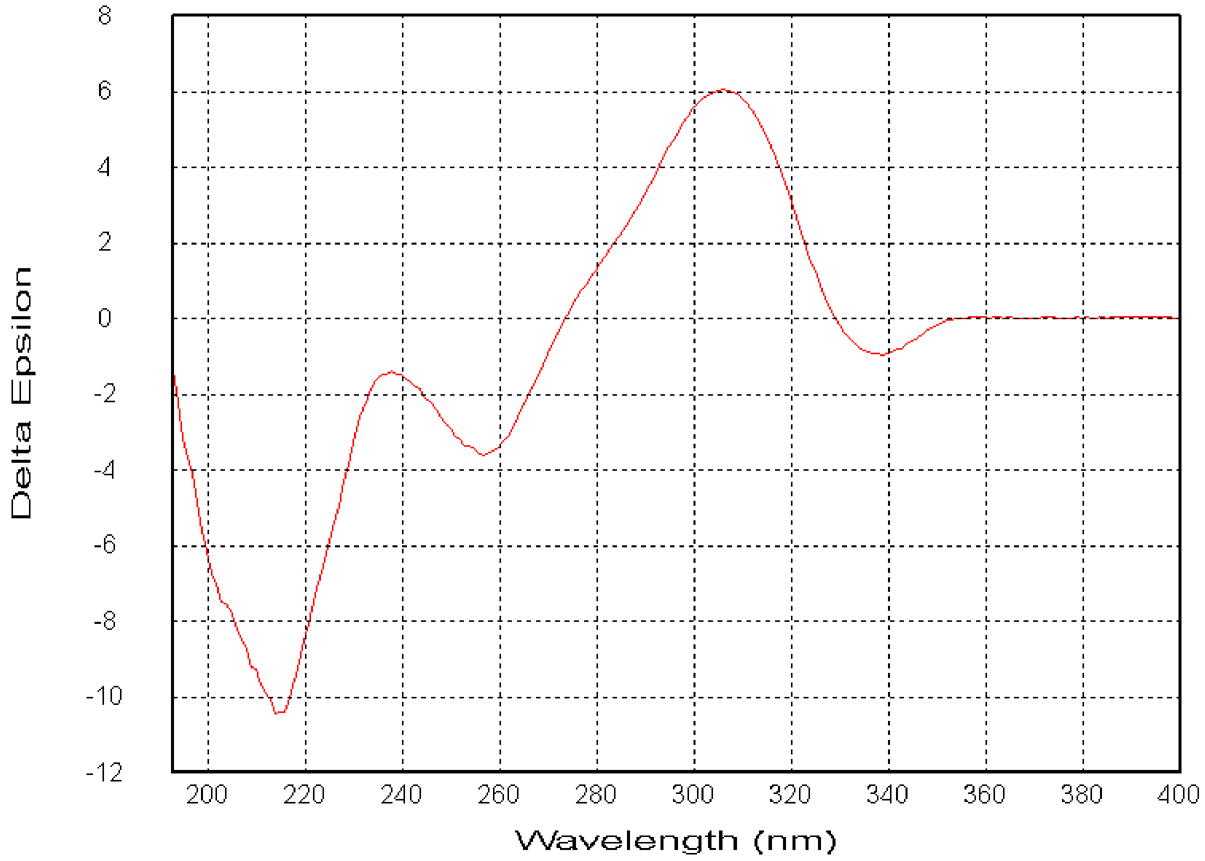

Figure S7. $^{1}$H NMR spectrum (500 MHz, acetone-$d_6$) of **1**

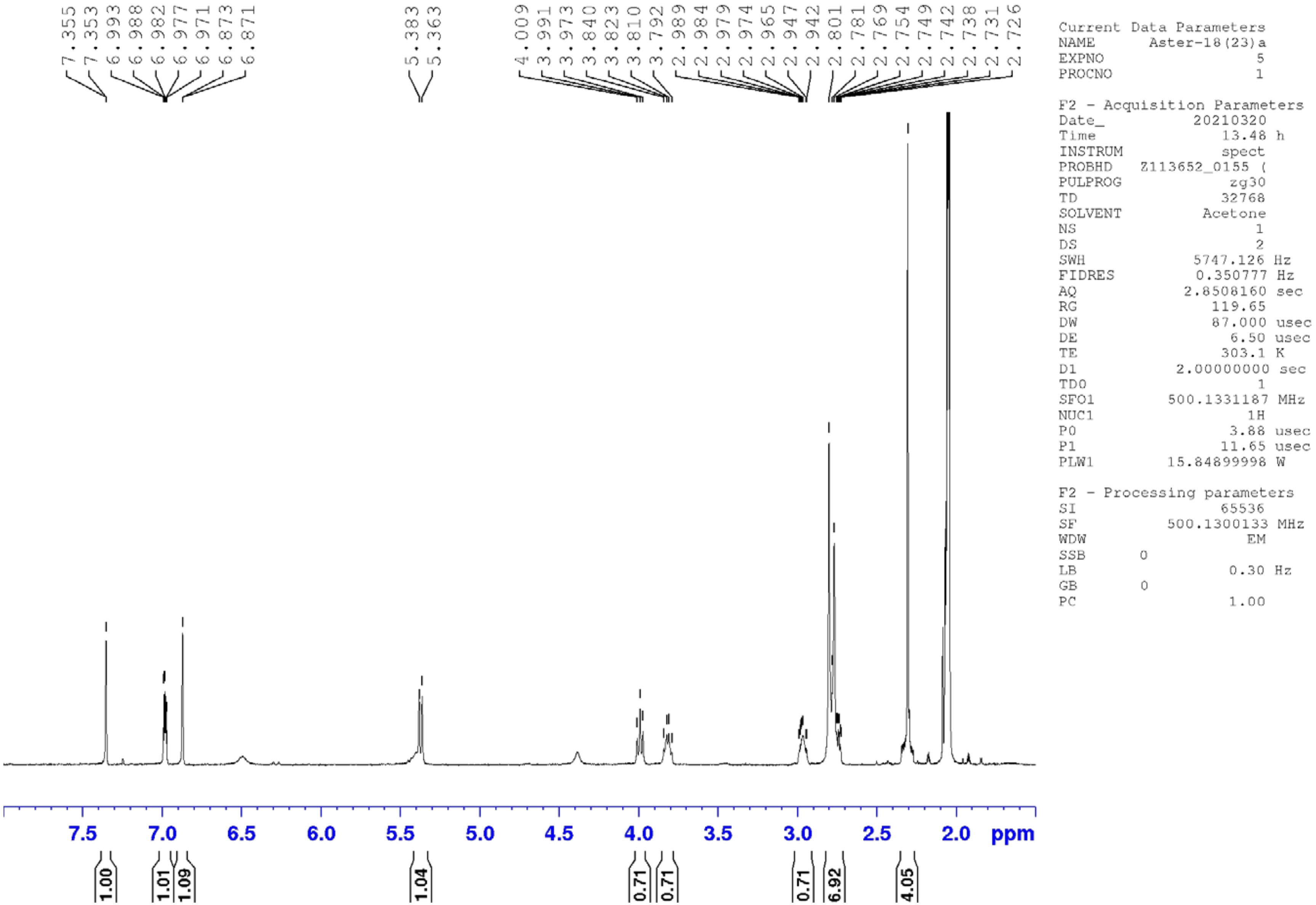

Figure S8. $^{13}$C NMR spectrum (125.75 MHz, acetone-$d_6$) of **1**

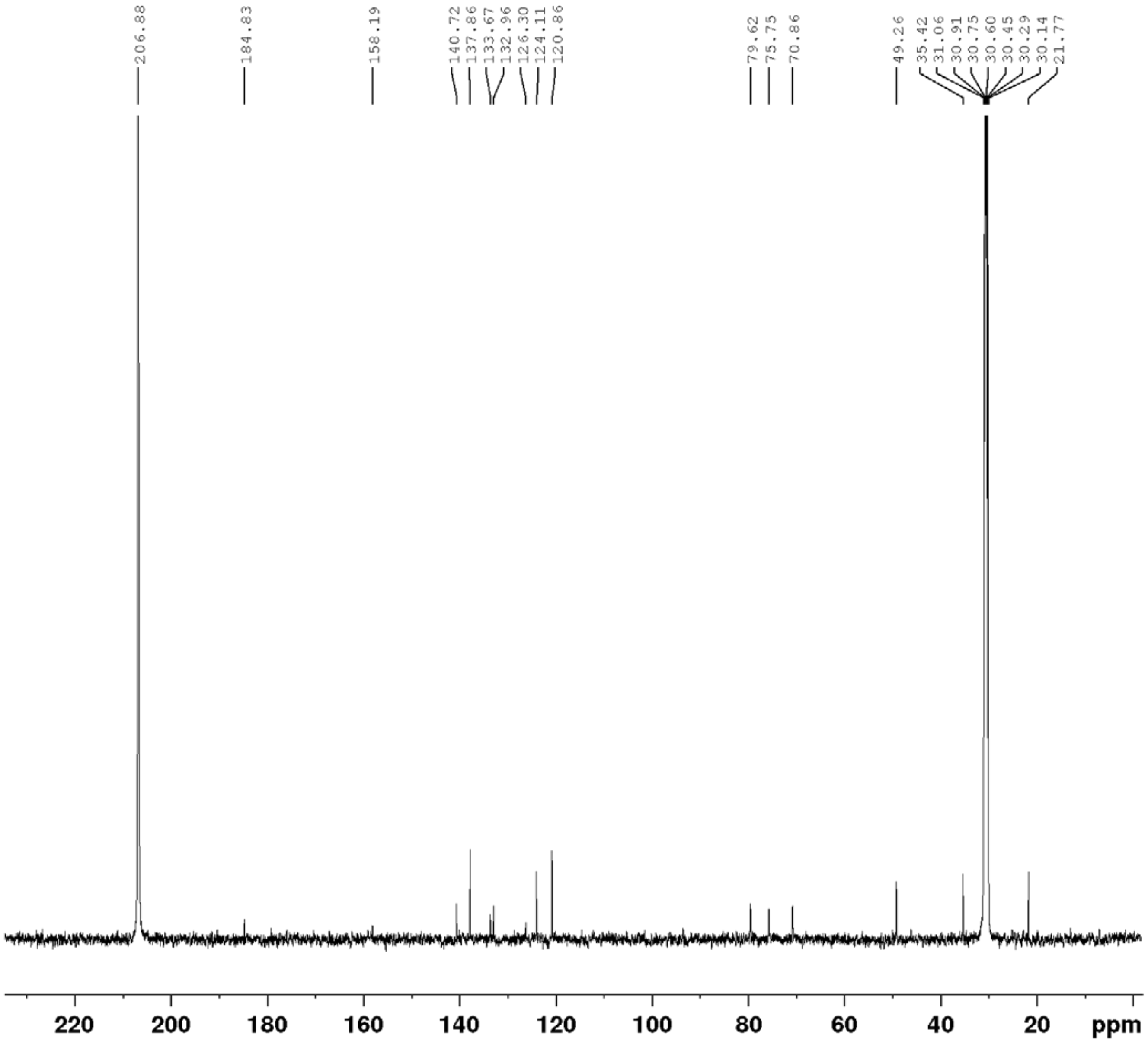


```
Current Data Parameters
NAME          Aster-18(23)a
EXPNO                    11
PROCNO                    1

F2 - Acquisition Parameters
Date_              20210320
Time                  13.46 h
INSTRUM               spect
PROBHD      Z113652_0155 (
PULPROG              zgpg30
TD                    32768
SOLVENT             Acetone
NS                     1291
DS                        4
SWH               29761.904 Hz
FIDRES             1.816522 Hz
AQ                0.5505024 sec
RG                   196.84
DW                   16.800 usec
DE                     6.50 usec
TE                    303.1 K
D1               1.50000000 sec
D11              0.03000000 sec
TD0                     128
SFO1            125.7722511 MHz
NUC1                    13C
P0                     4.03 usec
P1                    12.10 usec
PLW1            79.43299866 W
SFO2            500.1320005 MHz
NUC2                     1H
CPDPRG[2            waltz16
PCPD2                 80.00 usec
PLW2            15.84899998 W
PLW12            0.33610001 W
PLW13            0.16906001 W

F2 - Processing parameters
SI                    65536
SF              125.7575780 MHz
WDW                      EM
SSB      0
LB                     5.00 Hz
GB       0
PC                     1.30
```

Figure S9. COSY-45 spectrum (500 MHz, acetone-$d_6$) of **1**

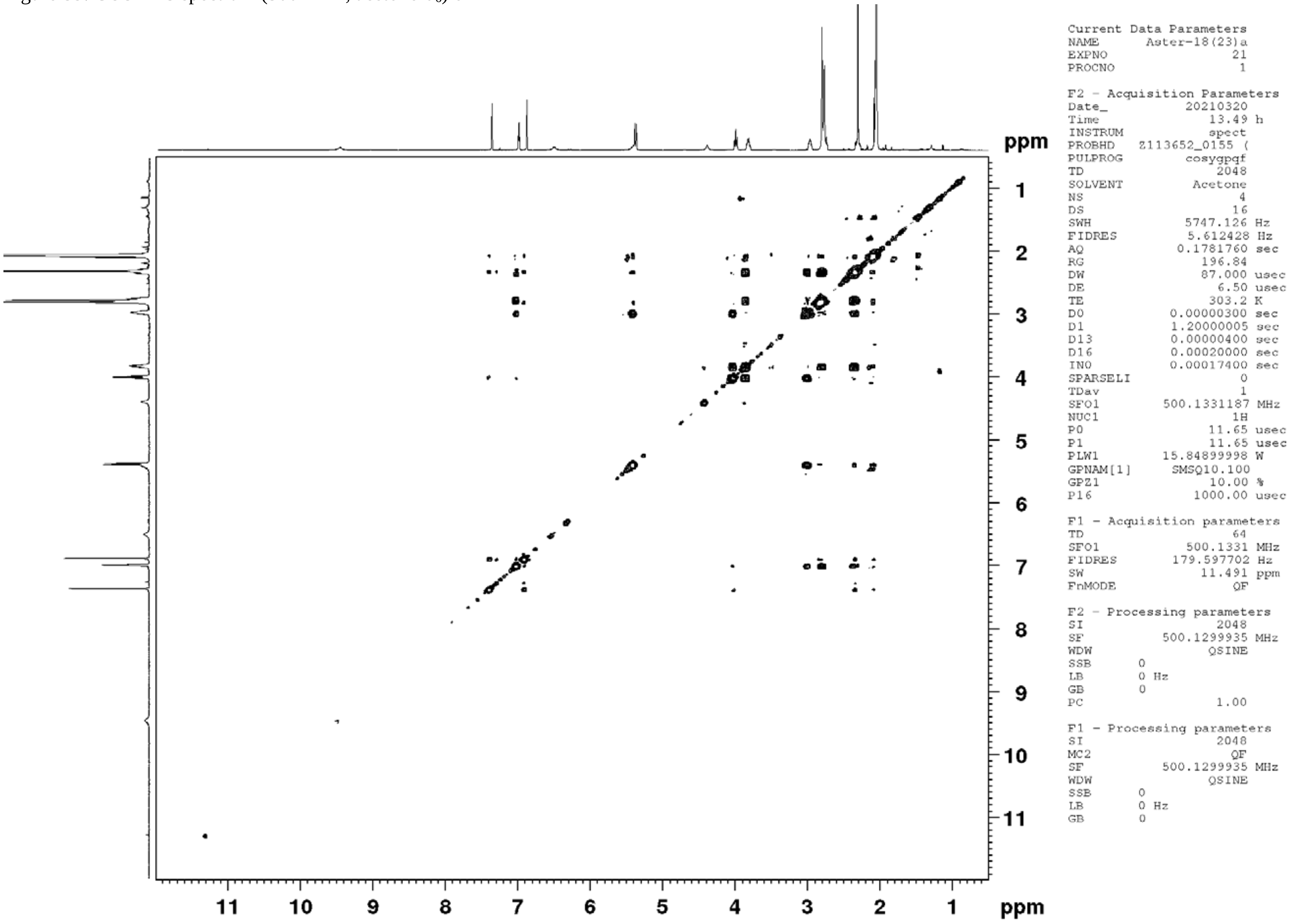

Figure S10. HSQC spectrum (500 MHz, acetone-$d_6$) of **1**

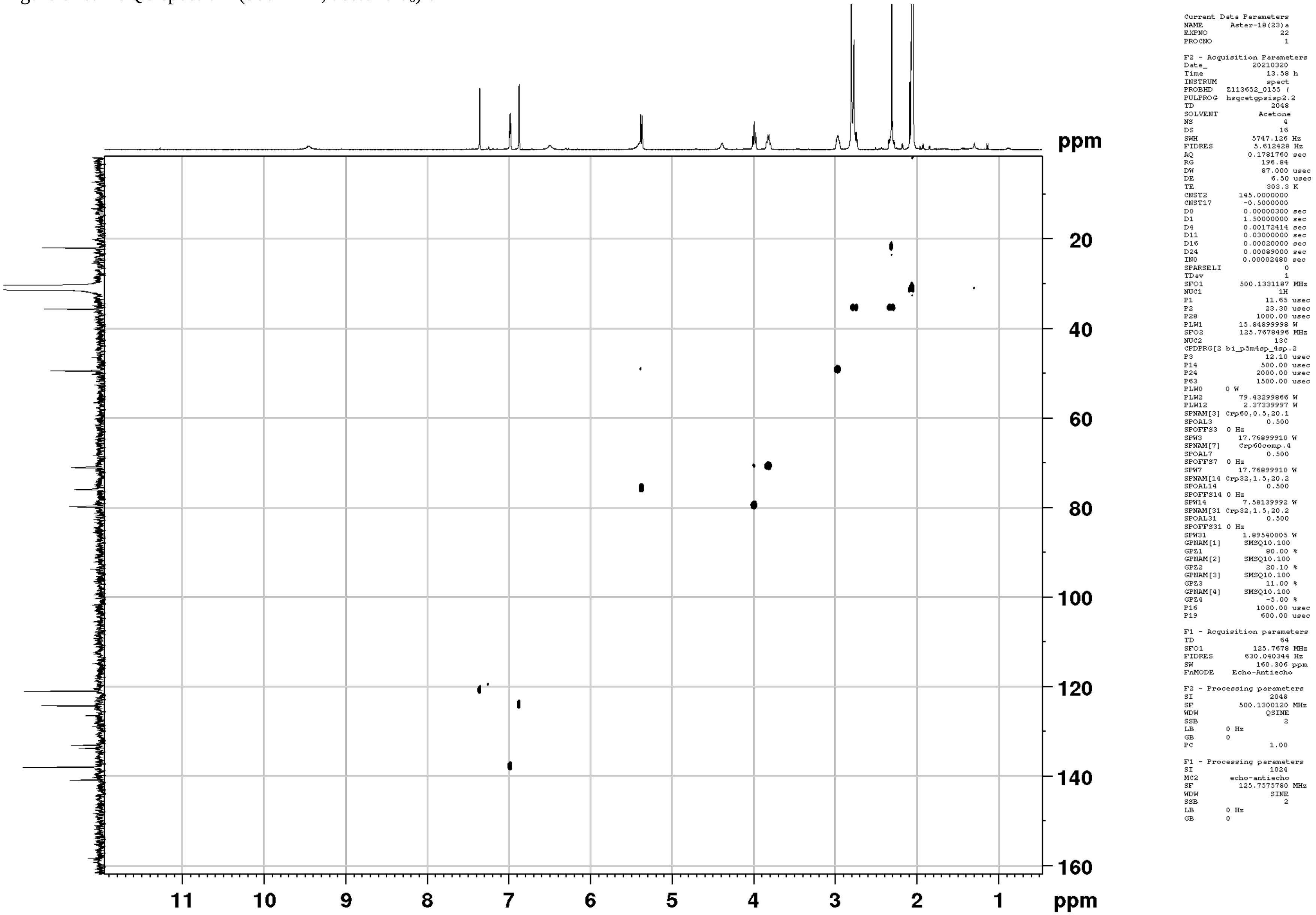

Figure S11. HMBC spectrum (500 MHz, acetone-$d_6$) of **1**

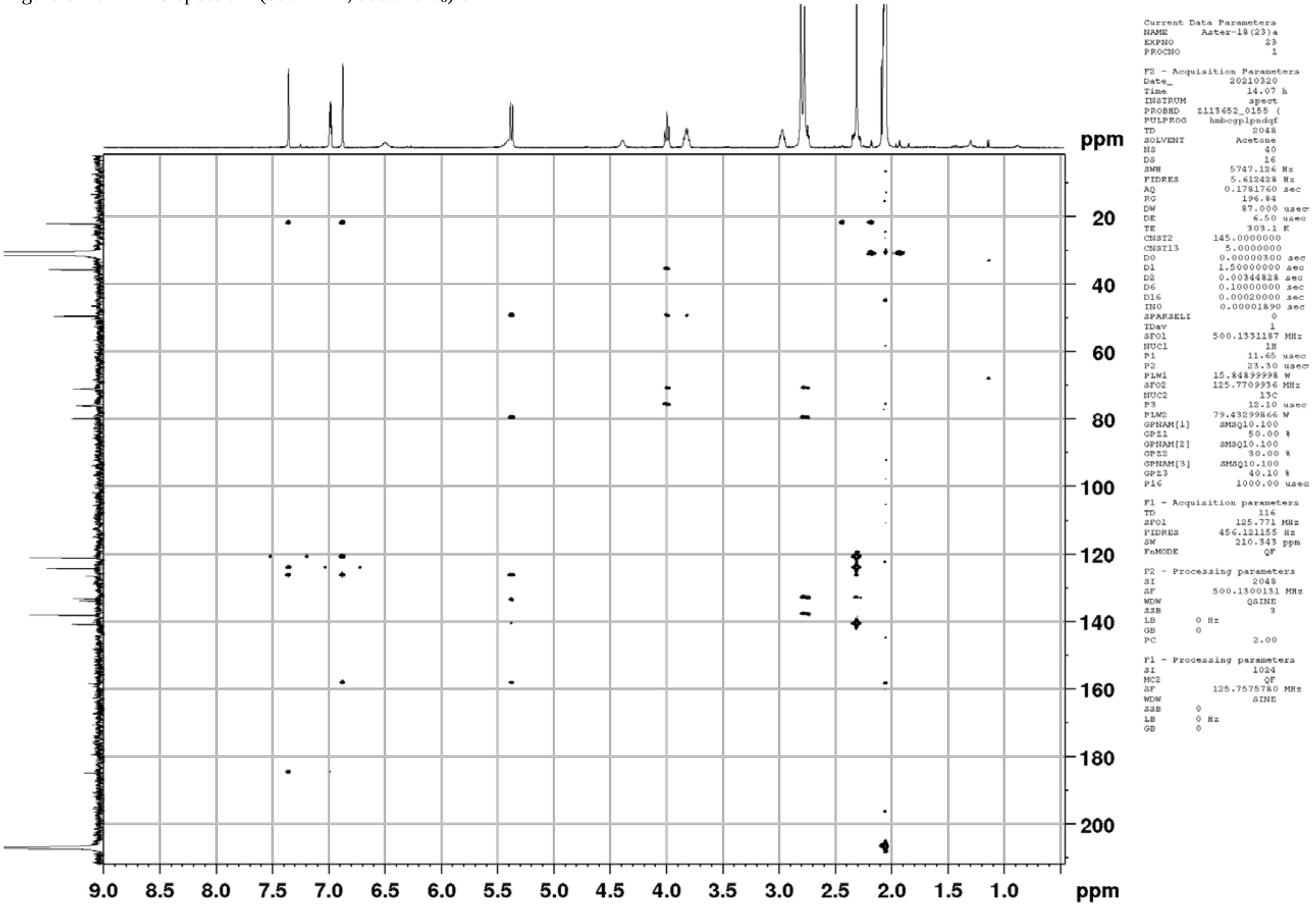

Figure S12. ROESY spectrum (700 MHz, acetone-$d_6$) of **1**

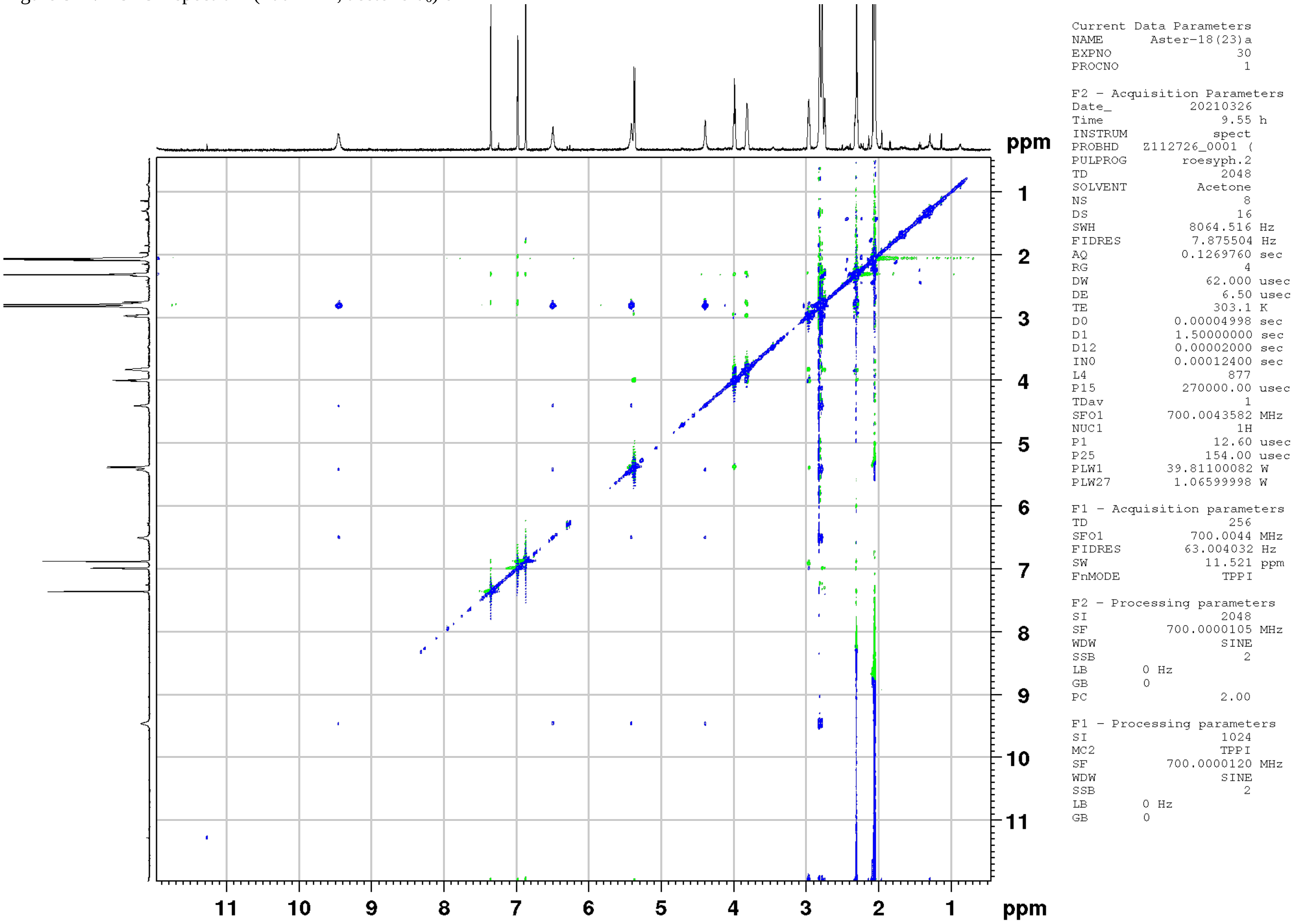

Figure S13. $^{1}$H NMR spectrum (500 MHz, acetone-$d_6$) of **2**

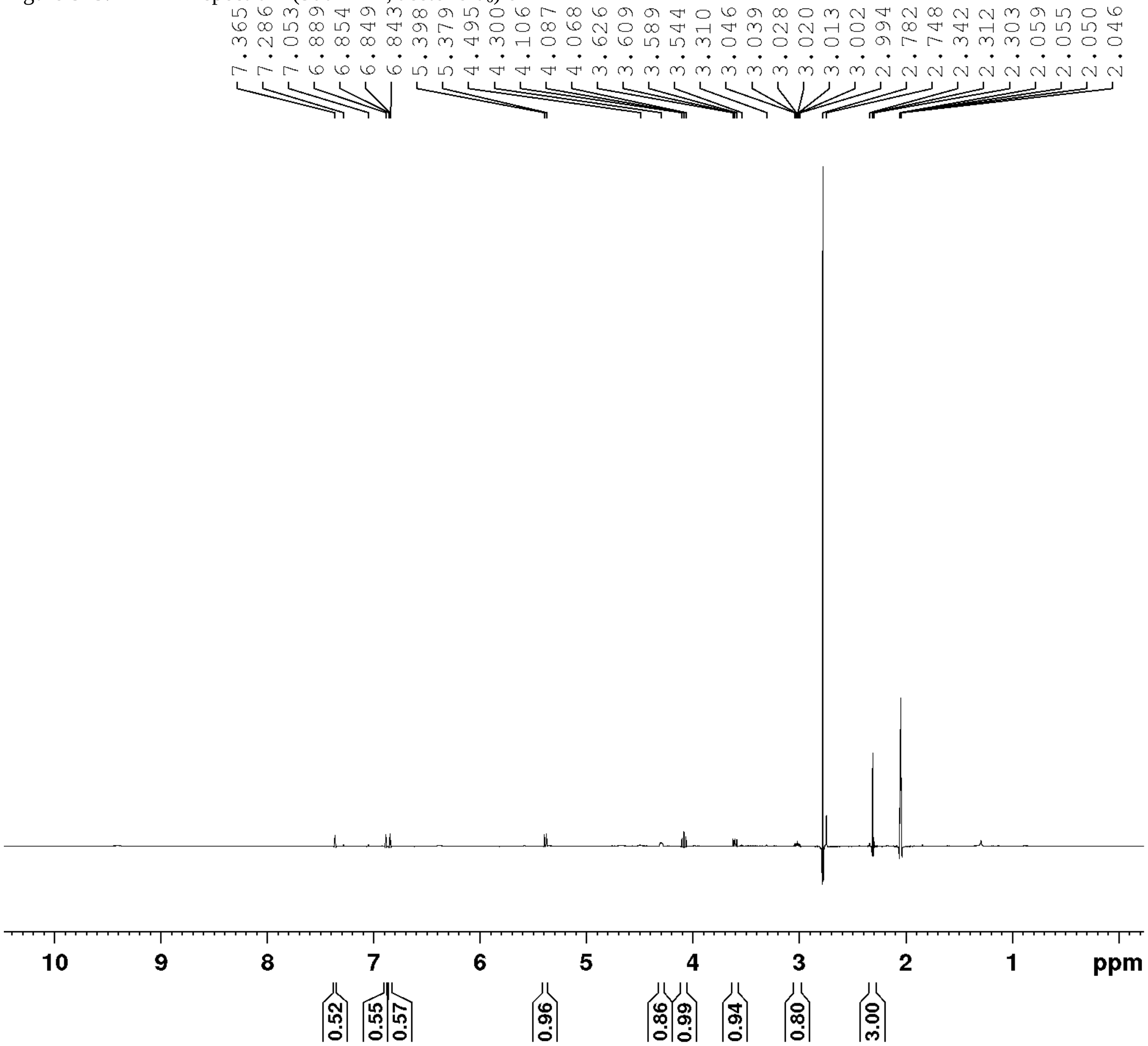


Current Data Parameters
NAME Aster-53-4
EXPNO 30
PROCNO 1

F2 - Acquisition Parameters
Date_ 20210913
Time 17.02 h
INSTRUM spect
PROBHD Z113652_0155 (
PULPROG zg
TD 32768
SOLVENT Acetone
NS 1
DS 2
SWH 8012.820 Hz
FIDRES 0.489064 Hz
AQ 2.0447233 sec
RG 91.04
DW 62.400 usec
DE 6.50 usec
TE 303.2 K
D1 1.00000000 sec
TD0 1
SFO1 500.1339010 MHz
NUC1 1H
P1 12.00 usec
PLW1 15.84899998 W

F2 - Processing parameters
SI 65536
SF 500.1300133 MHz
WDW GM
SSB 0
LB -2.50 Hz
GB 0.1
PC 3.00

Figure S14. $^{13}C$ NMR spectrum (125.75 MHz, acetone-$d_6$) of **2**

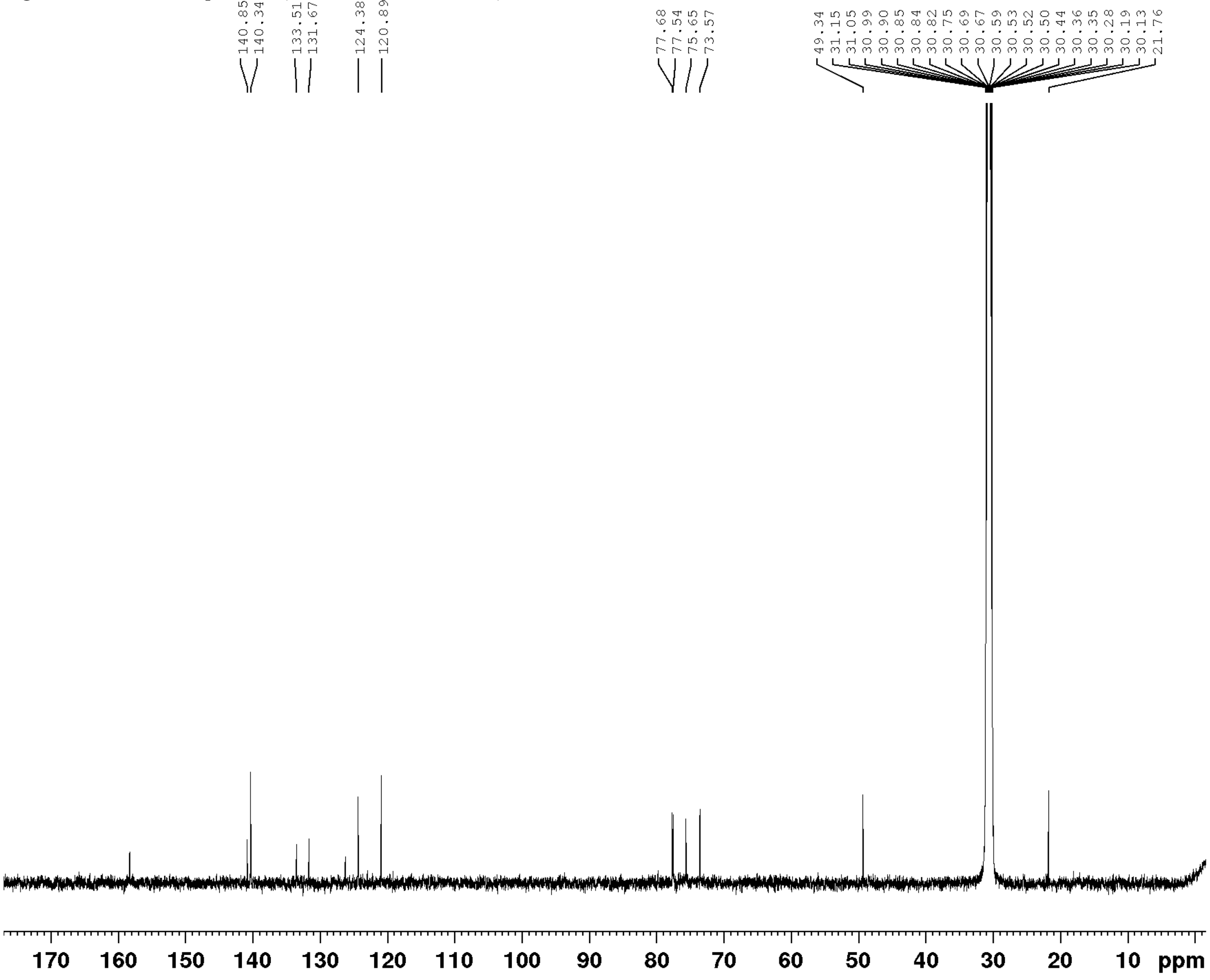


```
Current Data Parameters
NAME          Aster-53-4
EXPNO               3130
PROCNO                 1

F2 - Acquisition Parameters
Date_           20210914
Time               10.33 h
INSTRUM            spect
PROBHD   Z113652_0155 (
PULPROG           zgpg30
TD                 32768
SOLVENT          Acetone
NS                 24072
DS                     4
SWH            29761.904 Hz
FIDRES          1.816522 Hz
AQ             0.5505024 sec
RG                196.84
DW                16.800 usec
DE                  6.50 usec
TE                 303.1 K
D1            2.00000000 sec
D11           0.03000000 sec
TD0                 4096
SFO1         125.7722511 MHz
NUC1                 13C
P0                  3.96 usec
P1                 11.88 usec
PLW1         79.43299866 W
SFO2         500.1325007 MHz
NUC2                  1H
CPDPRG[2         waltz16
PCPD2              80.00 usec
PLW2         15.84899998 W
PLW12         0.33610001 W
PLW13         0.16906001 W

F2 - Processing parameters
SI                 65536
SF           125.7575780 MHz
WDW                   EM
SSB      0
LB                  2.00 Hz
GB       0
PC                  1.30
```

Figure S15. DEPT-135 spectrum (125.75 MHz, acetone-d$_6$) of **2**

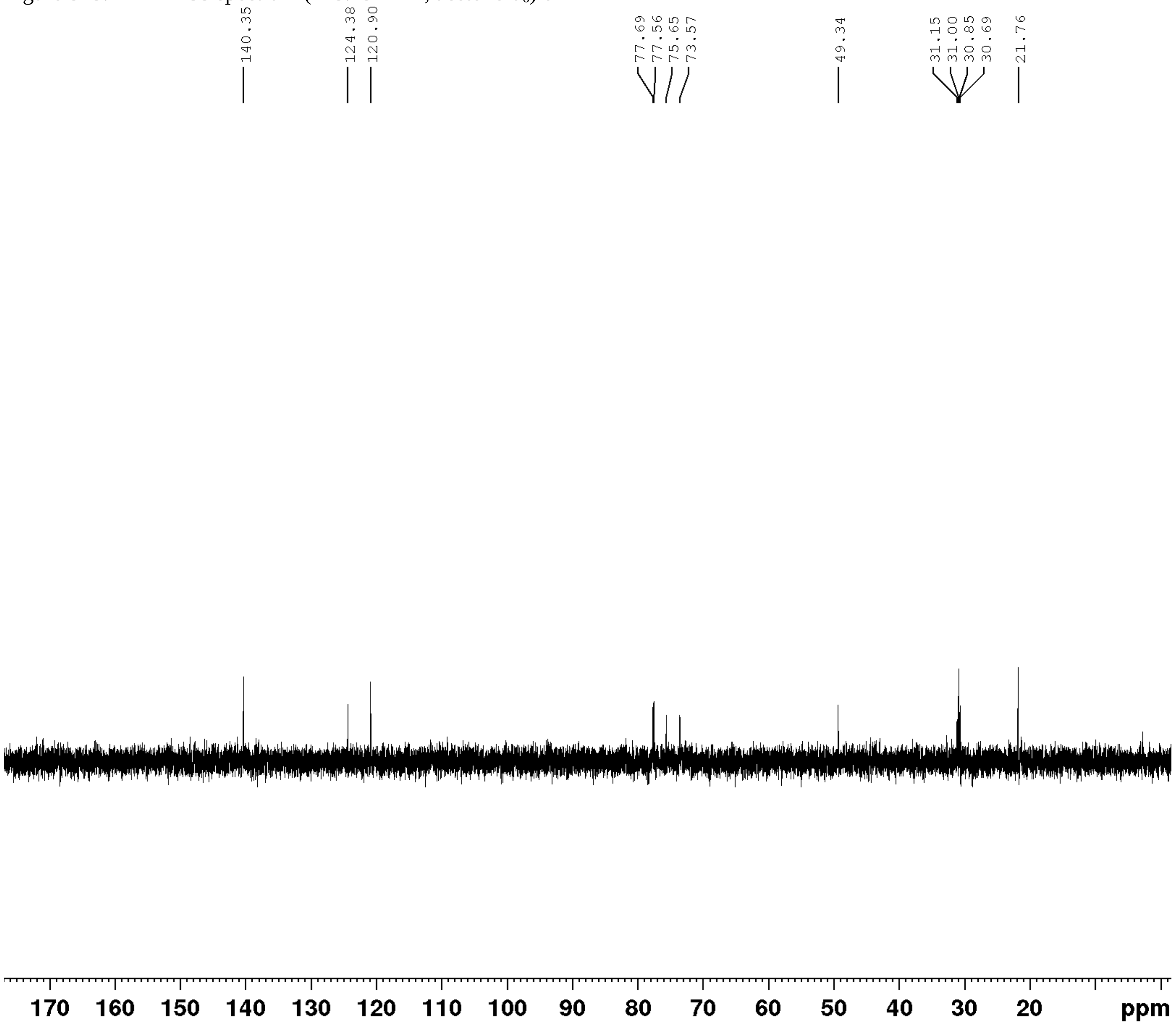


```
Current Data Parameters
NAME          Aster-53-4
EXPNO               3135
PROCNO                 1

F2 - Acquisition Parameters
Date_           20210914
Time               11.50 h
INSTRUM            spect
PROBHD   Z113652_0155 (
PULPROG        deptsp135
TD                 65536
SOLVENT          Acetone
NS                  1285
DS                     2
SWH            22727.273 Hz
FIDRES          0.693581 Hz
AQ             1.4417920 sec
RG                196.84
DW                22.000 usec
DE                  6.50 usec
TE                 303.1 K
CNST2        145.0000000
D1            2.00000000 sec
D2            0.00344828 sec
D12           0.00002000 sec
TD0                 4096
SFO1         125.7684784 MHz
NUC1                 13C
P1                 11.88 usec
P13              2000.00 usec
PLW0      0 W
PLW1         79.43299866 W
SPNAM[5]     Crp60comp.4
SPOAL5             0.500
SPOFFS5   0 Hz
SPW5         17.12899971 W
SFO2         500.1320005 MHz
NUC2                  1H
CPDPRG[2         waltz16
P3                 11.65 usec
P4                 23.30 usec
PCPD2              80.00 usec
PLW2         15.84899998 W
PLW12         0.33610001 W

F2 - Processing parameters
SI                 65536
SF           125.7575775 MHz
WDW                   EM
SSB      0
LB                  1.00 Hz
GB       0
PC                  1.30
```

Figure S16. COSY-45spectrum (700 MHz, acetone-d$_6$) of **2**

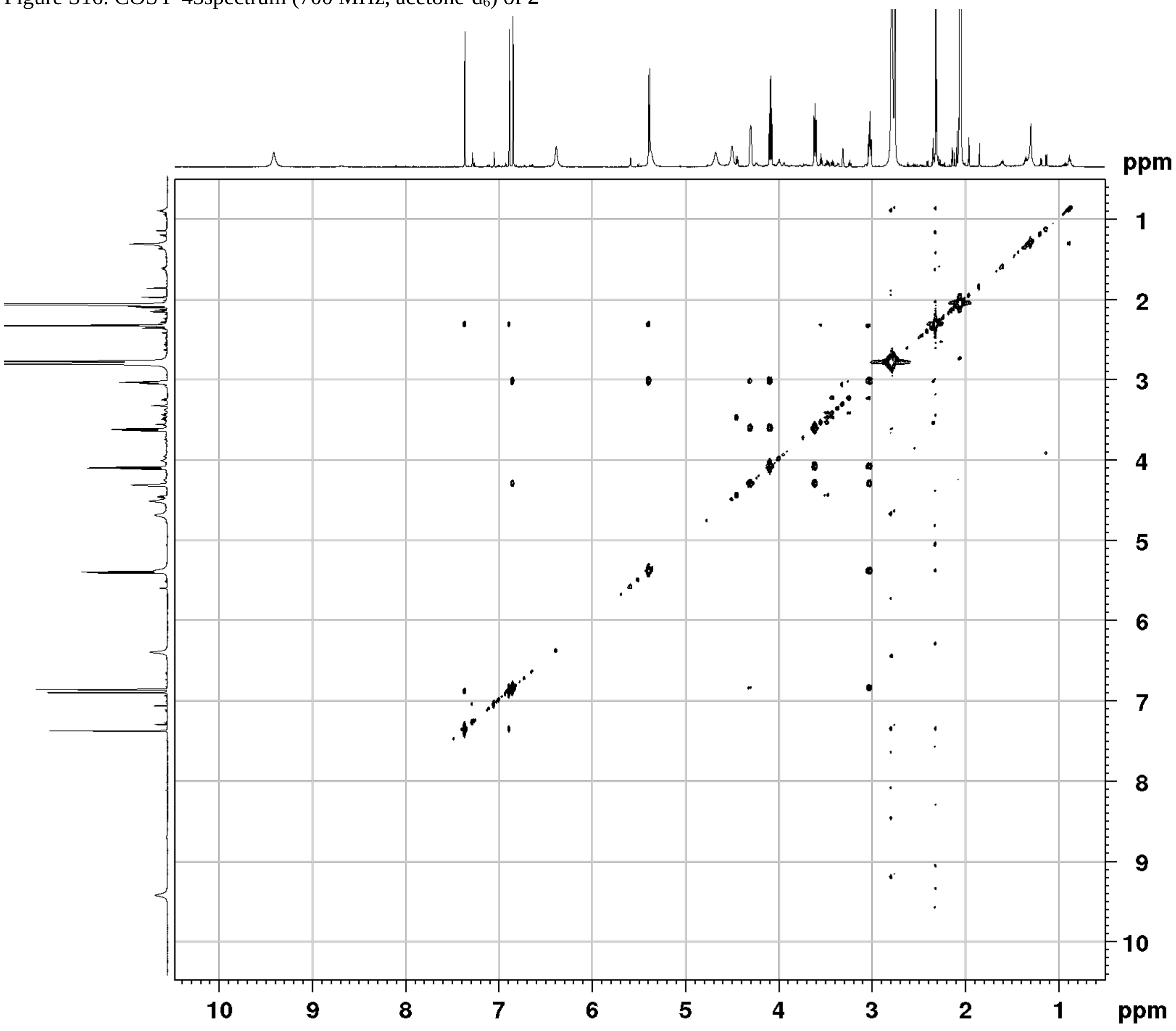


```
Current Data Parameters
NAME          Aster-53-4
EXPNO                455
PROCNO                 1

F2 - Acquisition Parameters
Date_           20210918
Time               14.30 h
INSTRUM            spect
PROBHD   Z180728_0001 (
PULPROG         cosygpqf
TD                  4096
SOLVENT          Acetone
NS                    16
DS                     8
SWH             7692.308 Hz
FIDRES          3.756010 Hz
AQ             0.2662400 sec
RG                191.01
DW                65.000 usec
DE                  6.50 usec
TE                 303.1 K
D0            0.00000300 sec
D1            2.00000000 sec
D13           0.00000400 sec
D16           0.00020000 sec
IN0           0.00013000 sec
SPARSELI               0
TDav                   1
SFO1         700.0035000 MHz
NUC1                  1H
P0                  8.00 usec
P1                  8.00 usec
PLW1         18.54100037 W
GPNAM[1]        SINE.100
GPZ1               10.00 %
P16              1000.00 usec

F1 - Acquisition parameters
TD                   128
SFO1           700.0035 MHz
FIDRES        120.192307 Hz
SW                10.989 ppm
FnMODE                QF

F2 - Processing parameters
SI                  2048
SF           700.0000133 MHz
WDW                 SINE
SSB      0
LB       0 Hz
GB       0
PC                  1.00

F1 - Processing parameters
SI                  2048
MC2                   QF
SF           700.0000123 MHz
WDW                 SINE
SSB      0
LB       0 Hz
GB       0
```

Figure S17. HSQC spectrum (700 MHz, acetone-$d_6$) of **2**

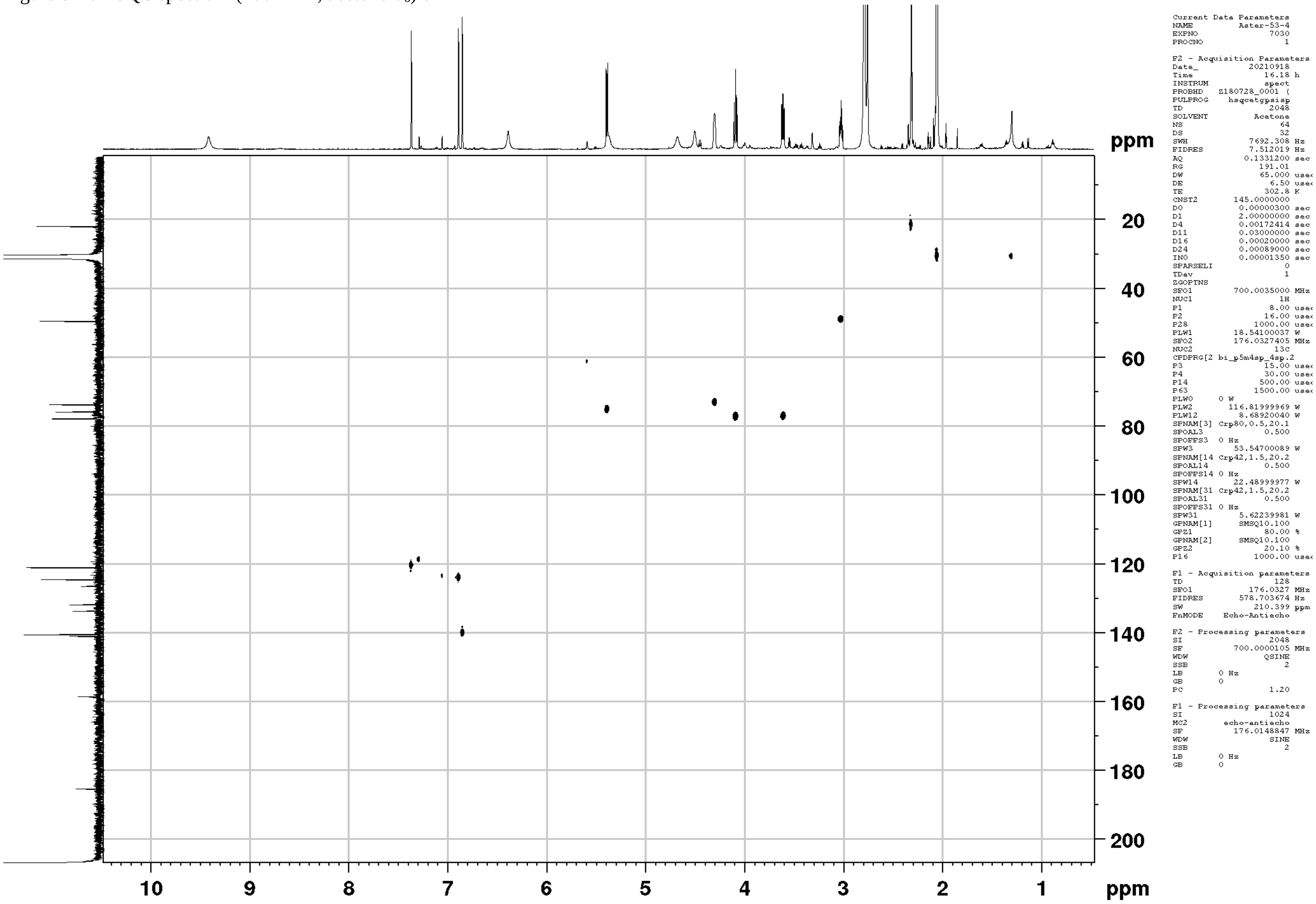

Figure S18. HMBC spectrum (700 MHz, acetone-$d_6$) of **2**

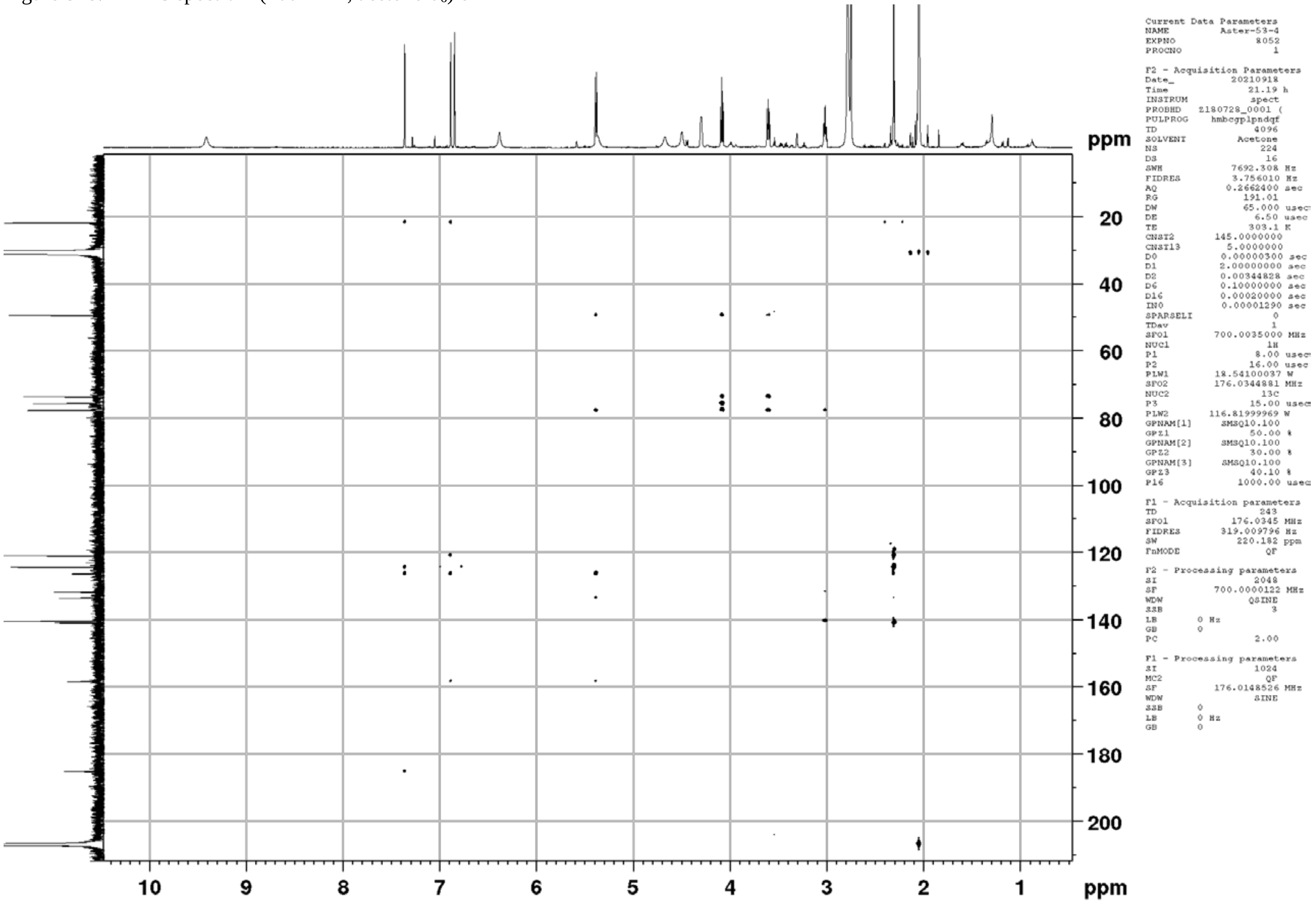

Figure S19. ROESY spectrum (700 MHz, acetone-d$_6$) of **2**

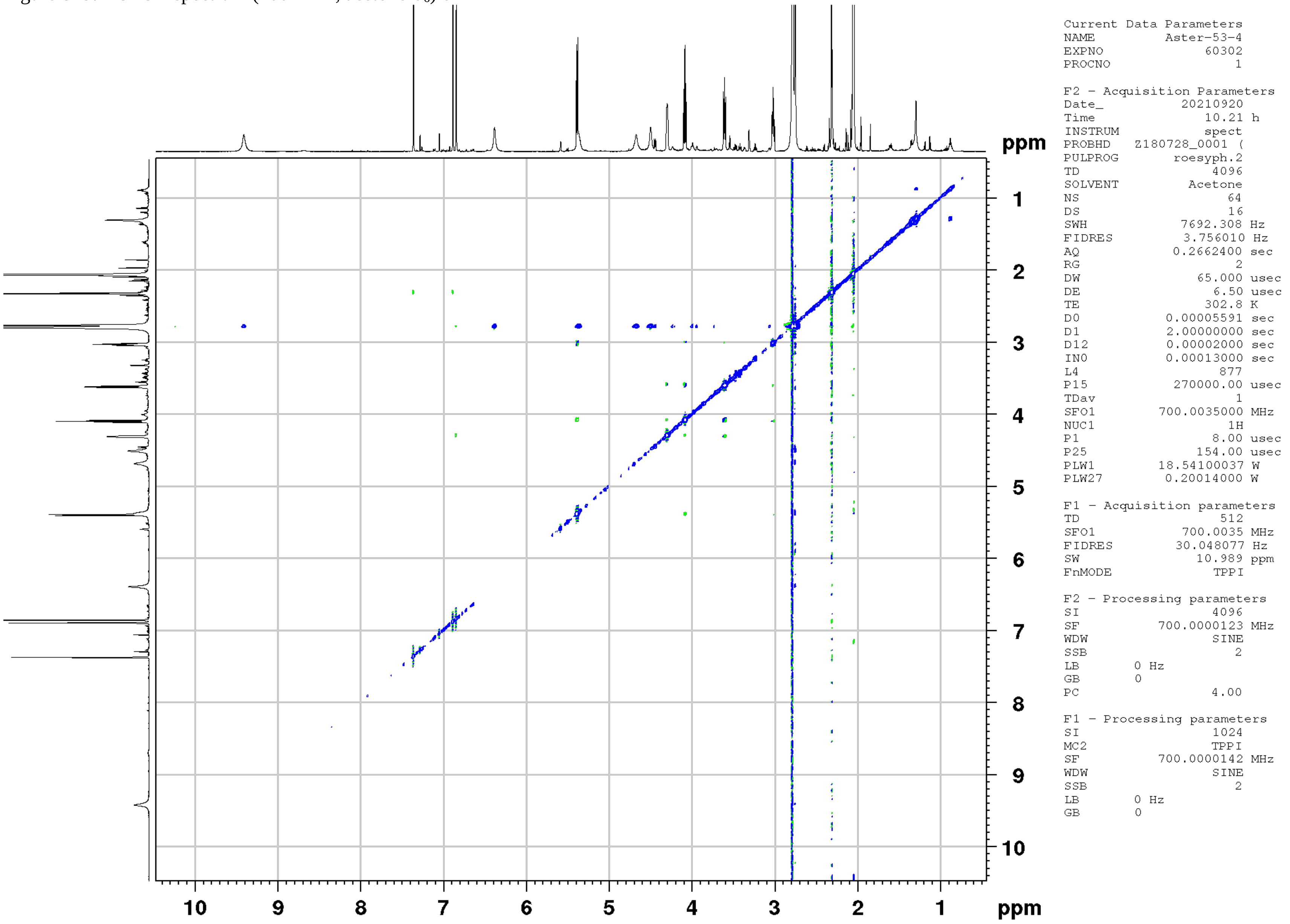

Figure S20. $^{1}H$ NMR spectrum (700 MHz, acetone-$d_6$) of **3**

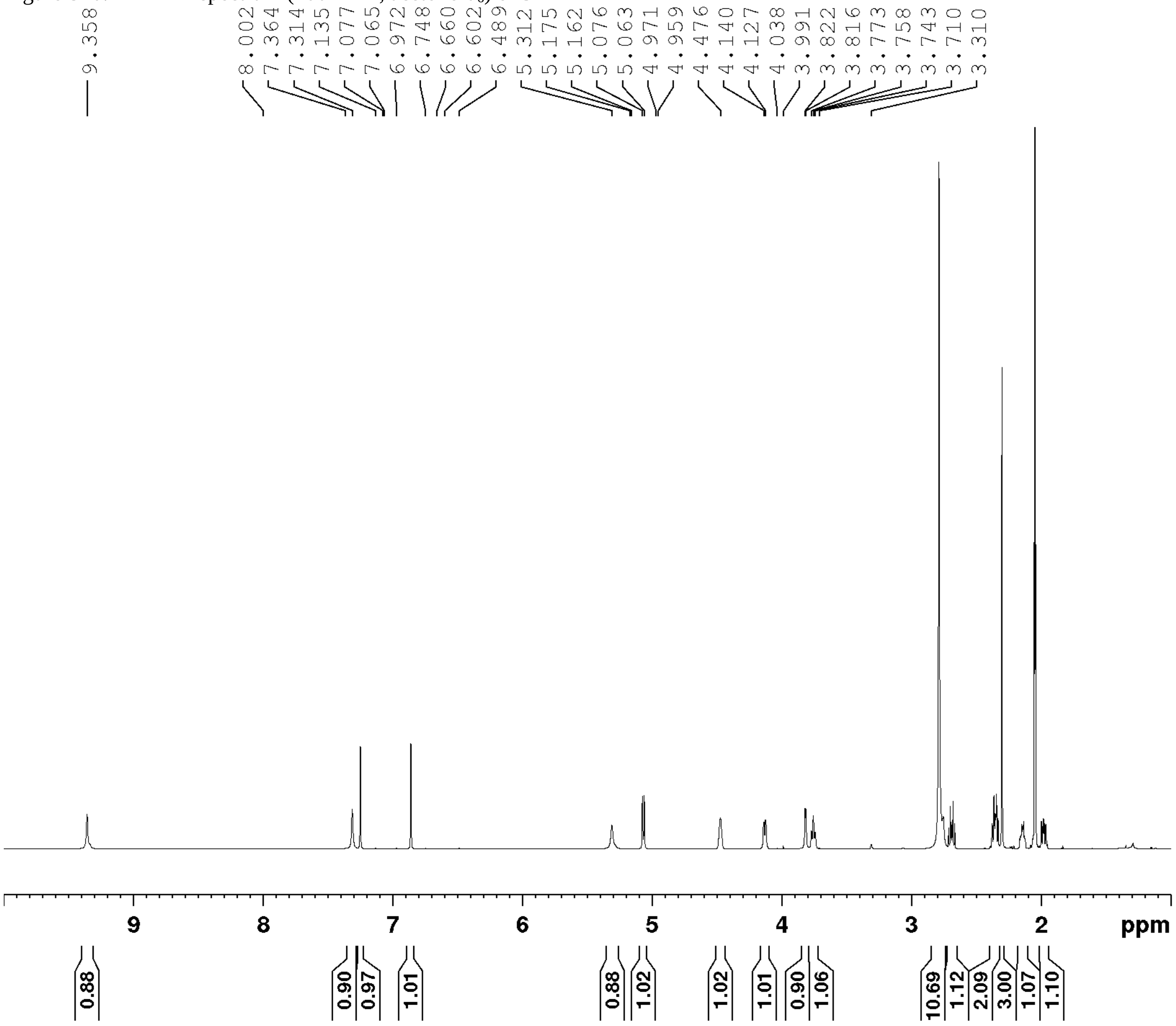


Current Data Parameters
NAME Aster-45a
EXPNO 32
PROCNO 1

F2 - Acquisition Parameters
Date_ 20210924
Time 12.15 h
INSTRUM spect
PROBHD Z180728_0001 (
PULPROG zg30
TD 32768
SOLVENT Acetone
NS 120
DS 4
SWH 8417.509 Hz
FIDRES 0.513764 Hz
AQ 1.9464192 sec
RG 165.68
DW 59.400 usec
DE 6.50 usec
TE 302.9 K
D1 2.00000000 sec
TD0 1
SFO1 700.0035000 MHz
NUC1 1H
P0 2.67 usec
P1 8.00 usec
PLW1 18.54100037 W

F2 - Processing parameters
SI 32768
SF 700.0000119 MHz
WDW EM
SSB 0
LB 0.30 Hz
GB 0
PC 4.00

Figure S21. $^{13}C$ NMR spectrum (75.48 MHz, acetone-$d_6$) of **3**

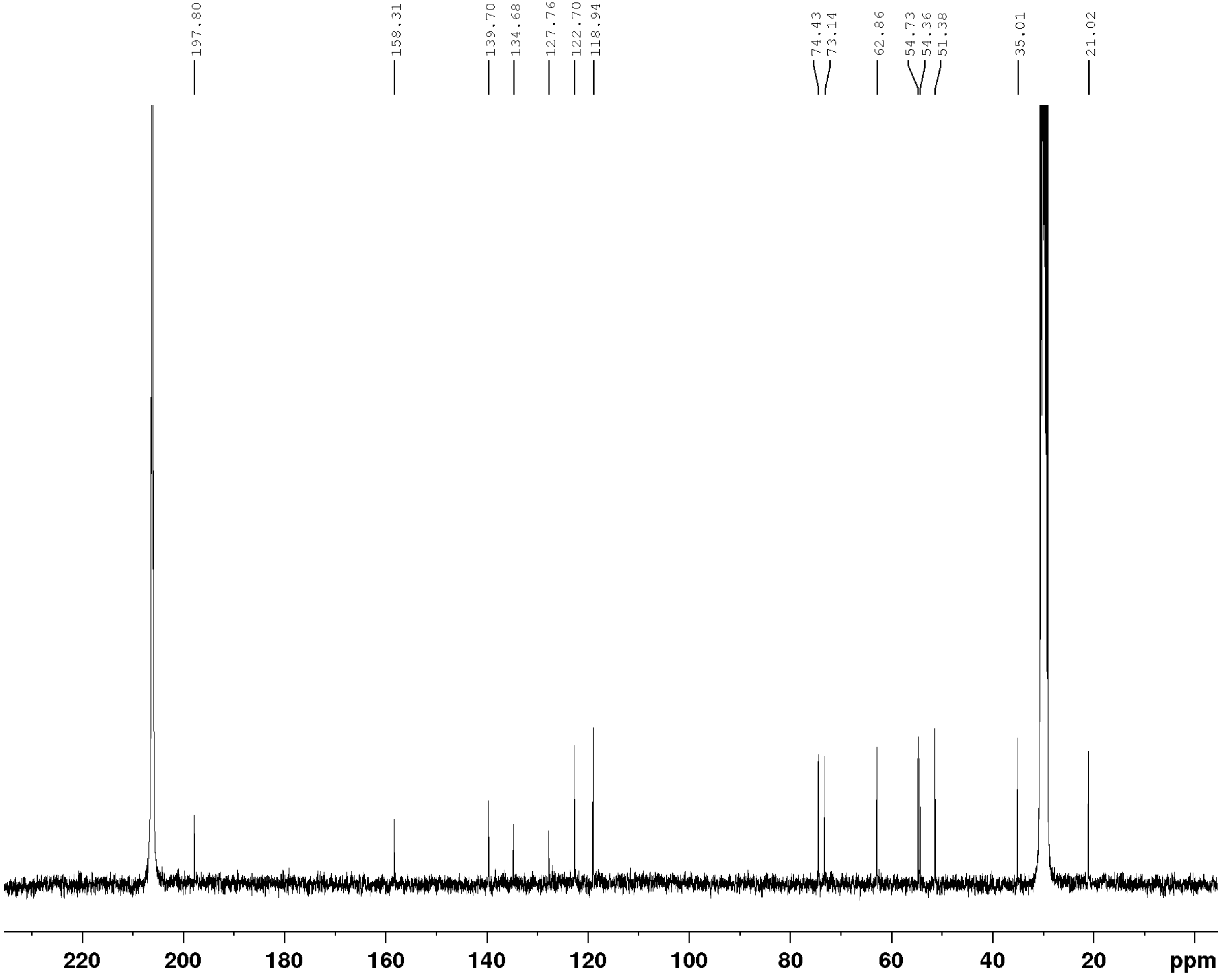


```
Current Data Parameters
NAME          Aster-45a
EXPNO              3330
PROCNO                1

F2 - Acquisition Parameters
Date_          20210927
Time              17.17
INSTRUM           spect
PROBHD   5 mm Dual 13C/
PULPROG         zgpg30
TD                32768
SOLVENT           CDCl3
NS                20224
DS                    2
SWH          18115.941 Hz
FIDRES        0.552855 Hz
AQ           0.9043968 sec
RG              11585.2
DW               27.600 usec
DE                 6.00 usec
TE                303.2 K
D1           2.00000000 sec
d11          0.03000000 sec
DELTA        1.89999998 sec
TD0                1536

======== CHANNEL f1 ========
NUC1                13C
P1                 5.80 usec
PL1               -4.00 dB
SFO1         75.4763978 MHz

======== CHANNEL f2 ========
CPDPRG[2        waltz16
NUC2                 1H
PCPD2             90.00 usec
PL2               -4.00 dB
PL12              20.00 dB
PL13              22.00 dB
SFO2        300.1312005 MHz

F2 - Processing parameters
SI                65536
SF           75.4676790 MHz
WDW                  EM
SSB      0
LB                 2.00 Hz
GB       0
PC                 1.30
```

Figure S22. COSY-45 spectrum (700 MHz, acetone-$d_6$) of **3**

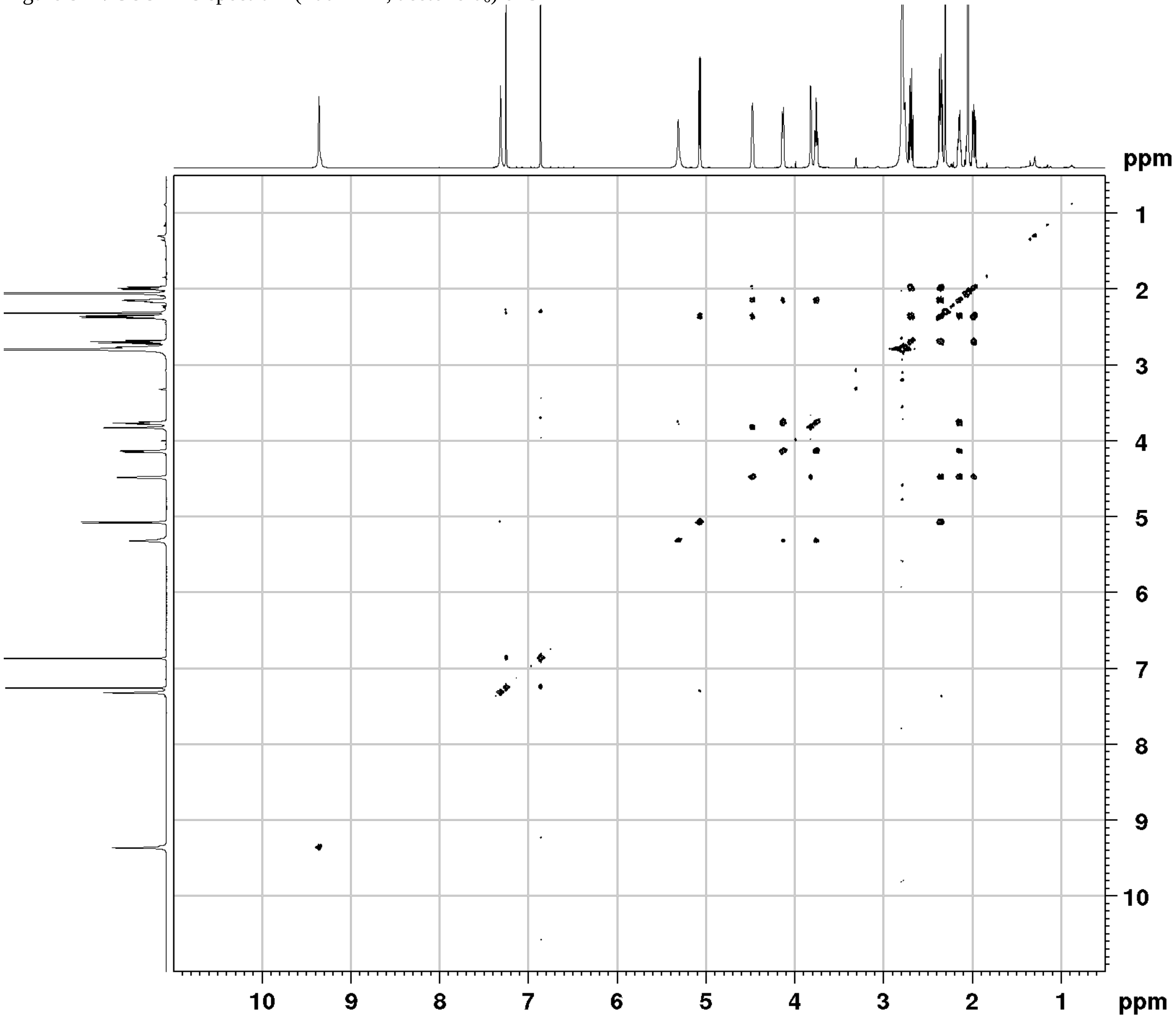


```
Current Data Parameters
NAME          Aster-45a
EXPNO               455
PROCNO                1

F2 - Acquisition Parameters
Date_          20210924
Time              12.32 h
INSTRUM           spect
PROBHD   Z180728_0001 (
PULPROG        cosygpqf
TD                 4096
SOLVENT         Acetone
NS                   16
DS                    8
SWH            8417.509 Hz
FIDRES         4.110112 Hz
AQ            0.2433024 sec
RG               191.01
DW               59.400 usec
DE                 6.50 usec
TE                302.7 K
D0           0.00000300 sec
D1           2.00000000 sec
D13          0.00000400 sec
D16          0.00020000 sec
IN0          0.00011880 sec
SPARSELI              0
TDav                  1
SFO1        700.0035000 MHz
NUC1                 1H
P0                 4.00 usec
P1                 8.00 usec
PLW1        18.54100037 W
GPNAM[1]       SINE.100
GPZ1              10.00 %
P16             1000.00 usec

F1 - Acquisition parameters
TD                  128
SFO1           700.0035 MHz
FIDRES       131.523575 Hz
SW               12.025 ppm
FnMODE               QF

F2 - Processing parameters
SI                 2048
SF          700.0000133 MHz
WDW                SINE
SSB      0
LB       0 Hz
GB       0
PC                 1.00

F1 - Processing parameters
SI                 2048
MC2                  QF
SF          700.0000123 MHz
WDW                SINE
SSB      0
LB       0 Hz
GB       0
```

Figure S23. HSQC spectrum (700 MHz, acetone-$d_6$) of **3**

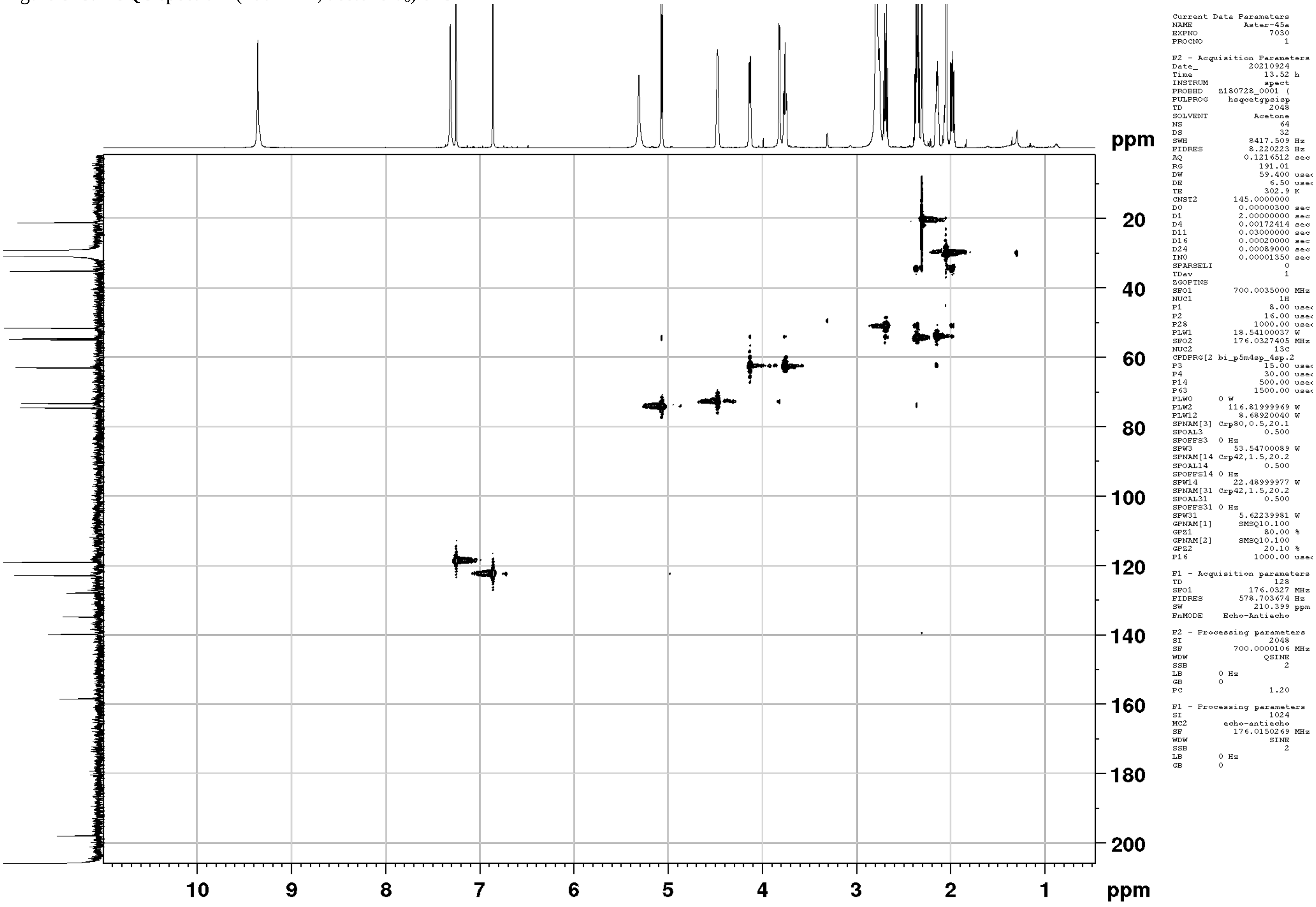

Figure S24. HMBC spectrum (700 MHz, acetone-$d_6$) of **3**

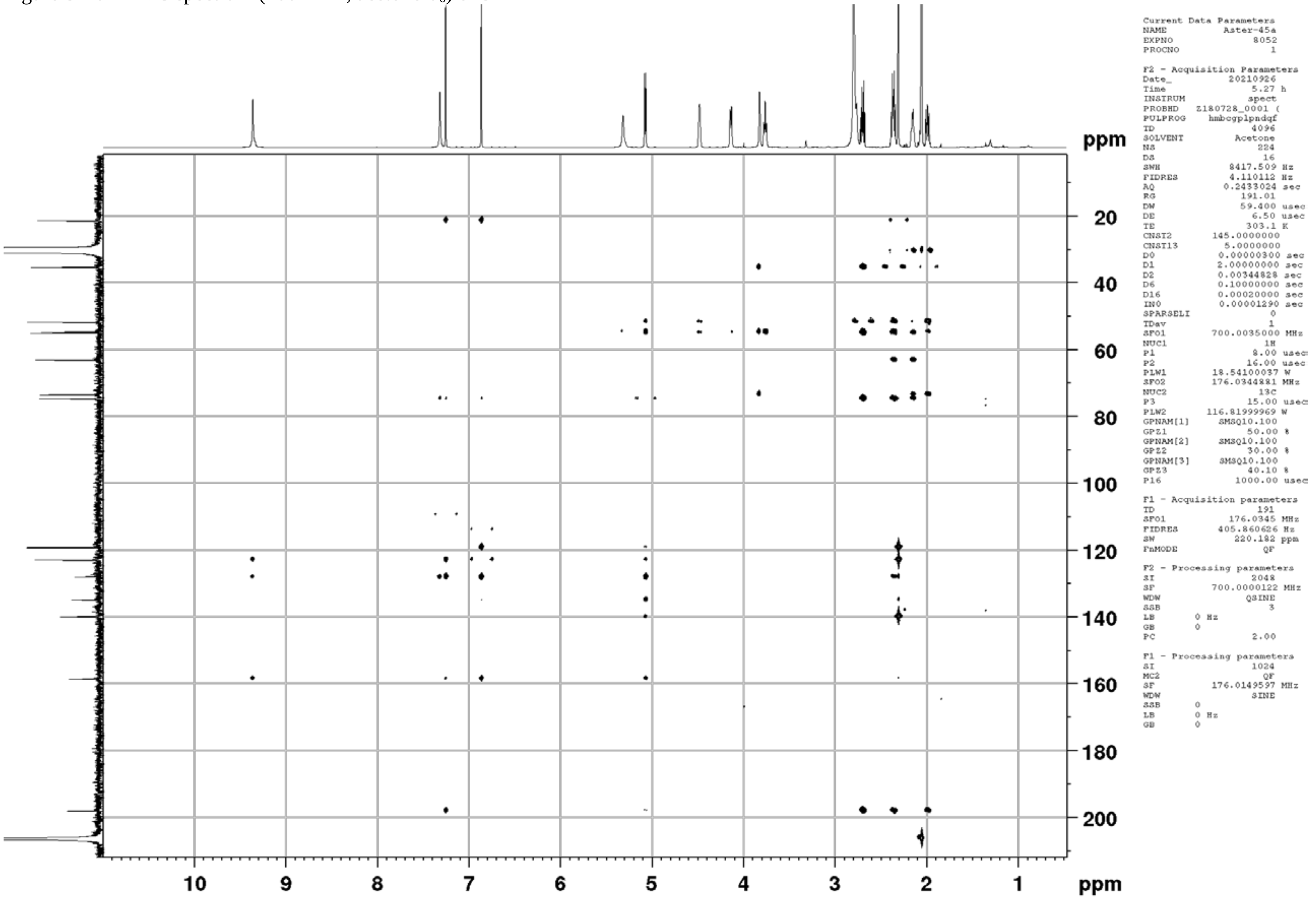

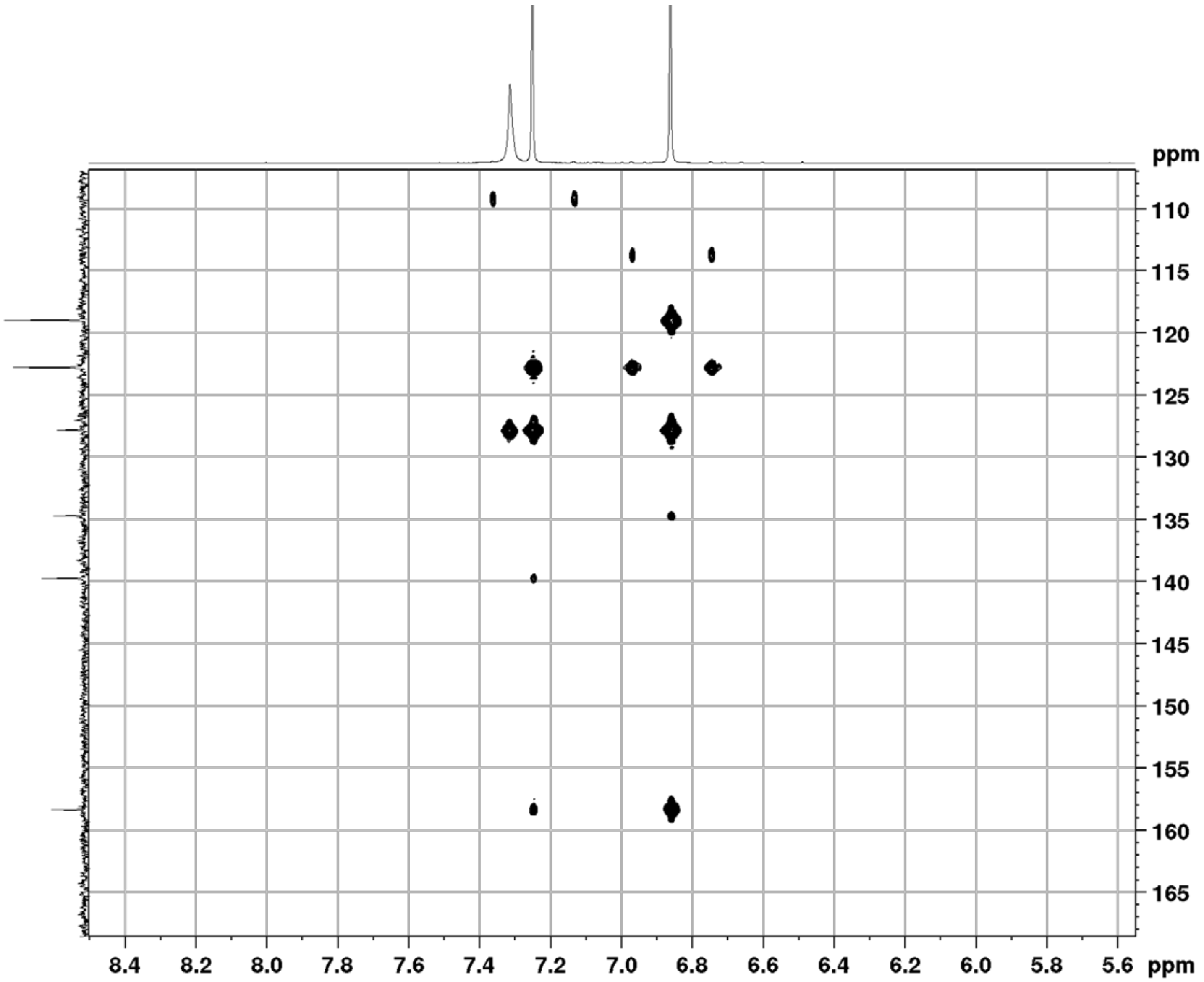


```
Current Data Parameters
NAME            Aster-45a
EXPNO                8052
PROCNO                  1

F2 - Acquisition Parameters
Date_            20210926
Time                5.27 h
INSTRUM             spect
PROBHD   Z180728_0001 (
PULPROG     hmbcgplpndqf
TD                   4096
SOLVENT           Acetone
NS                    224
DS                     16
SWH             8417.509 Hz
FIDRES          4.110112 Hz
AQ             0.2433024 sec
RG                 191.01
DW                 59.400 usec
DE                   6.50 usec
TE                  303.1 K
CNST2         145.0000000
CNST13          5.0000000
D0             0.00000300 sec
D1             2.00000000 sec
D2             0.00344828 sec
D6             0.10000000 sec
D16            0.00020000 sec
IN0            0.00001290 sec
SPARSELI                0
TDav                    1
SFO1        700.0035000 MHz
NUC1                   1H
P1                   8.00 usec
P2                  16.00 usec
PLW1          18.54100037 W
SFO2        176.0344881 MHz
NUC2                  13C
P3                  15.00 usec
PLW2         116.81999969 W
GPNAM[1]      SMSQ10.100
GPZ1                50.00 %
GPNAM[2]      SMSQ10.100
GPZ2                30.00 %
GPNAM[3]      SMSQ10.100
GPZ3                40.10 %
P16               1000.00 usec

F1 - Acquisition parameters
TD                    191
SFO1             176.0345 MHz
FIDRES         405.860626 Hz
SW                220.182 ppm
FnMODE                 QF

F2 - Processing parameters
SI                   2048
SF            700.0000122 MHz
WDW                 QSINE
SSB                     3
LB         0 Hz
GB         0
PC                   2.00

F1 - Processing parameters
SI                   1024
MC2                    QF
SF            176.0149597 MHz
WDW                  SINE
SSB        0
LB         0 Hz
GB         0
```

Figure S25. ROESY spectrum (700 MHz, acetone-d$_6$) of **3**

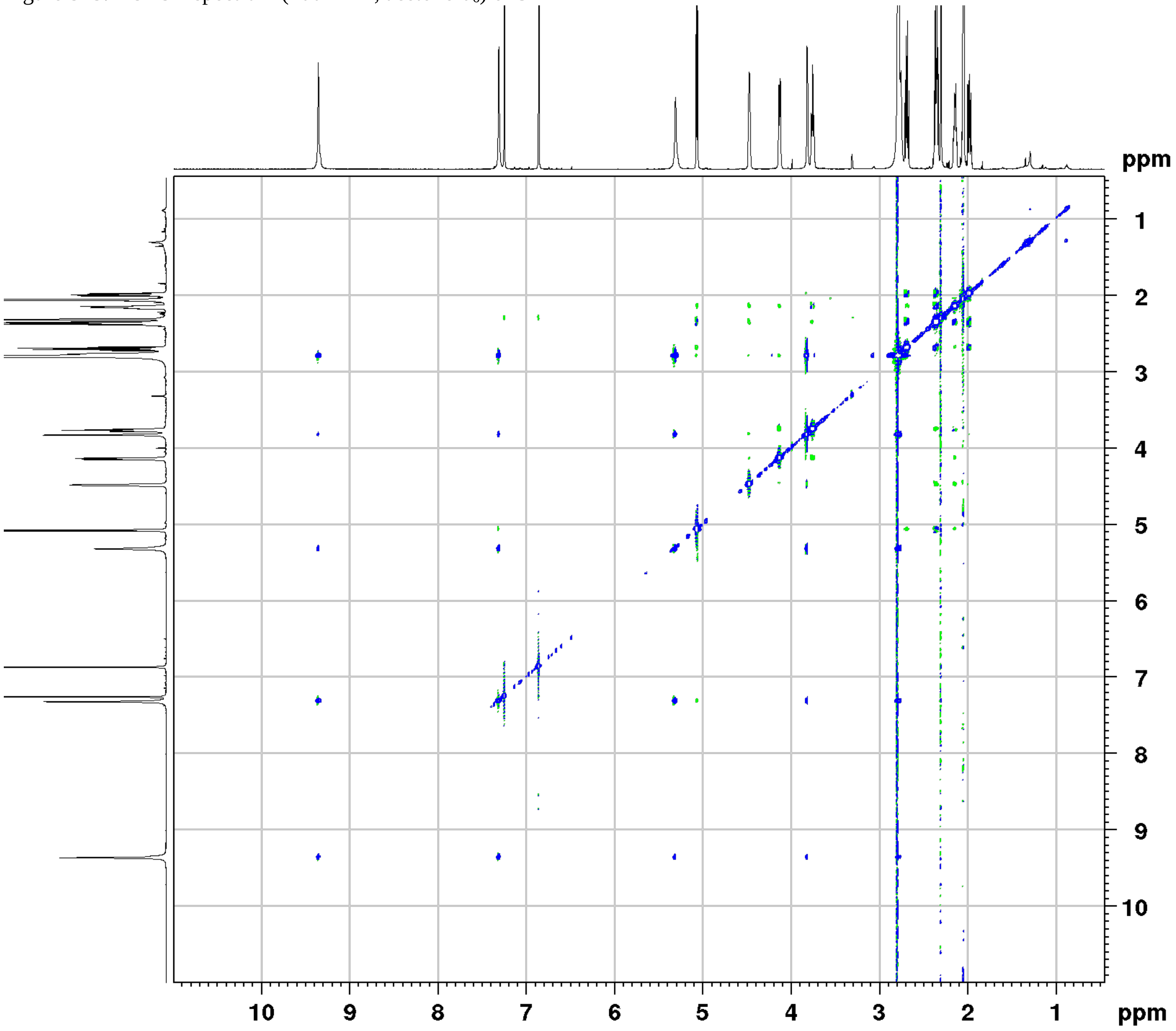


```
Current Data Parameters
NAME             Aster-45a
EXPNO                 6030
PROCNO                   1

F2 - Acquisition Parameters
Date_             20210924
Time                 18.47 h
INSTRUM              spect
PROBHD       Z180728_0001 (
PULPROG          roesyph.2
TD                    4096
SOLVENT            Acetone
NS                      96
DS                      16
SWH               8417.509 Hz
FIDRES            4.110112 Hz
AQ               0.2433024 sec
RG                       2
DW                  59.400 usec
DE                    6.50 usec
TE                   303.2 K
D0              0.00005031 sec
D1              2.00000000 sec
D12             0.00002000 sec
IN0             0.00011880 sec
L4                     877
P15              270000.00 usec
TDav                     1
SFO1           700.0035000 MHz
NUC1                    1H
P1                    8.00 usec
P25                 154.00 usec
PLW1           18.54100037 W
PLW27           0.20014000 W

F1 - Acquisition parameters
TD                     512
SFO1              700.0035 MHz
FIDRES           32.880894 Hz
SW                  12.025 ppm
FnMODE                TPPI

F2 - Processing parameters
SI                    4096
SF             700.0000120 MHz
WDW                   SINE
SSB                      2
LB            0 Hz
GB            0
PC                    4.00

F1 - Processing parameters
SI                    1024
MC2                   TPPI
SF             700.0000102 MHz
WDW                   SINE
SSB                      2
LB            0 Hz
GB            0
```

Figure S26. $^{1}H$ spectrum (500 MHz, acetone-$d_6$) of **4**

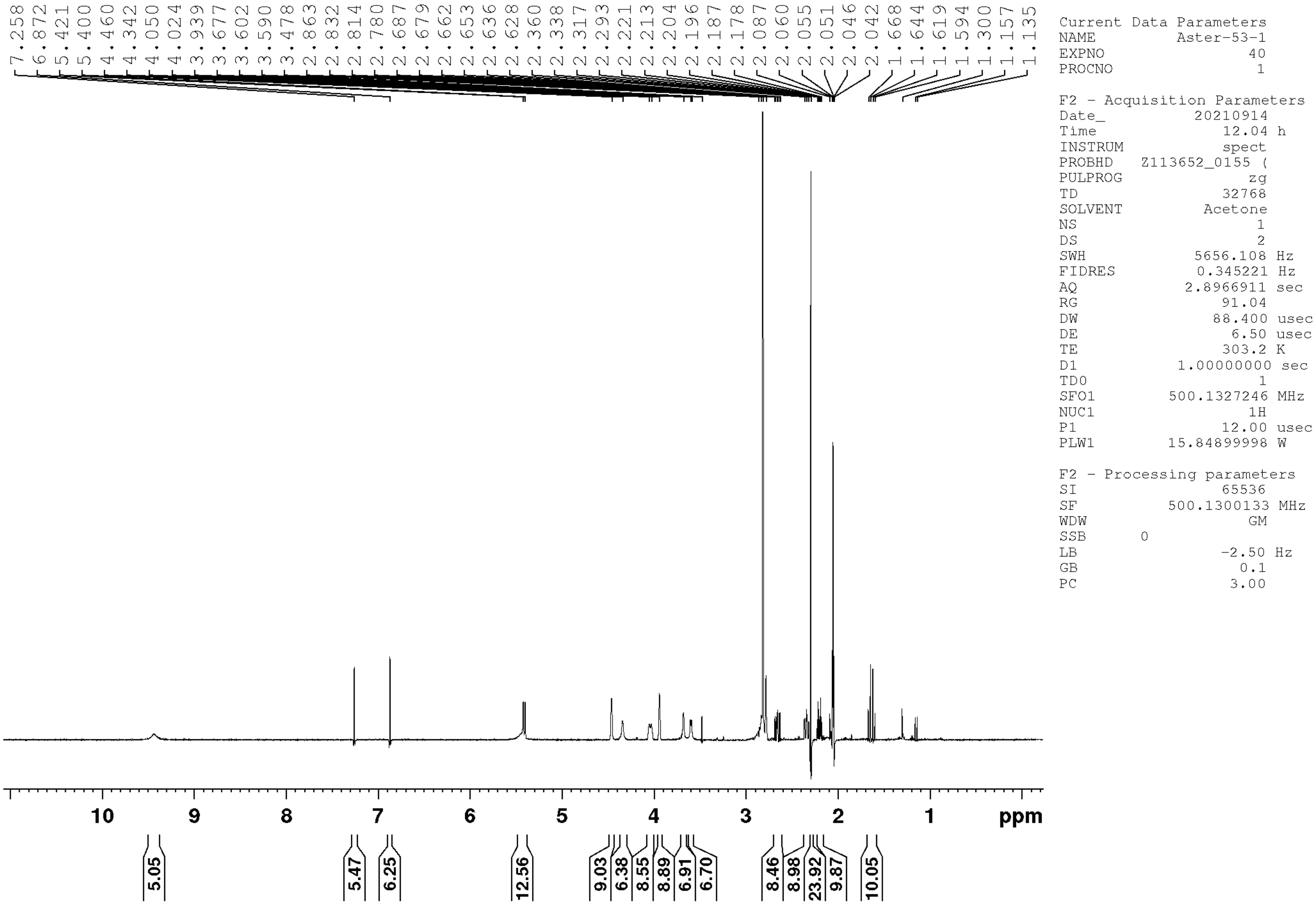

Figure S27. $^{13}$C spectrum (125.77 MHz, acetone-$d_6$) of **4**

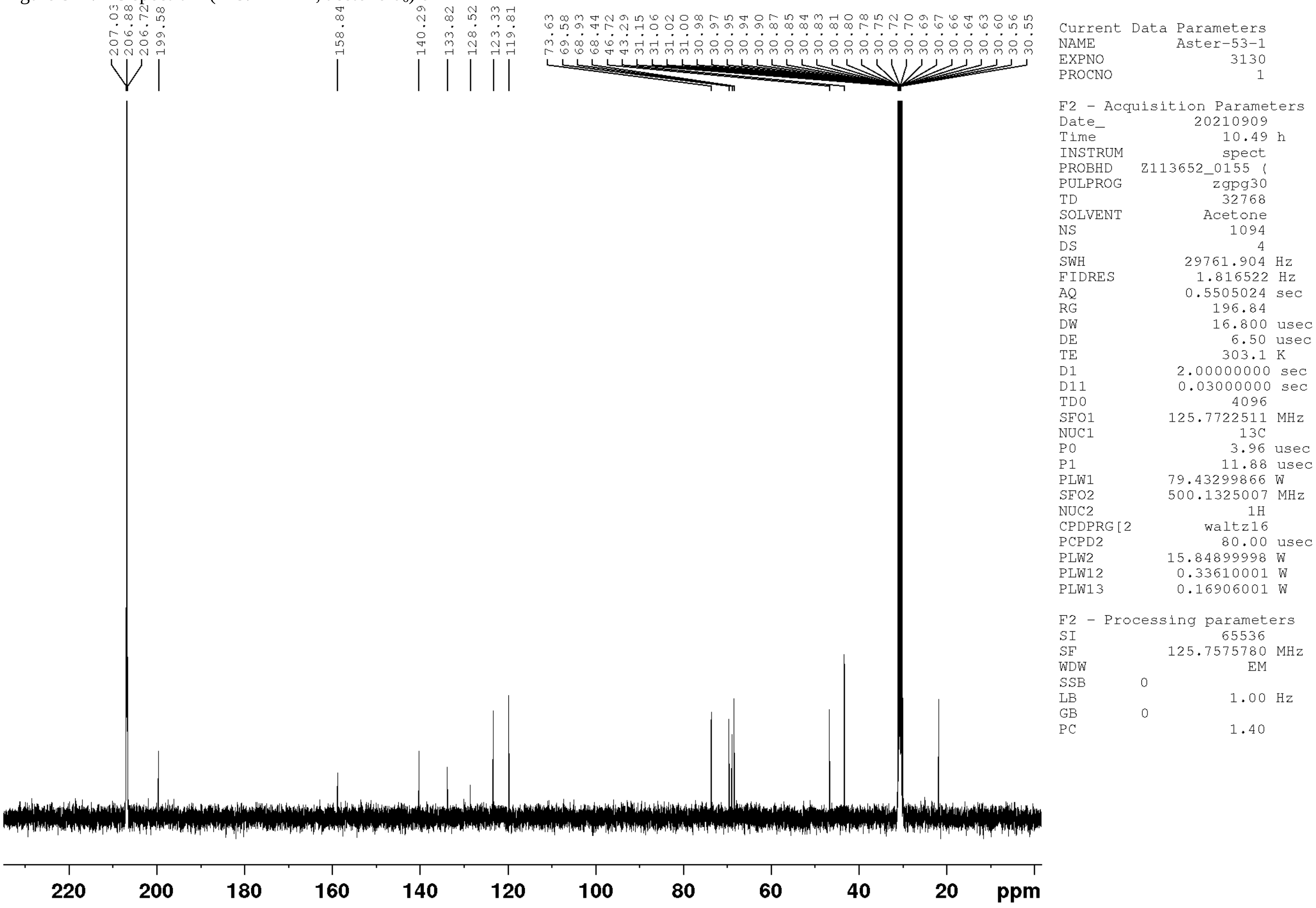

Figure S28. DEPT-135 NMR spectrum (125.77 MHz, acetone-$d_6$) of **4**

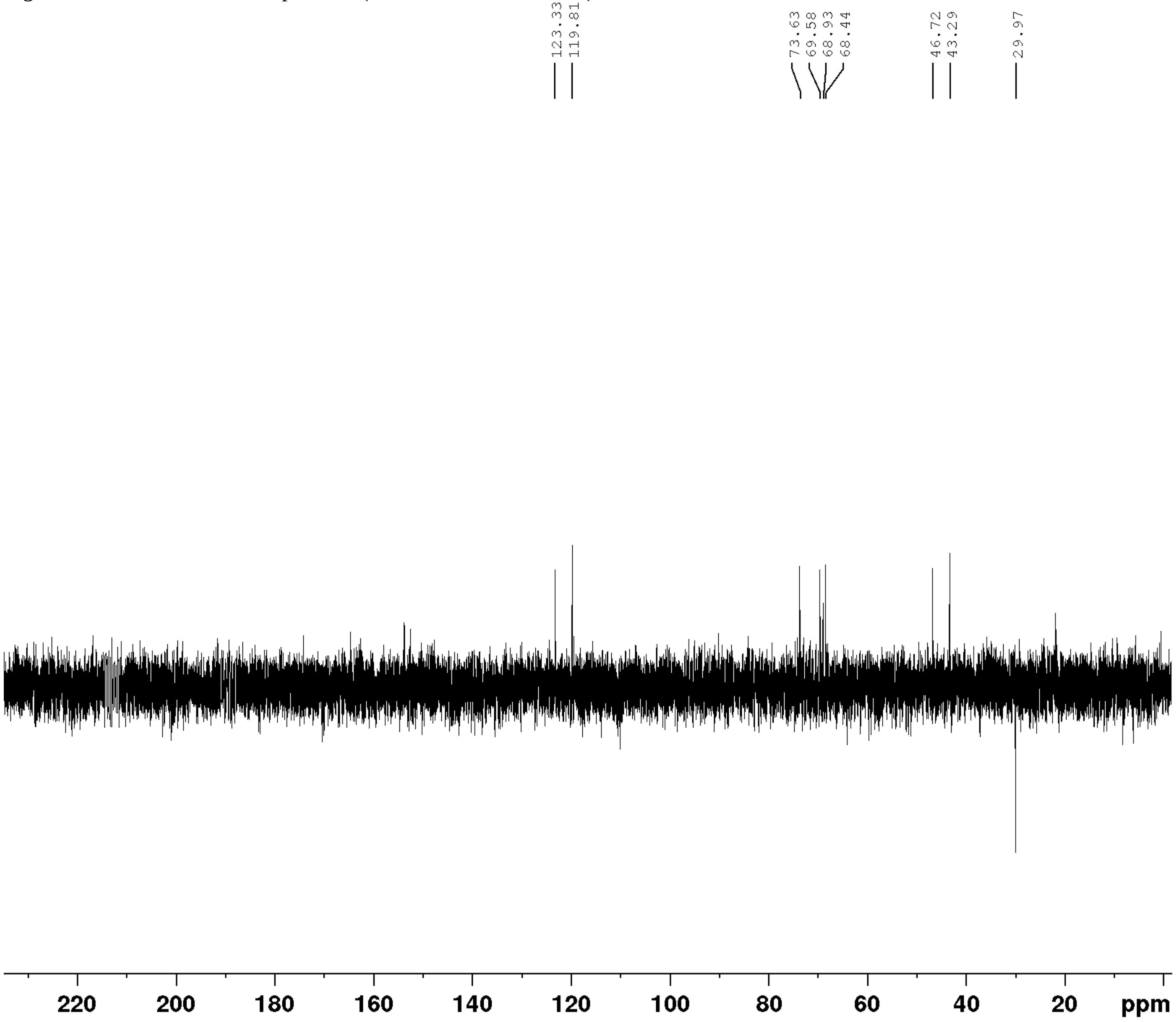


Current Data Parameters
NAME Aster-53-1
EXPNO 4000
PROCNO 1

F2 - Acquisition Parameters
Date_ 20210909
Time 10.56 h
INSTRUM spect
PROBHD Z113652_0155 (
PULPROG dept135
TD 32768
SOLVENT Acetone
NS 113
DS 32
SWH 29761.904 Hz
FIDRES 1.816522 Hz
AQ 0.5505024 sec
RG 196.84
DW 16.800 usec
DE 6.50 usec
TE 303.1 K
CNST2 145.0000000
D1 2.00000000 sec
D2 0.00344828 sec
D12 0.00002000 sec
TD0 4096
SFO1 125.7722511 MHz
NUC1 13C
P1 11.88 usec
P2 23.76 usec
PLW1 79.43299866 W
SFO2 500.1325007 MHz
NUC2 1H
CPDPRG[2 waltz16
P3 11.65 usec
P4 23.30 usec
PCPD2 80.00 usec
PLW2 15.84899998 W
PLW12 0.33610001 W

F2 - Processing parameters
SI 65536
SF 125.7575780 MHz
WDW EM
SSB 0
LB 1.00 Hz
GB 0
PC 1.50

Figure S29. COSY-45 spectrum (500 MHz, acetone-$d_6$) of **4**

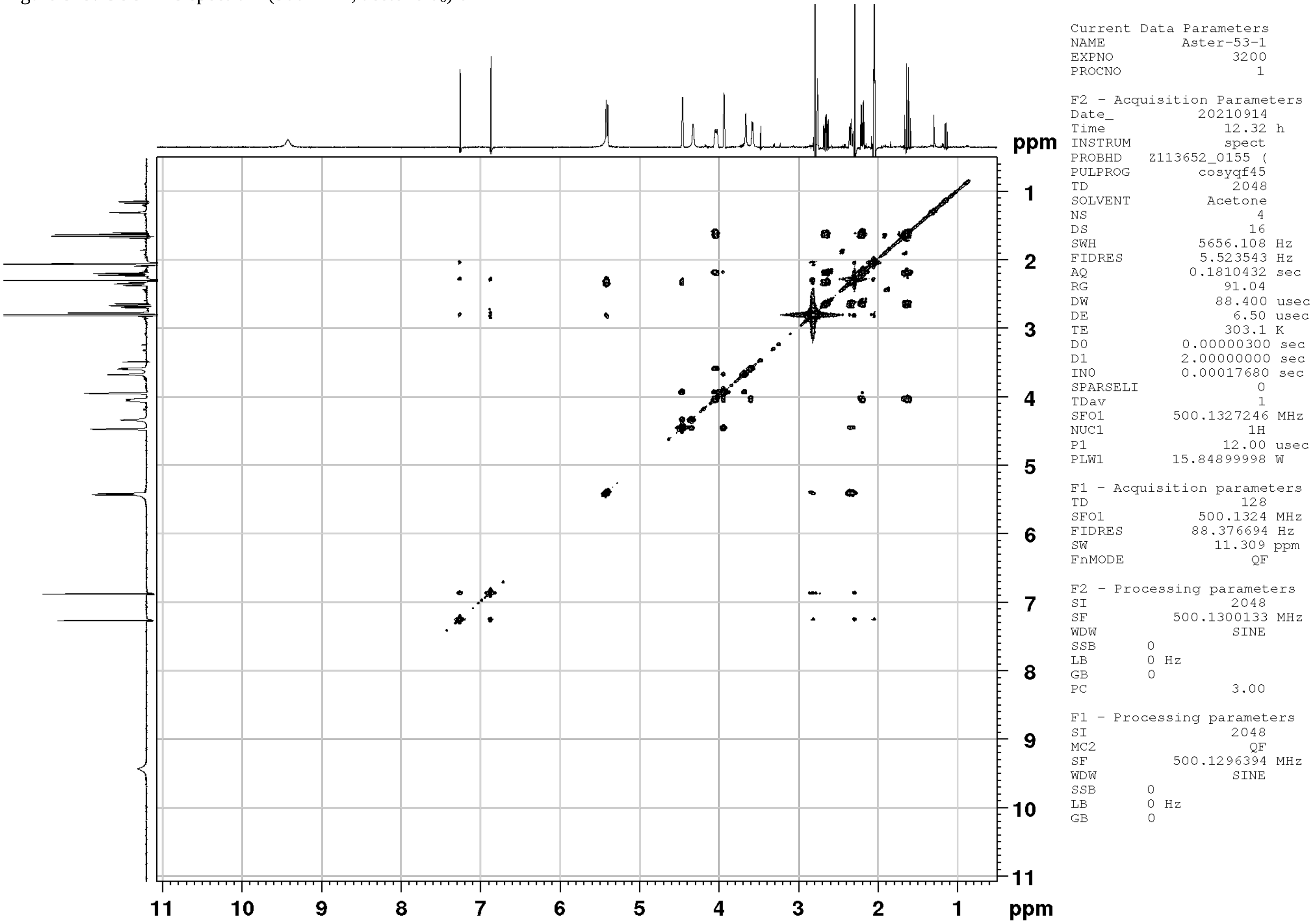

Figure S30. HSQC spectrum (500 MHz, acetone-$d_6$) of **4**

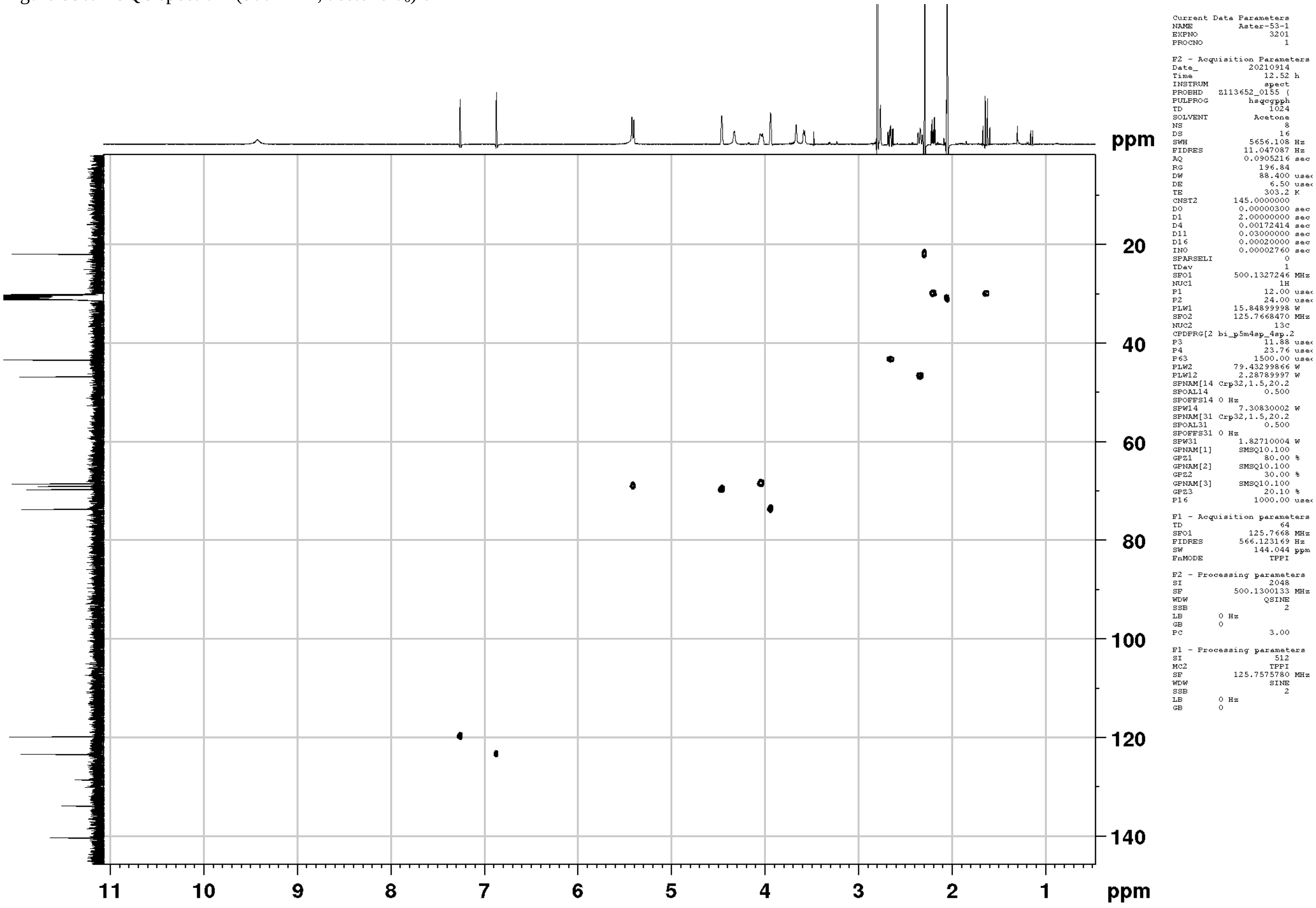

Figure S31. HMBC spectrum (500 MHz, acetone-$d_6$) of **4**

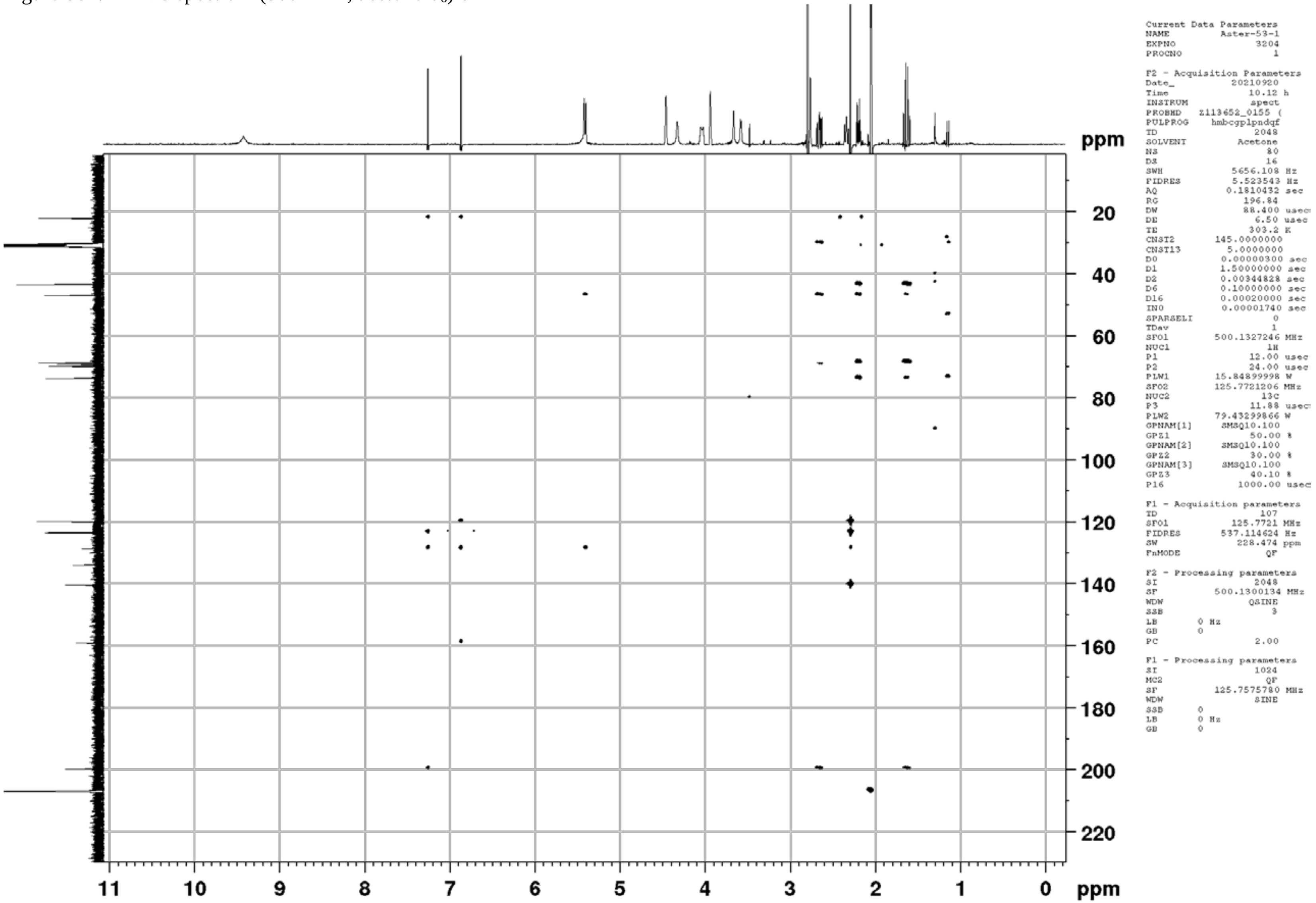

Figure S32. ROESY spectrum (500 MHz, acetone-$d_6$) of **4**

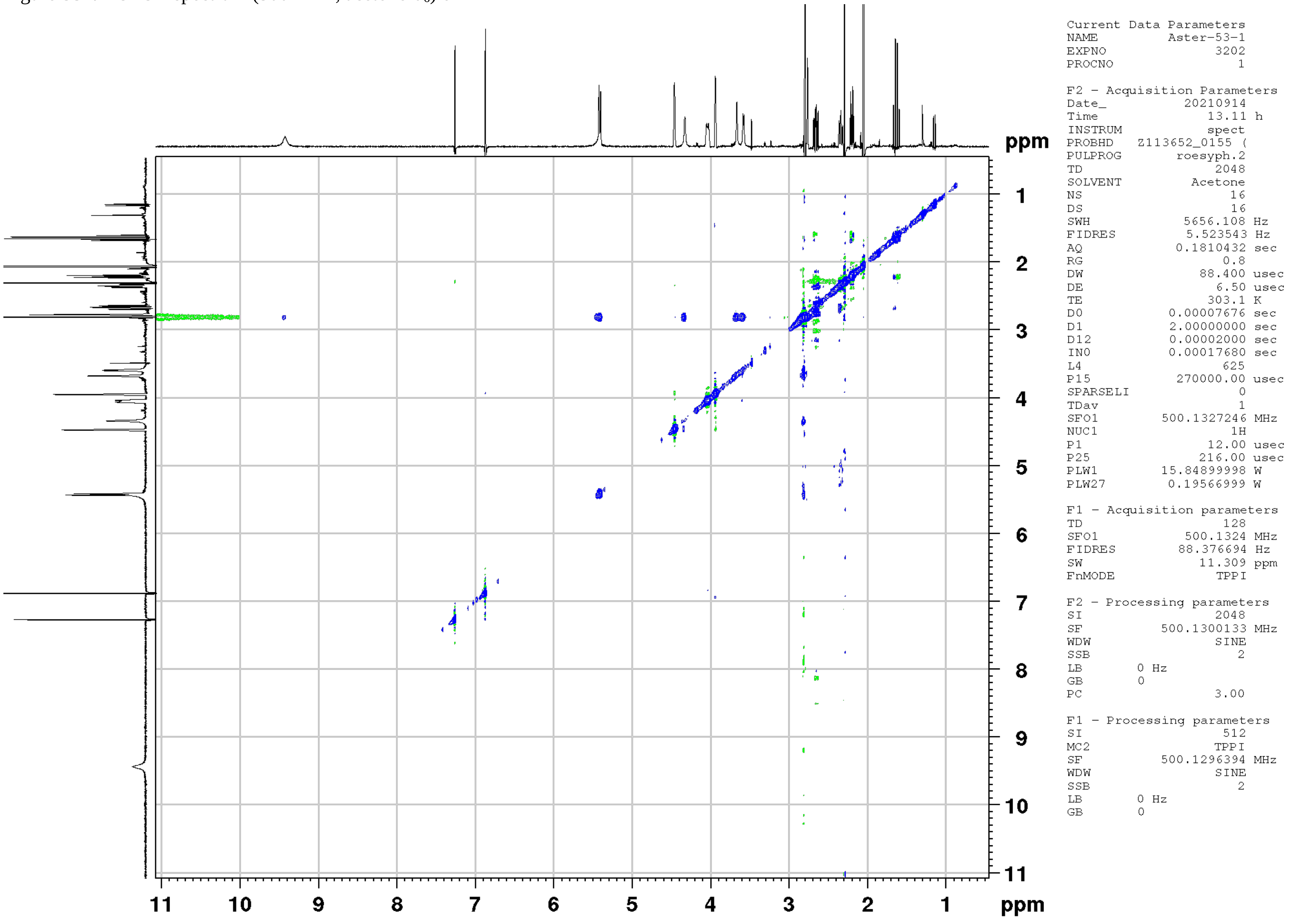

Figure S33. $^{1}H$ NMR spectrum (500 MHz, $CDCl_3$) of **5**

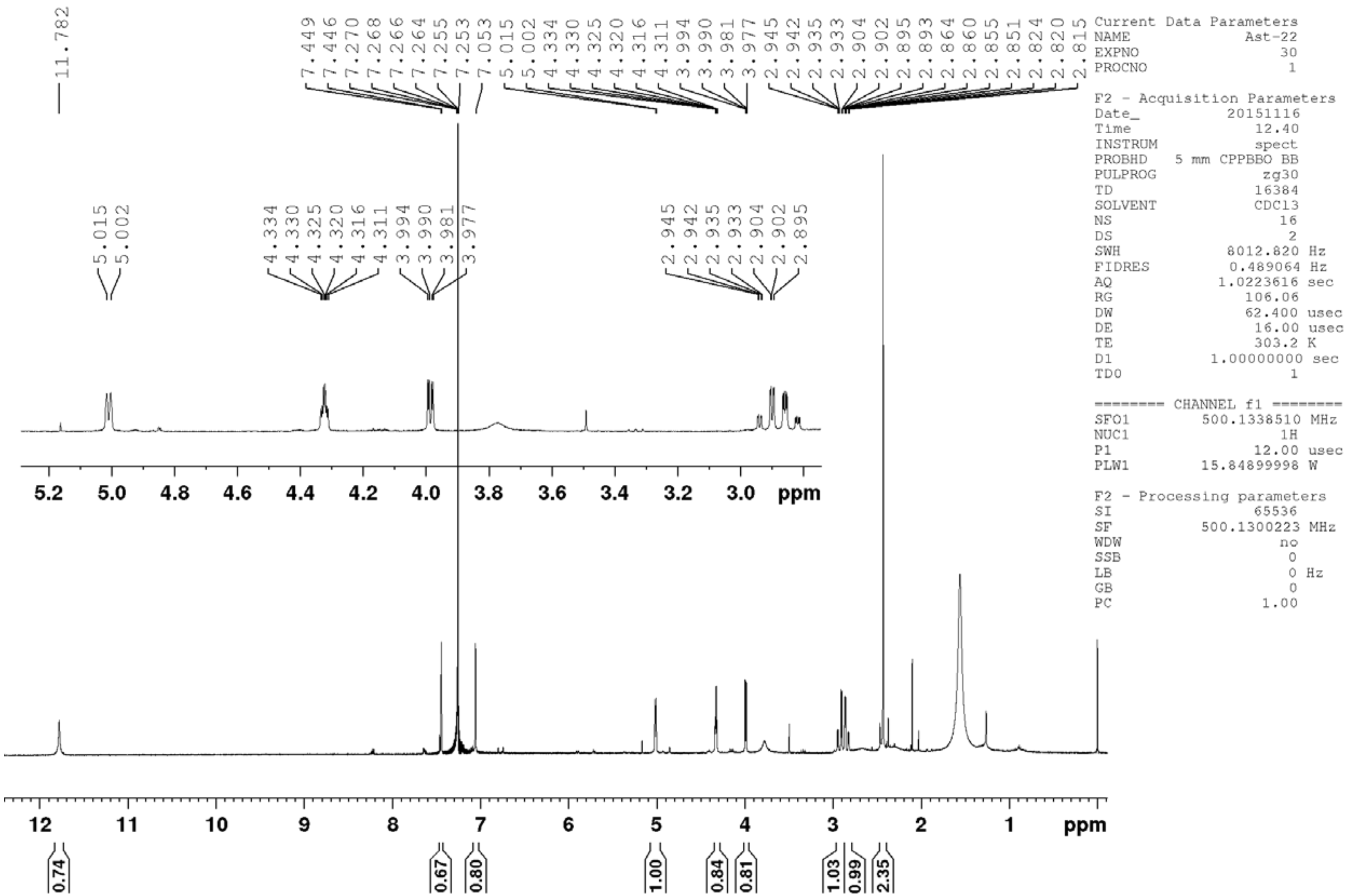

Figure S34. $^{13}C$ NMR spectrum (125.77 MHz, $CDCl_3$) of **5**

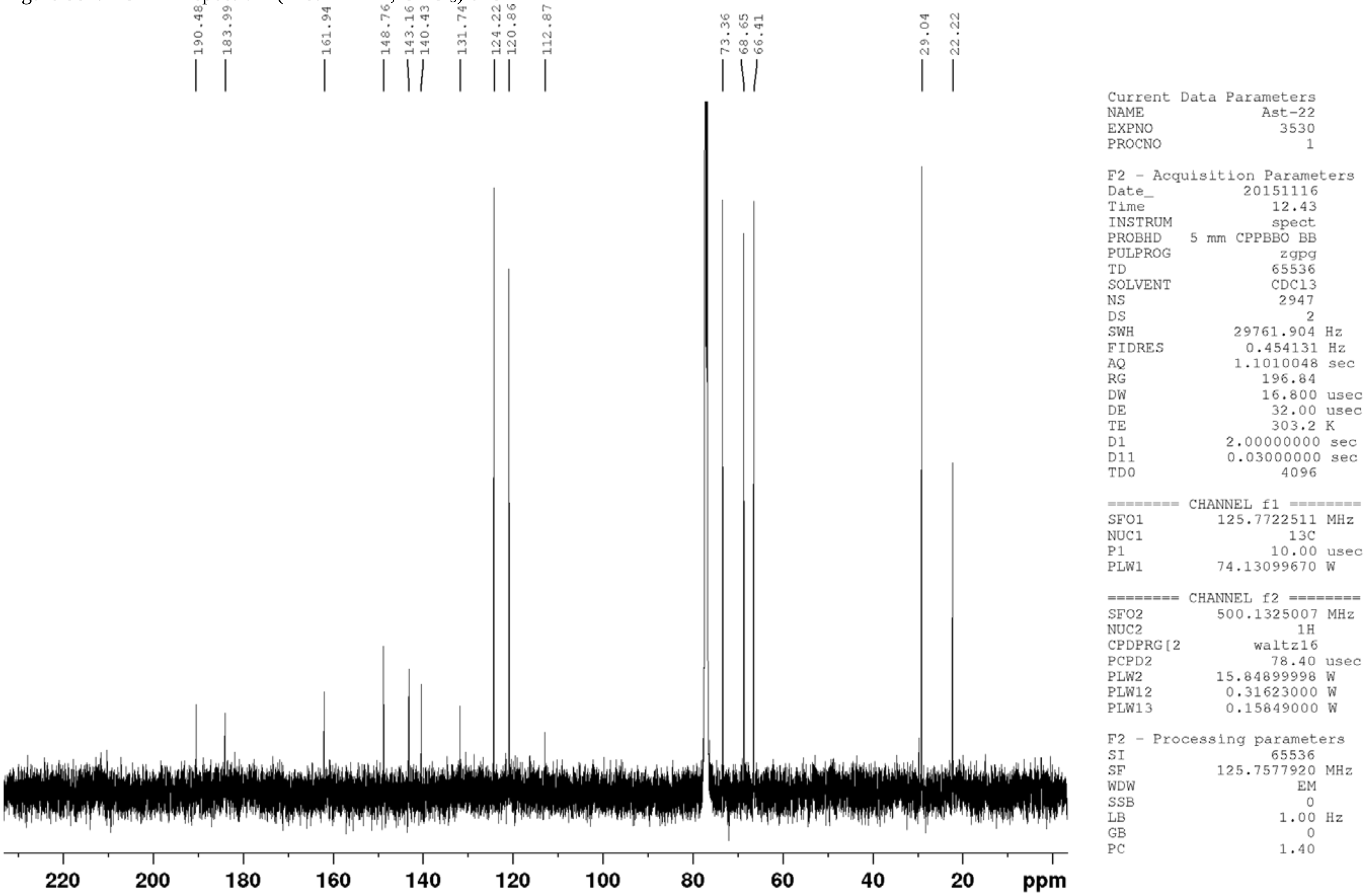

Figure S35. DEPT-135 spectrum (125.77 MHz, $CDCl_3$) of **5**

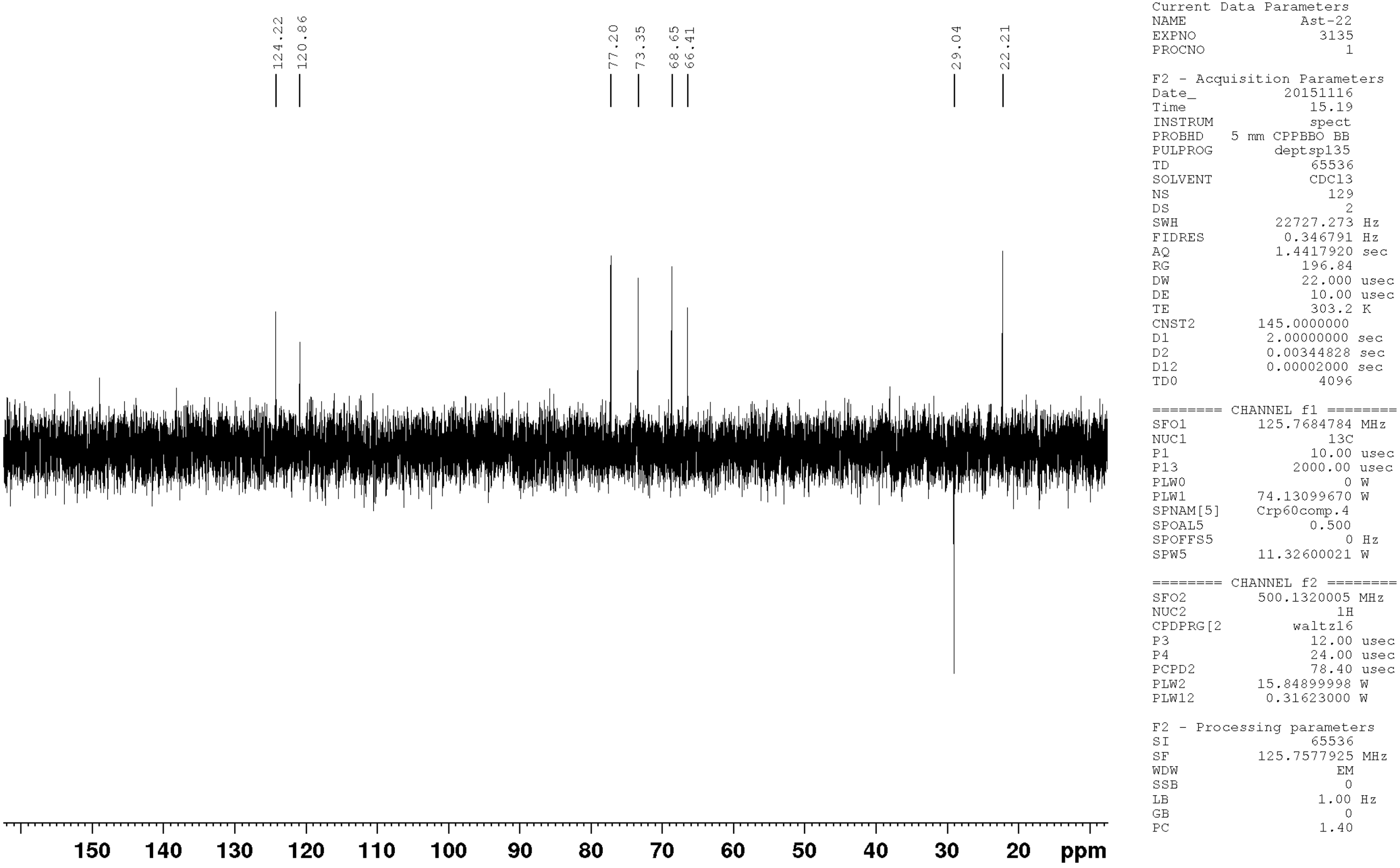

Figure S36. $^{1}H$ NMR spectrum (700 MHz, acetone-$d_6$) of **6**

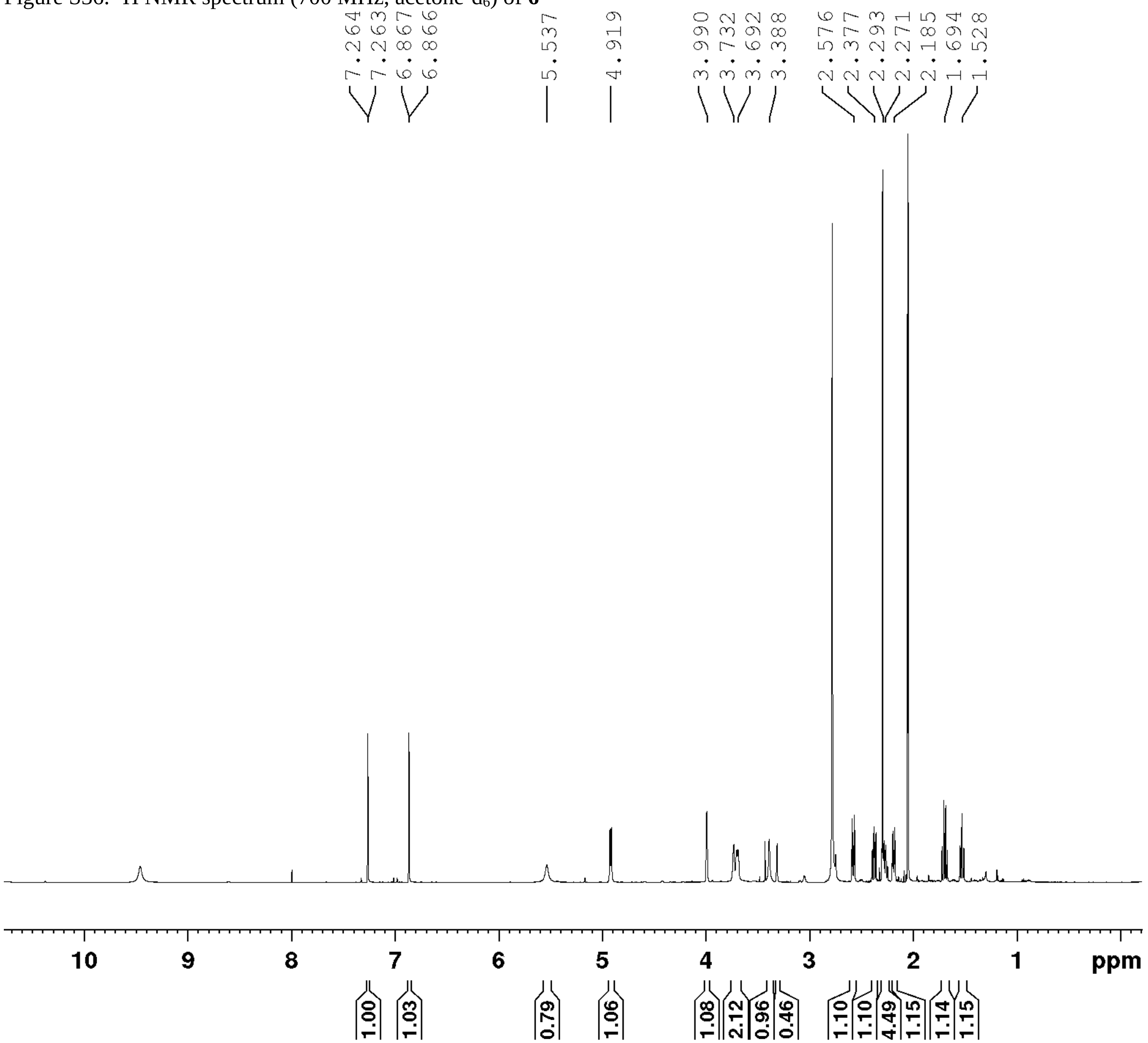

Figure S37. $^{13}C$ NMR spectrum (125.77 MHz, acetone-$d_6$) of **6**

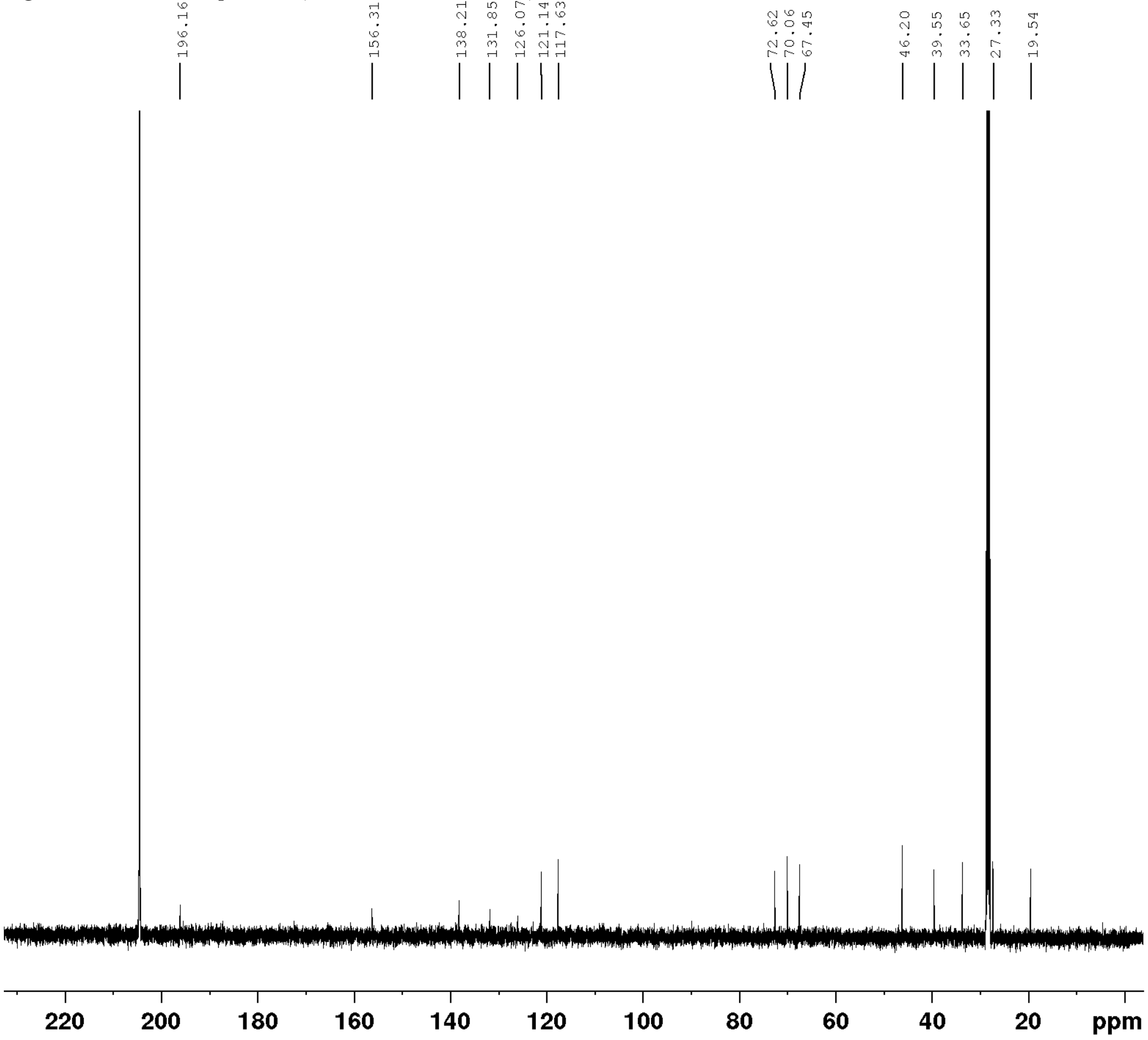


| | |
|---|---|
| Current Data Parameters | |
| NAME | Aster-44a |
| EXPNO | 3535 |
| PROCNO | 1 |
| F2 - Acquisition Parameters | |
| Date_ | 20210817 |
| Time | 15.53 h |
| INSTRUM | spect |
| PROBHD | Z113652_0155 ( |
| PULPROG | zgpg30 |
| TD | 32768 |
| SOLVENT | Acetone |
| NS | 256 |
| DS | 2 |
| SWH | 29761.904 Hz |
| FIDRES | 1.816522 Hz |
| AQ | 0.5505024 sec |
| RG | 196.84 |
| DW | 16.800 usec |
| DE | 6.50 usec |
| TE | 308.2 K |
| D1 | 2.00000000 sec |
| D11 | 0.03000000 sec |
| TD0 | 512 |
| SFO1 | 125.7722511 MHz |
| NUC1 | 13C |
| P0 | 3.96 usec |
| P1 | 11.88 usec |
| PLW1 | 79.43299866 W |
| SFO2 | 500.1325007 MHz |
| NUC2 | 1H |
| CPDPRG[2 | waltz16 |
| PCPD2 | 80.00 usec |
| PLW2 | 15.84899998 W |
| PLW12 | 0.33610001 W |
| PLW13 | 0.16906001 W |
| F2 - Processing parameters | |
| SI | 65536 |
| SF | 125.7578616 MHz |
| WDW | EM |
| SSB | 0 |
| LB | 1.00 Hz |
| GB | 0 |
| PC | 1.50 |

Figure S38. DEPT-135 spectrum (125.77 MHz, acetone-$d_6$) of **6**

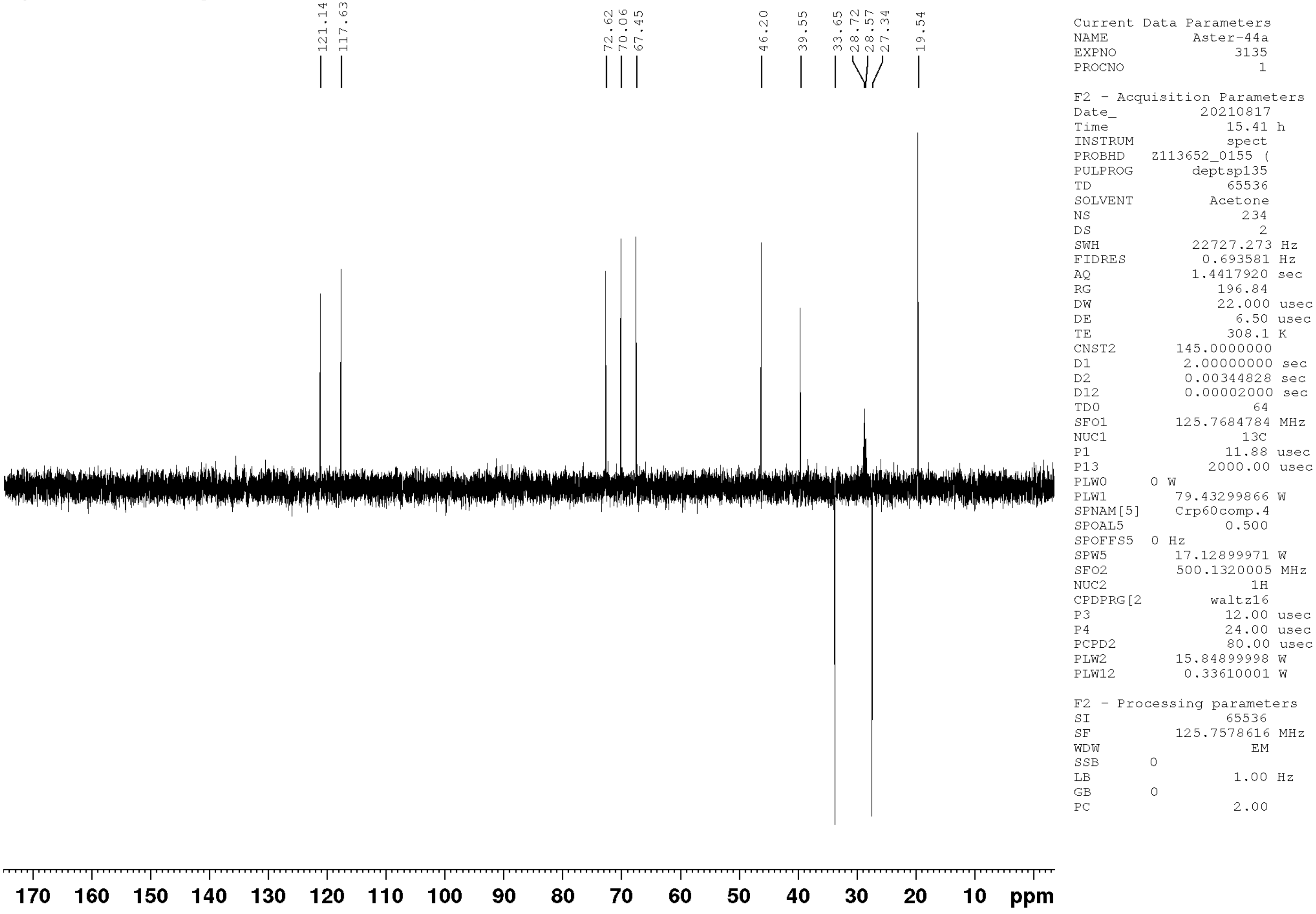

Figure S39. COSY-45 spectrum (700 MHz, acetone-$d_6$) of **6**

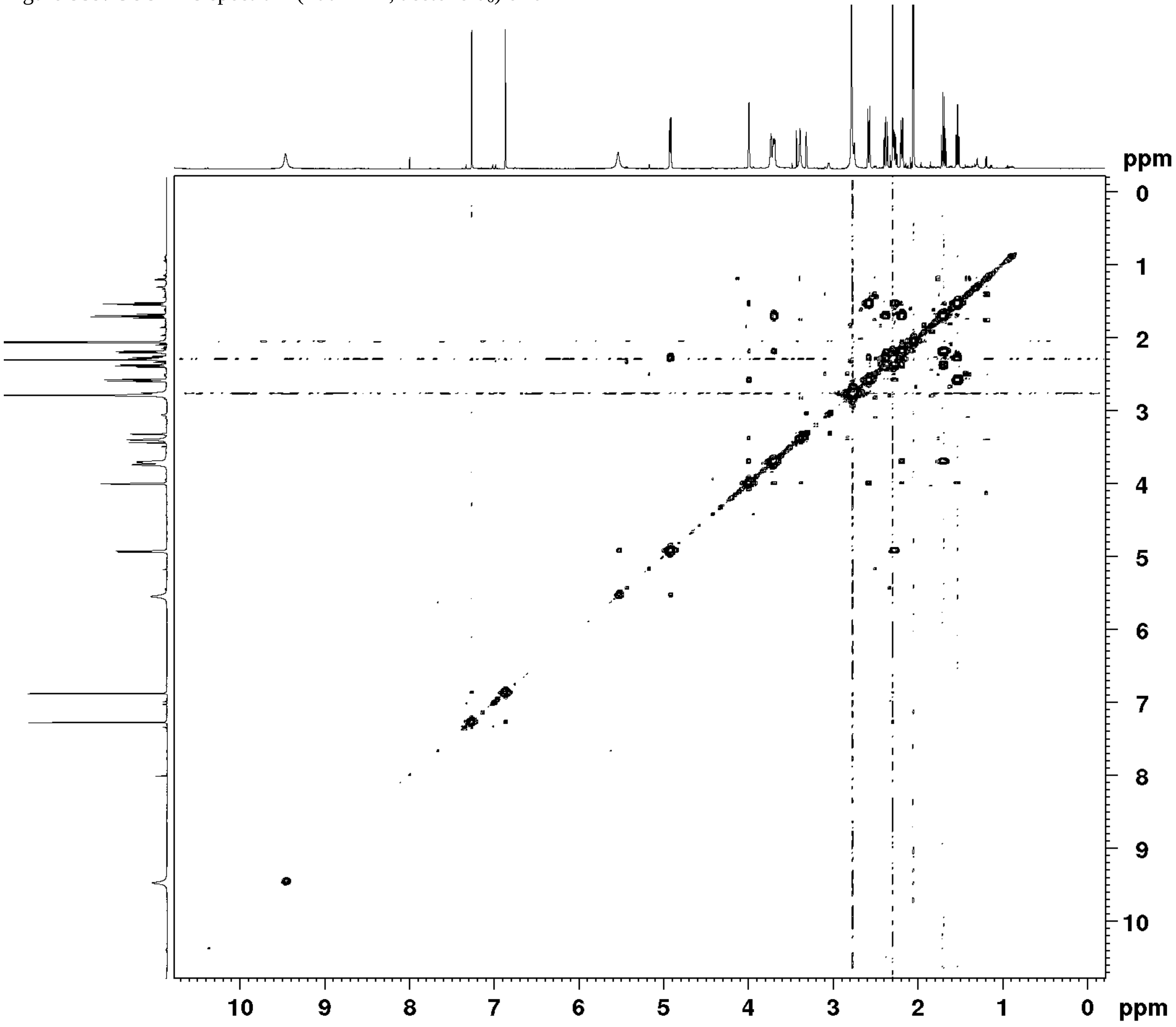


```
Current Data Parameters
NAME            Aster-44a
EXPNO                 455
PROCNO                  1

F2 - Acquisition Parameters
Date_           20210813
Time               18.01 h
INSTRUM            spect
PROBHD   Z180728_0001 (
PULPROG         cosygpqf
TD                  4096
SOLVENT          Acetone
NS                    16
DS                     8
SWH             7692.308 Hz
FIDRES          3.756010 Hz
AQ             0.2662400 sec
RG                191.01
DW                65.000 usec
DE                  6.50 usec
TE                 308.0 K
D0            0.00000300 sec
D1            2.00000000 sec
D13           0.00000400 sec
D16           0.00020000 sec
IN0           0.00013000 sec
SPARSELI               0
TDav                   1
SFO1         700.0037100 MHz
NUC1                  1H
P0                  4.00 usec
P1                  8.00 usec
PLW1         18.54100037 W
GPNAM[1]        SINE.100
GPZ1               10.00 %
P16              1000.00 usec

F1 - Acquisition parameters
TD                   128
SFO1            700.0026 MHz
FIDRES        120.192307 Hz
SW                10.989 ppm
FnMODE                QF

F2 - Processing parameters
SI                  2048
SF           700.0000135 MHz
WDW                 SINE
SSB      0
LB       0 Hz
GB       0
PC                  1.00

F1 - Processing parameters
SI                  2048
MC2                   QF
SF           699.9988935 MHz
WDW                 SINE
SSB      0
LB       0 Hz
GB       0
```

Figure S40. HSQC spectrum (700 MHz, acetone-$d_6$) of **6**

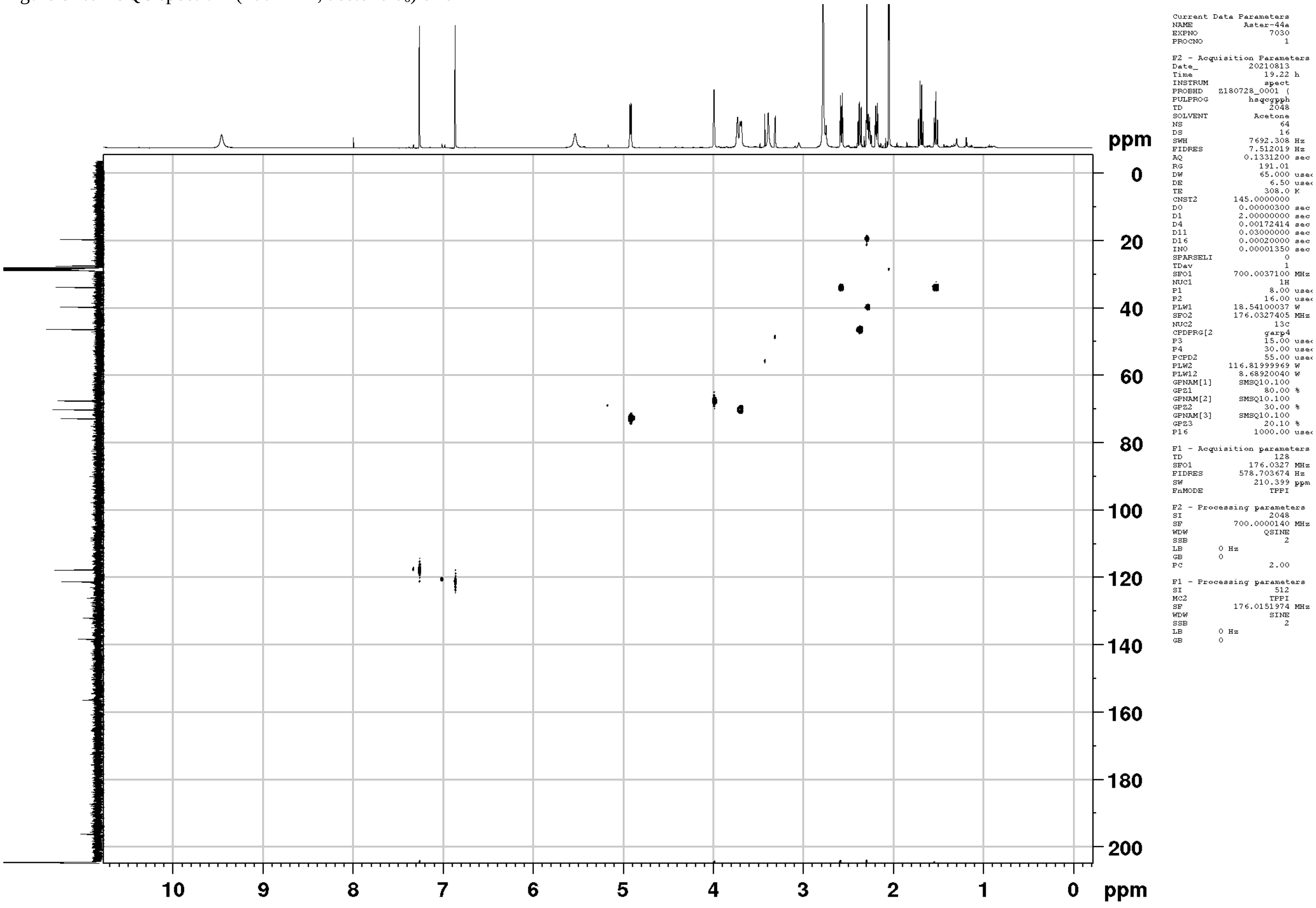

Figure S41. HMBC spectrum (700 MHz, acetone-$d_6$) of **6**

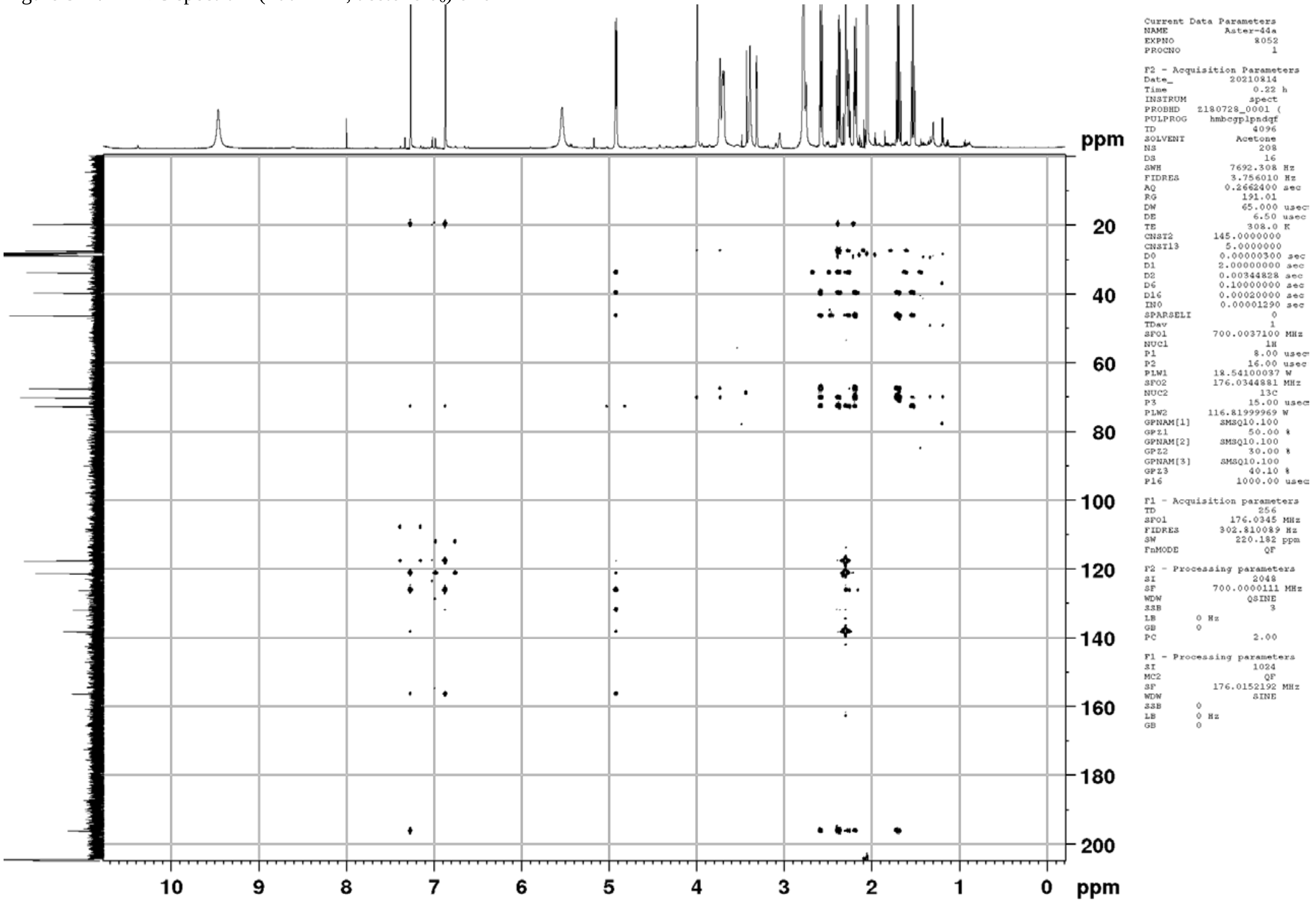

Figure S42. ROESY spectrum (500 MHz, acetone-$d_6$) of **6**

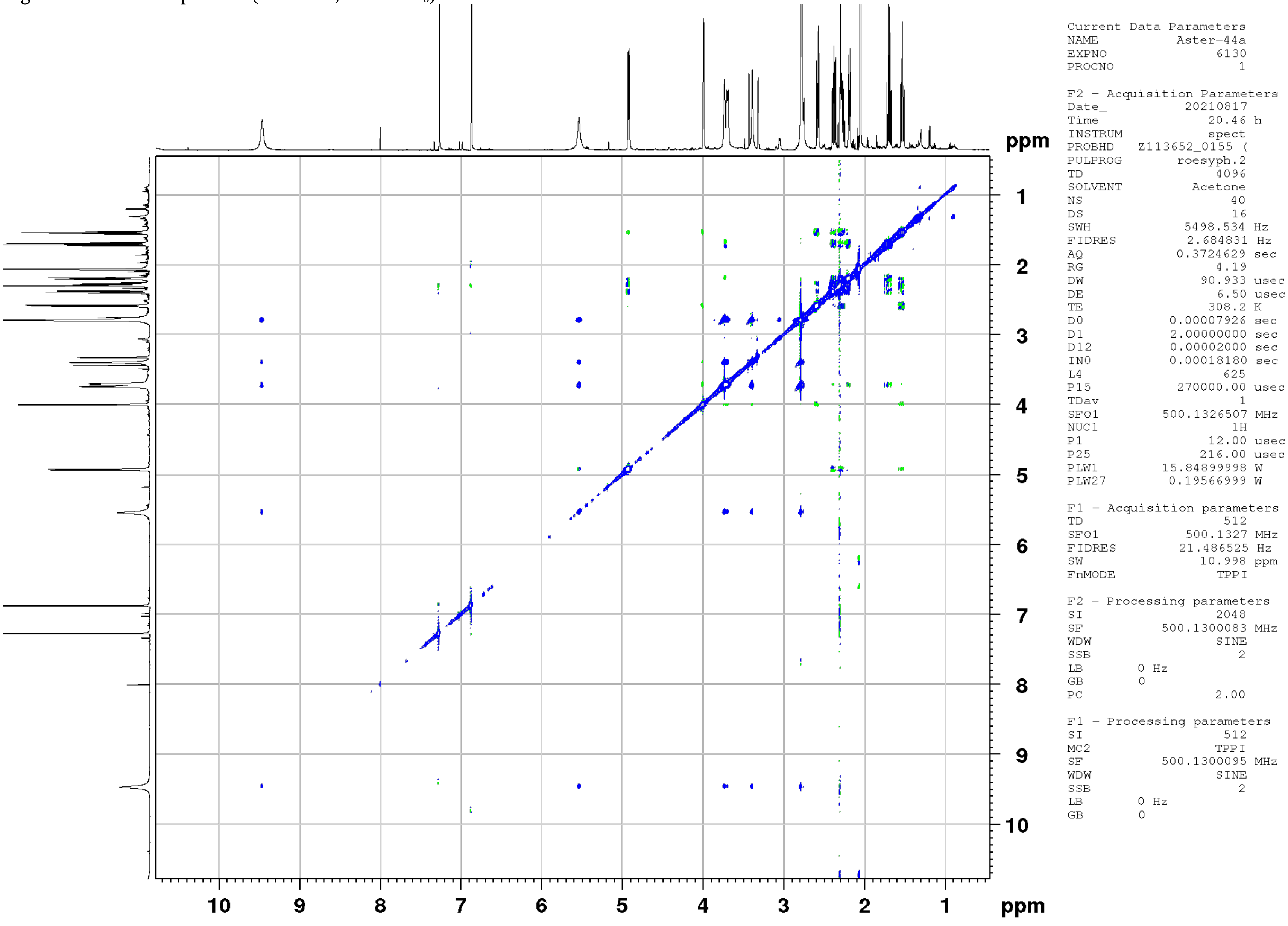

Figure S43. $^{1}$H NMR spectrum (700 MHz, acetone-$d_6$) of **7**

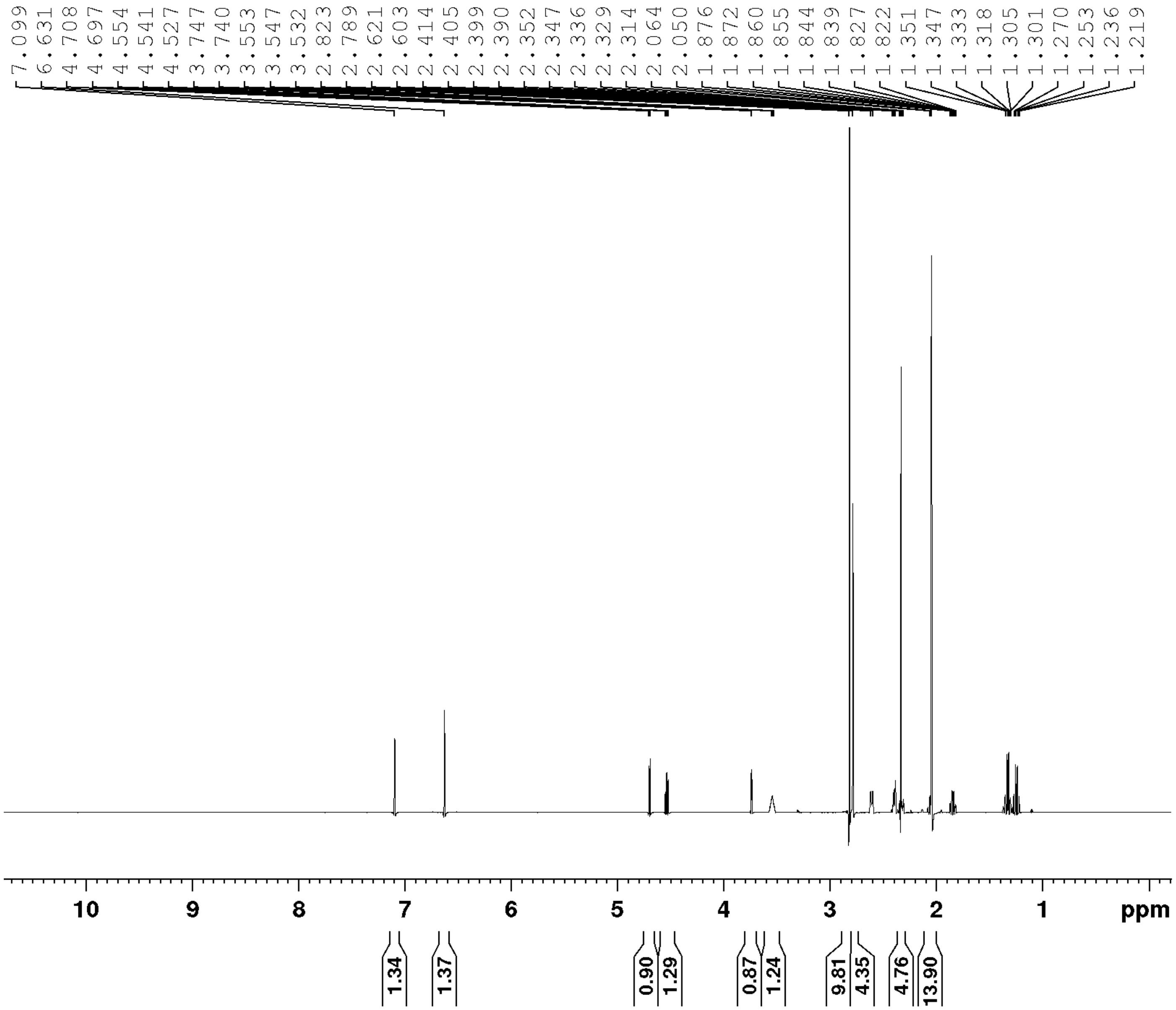


```
Current Data Parameters
NAME           Aster-41a
EXPNO                  1
PROCNO                 1

F2 - Acquisition Parameters
Date_          20210501
Time              10.58 h
INSTRUM           spect
PROBHD   Z112726_0001 (
PULPROG              zg
TD                32768
SOLVENT         Acetone
NS                    1
DS                    0
SWH          11160.714 Hz
FIDRES        0.681196 Hz
AQ           1.4680064 sec
RG                  181
DW               44.800 usec
DE                 6.50 usec
TE                303.0 K
D1       0 sec
TD0                   1
SFO1       700.0053900 MHz
NUC1                 1H
P1                12.60 usec
PLW1       39.81100082 W

F2 - Processing parameters
SI                32768
SF         700.0000117 MHz
WDW                  GM
SSB      0
LB                -2.50 Hz
GB                 0.08
PC                 4.00
```

Figure S44. $^{13}$C NMR spectrum (176.04 MHz, acetone-$d_6$) of **7**

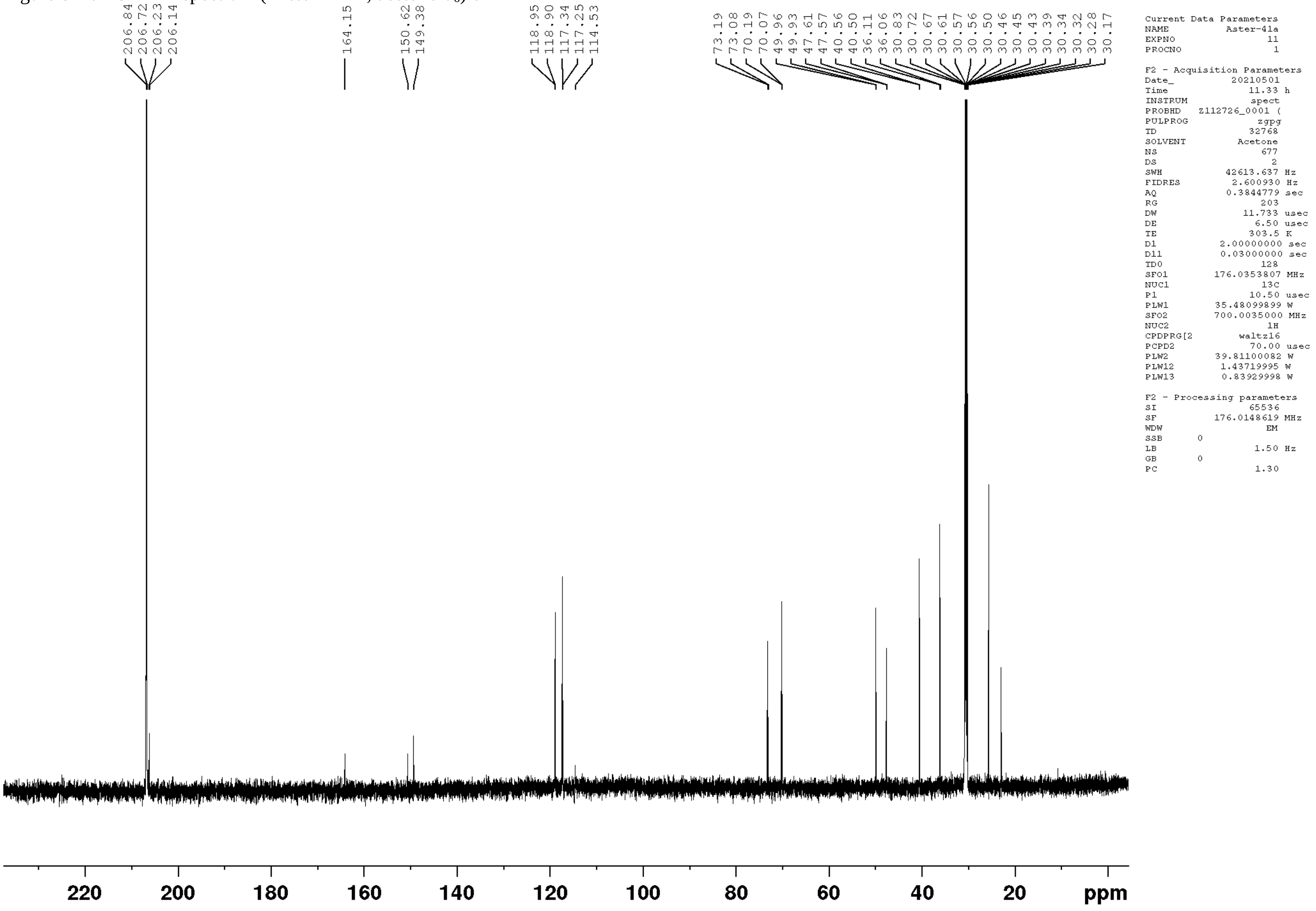

Figure S45. DEPT-135 spectrum (176.04 MHz, acetone-d$_6$) of **7**

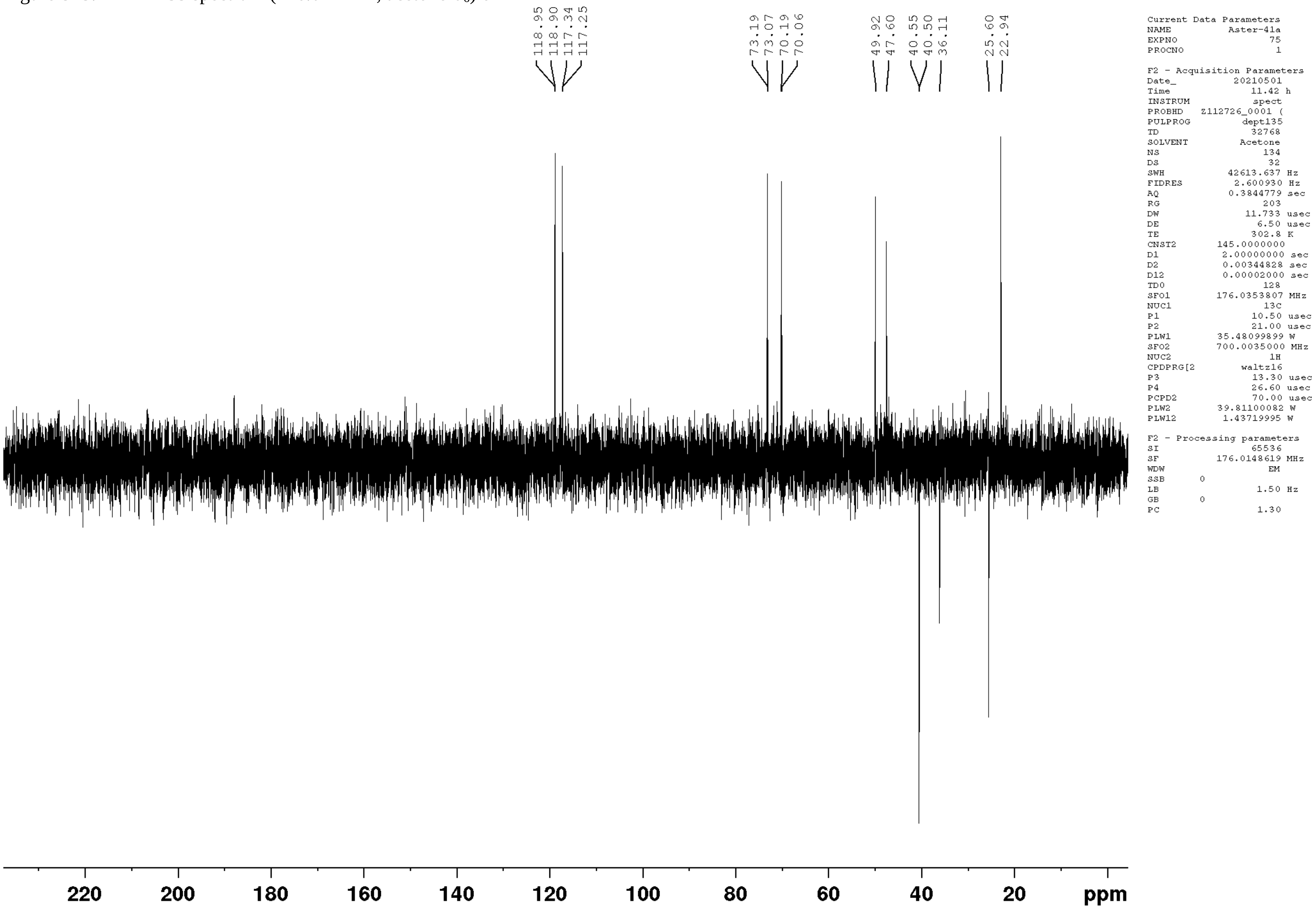

Figure S46. COSY-45 spectrum (700 MHz, acetone-$d_6$) of **7**

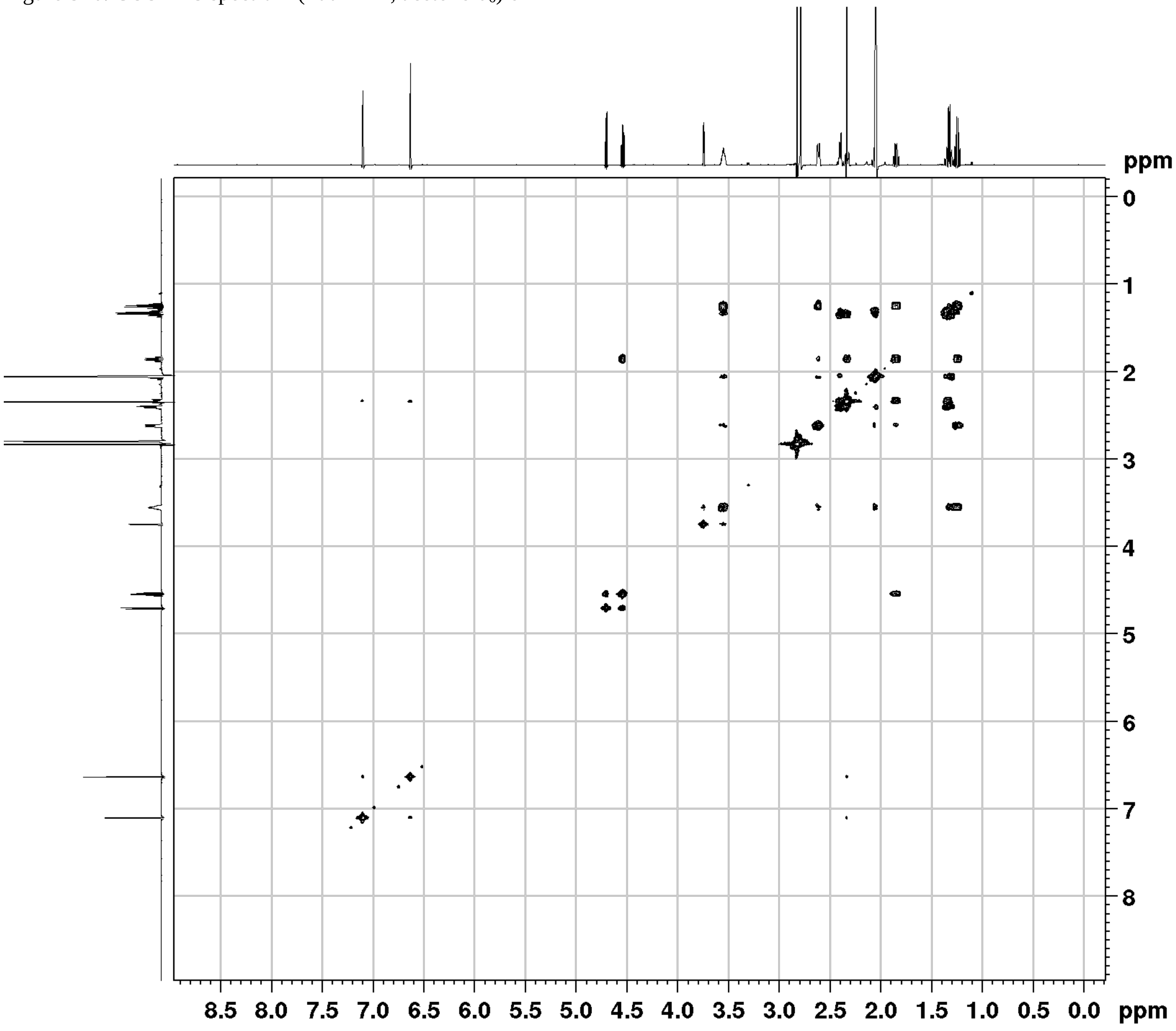


```
Current Data Parameters
NAME          Aster-41a
EXPNO                21
PROCNO                1

F2 - Acquisition Parameters
Date_          20210501
Time              12.01 h
INSTRUM           spect
PROBHD   Z112726_0001 (
PULPROG        cosygpqf
TD                 2048
SOLVENT         Acetone
NS                    8
DS                    8
SWH            6465.517 Hz
FIDRES         6.313982 Hz
AQ            0.1583787 sec
RG                  203
DW               77.333 usec
DE                 6.50 usec
TE                303.0 K
D0           0.00000300 sec
D1           1.20000005 sec
D13          0.00000400 sec
D16          0.00020000 sec
IN0          0.00015460 sec
SPARSELI              0
TDav                  1
SFO1        700.0030515 MHz
NUC1                 1H
P0                12.60 usec
P1                12.60 usec
PLW1        39.81100082 W
GPNAM[1]       SINE.100
GPZ1              10.00 %
P16             1000.00 usec

F1 - Acquisition parameters
TD                  128
SFO1           700.0031 MHz
FIDRES       101.067268 Hz
SW                9.240 ppm
FnMODE               QF

F2 - Processing parameters
SI                 2048
SF          700.0000117 MHz
WDW                SINE
SSB      0
LB       0 Hz
GB       0
PC                 1.00

F1 - Processing parameters
SI                 2048
MC2                  QF
SF          700.0000117 MHz
WDW                SINE
SSB      0
LB       0 Hz
GB       0
```

Figure S47. HSQC spectrum (700 MHz, acetone-$d_6$) of **7**

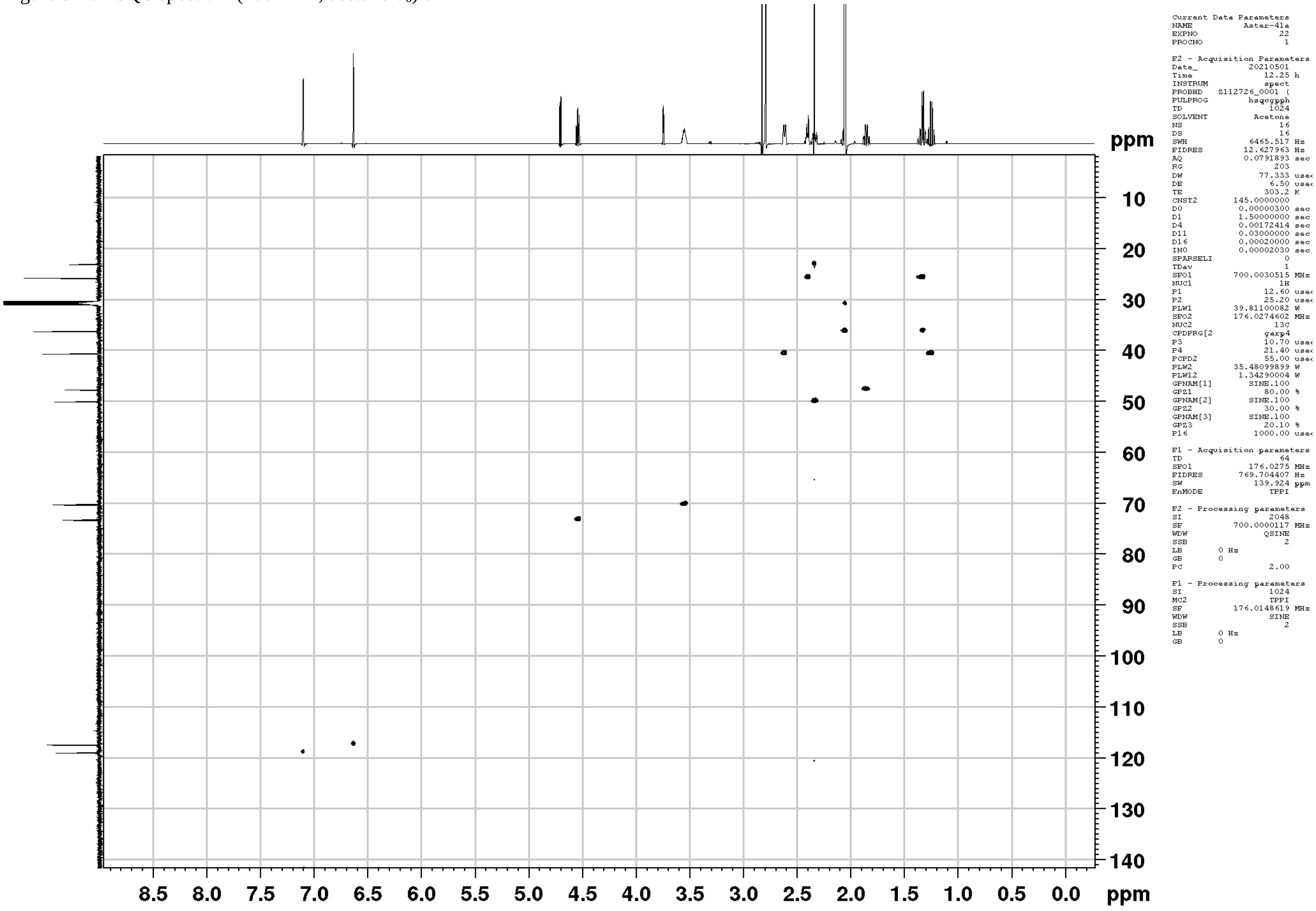

Figure S48. HMBC spectrum (700 MHz, acetone-$d_6$) of **7**

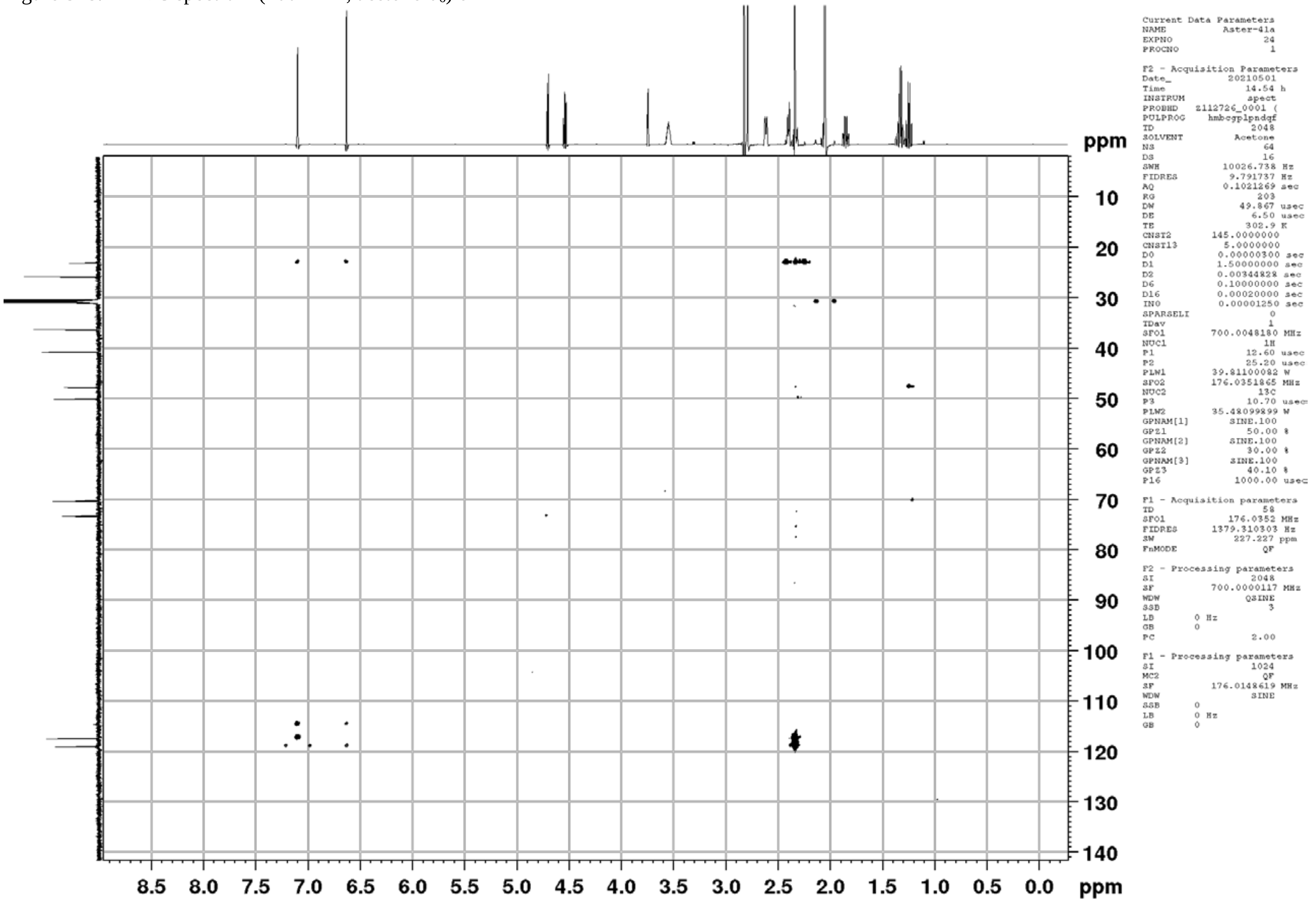

Figure S49. ROESY spectrum (700 MHz, acetone-$d_6$) of **7**

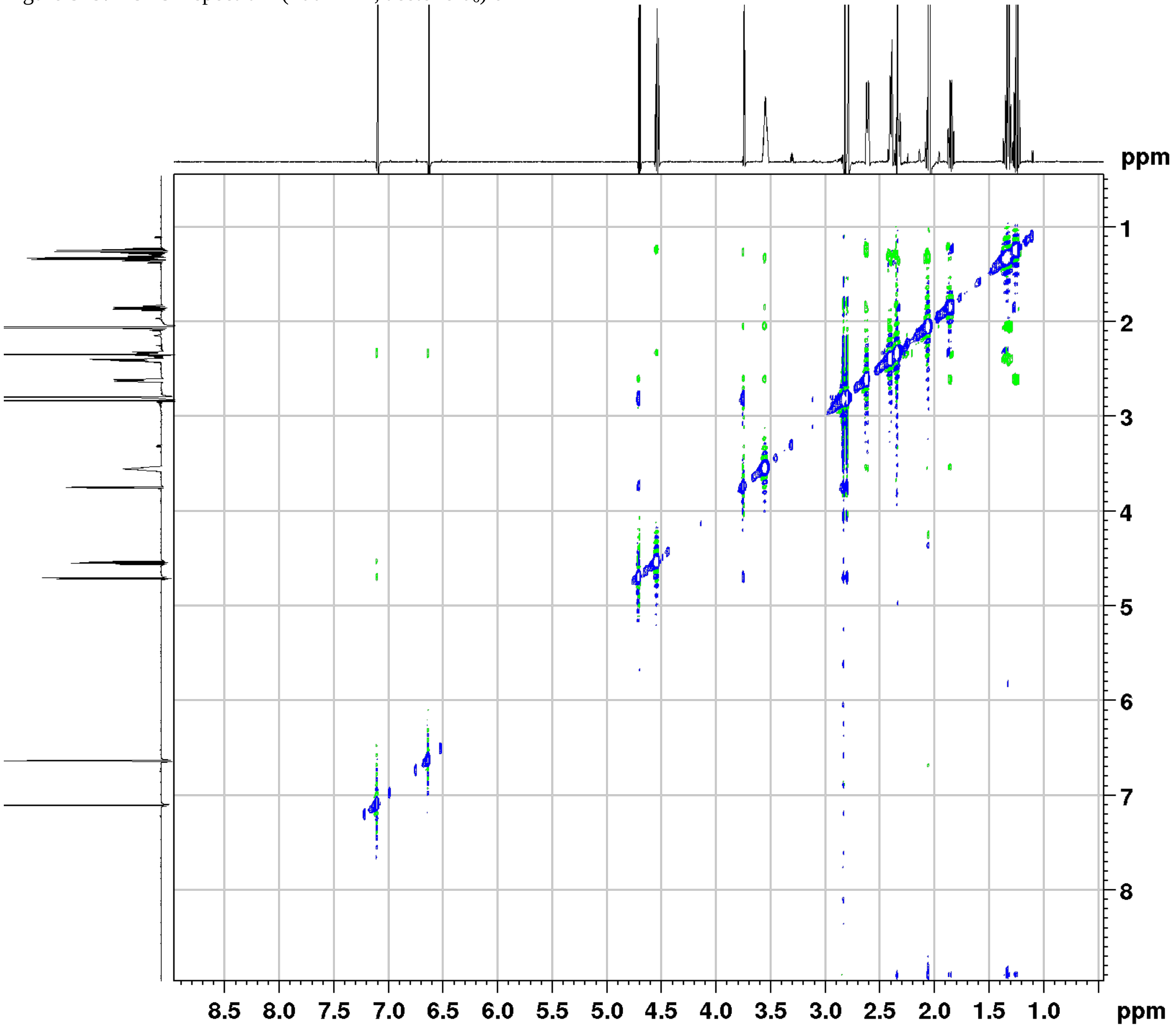


```
Current Data Parameters
NAME            Aster-41a
EXPNO                  23
PROCNO                  1

F2 - Acquisition Parameters
Date_            20210501
Time              12.53 h
INSTRUM             spect
PROBHD    Z112726_0001 (
PULPROG         roesyph.2
TD                   2048
SOLVENT           Acetone
NS                     16
DS                     16
SWH              6465.517 Hz
FIDRES           6.313982 Hz
AQ              0.1583787 sec
RG                      2
DW                 77.333 usec
DE                   6.50 usec
TE                  303.0 K
D0             0.00006528 sec
D1             2.00000000 sec
D12            0.00002000 sec
IN0            0.00015460 sec
L4                    877
P15             270000.00 usec
TDav                    1
SFO1          700.0030515 MHz
NUC1                   1H
P1                  12.60 usec
P25                154.00 usec
PLW1          39.81100082 W
PLW27          1.06599998 W

F1 - Acquisition parameters
TD                    183
SFO1             700.0033 MHz
FIDRES          70.691864 Hz
SW                  9.240 ppm
FnMODE               TPPI

F2 - Processing parameters
SI                   2048
SF            700.0000074 MHz
WDW                  SINE
SSB                     2
LB         0 Hz
GB         0
PC                   4.00

F1 - Processing parameters
SI                    512
MC2                  TPPI
SF            700.0002528 MHz
WDW                  SINE
SSB                     2
LB         0 Hz
GB         0
```

Figure S50. $^{1}$H NMR spectrum (700 MHz, acetone-$d_6$) of **8**

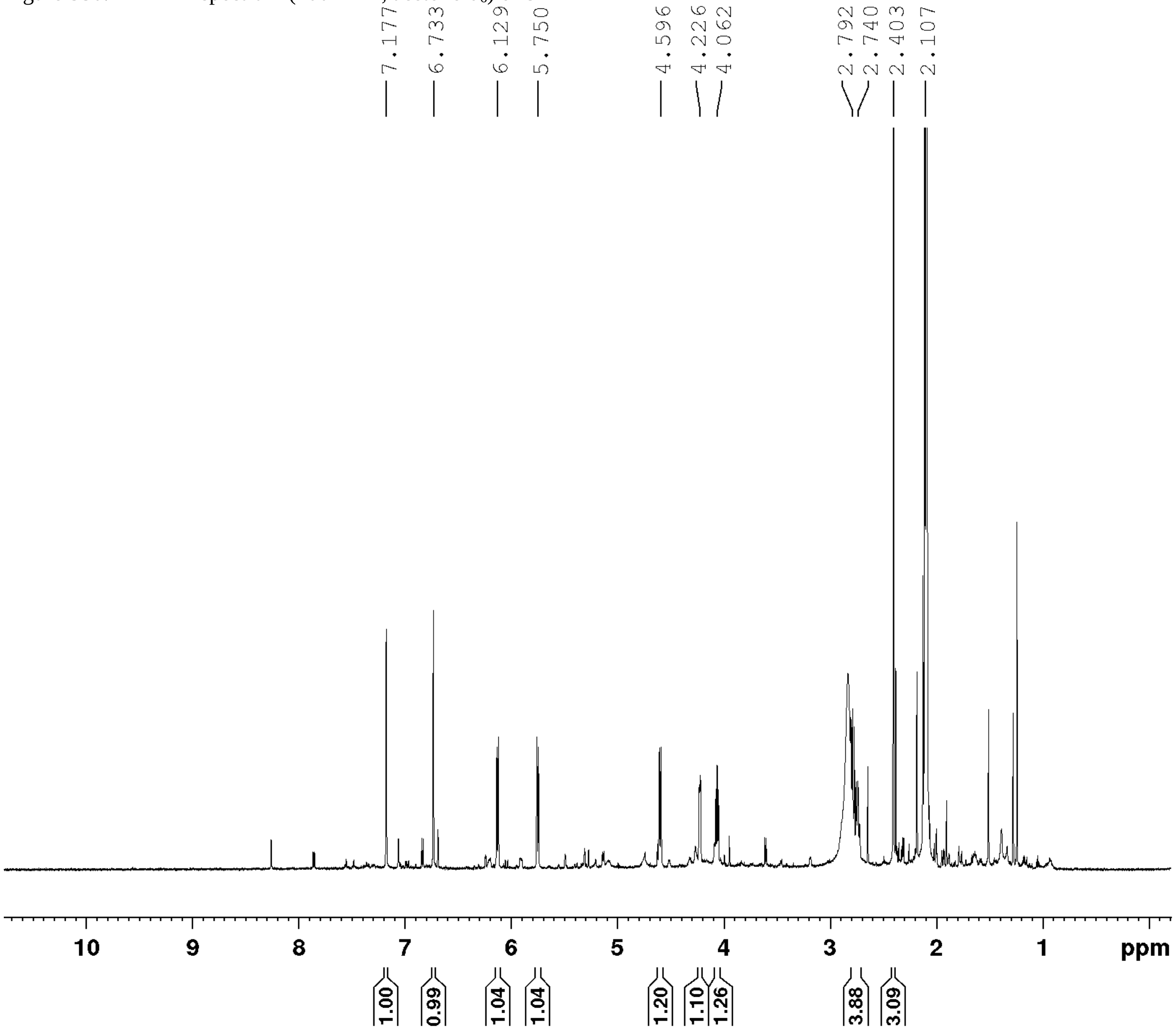


```
Current Data Parameters
NAME              Aster-3
EXPNO                   1
PROCNO                  1

F2 - Acquisition Parameters
Date_            20210211
Time                16.27 h
INSTRUM             spect
PROBHD   Z112726_0001 (
PULPROG              zg30
TD                  32768
SOLVENT           Acetone
NS                      8
DS                      2
SWH             11160.714 Hz
FIDRES           0.681196 Hz
AQ              1.4680064 sec
RG                    203
DW                 44.800 usec
DE                   6.50 usec
TE                  303.1 K
D1             2.00000000 sec
TD0                     1
SFO1          700.0053900 MHz
NUC1                   1H
P0                   4.20 usec
P1                  12.60 usec
PLW1          39.81100082 W

F2 - Processing parameters
SI                  32768
SF            699.9999836 MHz
WDW                    EM
SSB      0
LB                   0.30 Hz
GB       0
PC                   1.00
```

Figure S51. $^{13}C$ NMR spectrum (176.04 MHz, acetone-d$_6$) of **8**

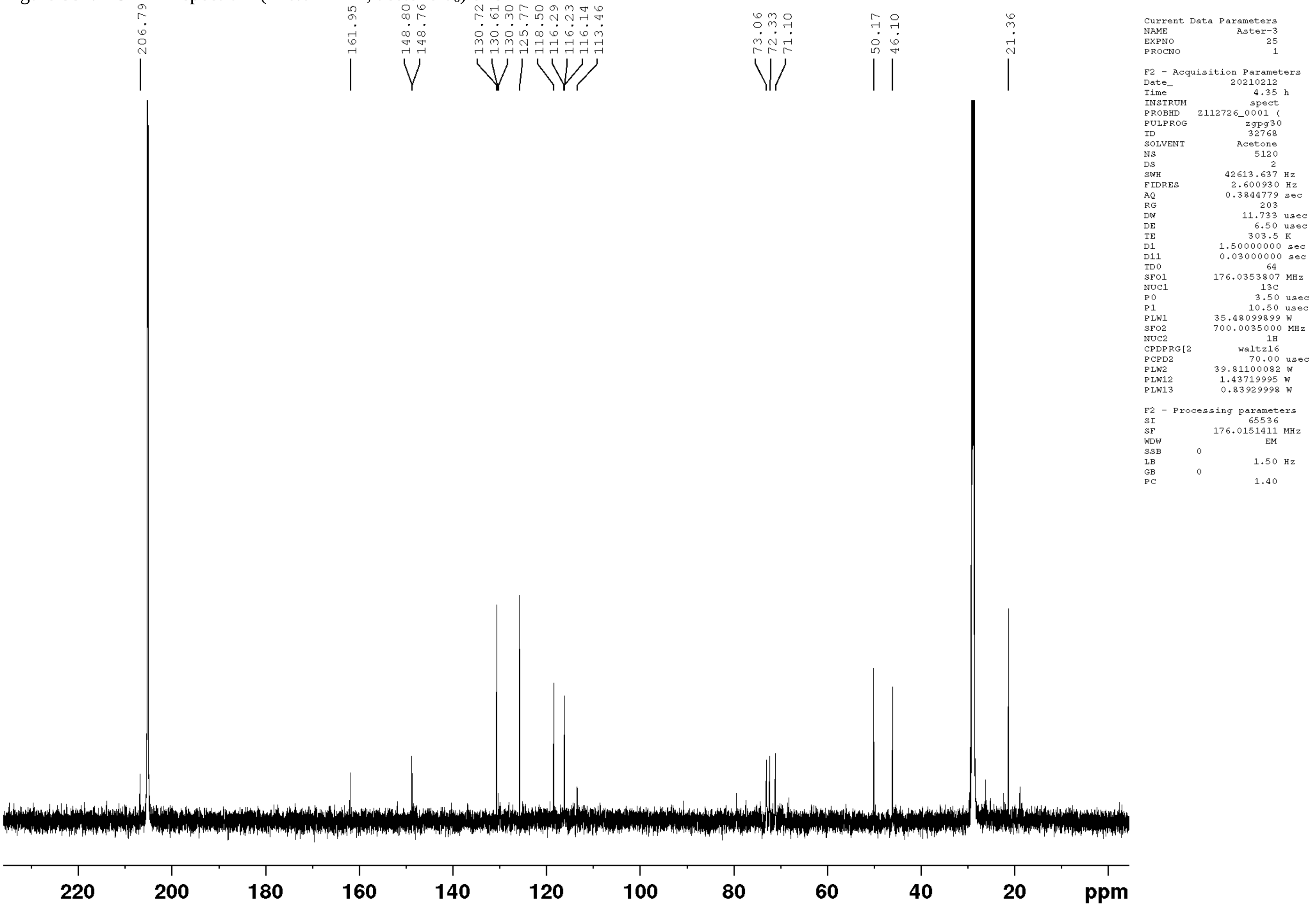

Figure S52. DEPT-135 spectrum (176.04 MHz, acetone-$d_6$) of **8**

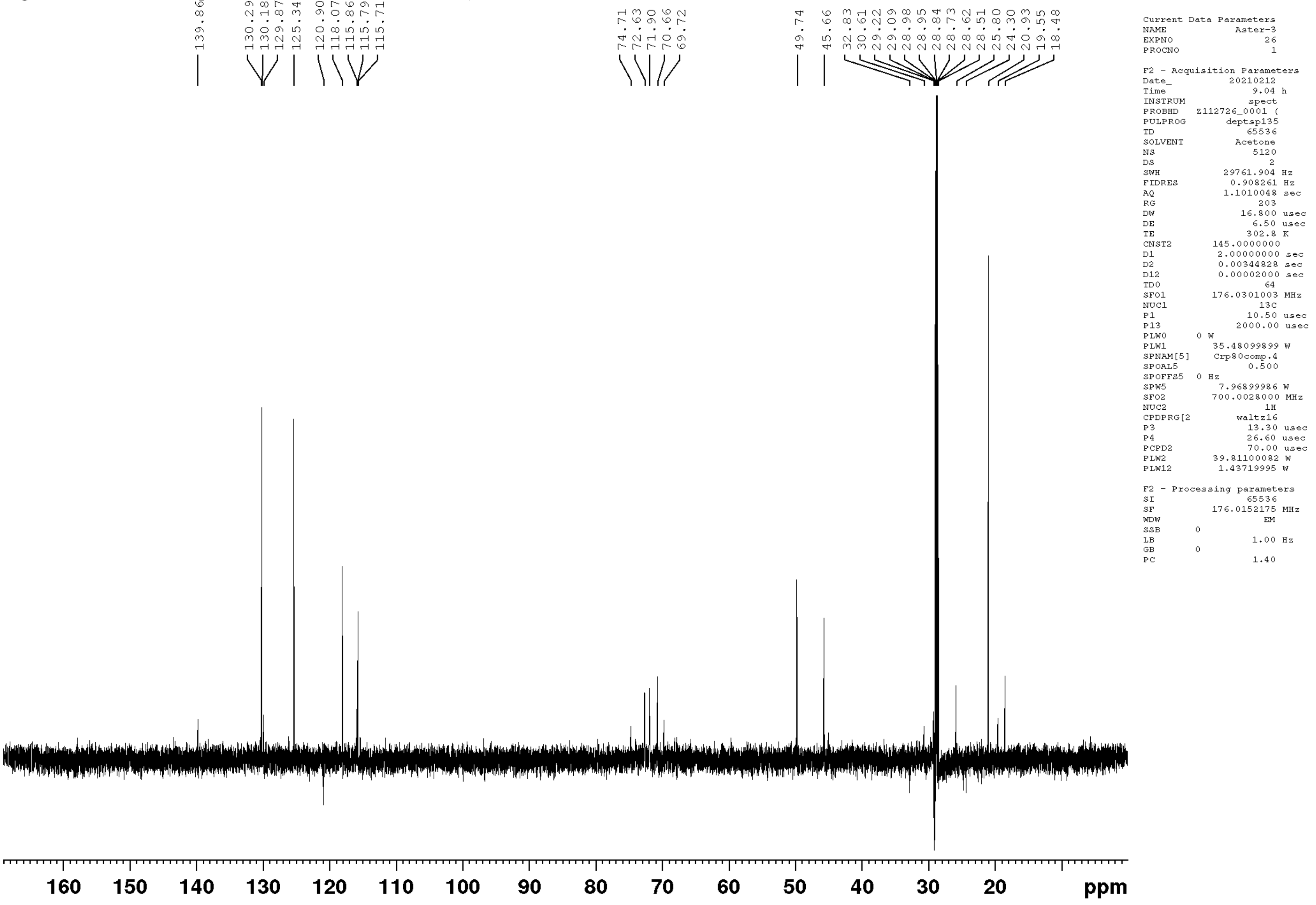

Figure S53. COSY-45 spectrum (700 MHz, acetone-$d_6$) of **8**

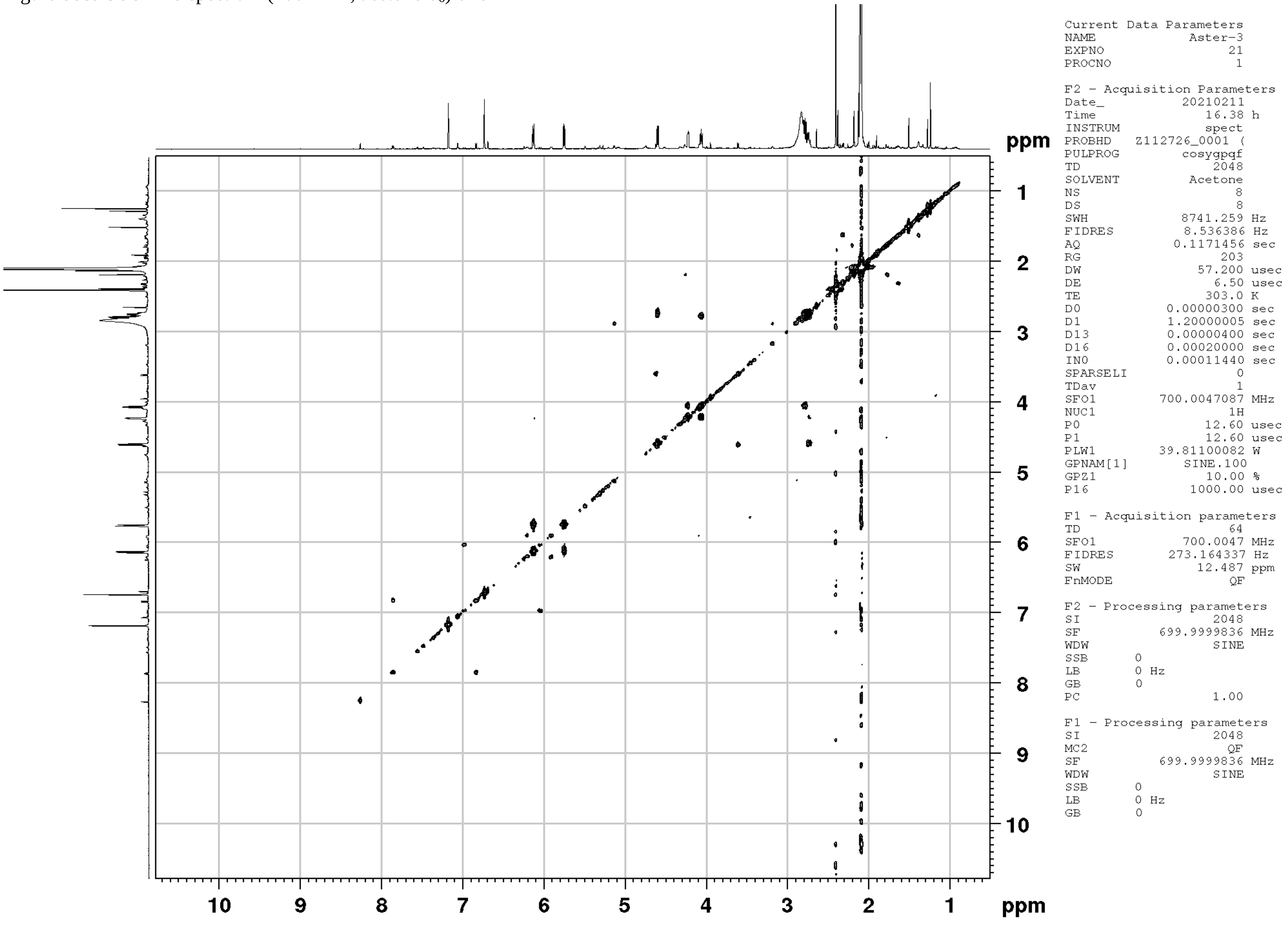

Figure S54. HSQC spectrum (700 MHz, acetone-$d_6$) of **8**

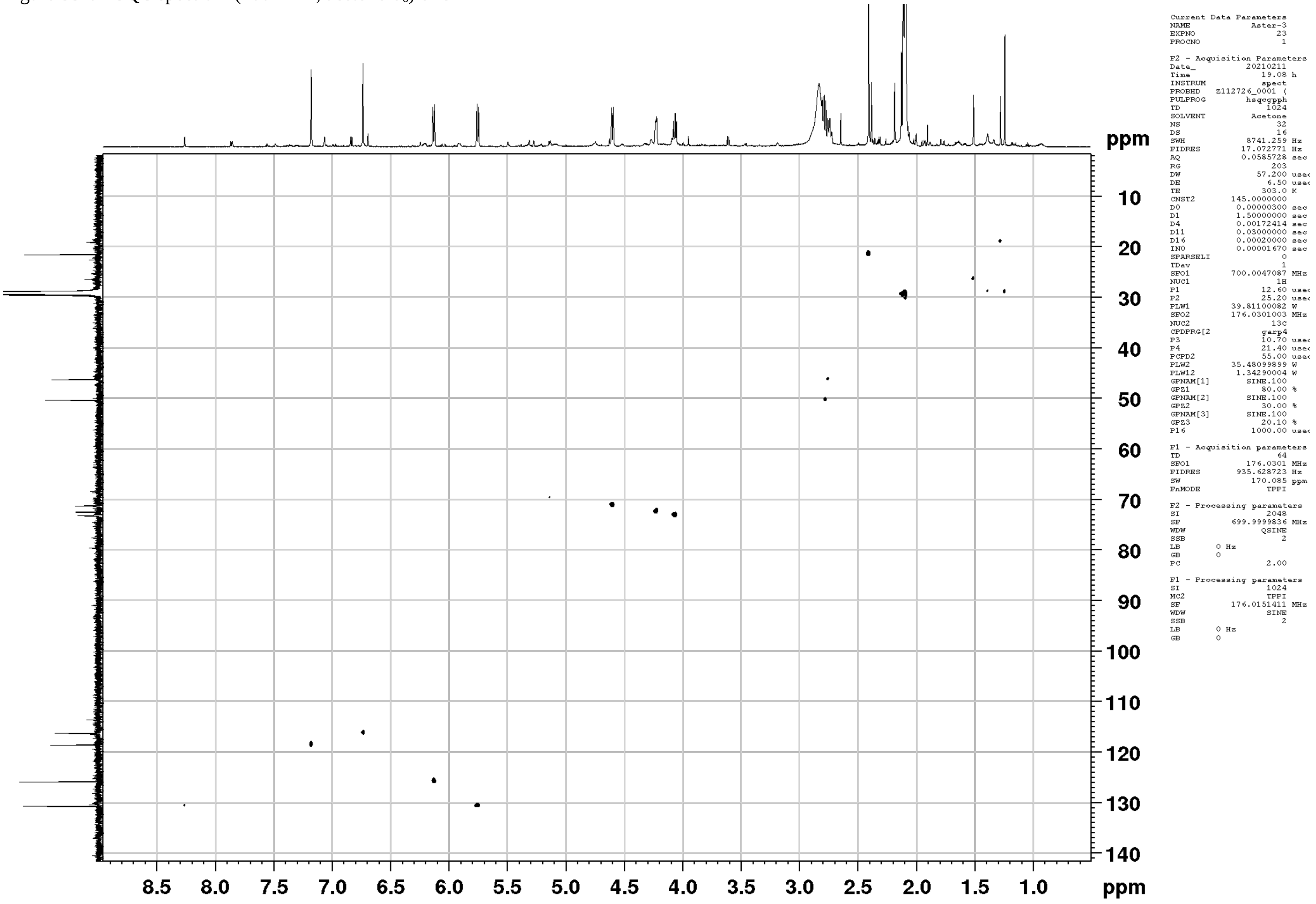

Figure S55. HMBC spectrum (700 MHz, acetone-$d_6$) of **8**

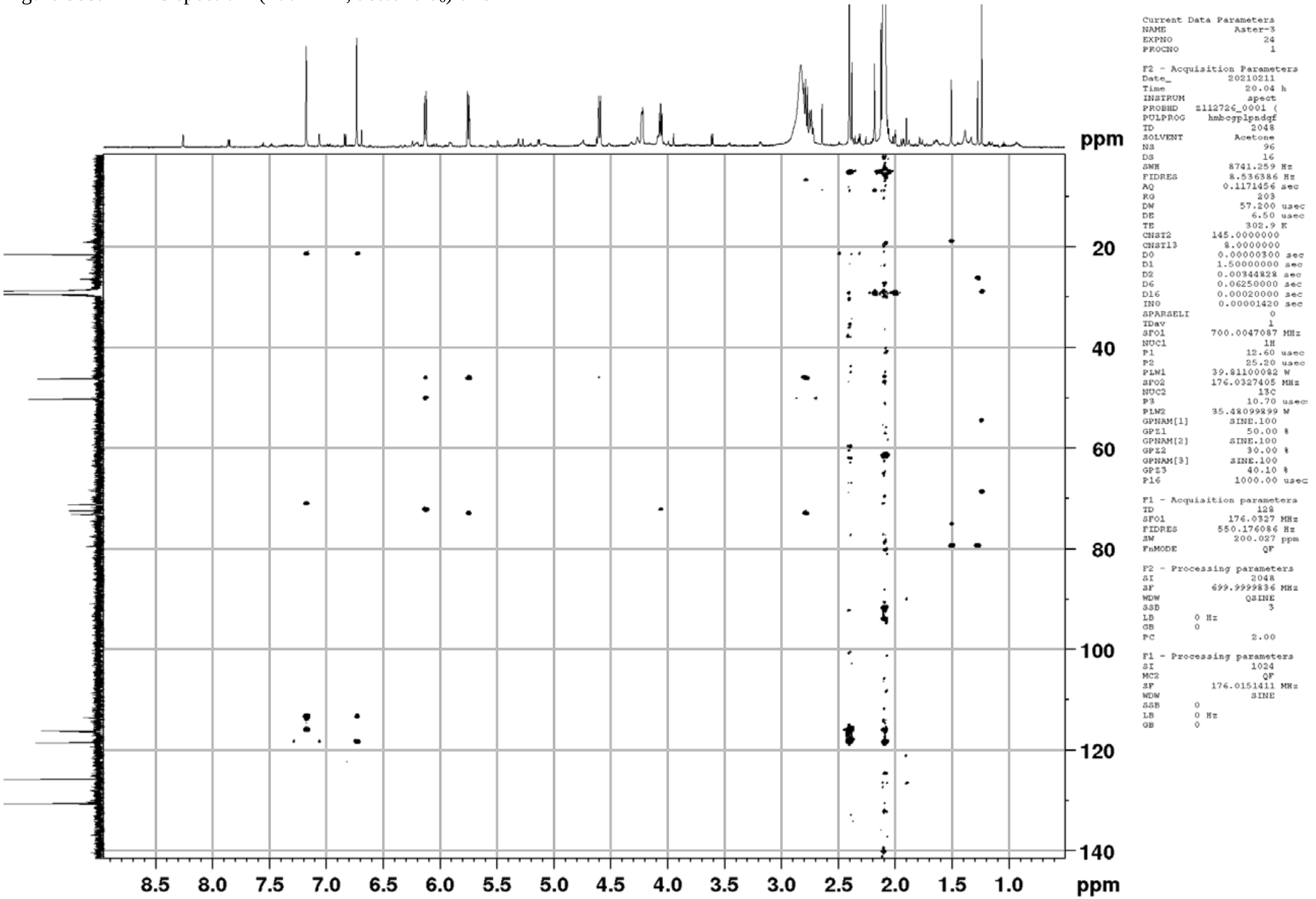

Figure S56. ROESY spectrum (700 MHz, acetone-$d_6$) of **8**

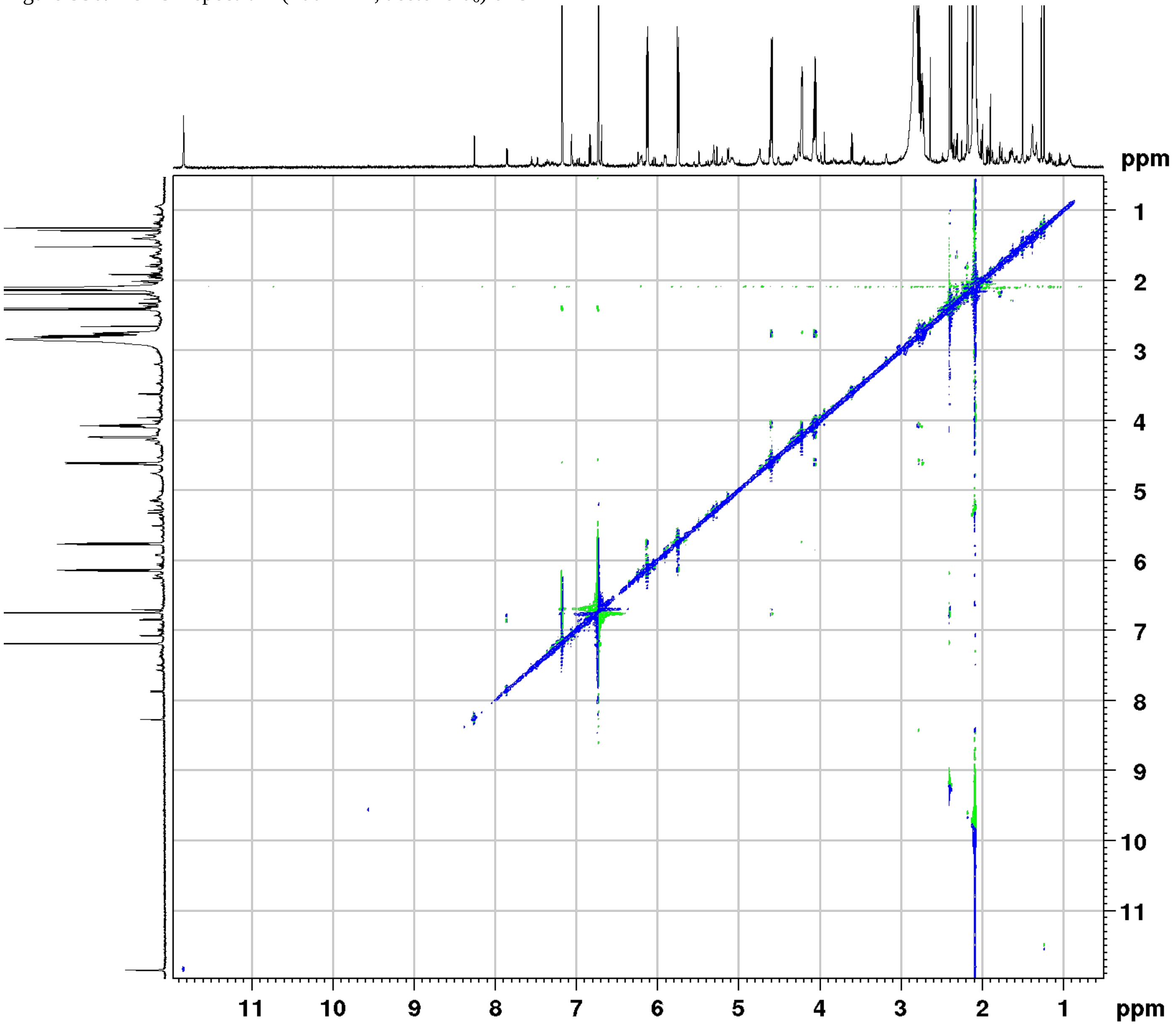


```
Current Data Parameters
NAME              Aster-3
EXPNO                  22
PROCNO                  1

F2 - Acquisition Parameters
Date_            20210211
Time                17.02 h
INSTRUM             spect
PROBHD   Z112726_0001 (
PULPROG         roesyph.2
TD                   2048
SOLVENT           Acetone
NS                     16
DS                     16
SWH              8741.259 Hz
FIDRES           8.536386 Hz
AQ              0.1171456 sec
RG                      4
DW                 57.200 usec
DE                   6.50 usec
TE                  303.1 K
D0             0.00004518 sec
D1             1.50000000 sec
D12            0.00002000 sec
IN0            0.00011440 sec
L4                    649
P15             200000.00 usec
TDav                    1
SFO1          700.0047087 MHz
NUC1                   1H
P1                  12.60 usec
P25                154.00 usec
PLW1          39.81100082 W
PLW27          1.06599998 W

F1 - Acquisition parameters
TD                    256
SFO1             700.0047 MHz
FIDRES          68.291084 Hz
SW                 12.487 ppm
FnMODE               TPPI

F2 - Processing parameters
SI                   2048
SF            699.9999836 MHz
WDW                  SINE
SSB                     2
LB          0 Hz
GB          0
PC                   2.00

F1 - Processing parameters
SI                   1024
MC2                  TPPI
SF            699.9999836 MHz
WDW                  SINE
SSB                     2
LB          0 Hz
GB          0
```

Figure S57. $^{1}H$ NMR spectrum (500 MHz, acetone-$d_6$) of **9**

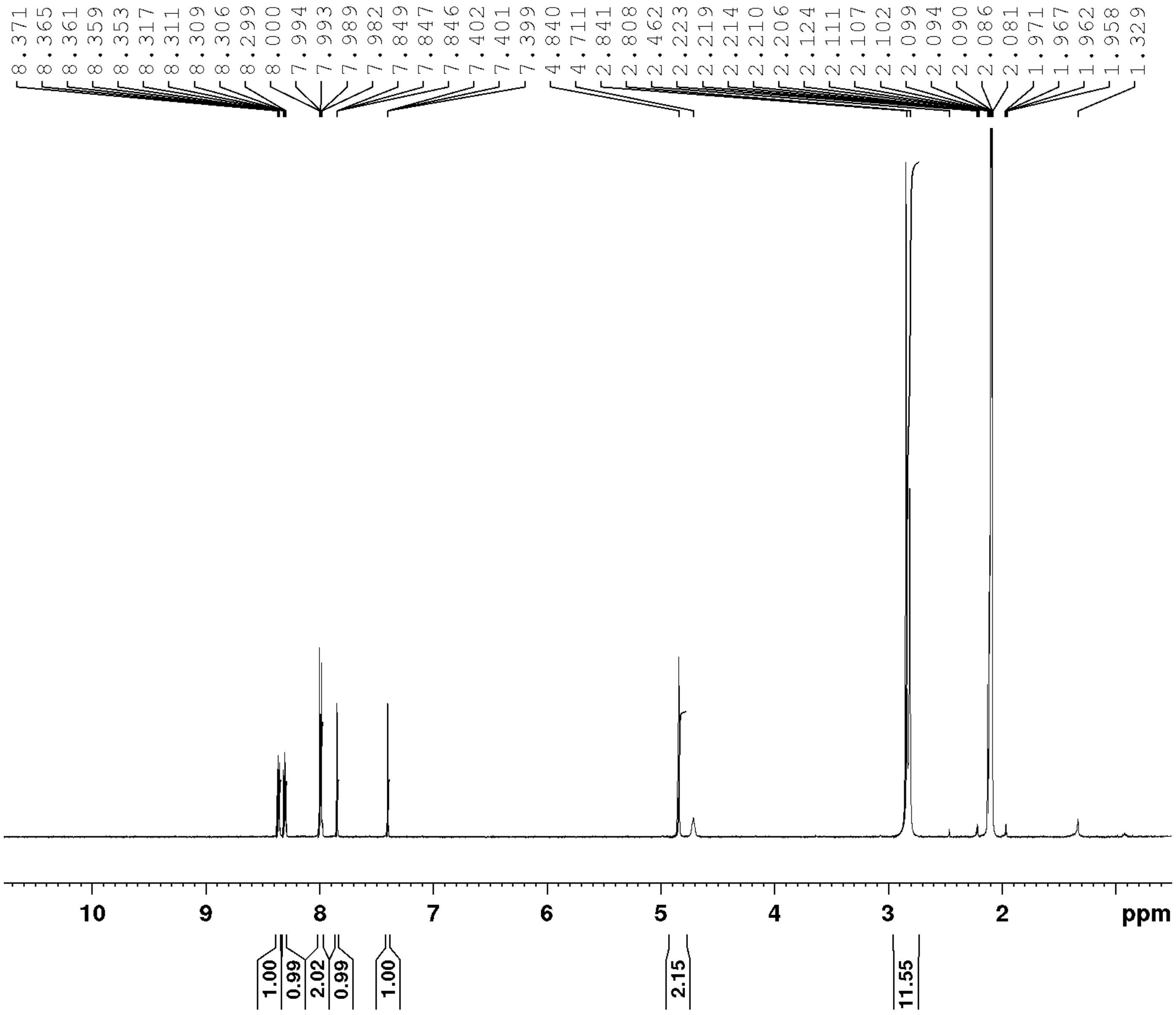


Current Data Parameters
NAME Aster-12
EXPNO 5
PROCNO 1

F2 - Acquisition Parameters
Date_ 20210218
Time 15.12 h
INSTRUM spect
PROBHD Z113652_0155 (
PULPROG zg30
TD 32768
SOLVENT Acetone
NS 1
DS 2
SWH 6493.506 Hz
FIDRES 0.396332 Hz
AQ 2.5231359 sec
RG 119.65
DW 77.000 usec
DE 6.50 usec
TE 303.2 K
D1 2.00000000 sec
TD0 1
SFO1 500.1334933 MHz
NUC1 1H
P0 3.88 usec
P1 11.65 usec
PLW1 15.84899998 W

F2 - Processing parameters
SI 65536
SF 500.1299935 MHz
WDW EM
SSB 0
LB 0.30 Hz
GB 0
PC 1.00

Figure S58. $^{13}C$ NMR spectrum (125.77 MHz, acetone-$d_6$) of **9**

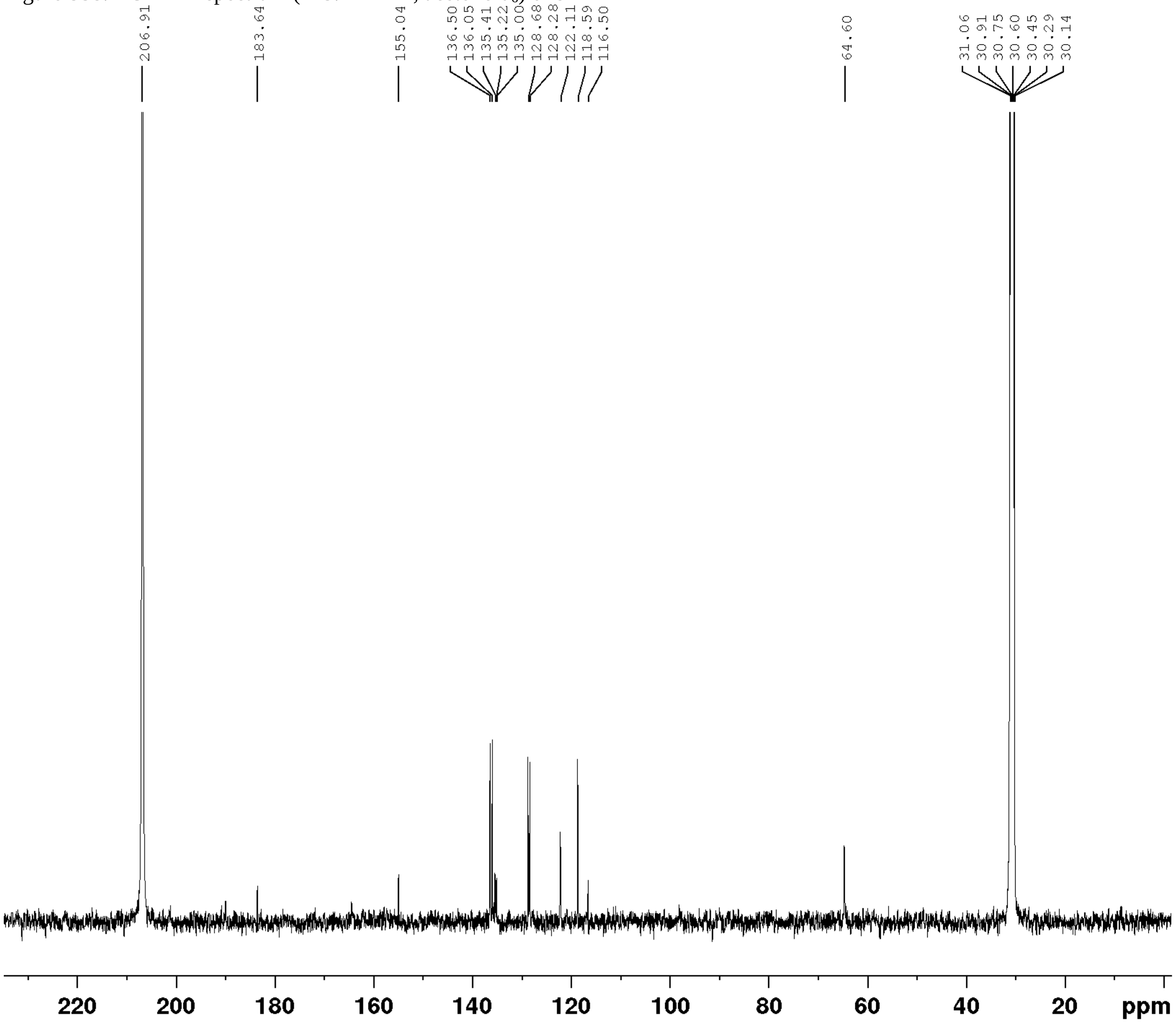


Current Data Parameters
NAME Aster-12
EXPNO 25
PROCNO 1

F2 - Acquisition Parameters
Date_ 20210219
Time 7.35 h
INSTRUM spect
PROBHD Z113652_0155 (
PULPROG zgpg30
TD 32768
SOLVENT Acetone
NS 2560
DS 4
SWH 29761.904 Hz
FIDRES 1.816522 Hz
AQ 0.5505024 sec
RG 196.84
DW 16.800 usec
DE 6.50 usec
TE 303.1 K
D1 1.50000000 sec
D11 0.03000000 sec
TD0 32
SFO1 125.7722511 MHz
NUC1 13C
P0 4.03 usec
P1 12.10 usec
PLW1 79.43299866 W
SFO2 500.1320005 MHz
NUC2 1H
CPDPRG[2 waltz16
PCPD2 80.00 usec
PLW2 15.84899998 W
PLW12 0.33610001 W
PLW13 0.16906001 W

F2 - Processing parameters
SI 65536
SF 125.7575777 MHz
WDW EM
SSB 0
LB 5.00 Hz
GB 0
PC 1.30

Figure S59. DEPT-135 spectrum (125.77 MHz, acetone-$d_6$) of **9**

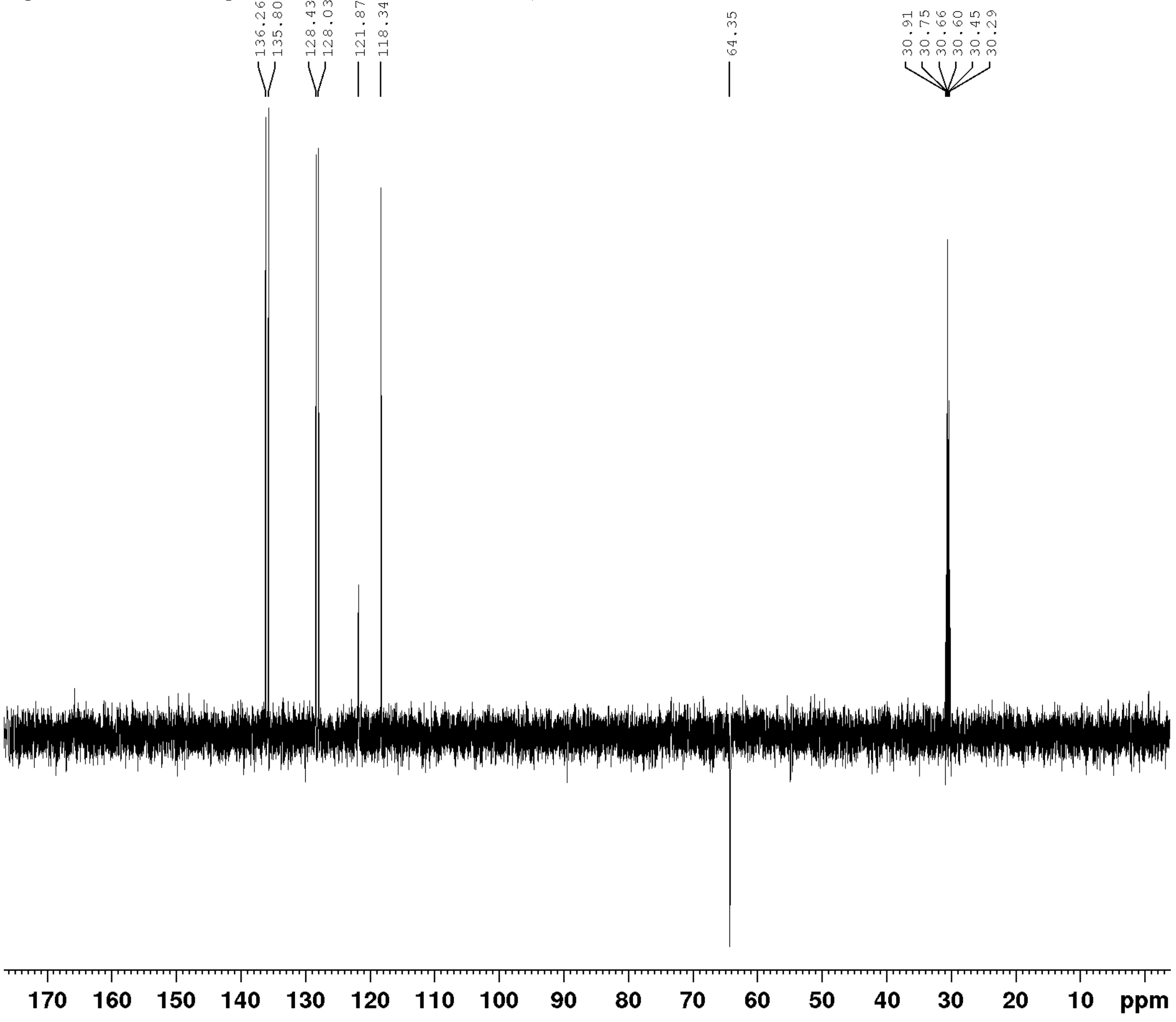


```
Current Data Parameters
NAME            Aster-12
EXPNO                 26
PROCNO                 1

F2 - Acquisition Parameters
Date_           20210219
Time               10.01 h
INSTRUM            spect
PROBHD   Z113652_0155 (
PULPROG        deptsp135
TD                 32768
SOLVENT          Acetone
NS                  3880
DS                     2
SWH            22727.273 Hz
FIDRES          1.387163 Hz
AQ             0.7208960 sec
RG                196.84
DW                22.000 usec
DE                  6.50 usec
TE                 303.2 K
CNST2        145.0000000
D1            1.50000000 sec
D2            0.00344828 sec
D12           0.00002000 sec
TD0                 4096
SFO1         125.7684784 MHz
NUC1                 13C
P1                 12.10 usec
P13              2000.00 usec
PLW0      0 W
PLW1         79.43299866 W
SPNAM[5]     Crp60comp.4
SPOAL5             0.500
SPOFFS5   0 Hz
SPW5         17.76899910 W
SFO2         500.1320005 MHz
NUC2                  1H
CPDPRG[2         waltz16
P3                 11.65 usec
P4                 23.30 usec
PCPD2              80.00 usec
PLW2         15.84899998 W
PLW12         0.33610001 W

F2 - Processing parameters
SI                 65536
SF           125.7576090 MHz
WDW                   EM
SSB       0
LB                  1.00 Hz
GB        0
PC                  1.40
```

Figure S60. COSY-45 spectrum (500 MHz, acetone-$d_6$) of **9**

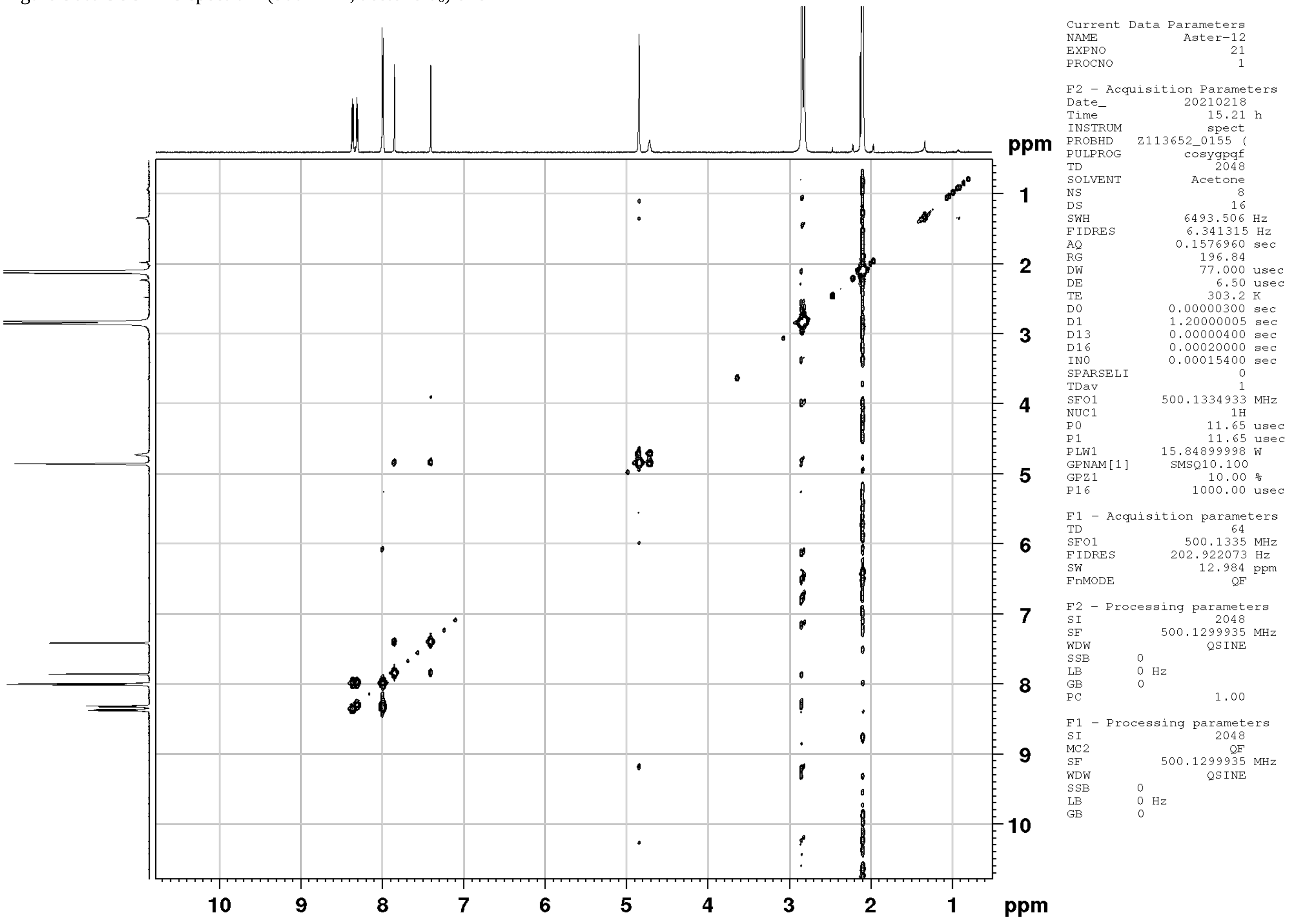

Figure S61. HSQC spectrum (500 MHz, acetone-$d_6$) of **9**

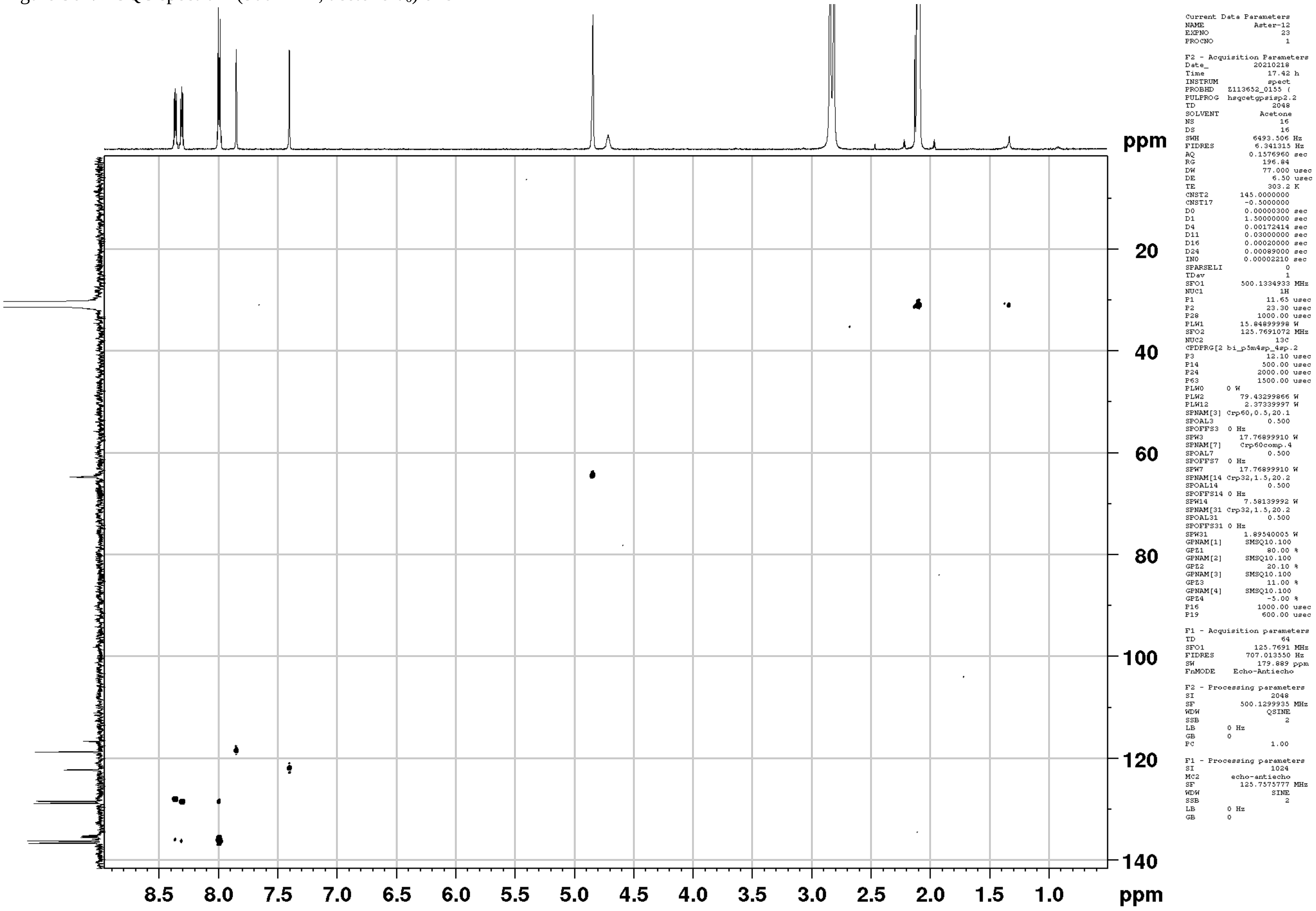

Figure S62. HMBC spectrum (500 MHz, acetone-$d_6$) of **9**

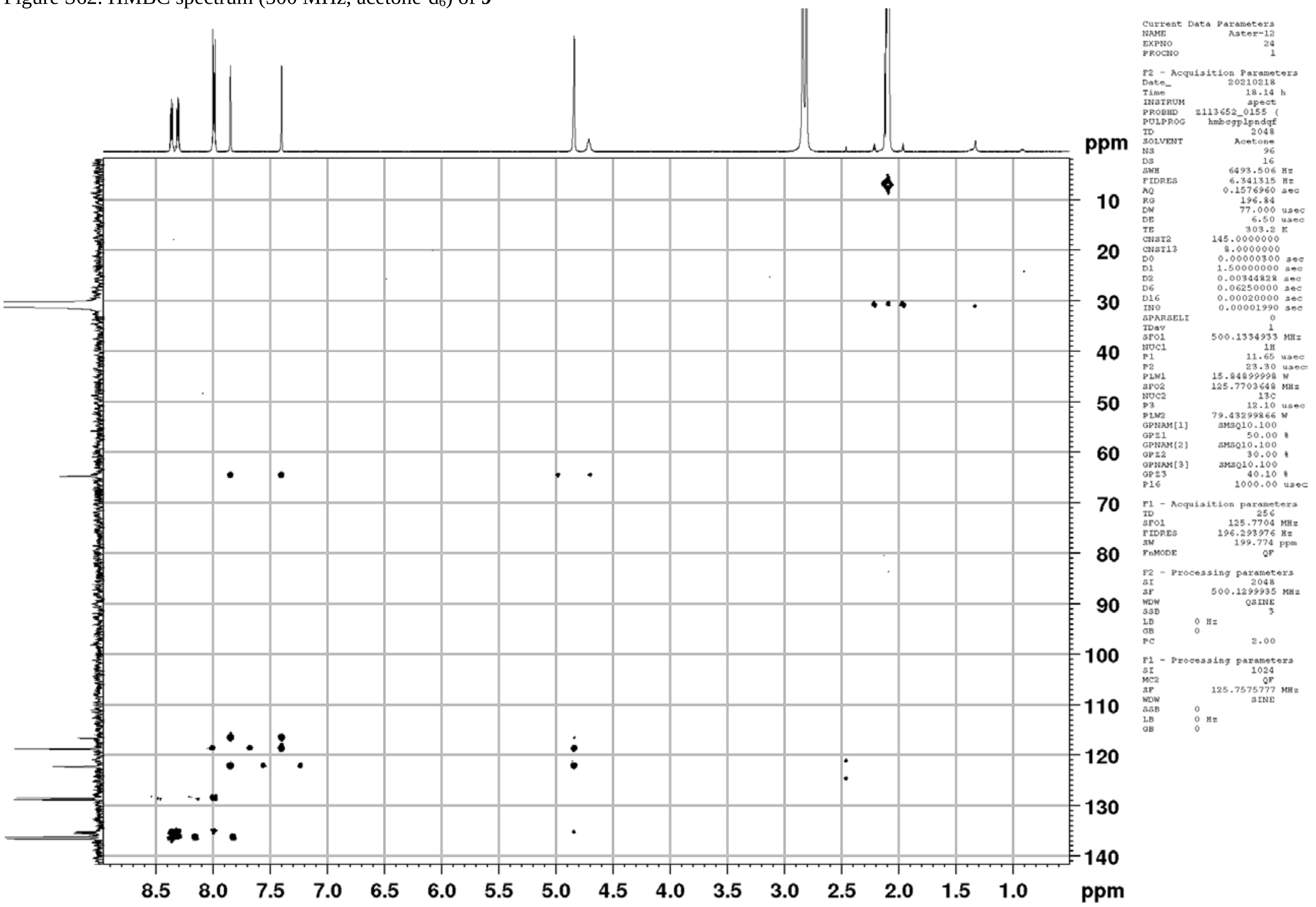

Figure S63. ROESY spectrum (500 MHz, acetone-$d_6$) of **9**

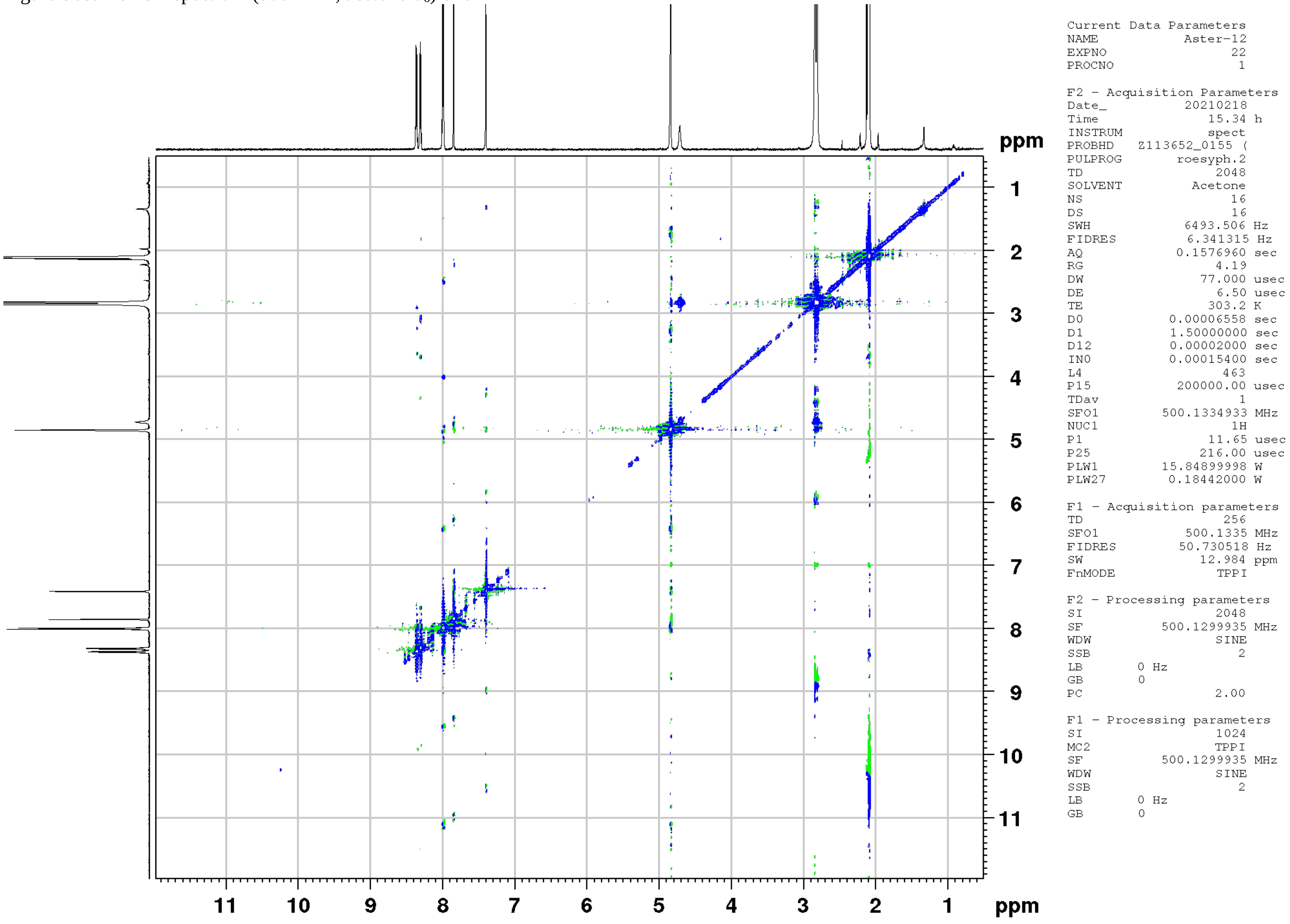

Figure S64. $^{1}H$ NMR spectrum (500 MHz, acetone-$d_6$) of **10**

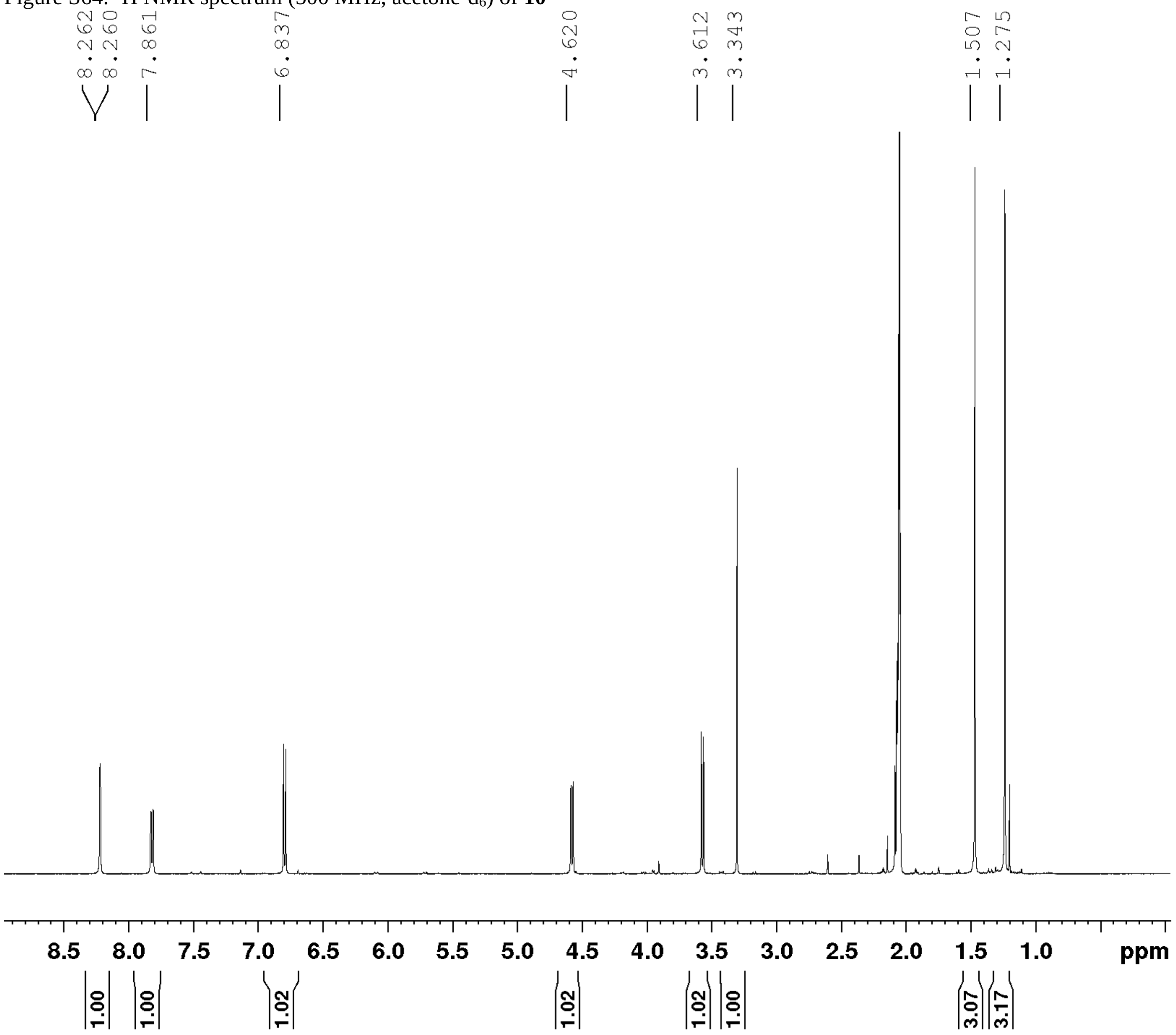


```
Current Data Parameters
NAME                Aster-4
EXPNO                     5
PROCNO                    1

F2 - Acquisition Parameters
Date_              20210129
Time                12.57 h
INSTRUM               spect
PROBHD   Z113652_0155 (
PULPROG                zg30
TD                    32768
SOLVENT             Acetone
NS                        1
DS                        2
SWH               4504.504 Hz
FIDRES            0.274933 Hz
AQ               3.6372480 sec
RG                   119.65
DW                  111.000 usec
DE                     6.50 usec
TE                    303.1 K
D1               2.00000000 sec
TD0                       1
SFO1            500.1322447 MHz
NUC1                     1H
P0                     3.88 usec
P1                    11.65 usec
PLW1            15.84899998 W

F2 - Processing parameters
SI                    65536
SF              500.1300134 MHz
WDW                      EM
SSB      0
LB                     0.30 Hz
GB       0
PC                     1.00
```

Figure S65. $^{13}C$ NMR spectrum (125.77 MHz, acetone-$d_6$) of **10**

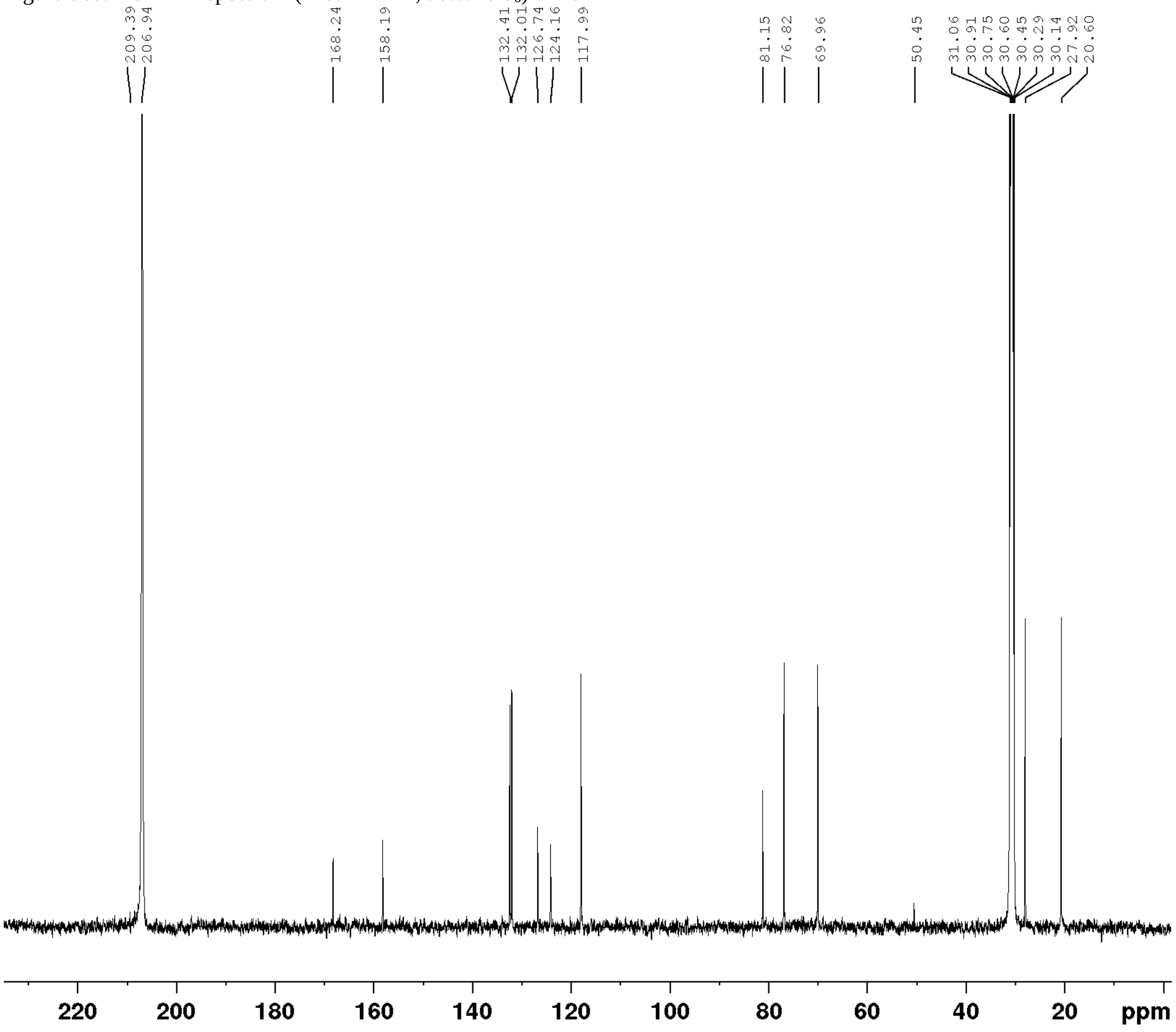


```
Current Data Parameters
NAME              Aster-4
EXPNO                  12
PROCNO                  1

F2 - Acquisition Parameters
Date_            20210128
Time                15.05 h
INSTRUM             spect
PROBHD   Z113652_0155 (
PULPROG            zgpg30
TD                  32768
SOLVENT           Acetone
NS                   1650
DS                      4
SWH             29761.904 Hz
FIDRES           1.816522 Hz
AQ              0.5505024 sec
RG                 196.84
DW                 16.800 usec
DE                   6.50 usec
TE                  303.1 K
D1             1.50000000 sec
D11            0.03000000 sec
TD0                  4096
SFO1          125.7722511 MHz
NUC1                  13C
P0                   4.03 usec
P1                  12.10 usec
PLW1          79.43299866 W
SFO2          500.1320005 MHz
NUC2                   1H
CPDPRG[2          waltz16
PCPD2               80.00 usec
PLW2          15.84899998 W
PLW12          0.33610001 W
PLW13          0.16906001 W

F2 - Processing parameters
SI                  65536
SF            125.7575787 MHz
WDW                    EM
SSB      0
LB                   5.00 Hz
GB       0
PC                   1.30
```

Figure S66. DEPT-135 spectrum (125.77 MHz, acetone-$d_6$) of **10**

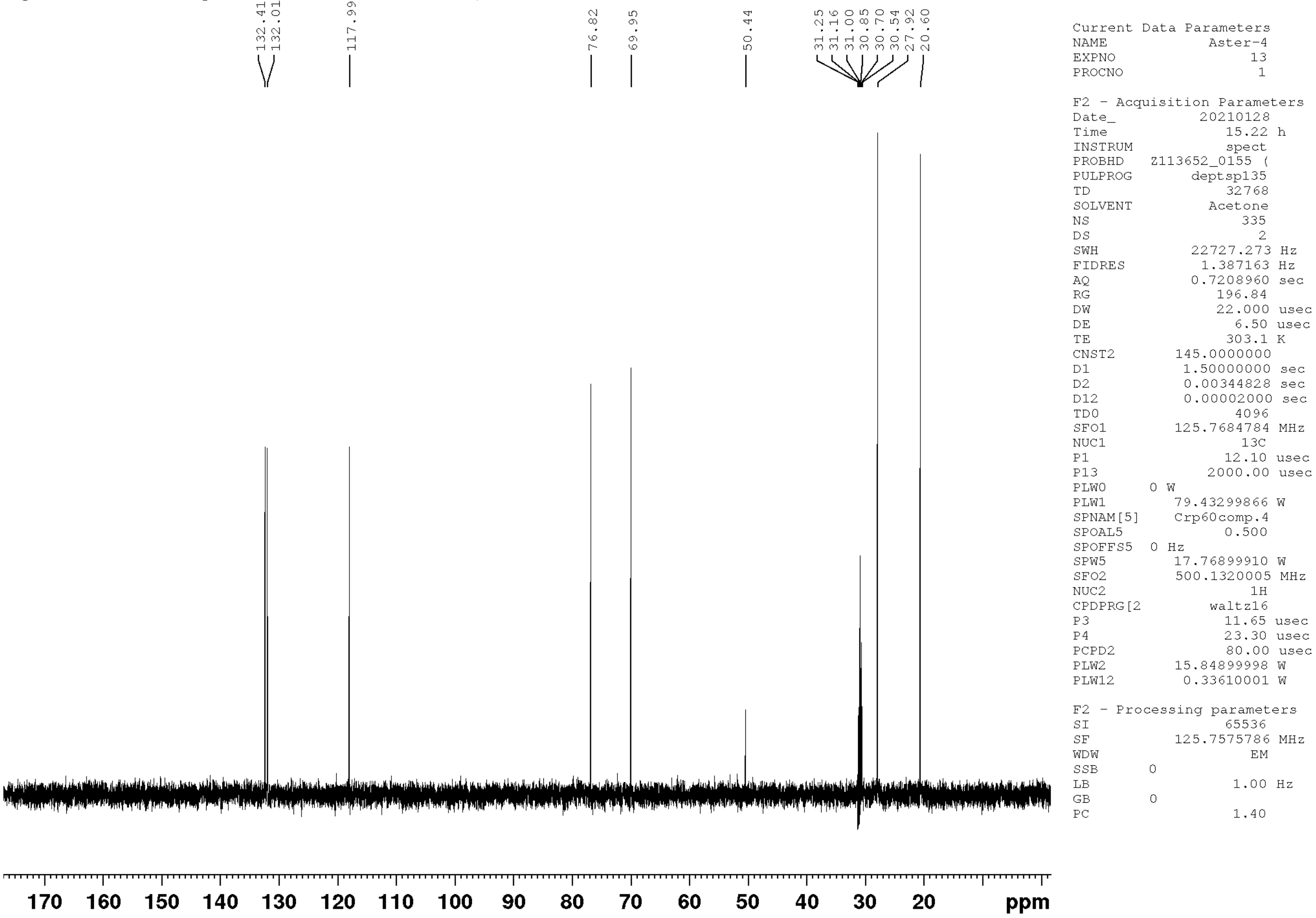

Figure S67. COSY-45 spectrum (500 MHz, acetone-$d_6$) of **10**

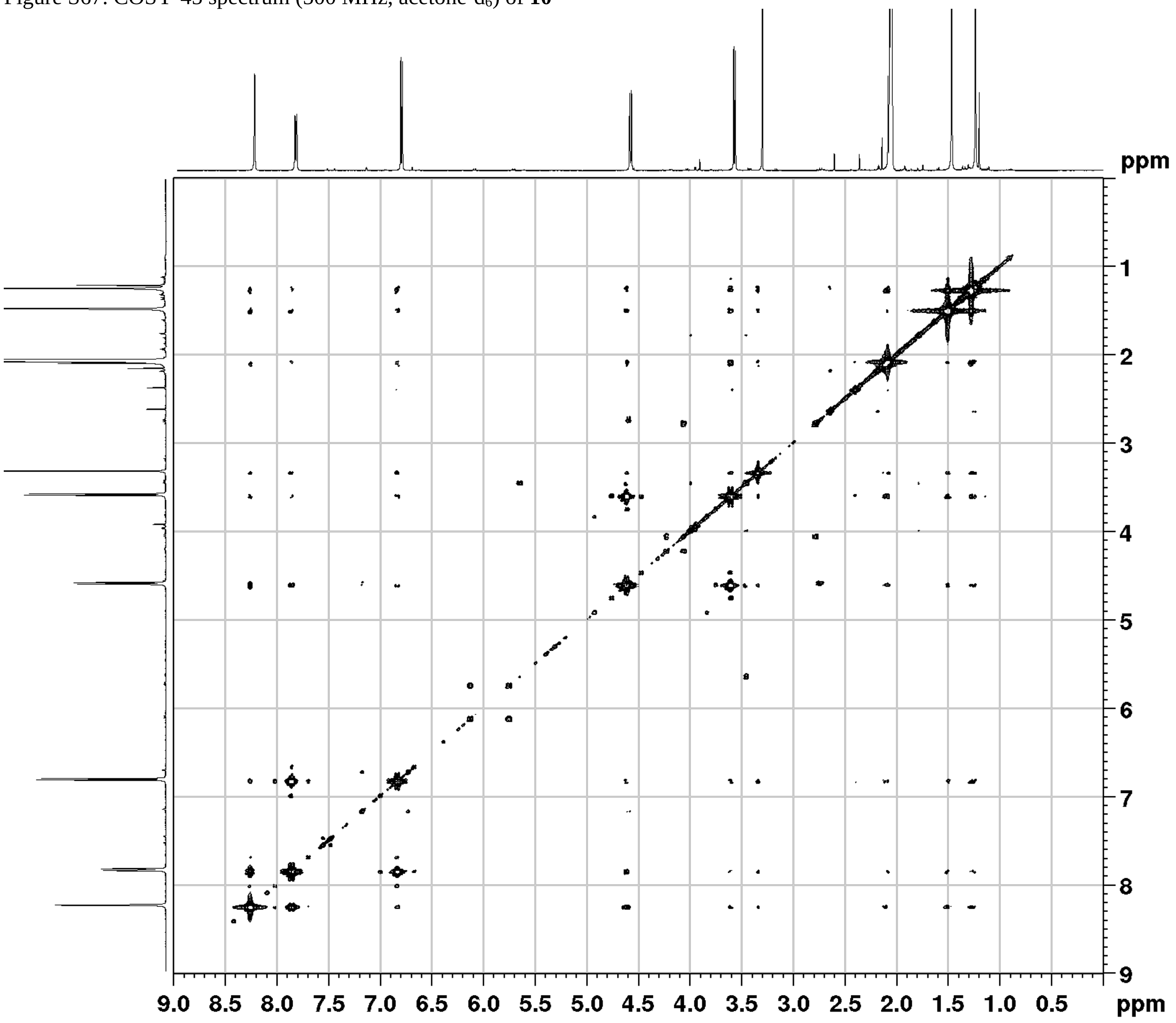


Current Data Parameters
NAME Aster-4
EXPNO 21
PROCNO 1

F2 - Acquisition Parameters
Date_ 20210129
Time 13.09 h
INSTRUM spect
PROBHD Z113652_0155 (
PULPROG cosygpqf
TD 2048
SOLVENT Acetone
NS 4
DS 16
SWH 4504.504 Hz
FIDRES 4.398930 Hz
AQ 0.2273280 sec
RG 50.73
DW 111.000 usec
DE 6.50 usec
TE 303.2 K
D0 0.00000300 sec
D1 1.50000000 sec
D13 0.00000400 sec
D16 0.00020000 sec
IN0 0.00022200 sec
SPARSELI 0
TDav 1
SFO1 500.1322447 MHz
NUC1 1H
P0 11.65 usec
P1 11.65 usec
PLW1 15.84899998 W
GPNAM[1] SMSQ10.100
GPZ1 10.00 %
P16 1000.00 usec

F1 - Acquisition parameters
TD 256
SFO1 500.1322 MHz
FIDRES 35.191441 Hz
SW 9.007 ppm
FnMODE QF

F2 - Processing parameters
SI 2048
SF 500.1299935 MHz
WDW SINE
SSB 0
LB 0 Hz
GB 0
PC 2.00

F1 - Processing parameters
SI 2048
MC2 QF
SF 500.1299935 MHz
WDW SINE
SSB 0
LB 0 Hz
GB 0

Figure S68. HSQC spectrum (500 MHz, acetone-$d_6$) of **10**

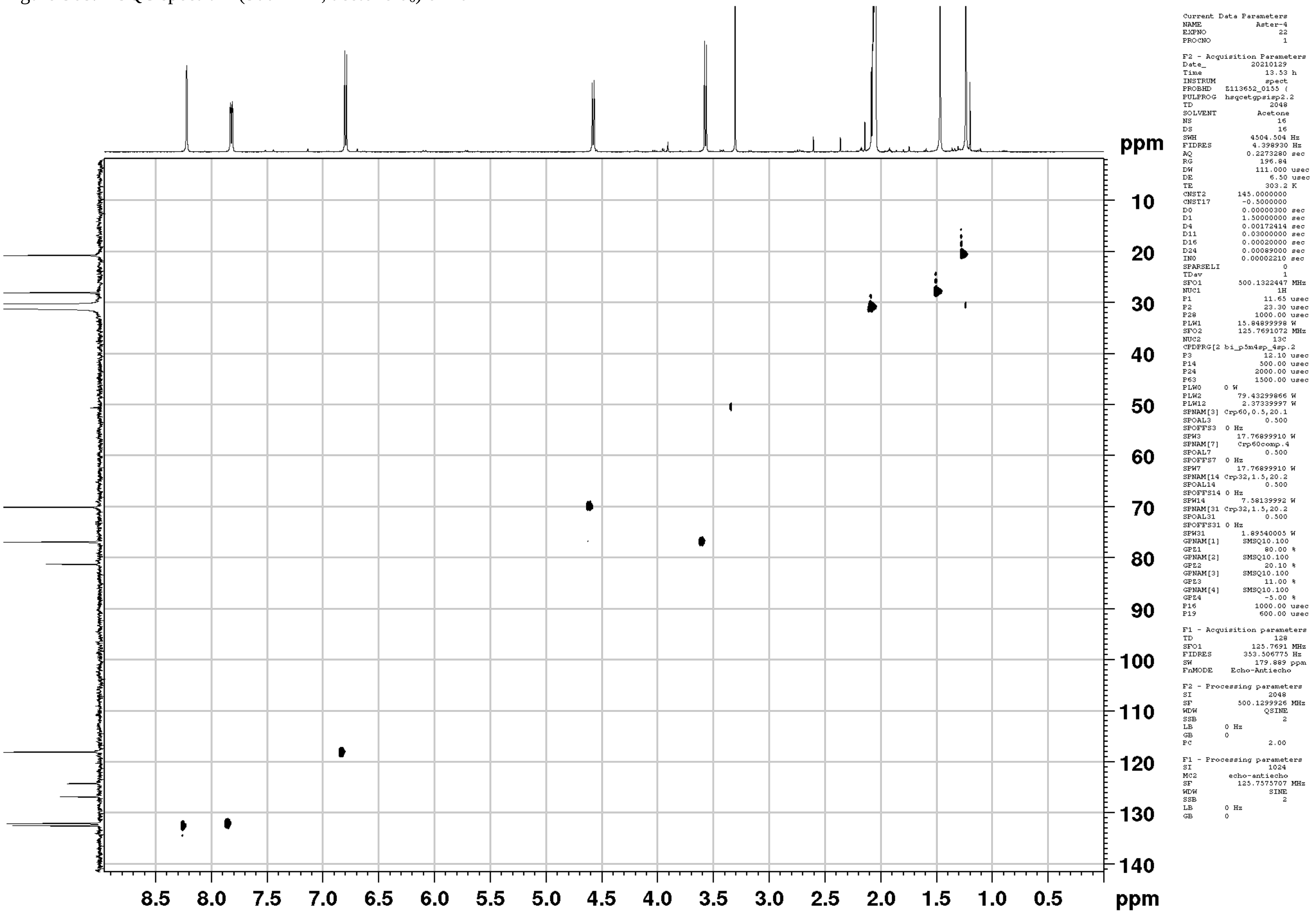

Figure S69. HMBC spectrum (500 MHz, acetone-$d_6$) of **10**

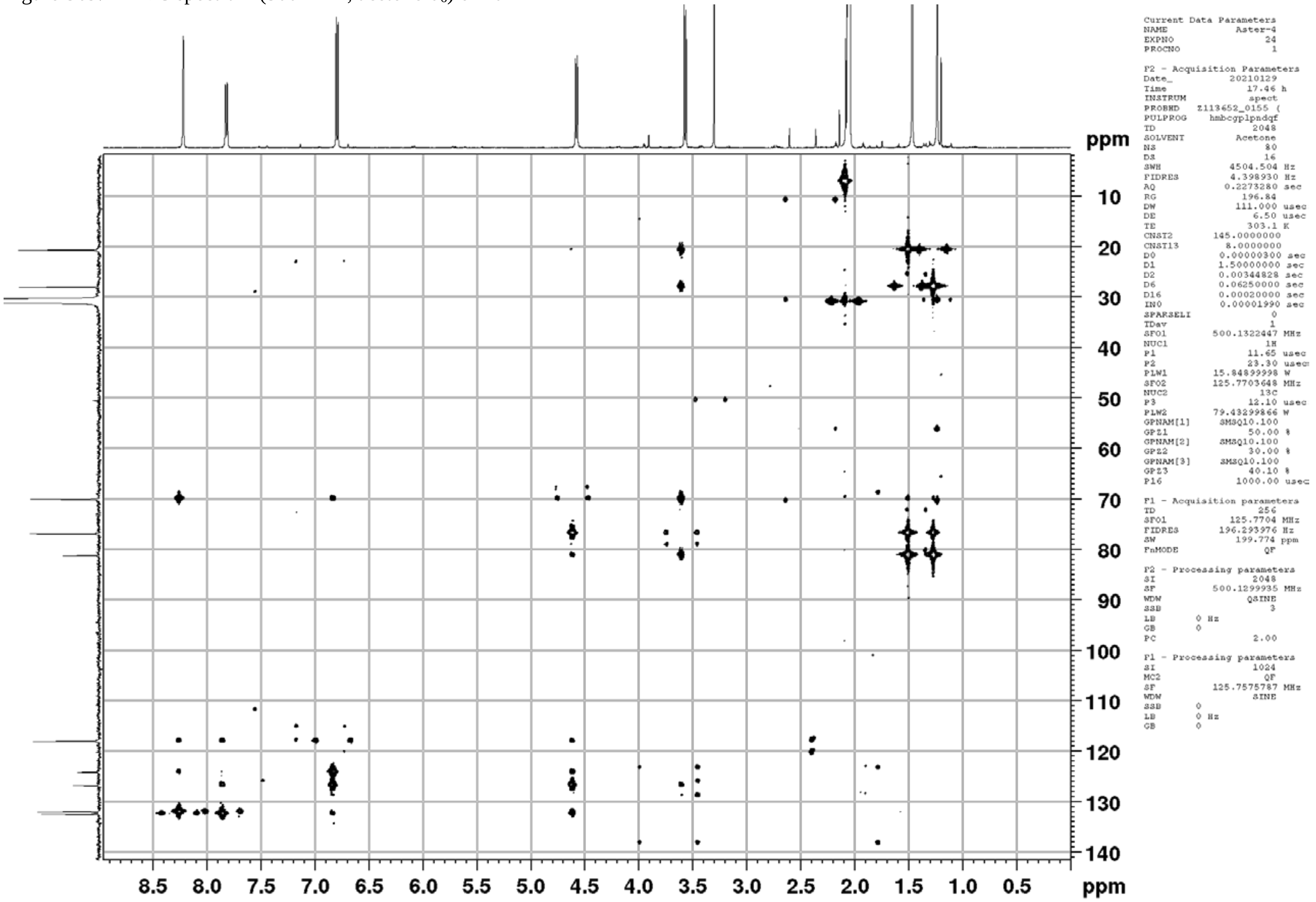

Figure S70. ROESY spectrum (500 MHz, acetone-$d_6$) of **10**

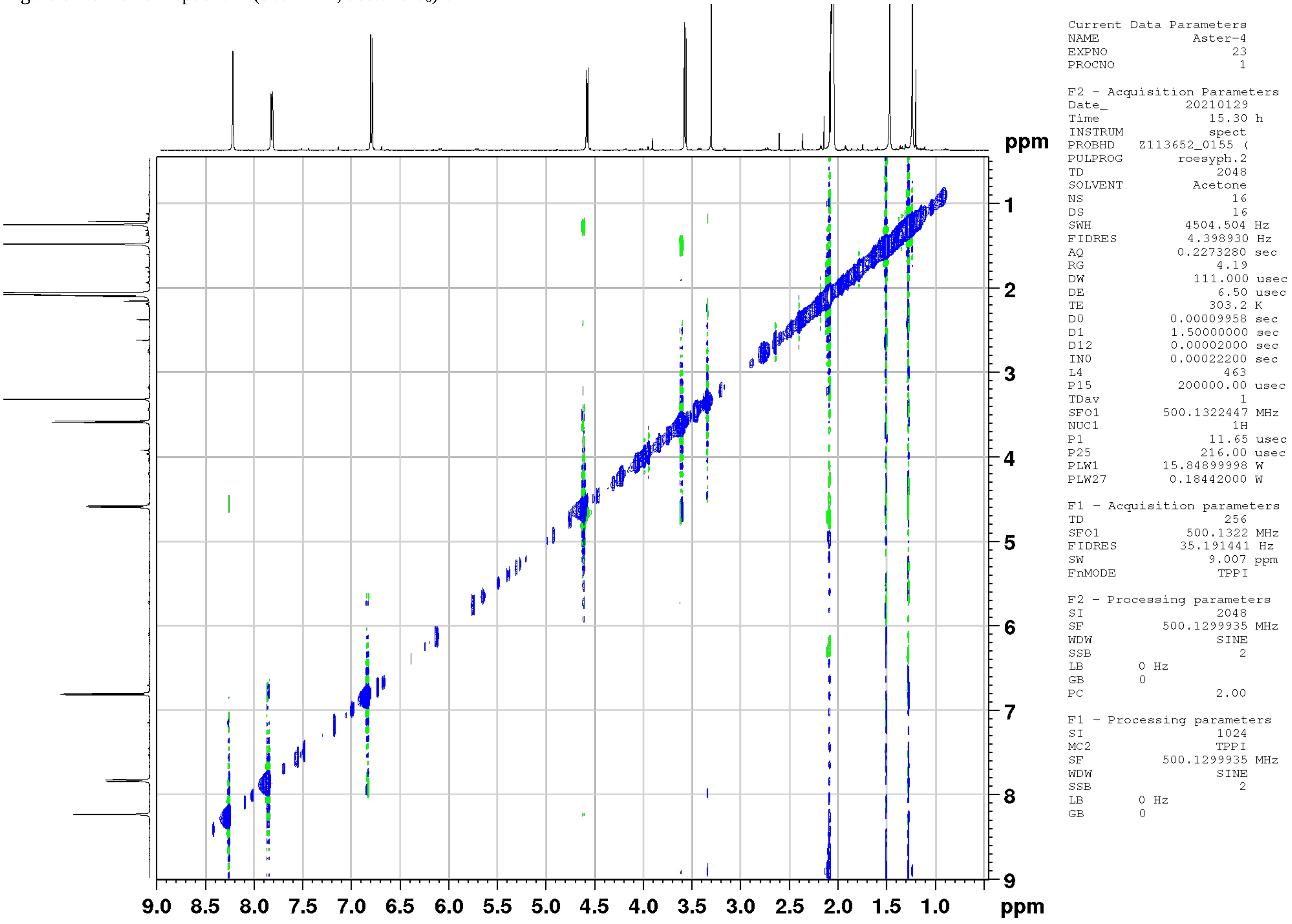

Figure S71. $^{1}$H NMR spectrum (700 MHz, acetone-$d_6$) of **11**

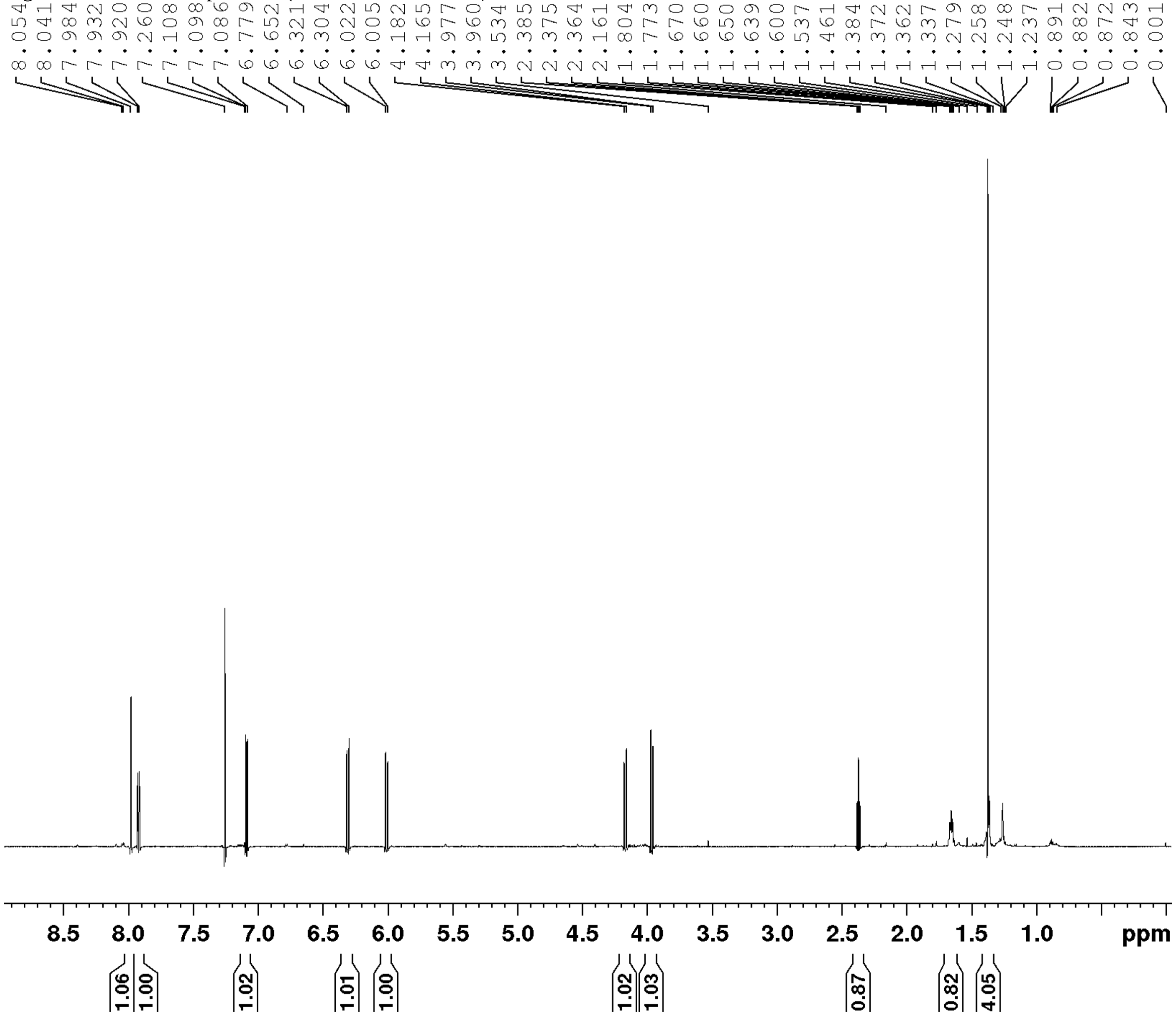


Current Data Parameters
NAME Aster-38
EXPNO 1
PROCNO 1

F2 - Acquisition Parameters
Date_ 20210501
Time 16.49 h
INSTRUM spect
PROBHD Z112726_0001 (
PULPROG zg
TD 32768
SOLVENT CDCl3
NS 1
DS 0
SWH 11160.714 Hz
FIDRES 0.681196 Hz
AQ 1.4680064 sec
RG 181
DW 44.800 usec
DE 6.50 usec
TE 303.0 K
D1 0 sec
TD0 1
SFO1 700.0053900 MHz
NUC1 1H
P1 12.60 usec
PLW1 39.81100082 W

F2 - Processing parameters
SI 32768
SF 700.0000165 MHz
WDW GM
SSB 0
LB -2.50 Hz
GB 0.08
PC 4.00

Figure S72. $^{13}C$ NMR spectrum (176.04 MHz, acetone-$d_6$) of **11**

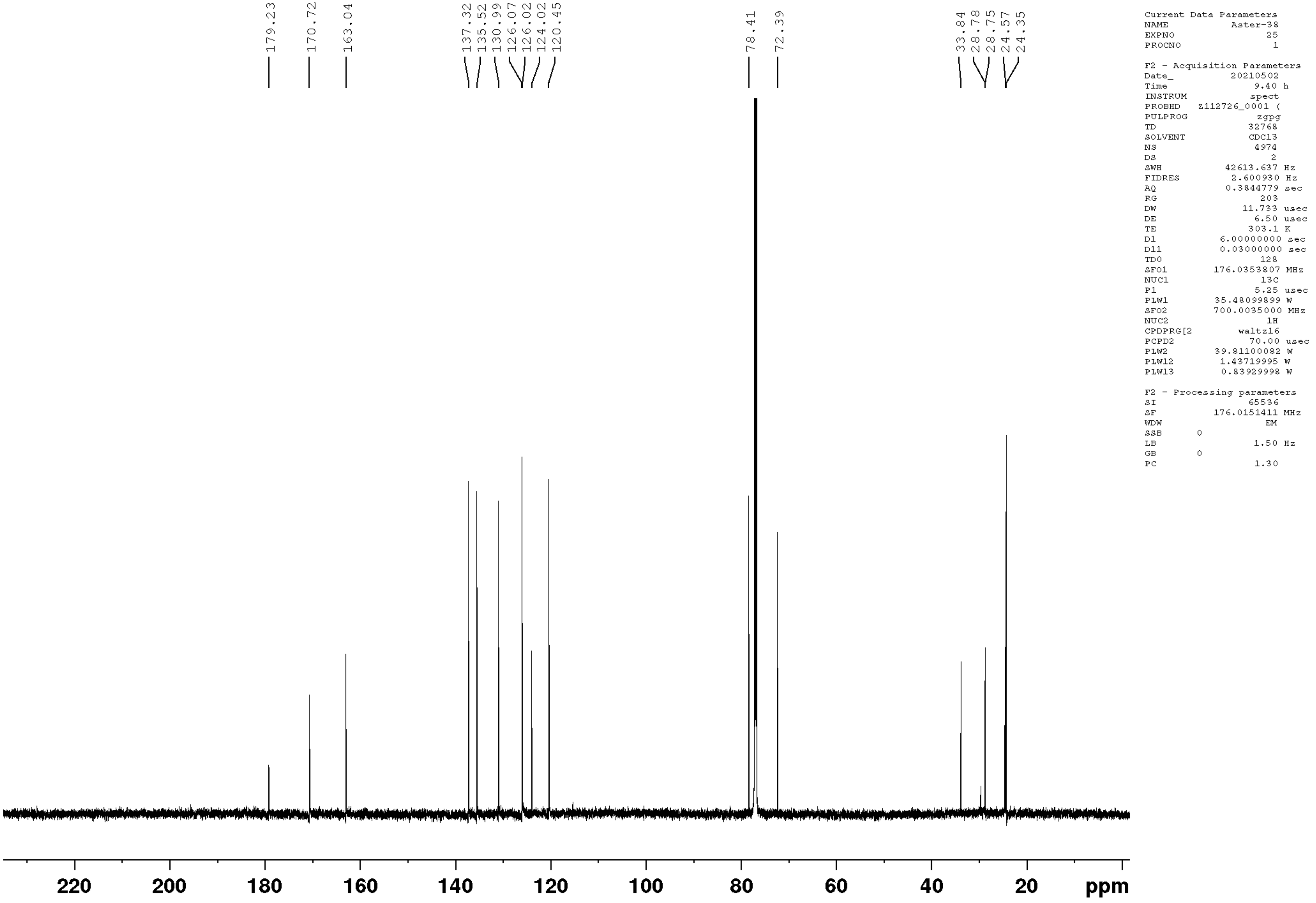

Figure S73. DEPT-135 NMR spectrum (176.04 MHz, acetone-$d_6$) of **11**

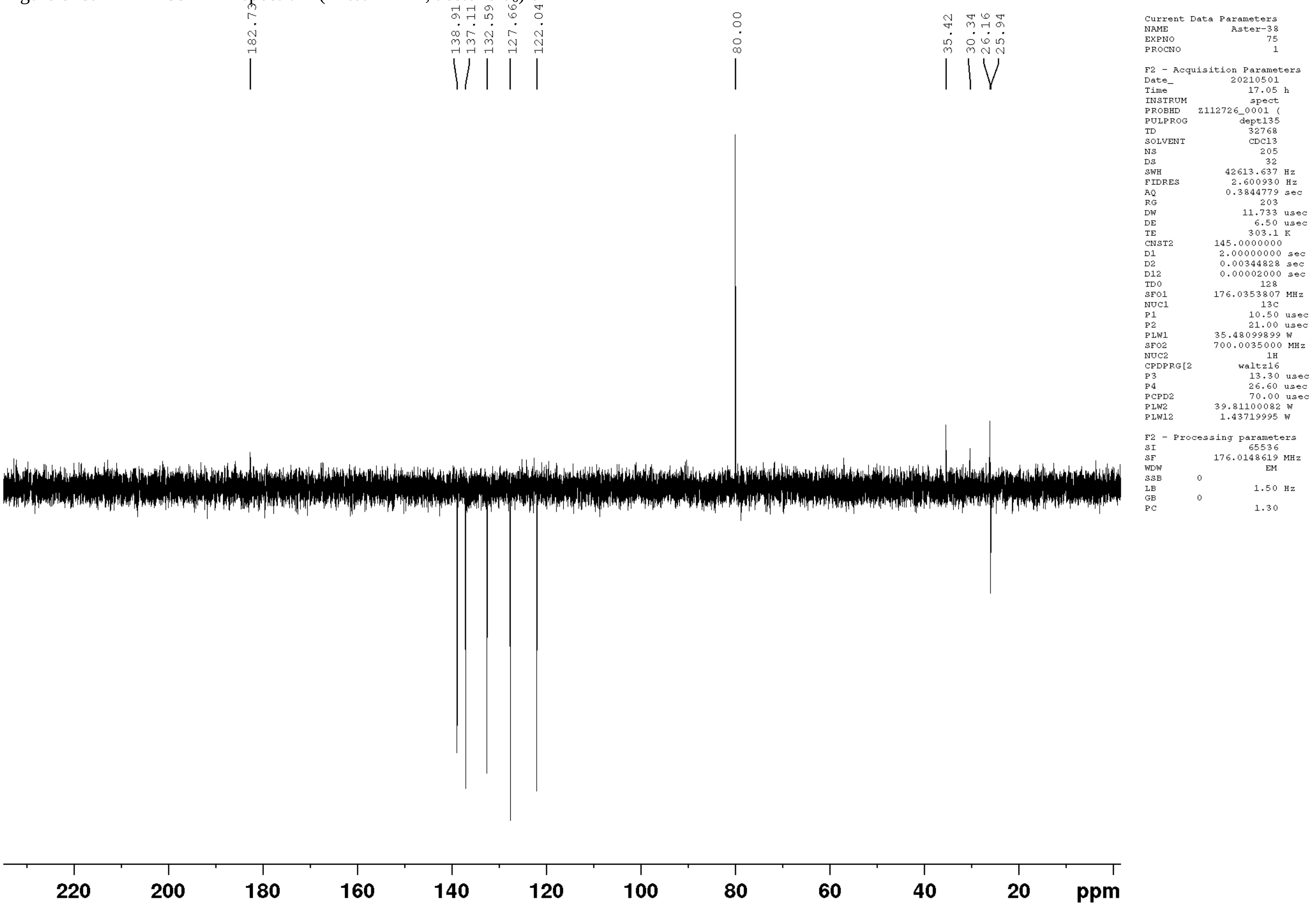

Figure S74. COSY-45 spectrum (700 MHz, acetone-$d_6$) of **11**

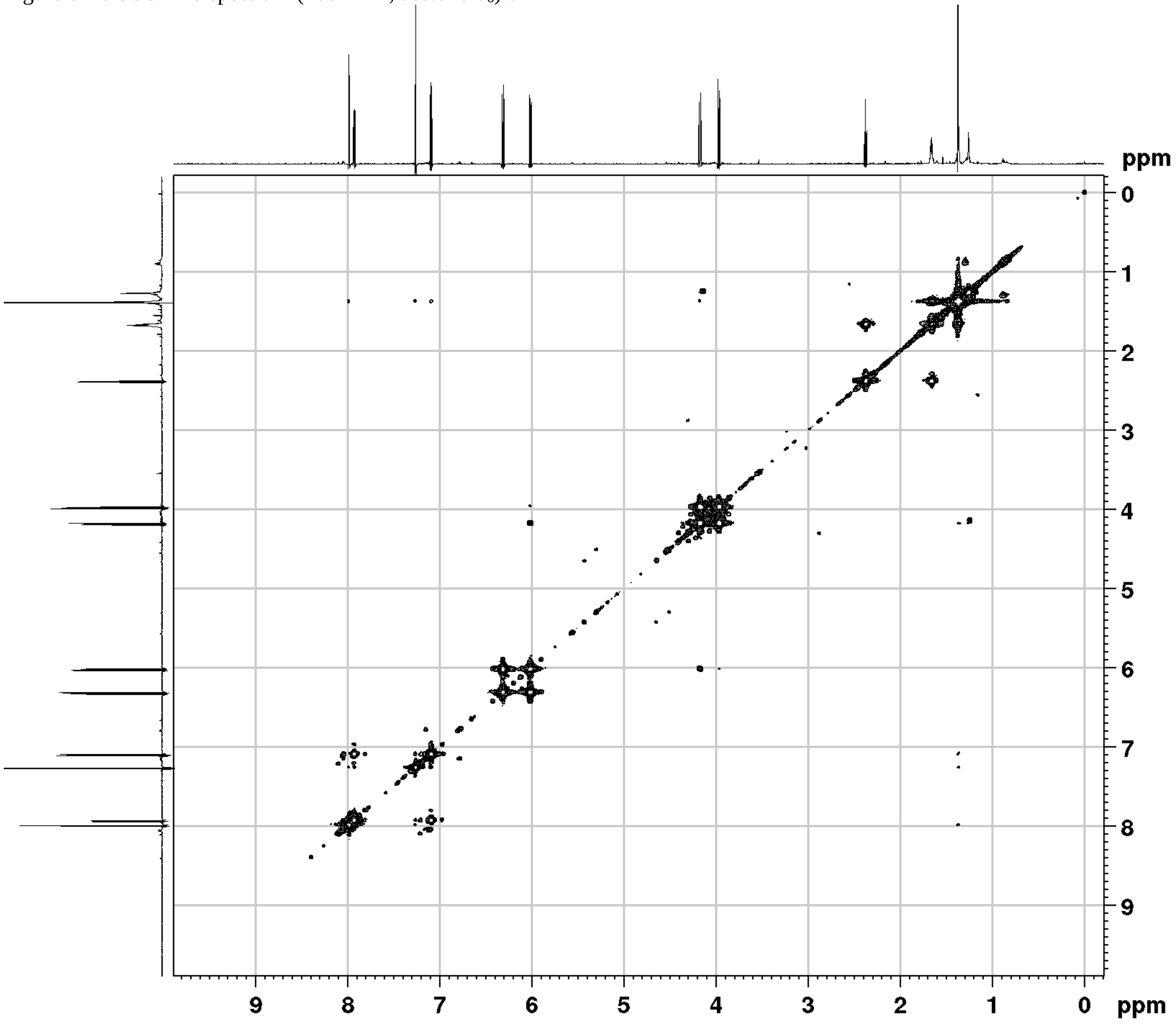


```
Current Data Parameters
NAME              Aster-38
EXPNO                   21
PROCNO                   1

F2 - Acquisition Parameters
Date_             20210501
Time                 17.08 h
INSTRUM              spect
PROBHD   Z112726_0001 (
PULPROG           cosygpqf
TD                    2048
SOLVENT              CDCl3
NS                       8
DS                       8
SWH               7530.121 Hz
FIDRES            7.353633 Hz
AQ               0.1359872 sec
RG                     203
DW                  66.400 usec
DE                    6.50 usec
TE                   302.9 K
D0              0.00000300 sec
D1              1.20000005 sec
D13             0.00000400 sec
D16             0.00020000 sec
IN0             0.00013280 sec
SPARSELI                 0
TDav                     1
SFO1           700.0031730 MHz
NUC1                    1H
P0                   12.60 usec
P1                   12.60 usec
PLW1           39.81100082 W
GPNAM[1]          SINE.100
GPZ1                 10.00 %
P16                1000.00 usec

F1 - Acquisition parameters
TD                     128
SFO1              700.0032 MHz
FIDRES          117.658134 Hz
SW                  10.757 ppm
FnMODE                  QF

F2 - Processing parameters
SI                    2048
SF             700.0000165 MHz
WDW                   SINE
SSB      0
LB       0 Hz
GB       0
PC                    1.00

F1 - Processing parameters
SI                    2048
MC2                     QF
SF             700.0000165 MHz
WDW                   SINE
SSB      0
LB       0 Hz
GB       0
```

Figure S75. HSQC spectrum (700 MHz, acetone-$d_6$) of **11**

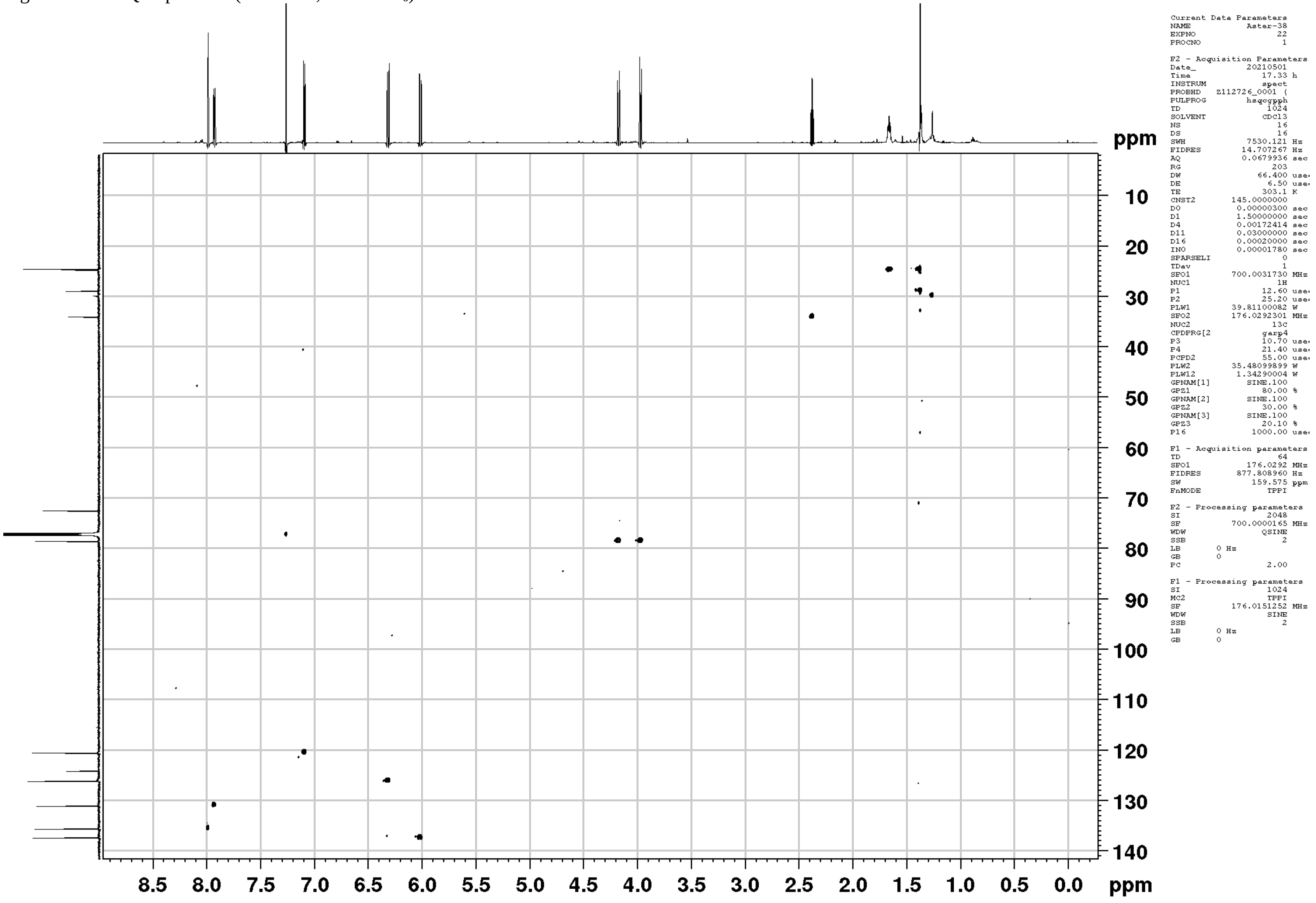

Figure S76. HMBC spectrum (700 MHz, acetone-d$_6$) of **11**

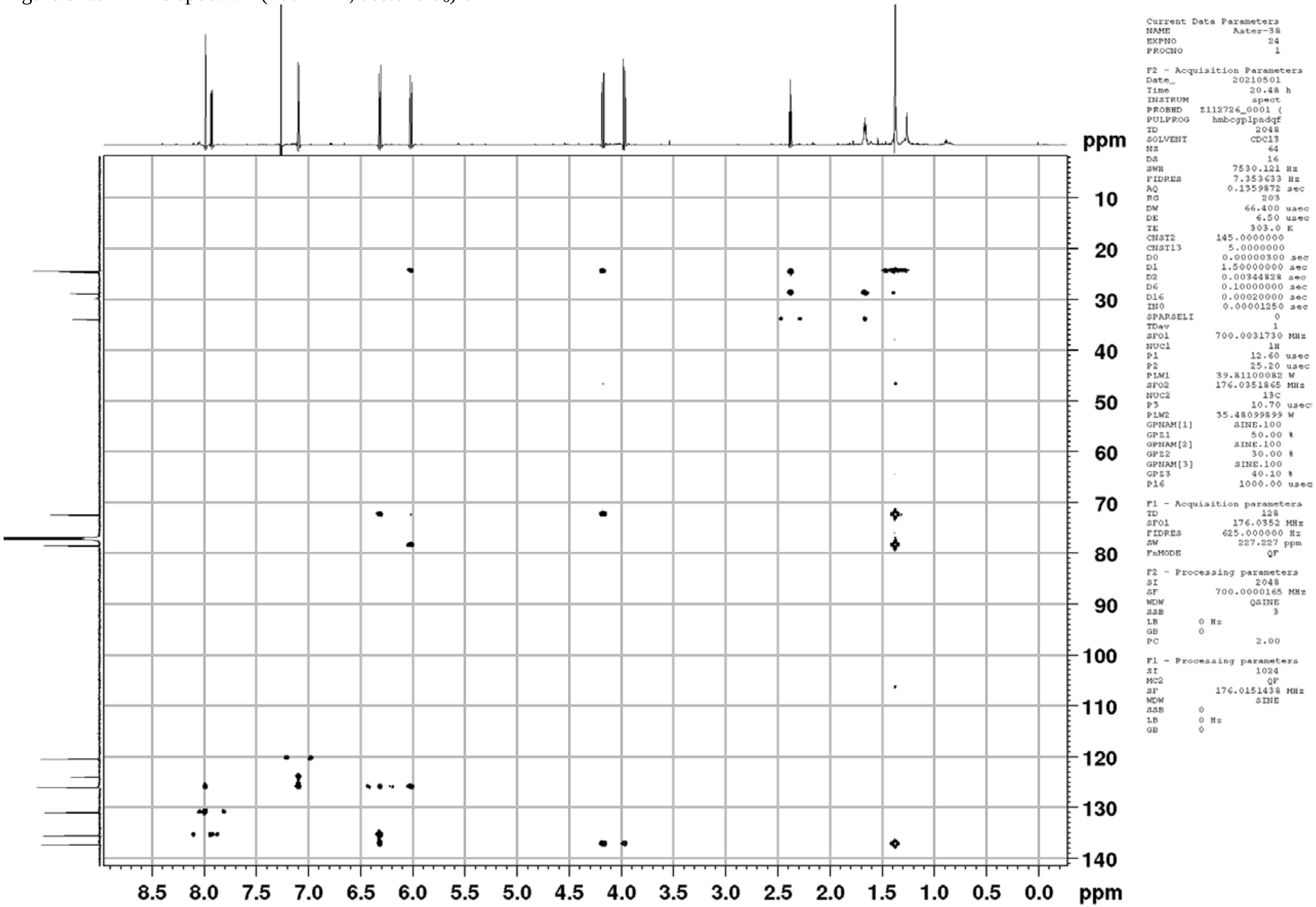

Figure S77. ROESY spectrum (700 MHz, acetone-d$_6$) of **11**

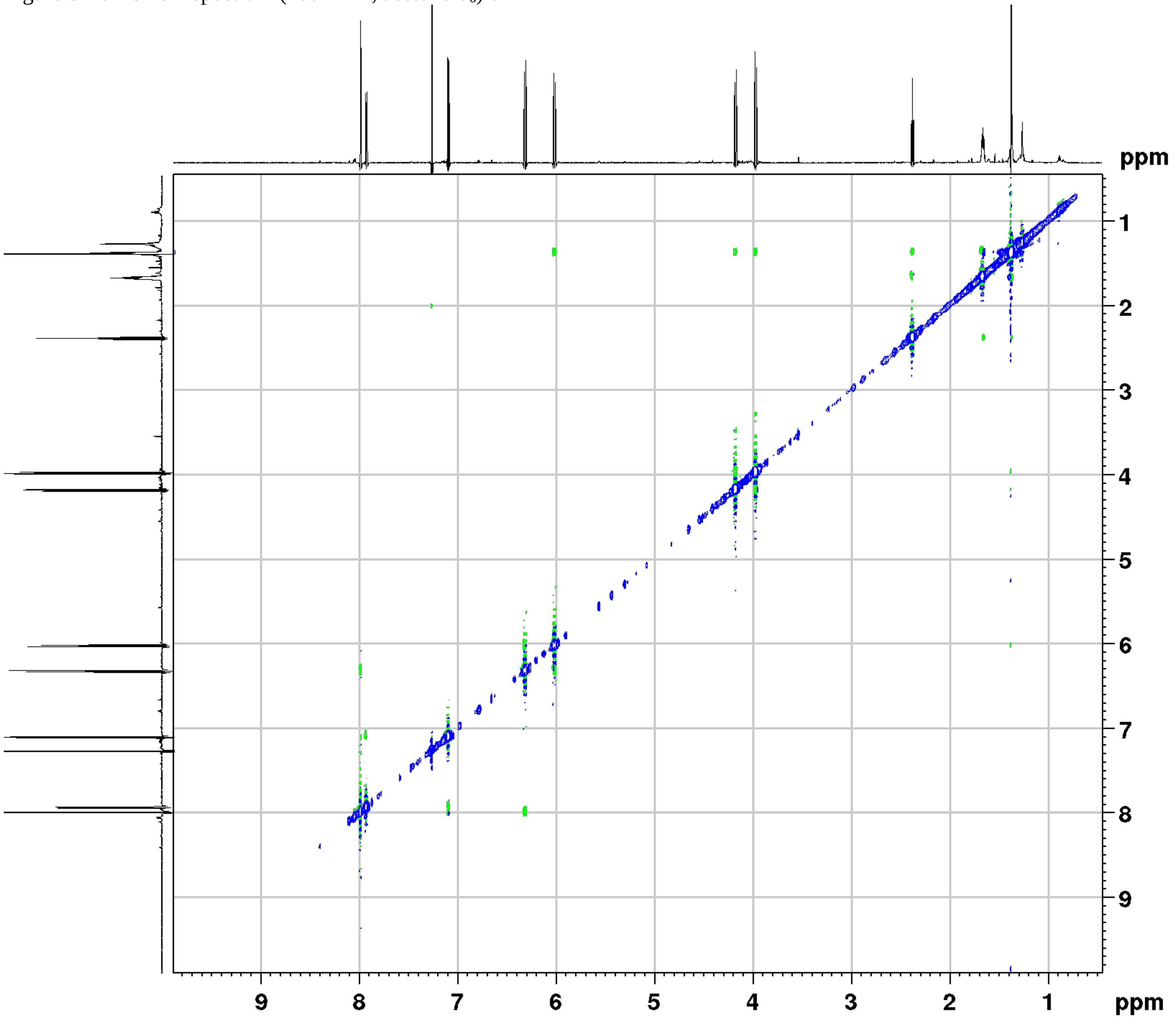


```
Current Data Parameters
NAME              Aster-38
EXPNO                   23
PROCNO                   1

F2 - Acquisition Parameters
Date_             20210501
Time                 18.01 h
INSTRUM              spect
PROBHD   Z112726_0001 (
PULPROG          roesyph.2
TD                    2048
SOLVENT              CDCl3
NS                      16
DS                      16
SWH               7530.121 Hz
FIDRES            7.353633 Hz
AQ               0.1359872 sec
RG                       2
DW                  66.400 usec
DE                    6.50 usec
TE                   303.0 K
D0              0.00005438 sec
D1              2.00000000 sec
D12             0.00002000 sec
IN0             0.00013280 sec
L4                     877
P15              270000.00 usec
TDav                     1
SFO1        700.0031730 MHz
NUC1                    1H
P1                   12.60 usec
P25                 154.00 usec
PLW1           39.81100082 W
PLW27           1.06599998 W

F1 - Acquisition parameters
TD                     256
SFO1           700.0032 MHz
FIDRES        58.829067 Hz
SW                  10.757 ppm
FnMODE                TPPI

F2 - Processing parameters
SI                    2048
SF             700.0000165 MHz
WDW                   SINE
SSB                      2
LB          0 Hz
GB          0
PC                    4.00

F1 - Processing parameters
SI                     512
MC2                   TPPI
SF             700.0000165 MHz
WDW                   SINE
SSB                      2
LB          0 Hz
GB          0
```

Figure S78. $^{1}$H NMR spectrum (500 MHz, acetone-$d_6$) of **12**

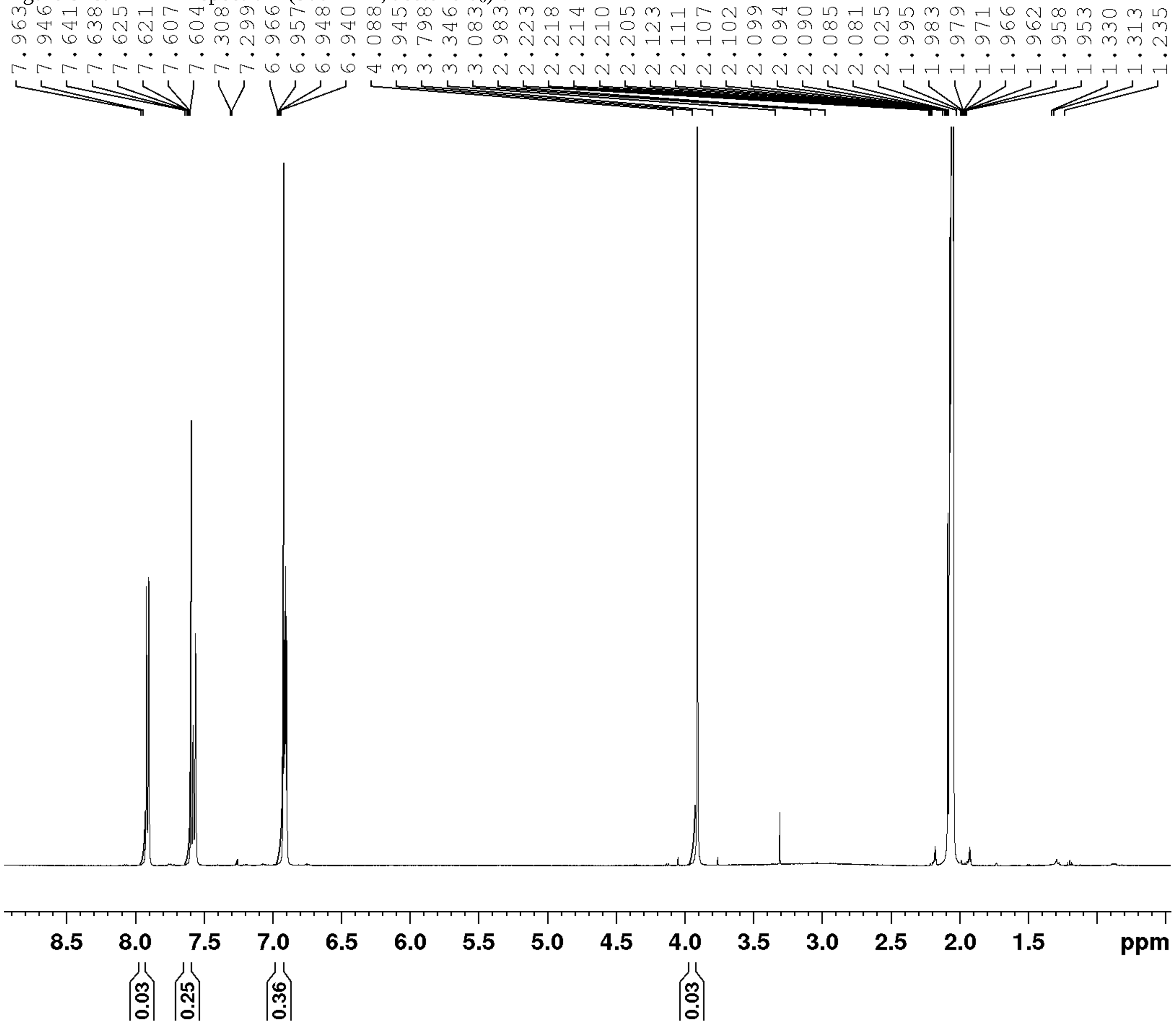


Current Data Parameters
NAME Aster-8a
EXPNO 5
PROCNO 1

F2 - Acquisition Parameters
Date_ 20210218
Time 12.50 h
INSTRUM spect
PROBHD Z113652_0155 (
PULPROG zg30
TD 32768
SOLVENT Acetone
NS 8
DS 2
SWH 4251.701 Hz
FIDRES 0.259503 Hz
AQ 3.8535168 sec
RG 119.65
DW 117.600 usec
DE 6.50 usec
TE 303.1 K
D1 2.00000000 sec
TD0 1
SFO1 500.1323695 MHz
NUC1 1H
P0 3.88 usec
P1 11.65 usec
PLW1 15.84899998 W

F2 - Processing parameters
SI 65536
SF 500.1300134 MHz
WDW EM
SSB 0
LB 0.30 Hz
GB 0
PC 1.00

Figure S79. $^{13}$C NMR spectrum (125.77 MHz, acetone-d$_6$) of **12**

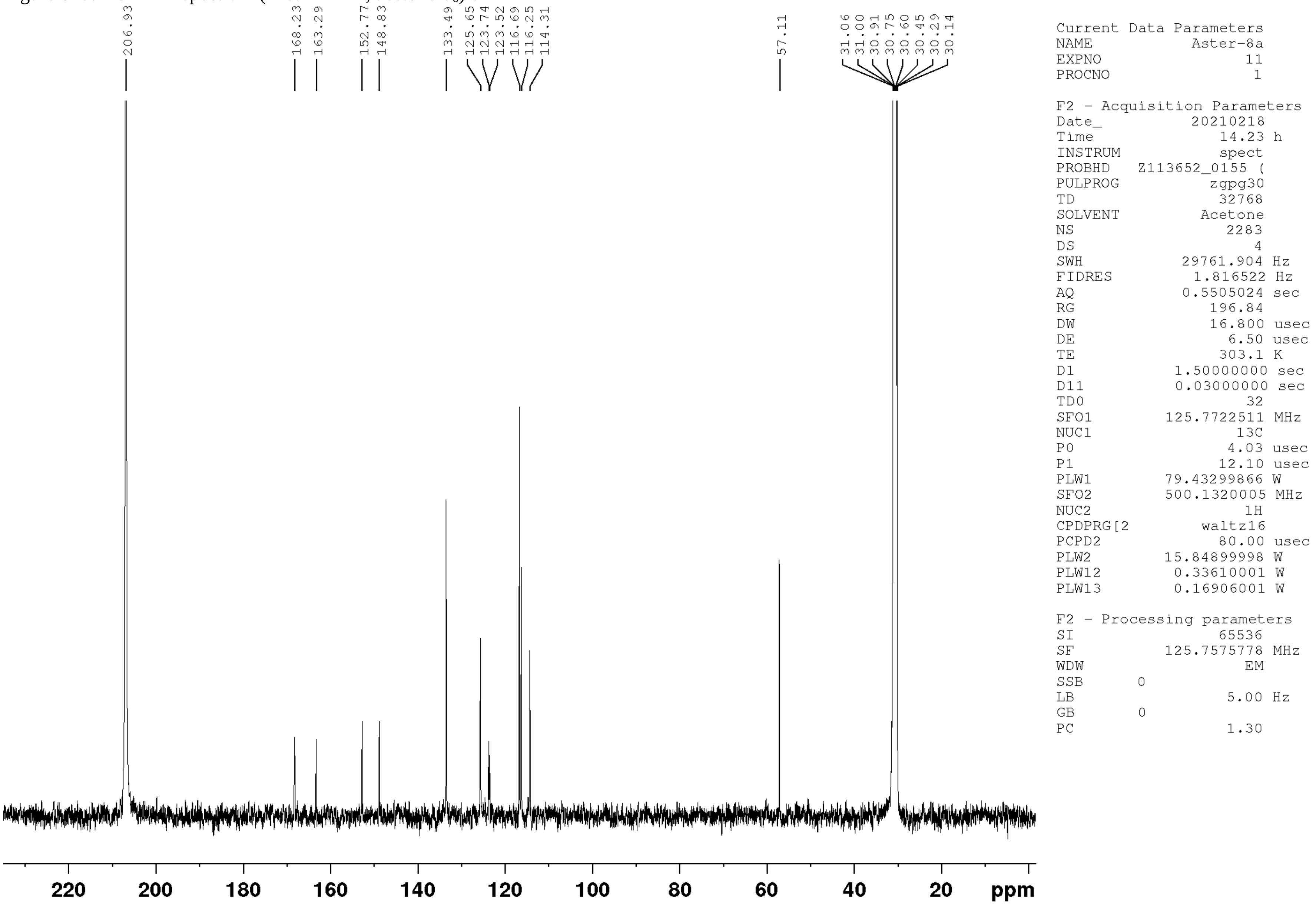

Figure S80. COSY-45 spectrum (500 MHz, acetone-$d_6$) of **12**

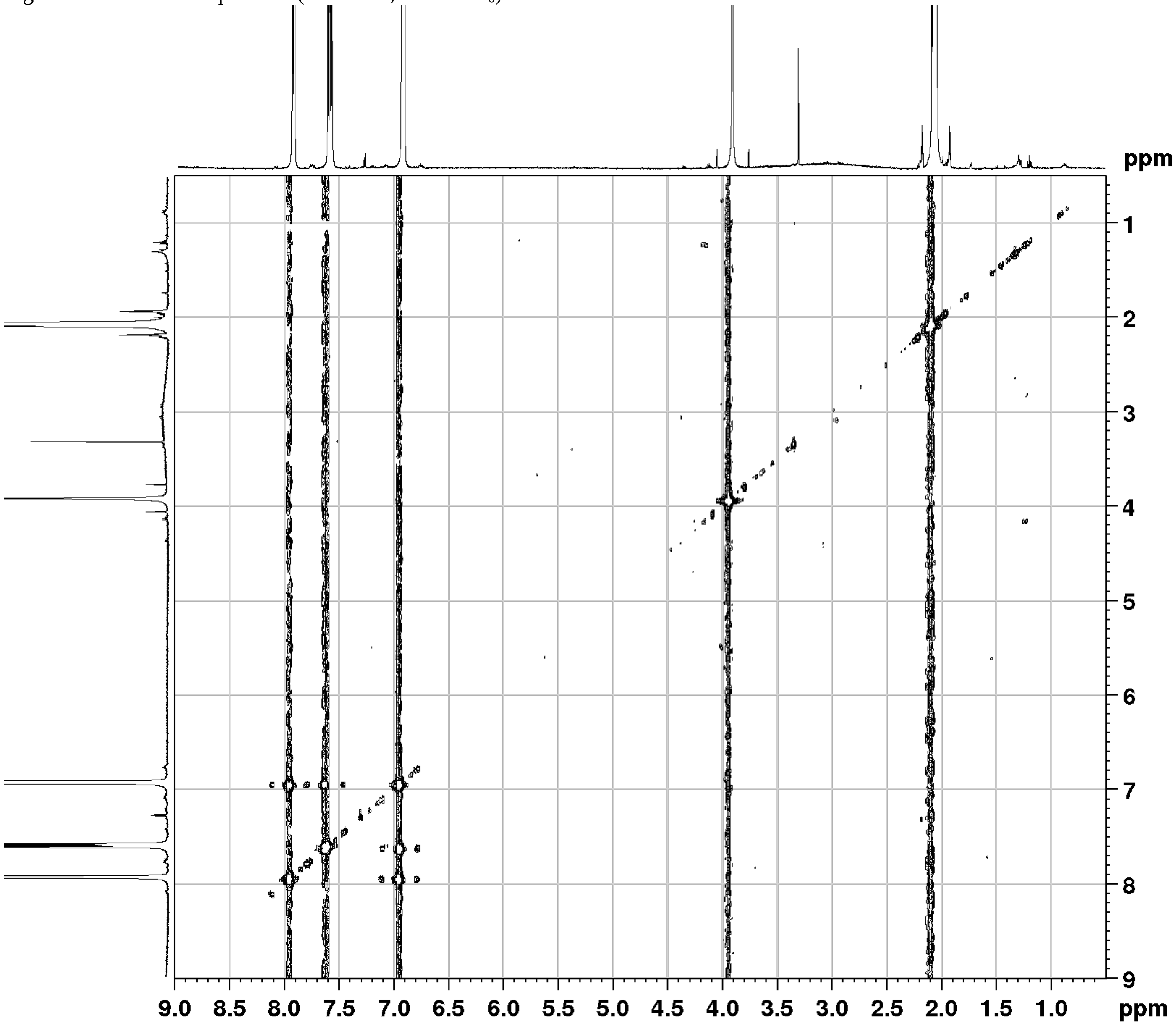


```
Current Data Parameters
NAME            Aster-8a
EXPNO                 21
PROCNO                 1

F2 - Acquisition Parameters
Date_           20210218
Time               12.53 h
INSTRUM            spect
PROBHD   Z113652_0155 (
PULPROG         cosygpqf
TD                  2048
SOLVENT          Acetone
NS                     4
DS                    16
SWH             4251.701 Hz
FIDRES          4.152051 Hz
AQ             0.2408448 sec
RG                196.84
DW               117.600 usec
DE                  6.50 usec
TE                 303.2 K
D0            0.00000300 sec
D1            1.20000005 sec
D13           0.00000400 sec
D16           0.00020000 sec
IN0           0.00023520 sec
SPARSELI               0
TDav                   1
SFO1         500.1323696 MHz
NUC1                  1H
P0                 11.65 usec
P1                 11.65 usec
PLW1         15.84899998 W
GPNAM[1]      SMSQ10.100
GPZ1               10.00 %
P16              1000.00 usec

F1 - Acquisition parameters
TD                    64
SFO1            500.1324 MHz
FIDRES        132.865646 Hz
SW                 8.501 ppm
FnMODE                QF

F2 - Processing parameters
SI                  2048
SF           500.1299935 MHz
WDW                QSINE
SSB      0
LB       0 Hz
GB       0
PC                  1.00

F1 - Processing parameters
SI                  2048
MC2                   QF
SF           500.1299935 MHz
WDW                QSINE
SSB      0
LB       0 Hz
GB       0
```

Figure S81. HSQC spectrum (500 MHz, acetone-d$_6$) of **12**

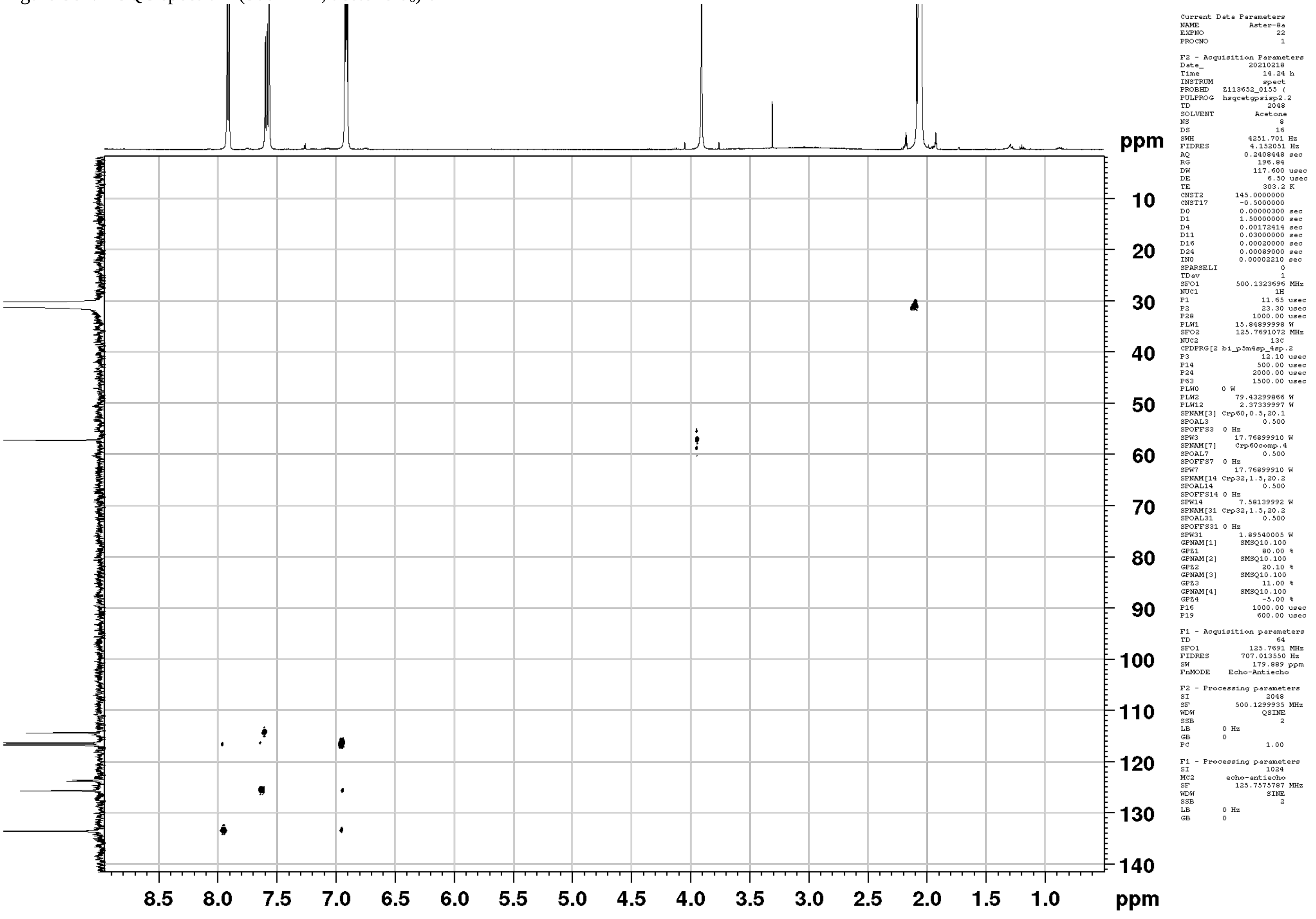

Figure S82. HMBC spectrum (700 MHz, acetone-$d_6$) of **12**

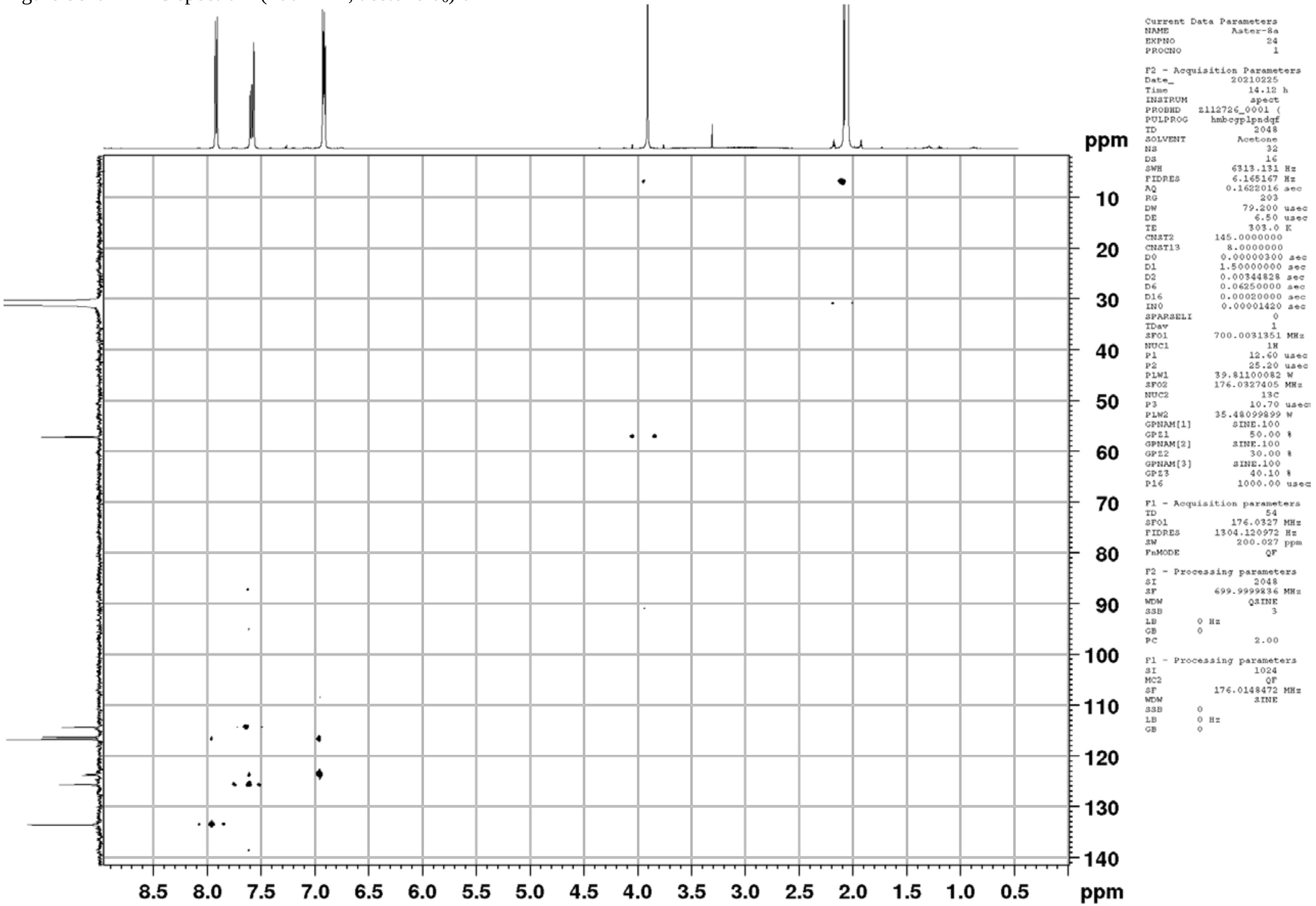

Figure S83. ROESY spectrum (700 MHz, acetone-$d_6$) of **12**

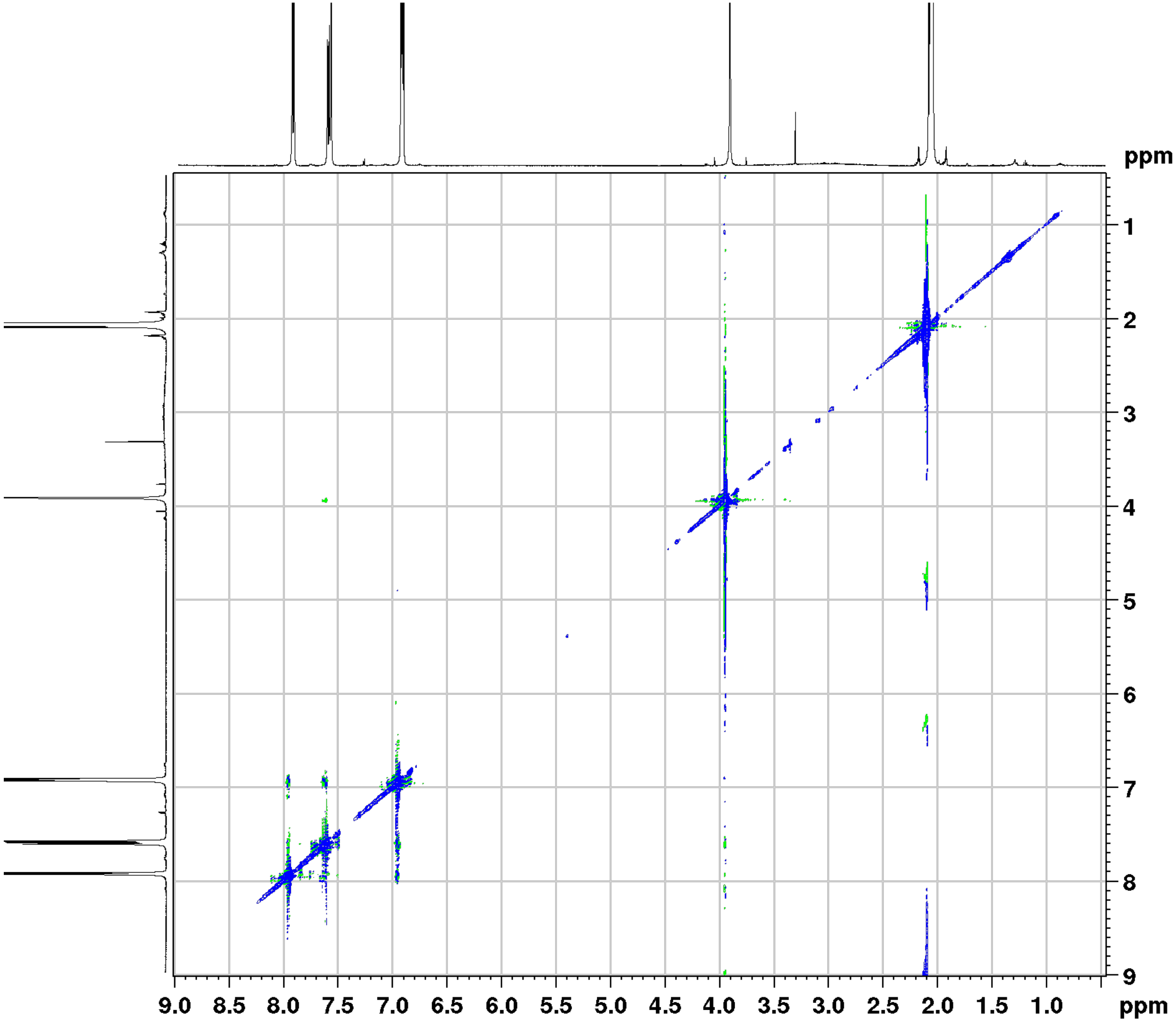


```
Current Data Parameters
NAME              Aster-8a
EXPNO                   23
PROCNO                   1

F2 - Acquisition Parameters
Date_             20210225
Time                 11.55 h
INSTRUM              spect
PROBHD    Z112726_0001 (
PULPROG          roesyph.2
TD                    2048
SOLVENT            Acetone
NS                      16
DS                      16
SWH               6313.131 Hz
FIDRES            6.165167 Hz
AQ               0.1622016 sec
RG                       4
DW                  79.200 usec
DE                    6.50 usec
TE                   303.1 K
D0              0.00006718 sec
D1              1.50000000 sec
D12             0.00002000 sec
IN0             0.00015840 sec
L4                     649
P15              200000.00 usec
TDav                     1
SFO1        700.0031351 MHz
NUC1                    1H
P1                   12.60 usec
P25                 154.00 usec
PLW1           39.81100082 W
PLW27           1.06599998 W

F1 - Acquisition parameters
TD                     256
SFO1           700.0031 MHz
FIDRES        49.321339 Hz
SW                   9.019 ppm
FnMODE                TPPI

F2 - Processing parameters
SI                    2048
SF             699.9999836 MHz
WDW                   SINE
SSB                      2
LB          0 Hz
GB          0
PC                    2.00

F1 - Processing parameters
SI                    1024
MC2                   TPPI
SF             699.9999836 MHz
WDW                   SINE
SSB                      2
LB          0 Hz
GB          0
```

Figure S84. $^{1}$H NMR spectrum (700 MHz, $CDCl_3$) of **13**

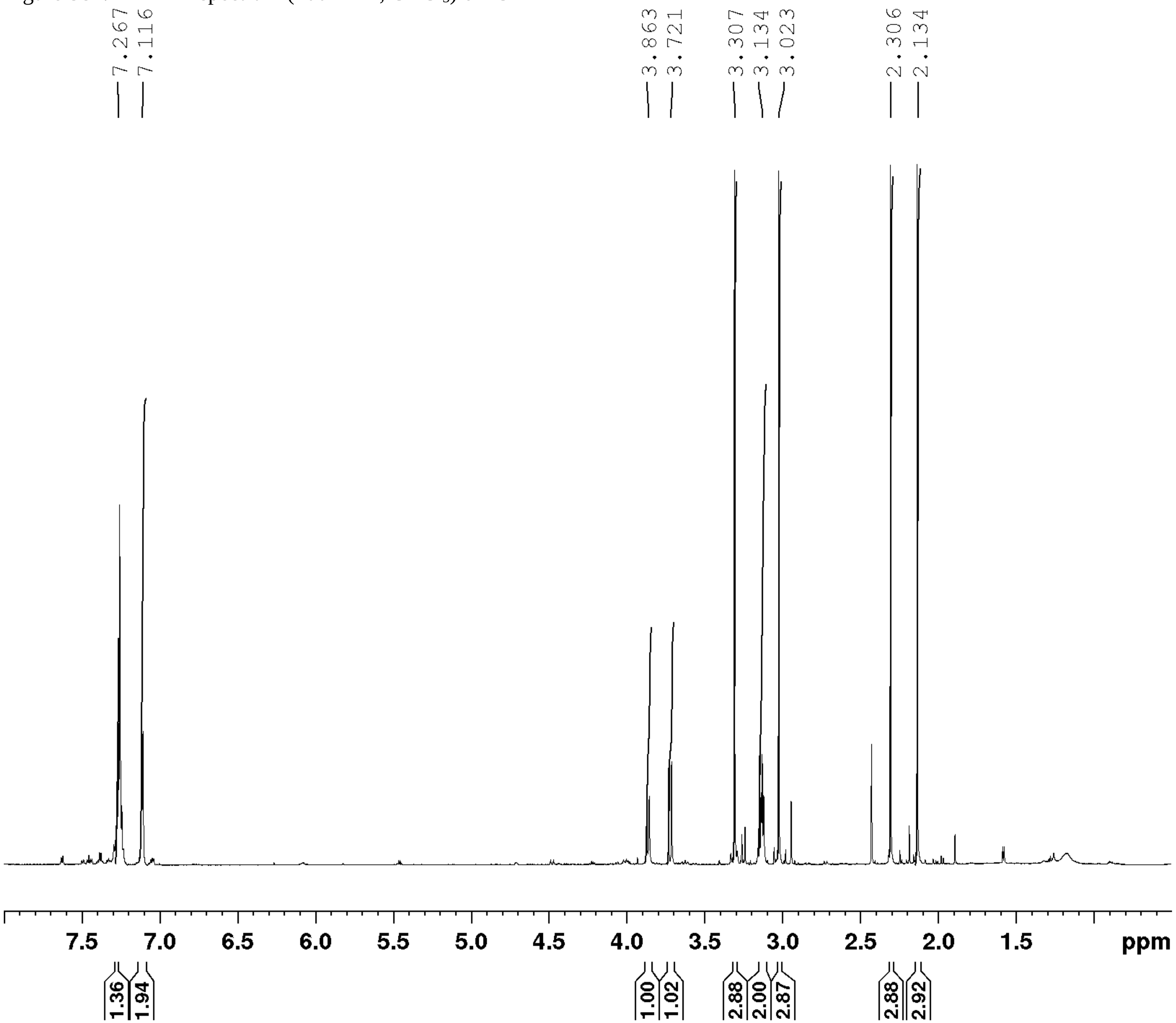


Current Data Parameters
NAME Aster-5
EXPNO 5
PROCNO 1

F2 - Acquisition Parameters
Date_ 20210201
Time 13.54 h
INSTRUM spect
PROBHD Z112726_0001 (
PULPROG zg30
TD 32768
SOLVENT CDCl3
NS 8
DS 2
SWH 5252.101 Hz
FIDRES 0.320563 Hz
AQ 3.1195135 sec
RG 203
DW 95.200 usec
DE 6.50 usec
TE 302.6 K
D1 2.00000000 sec
TD0 1
SFO1 700.0029934 MHz
NUC1 1H
P0 4.20 usec
P1 12.60 usec
PLW1 39.81100082 W

F2 - Processing parameters
SI 32768
SF 700.0000161 MHz
WDW EM
SSB 0
LB 0.30 Hz
GB 0
PC 1.00

Figure S85. $^{13}C$ NMR spectrum (176.04 MHz, $CDCl_3$) of **13**

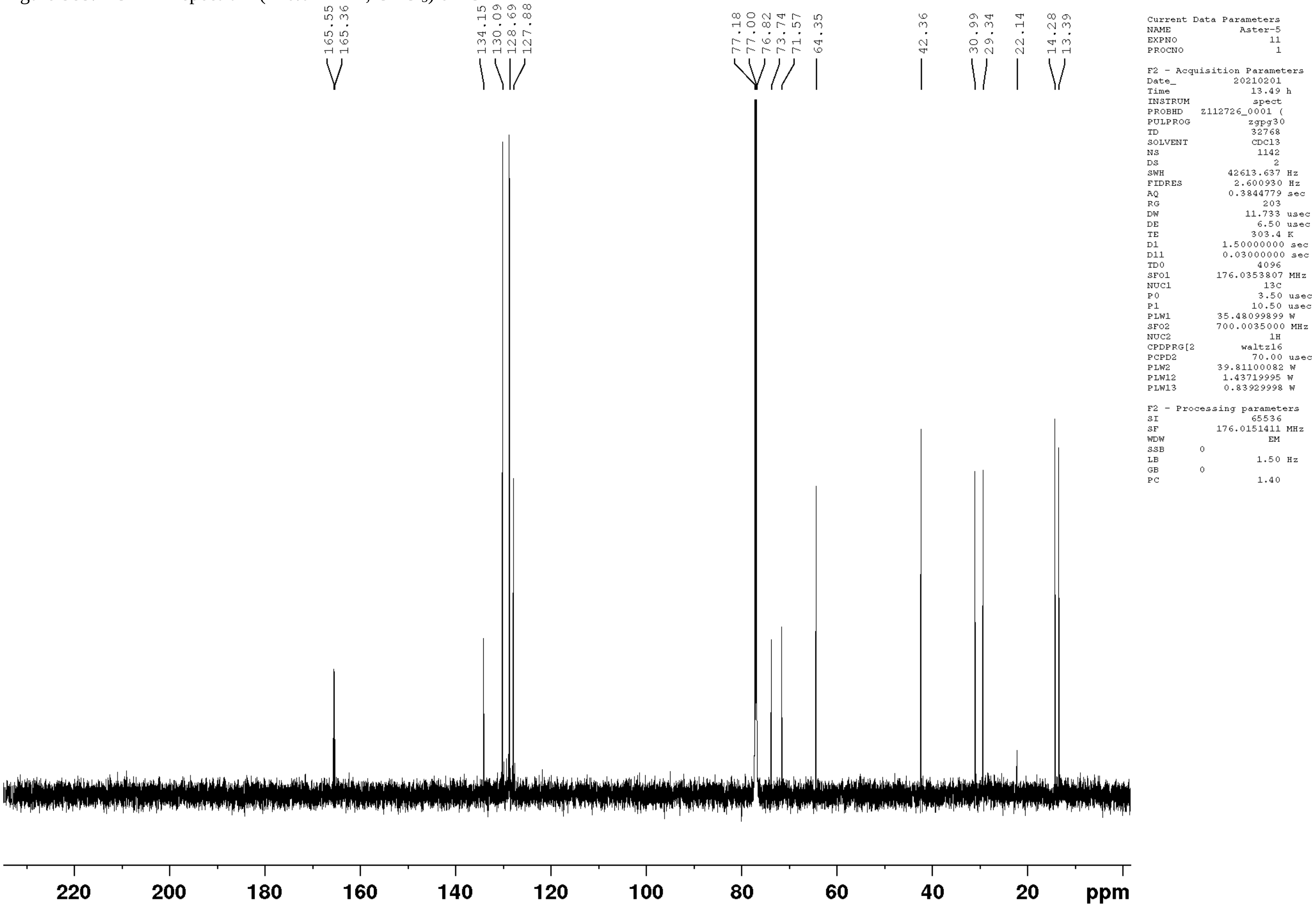

Figure S86. DEPT-135 NMR spectrum (176.04 MHz, $CDCl_3$) of **13**

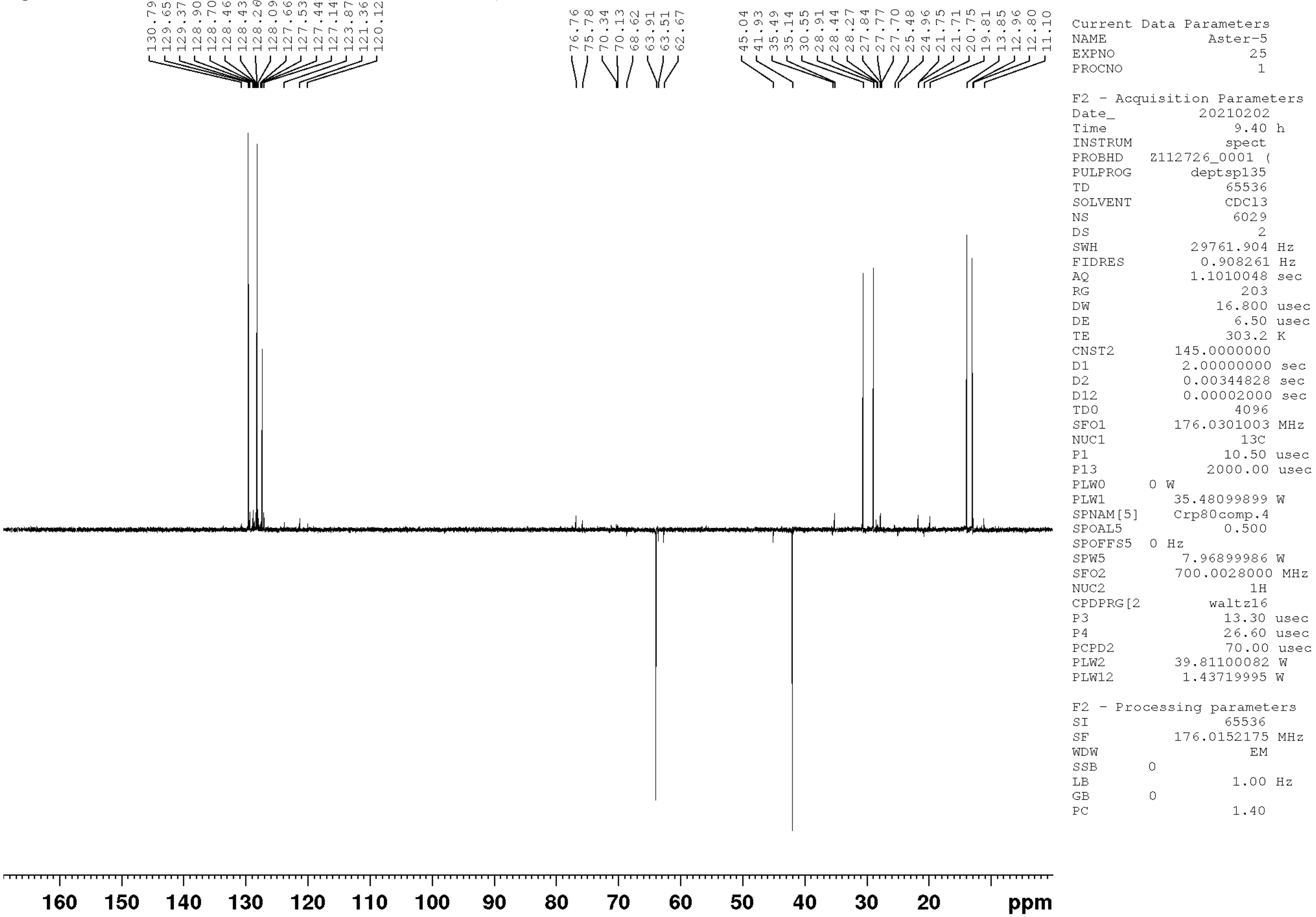

Figure S87. COSY-45 spectrum (700 MHz, $CDCl_3$) of **13**

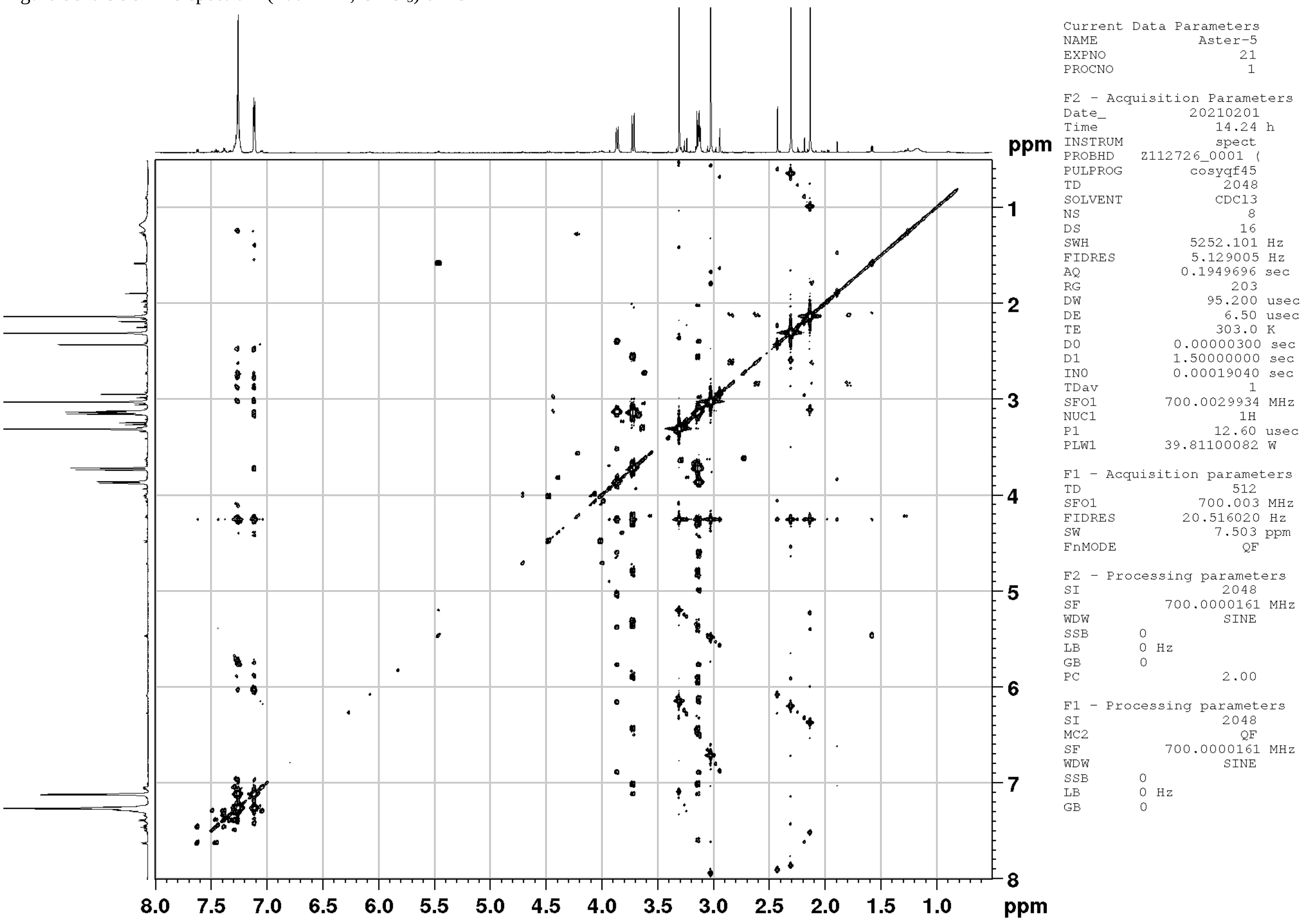

Figure S88. HSQC spectrum (700 MHz, $CDCl_3$) of **13**

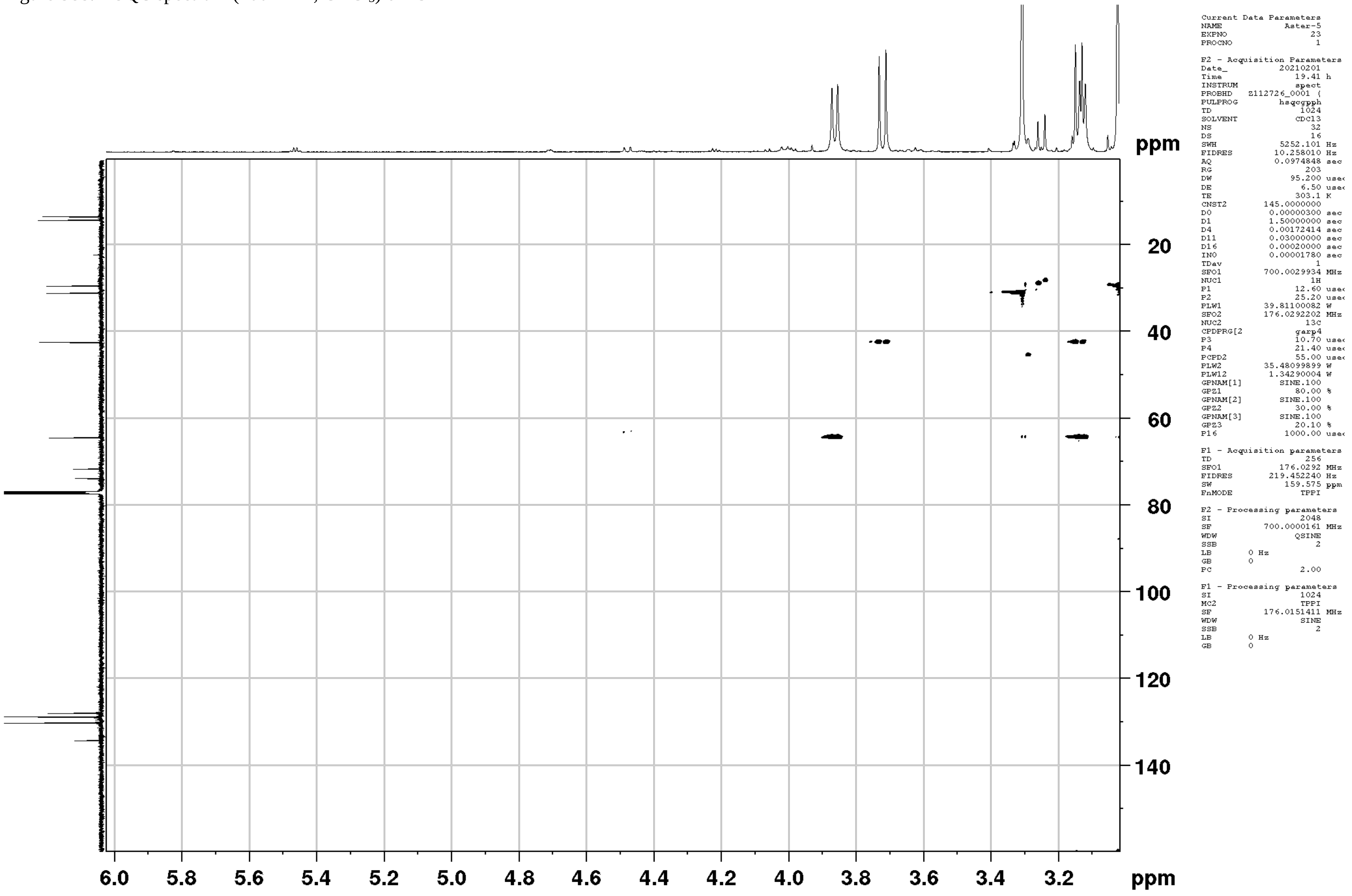

Figure S89. HMBC spectrum (700 MHz, $CDCl_3$) of **13**

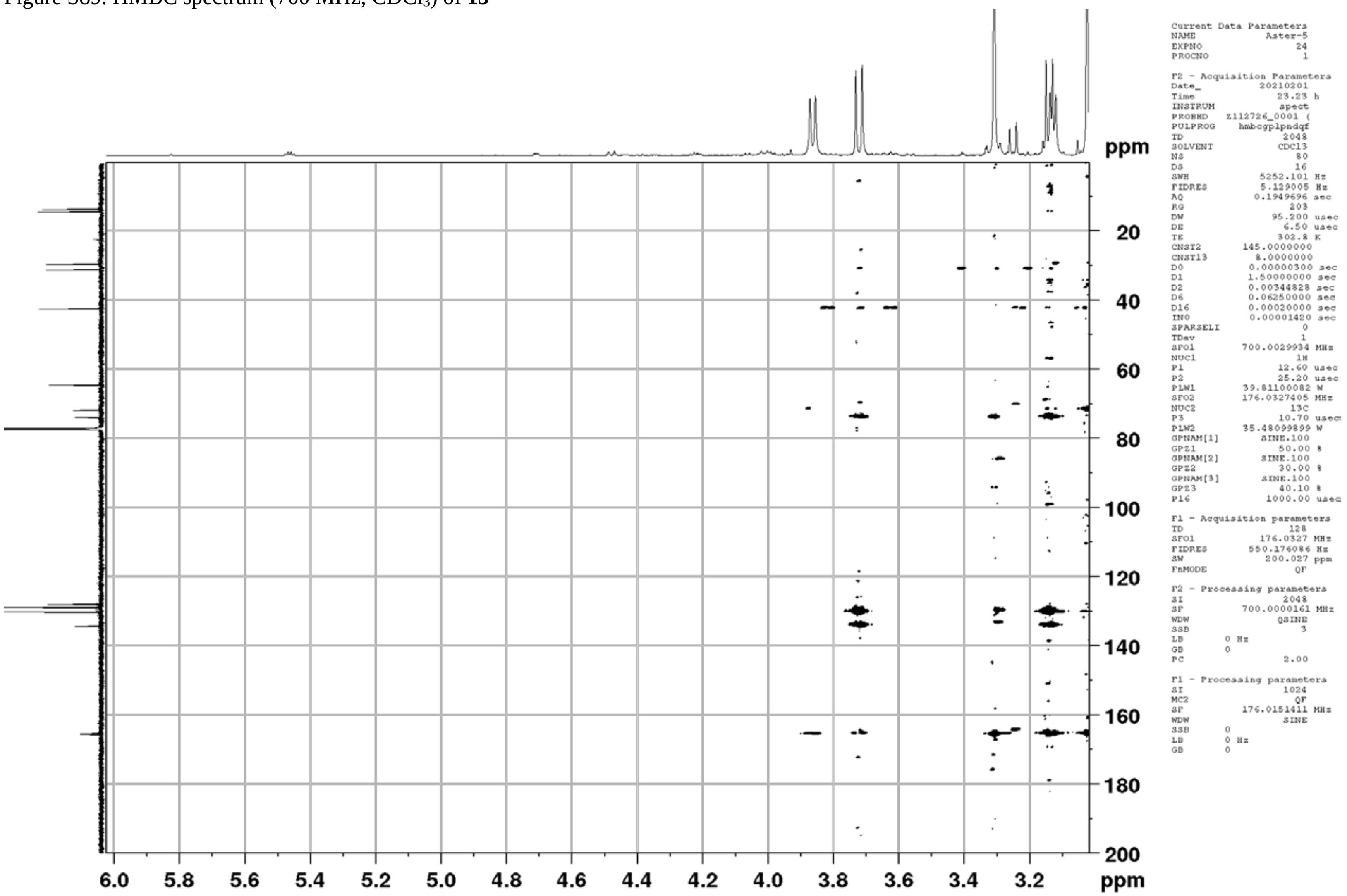

Figure S90. ROESY spectrum (700 MHz, $CDCl_3$) of **13**

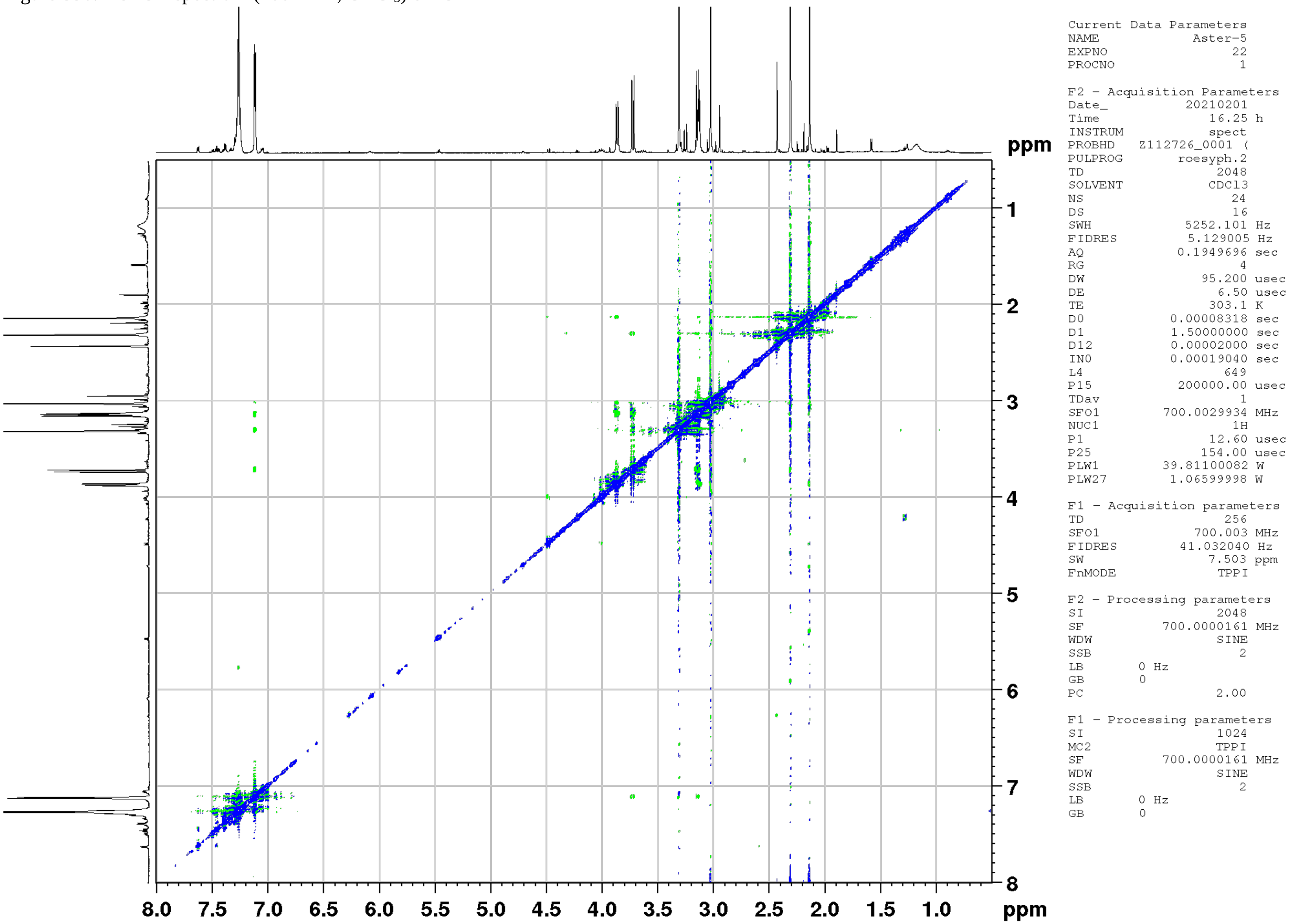

Figure S91. (+) ESI MS of **1**

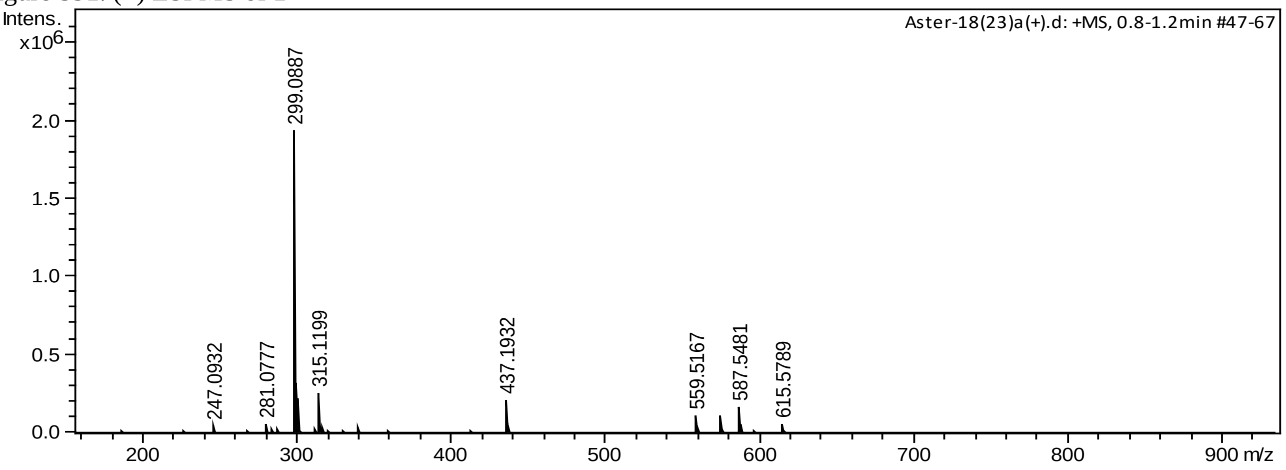


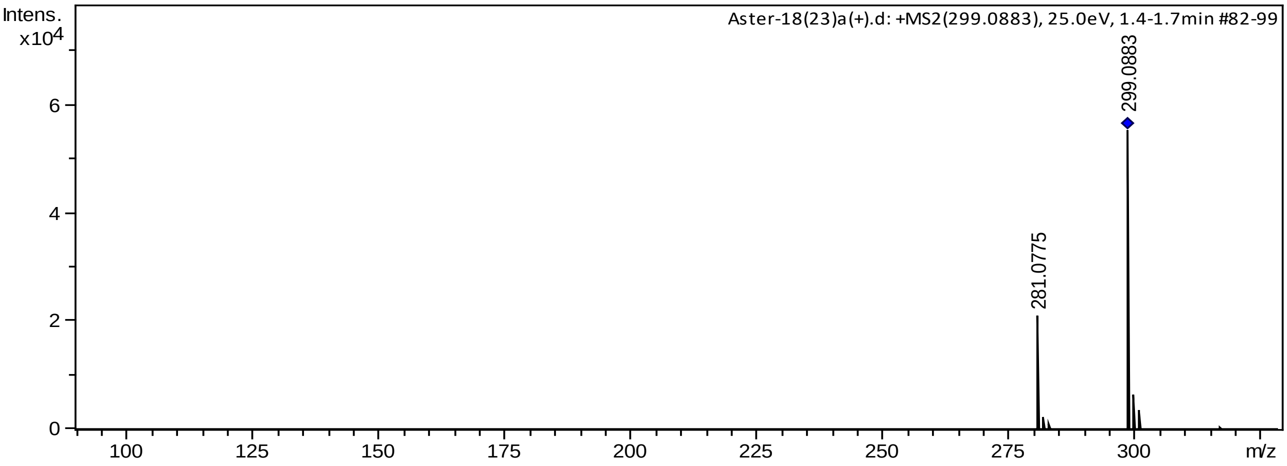


Figure S92. (-) ESI MS of **1**

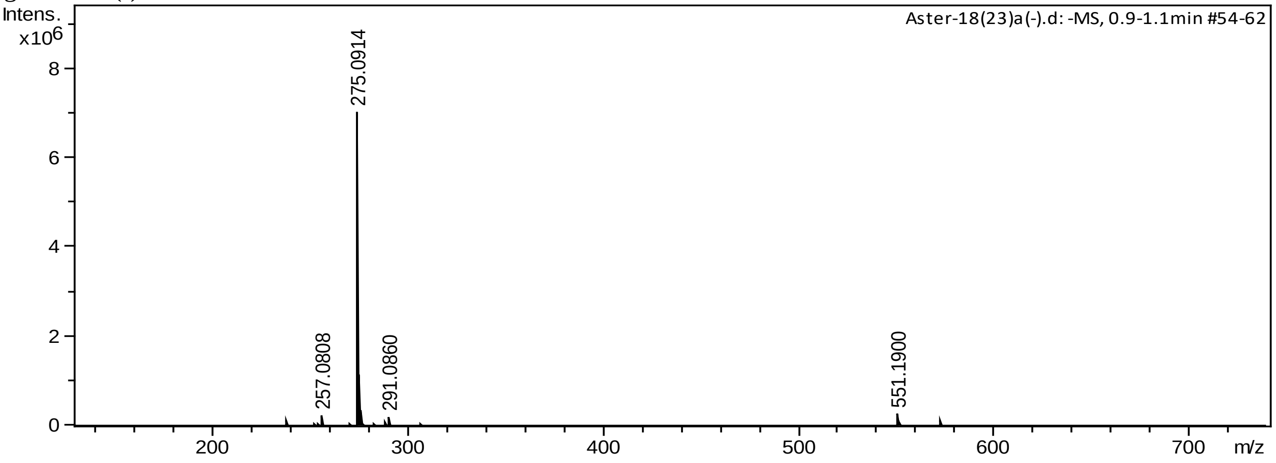


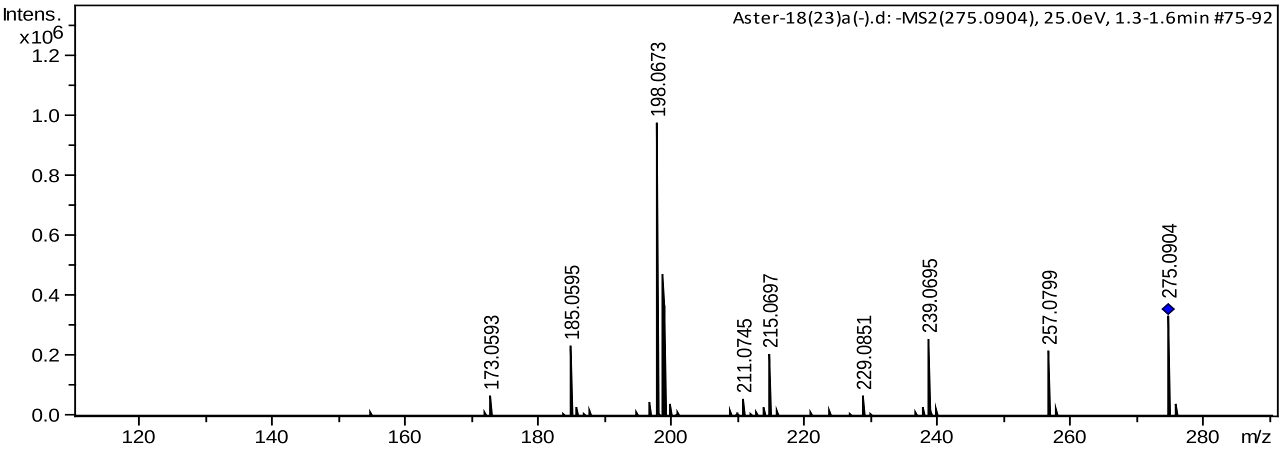

Figure S93. (+) ESI MS of **2**

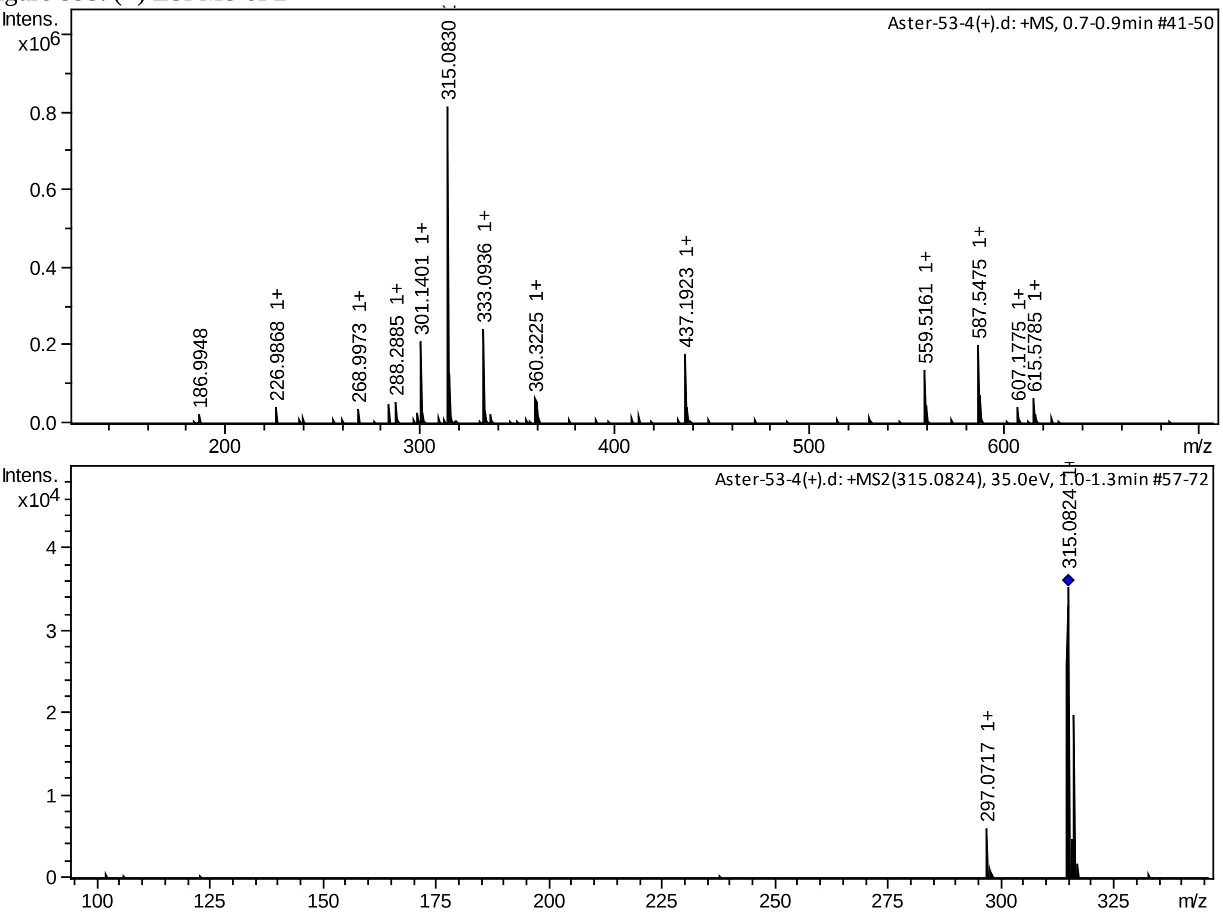


Figure S94. (-) ESI MS of **2**

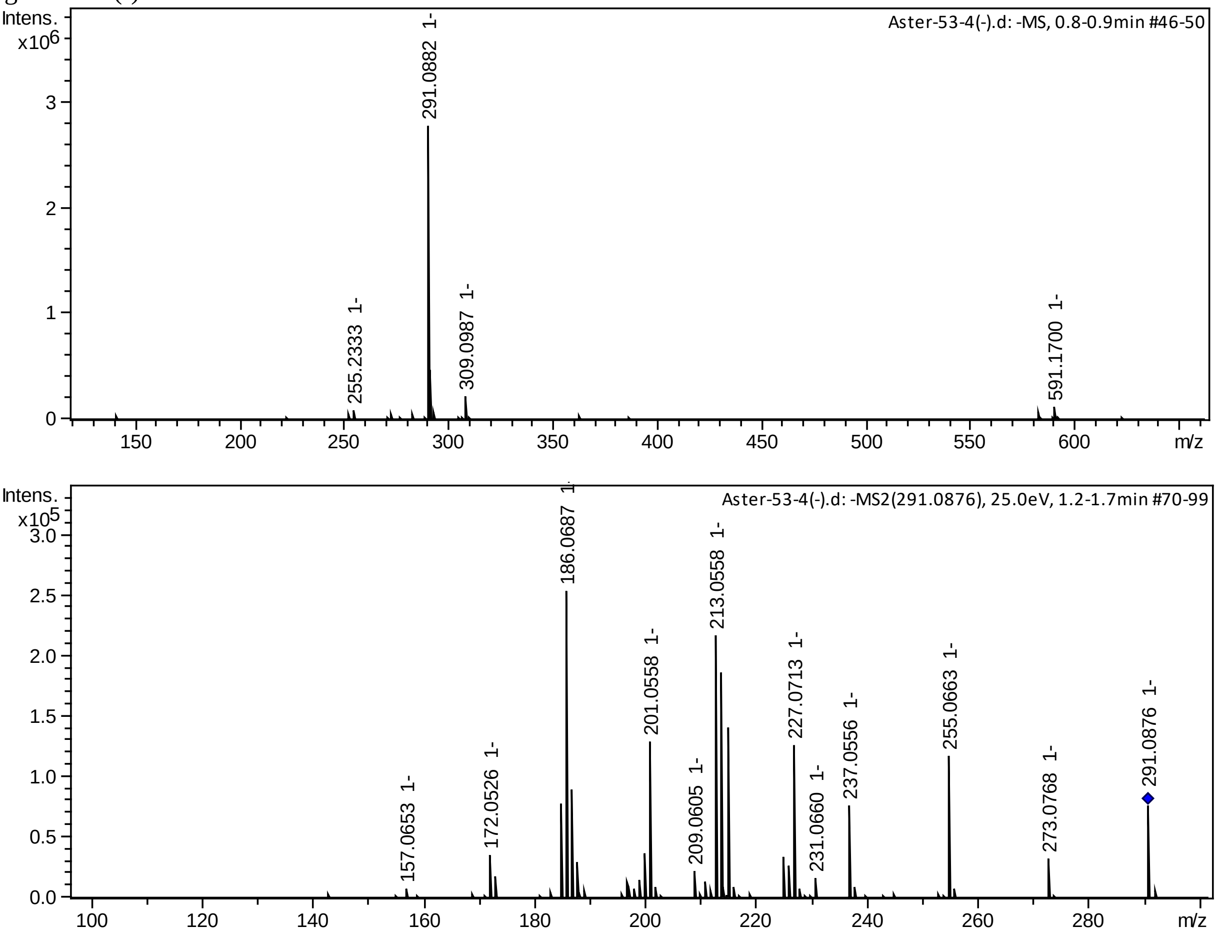

Figure S95. (+) ESI MS of **3**

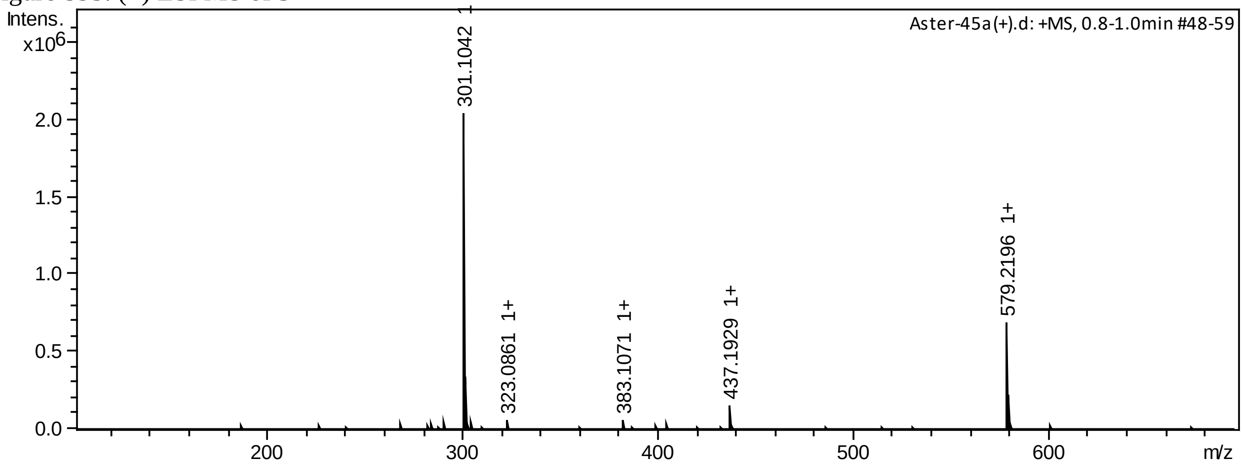


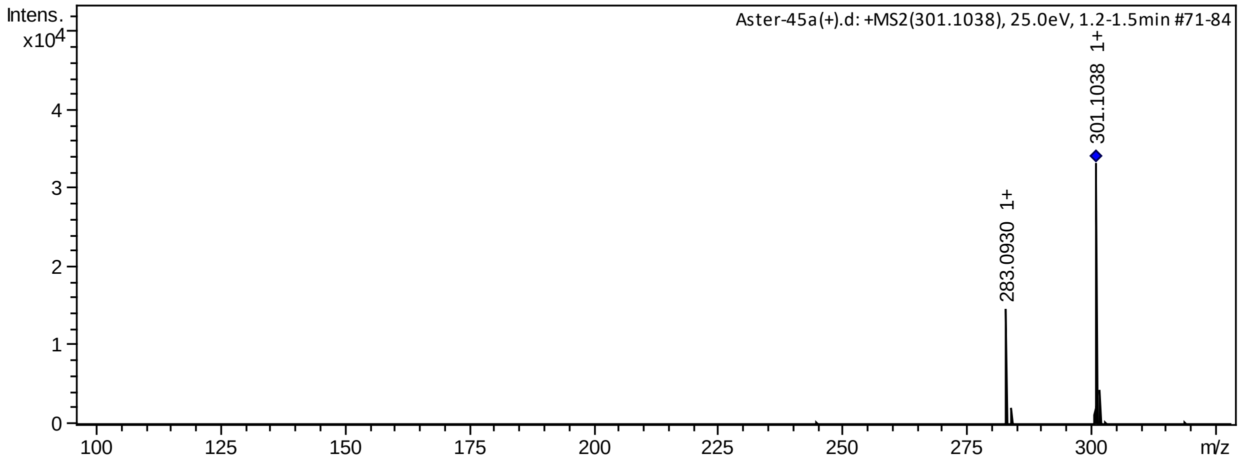


Figure S96. (-) ESI MS of **3**

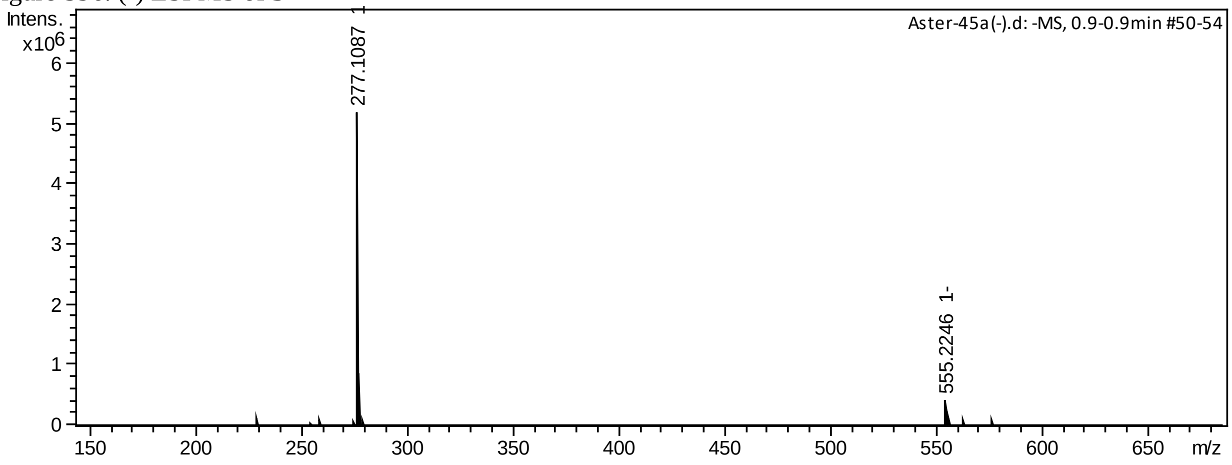


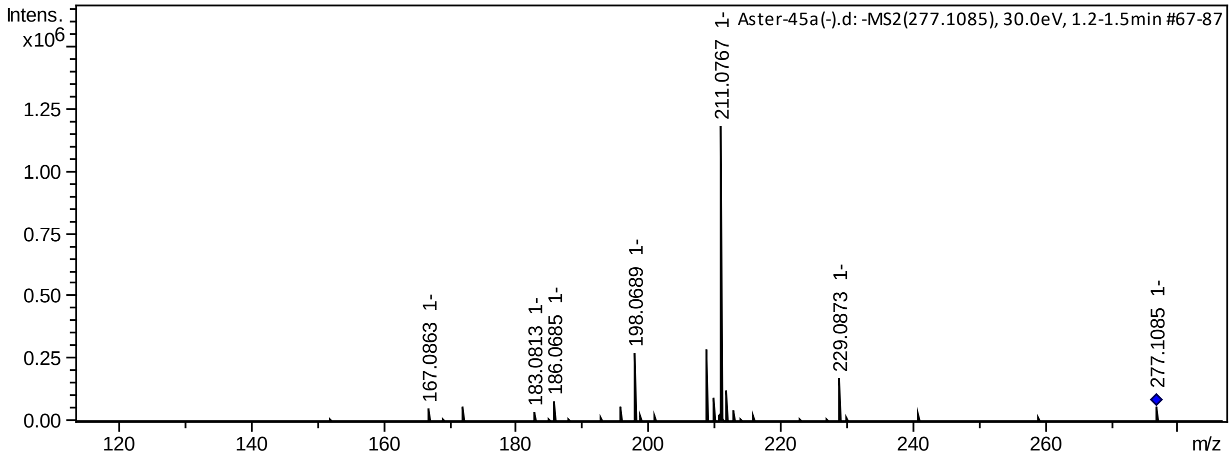

Figure S97. (+) ESI MS of **6**

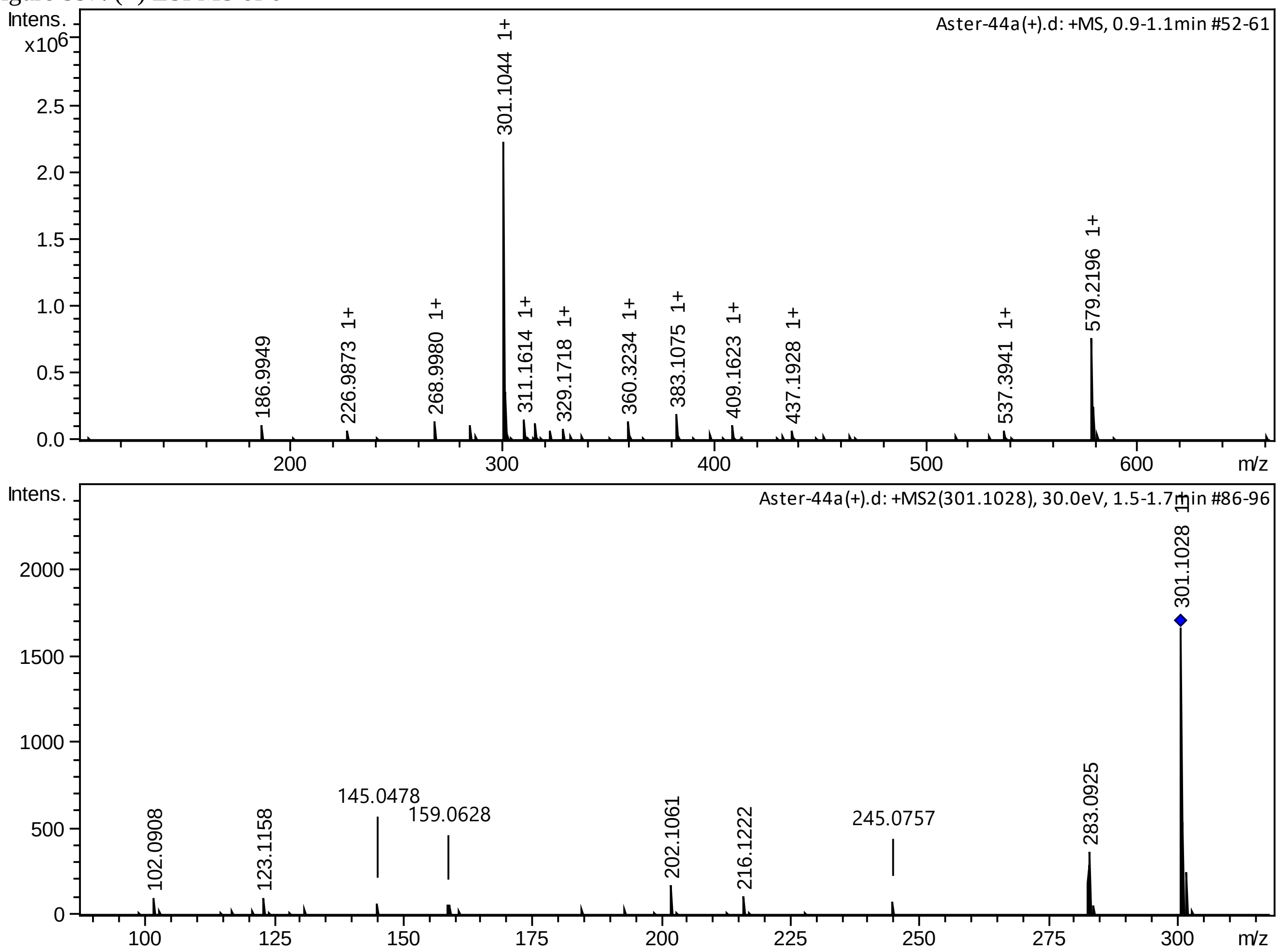

Figure S98. (+) ESI MS of **7**

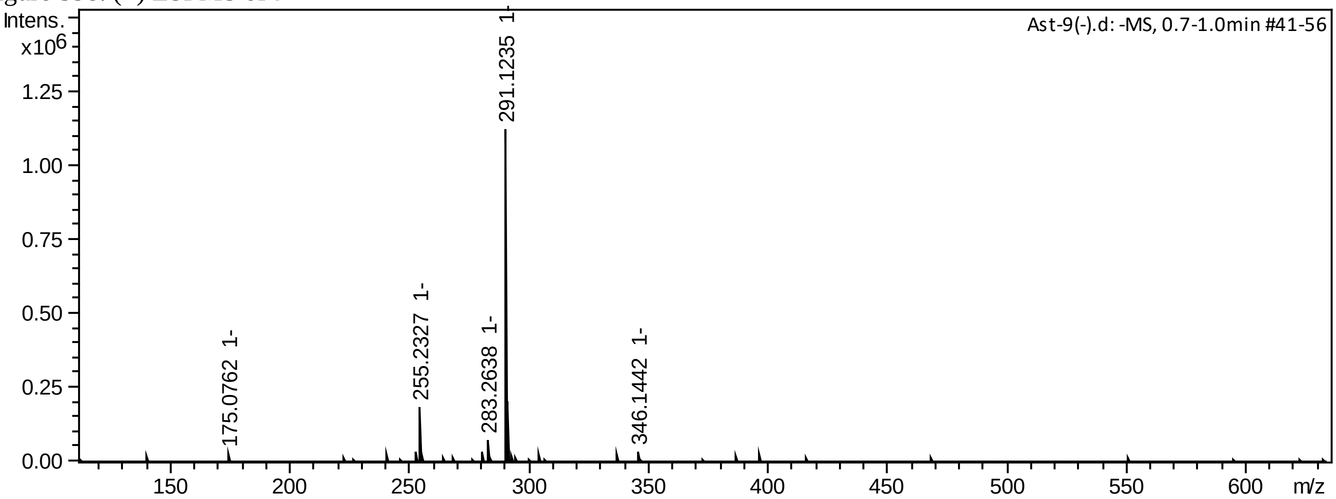


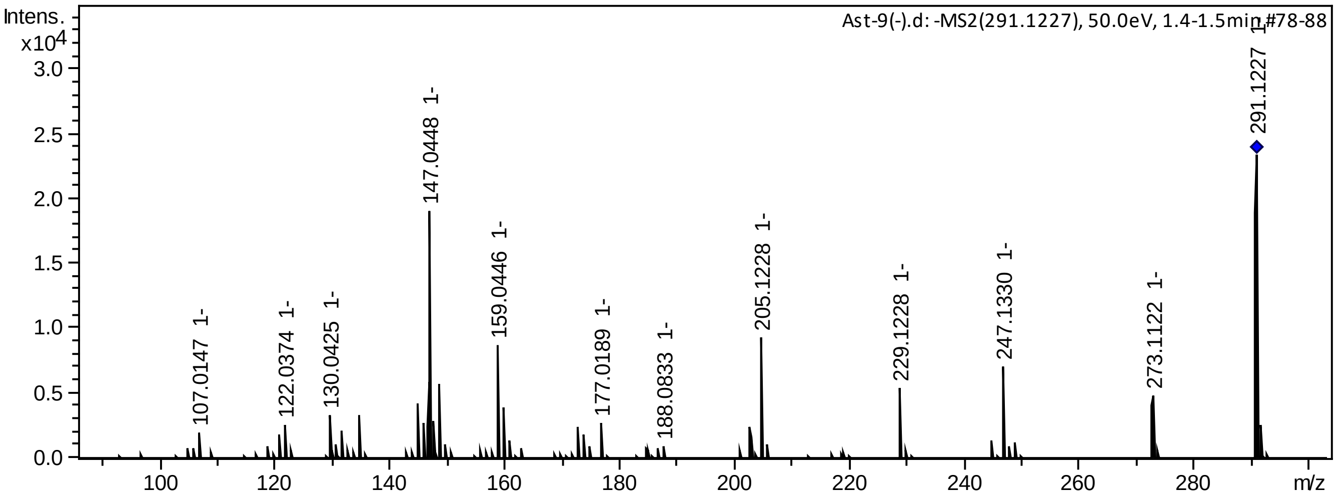

Figure S99. (+) ESI MS of **8**

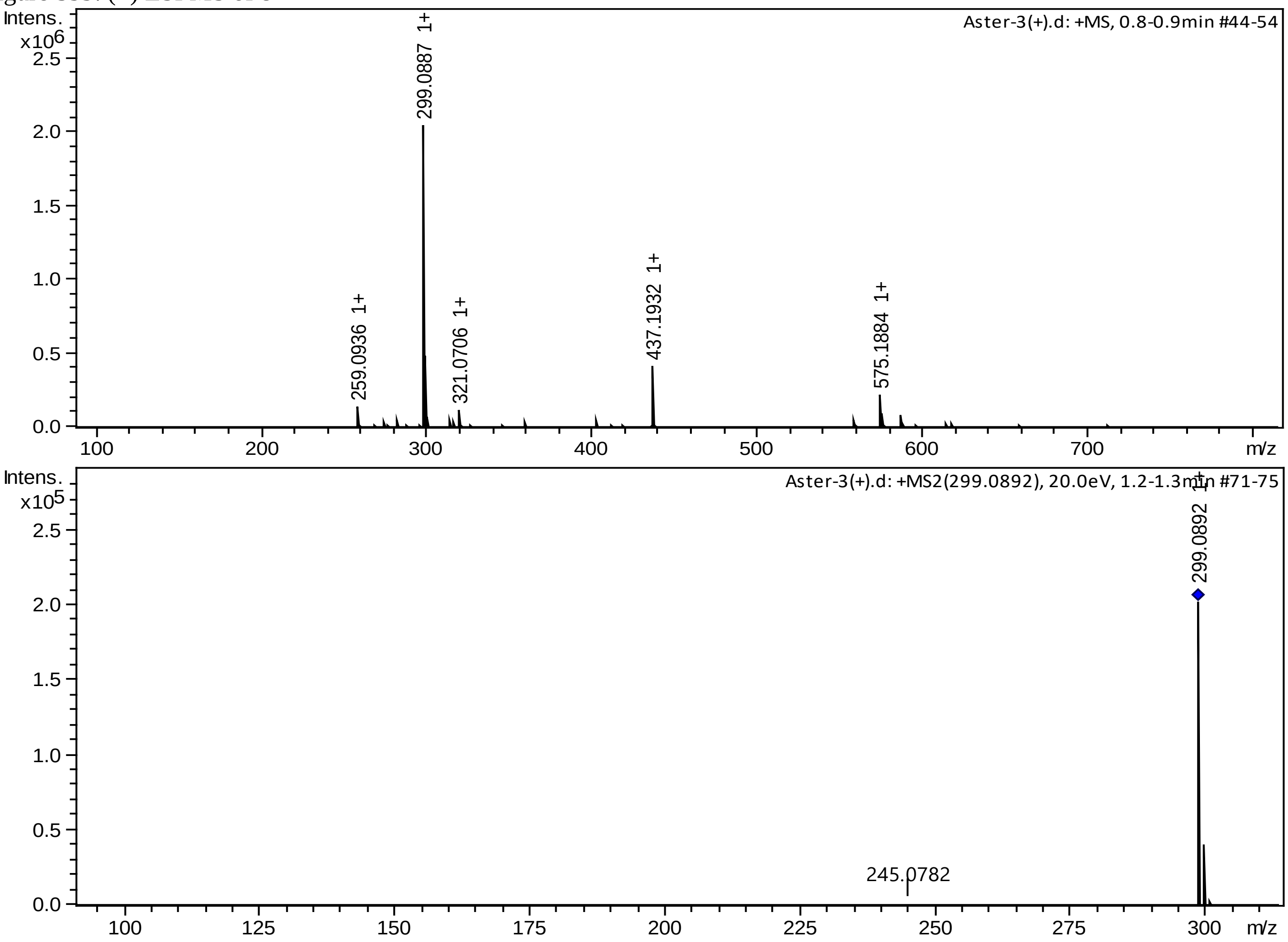

Figure S100. (-) ESI MS of **9**

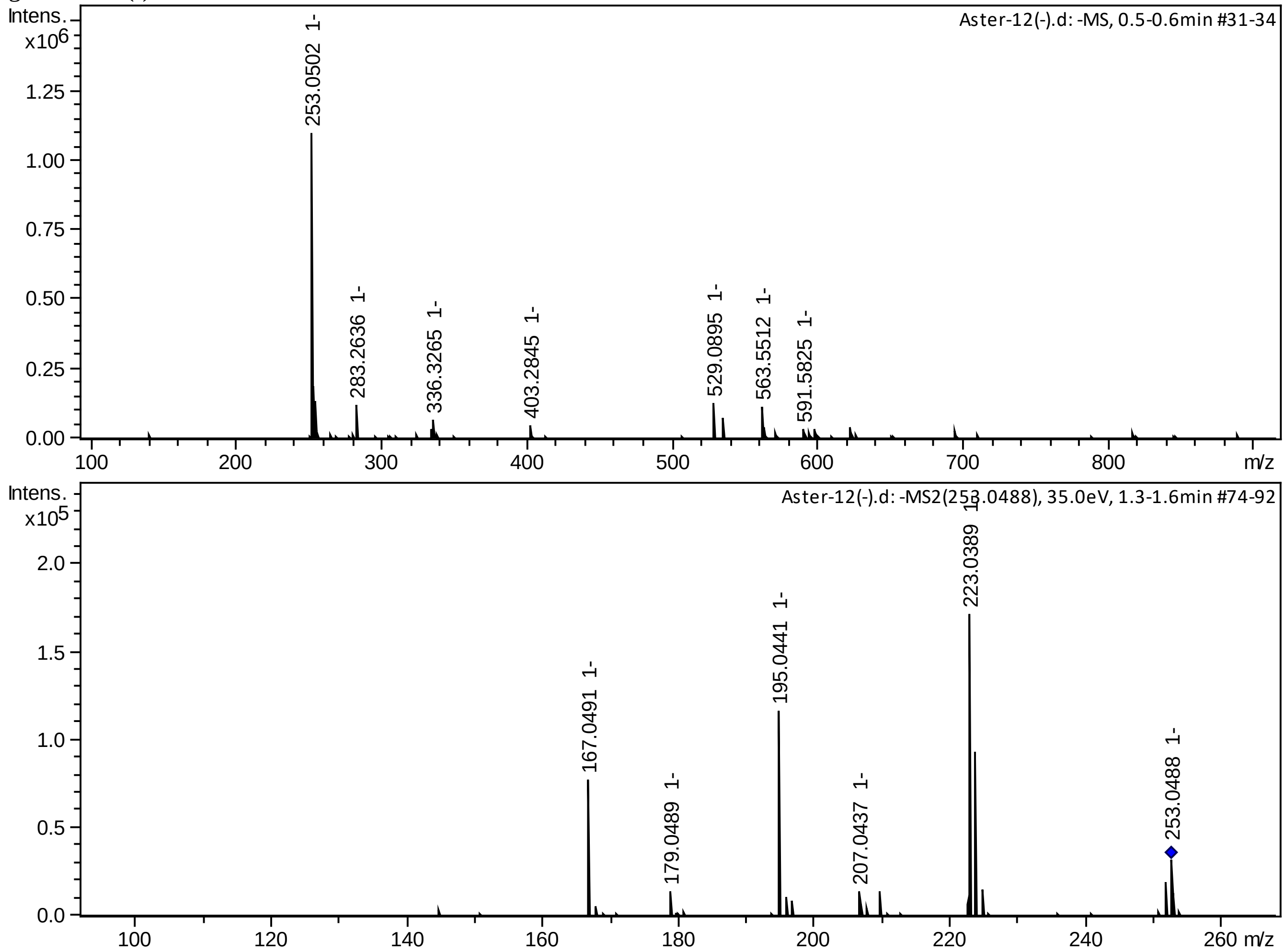

Figure S101. (-) ESI MS of **10**

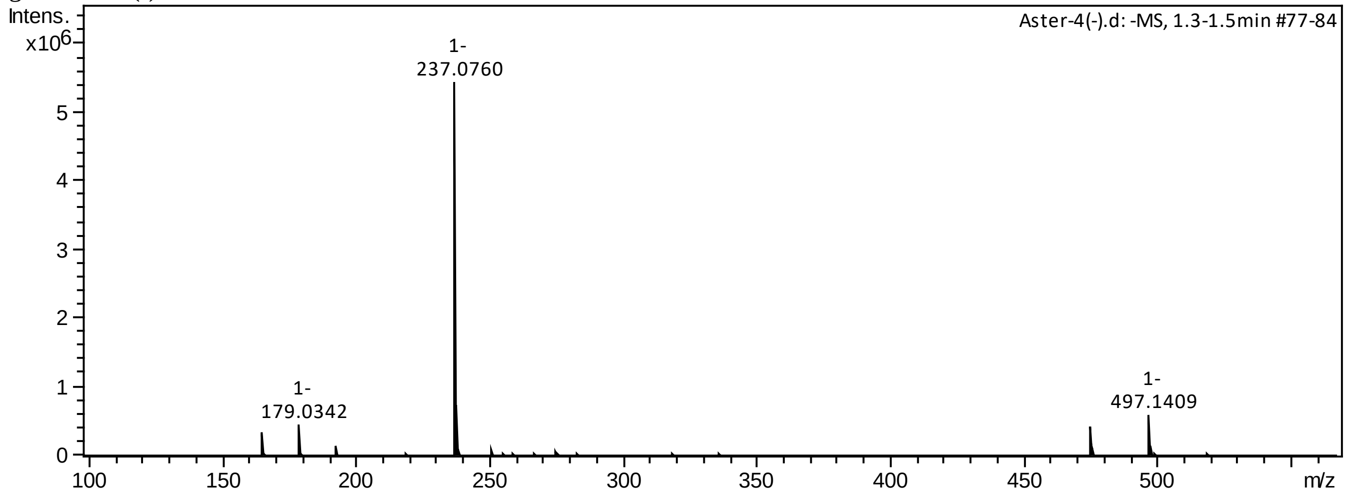


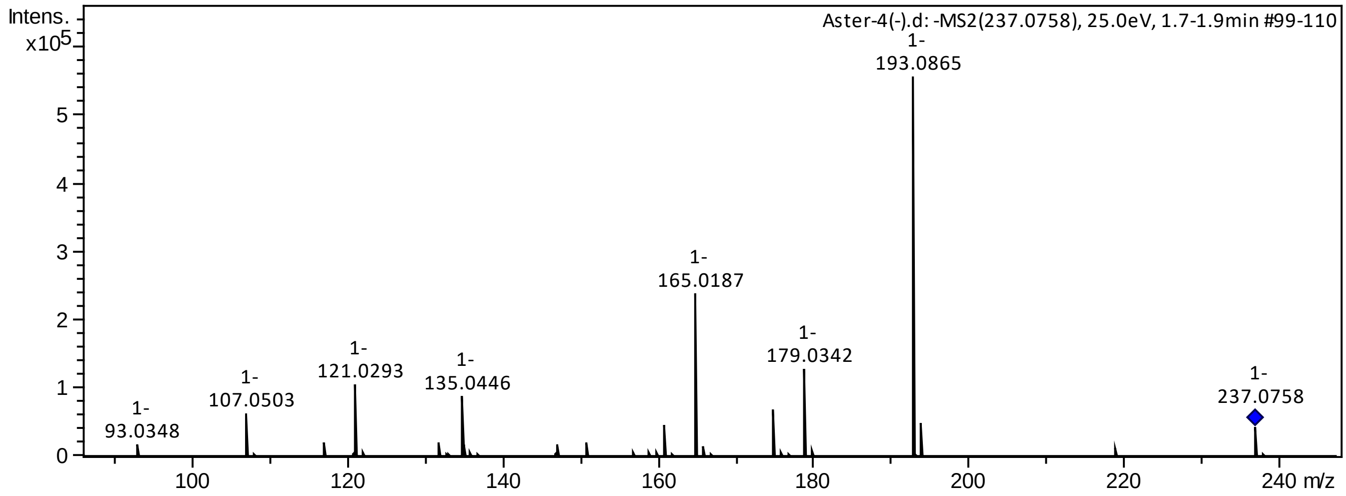

Figure S102. (-) ESI MS of **11**

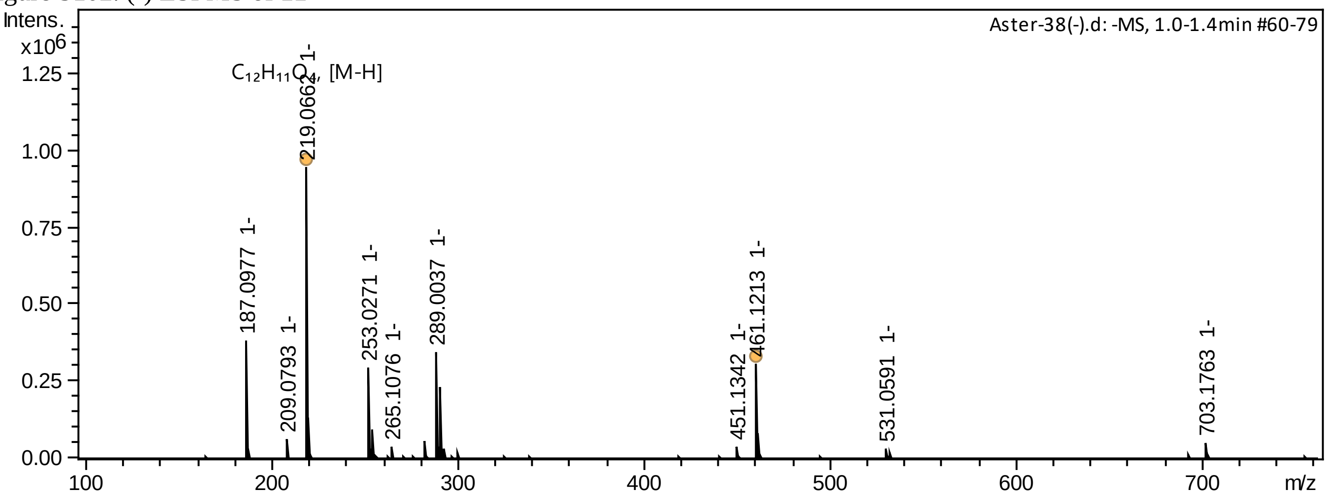


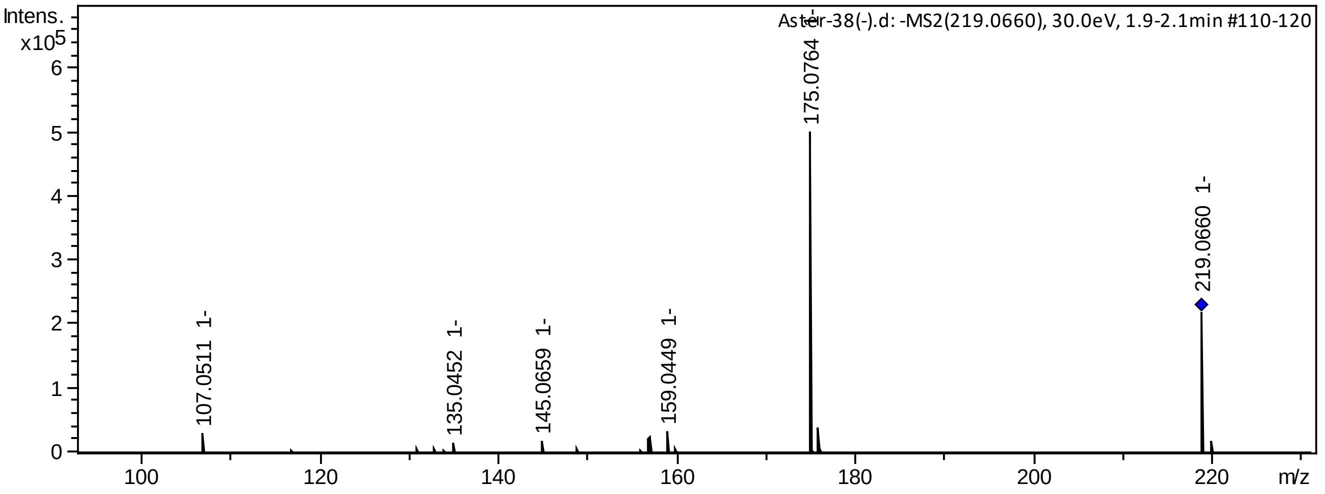


Figure S103. (+) ESI MS of **12**

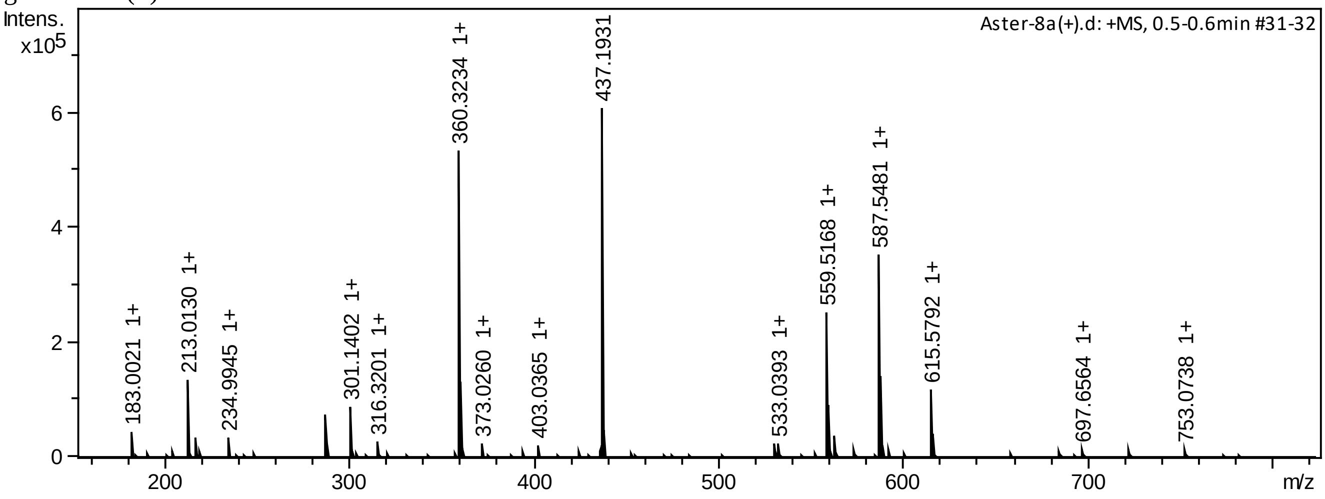

Figure S104. (+) ESI MS of **13**

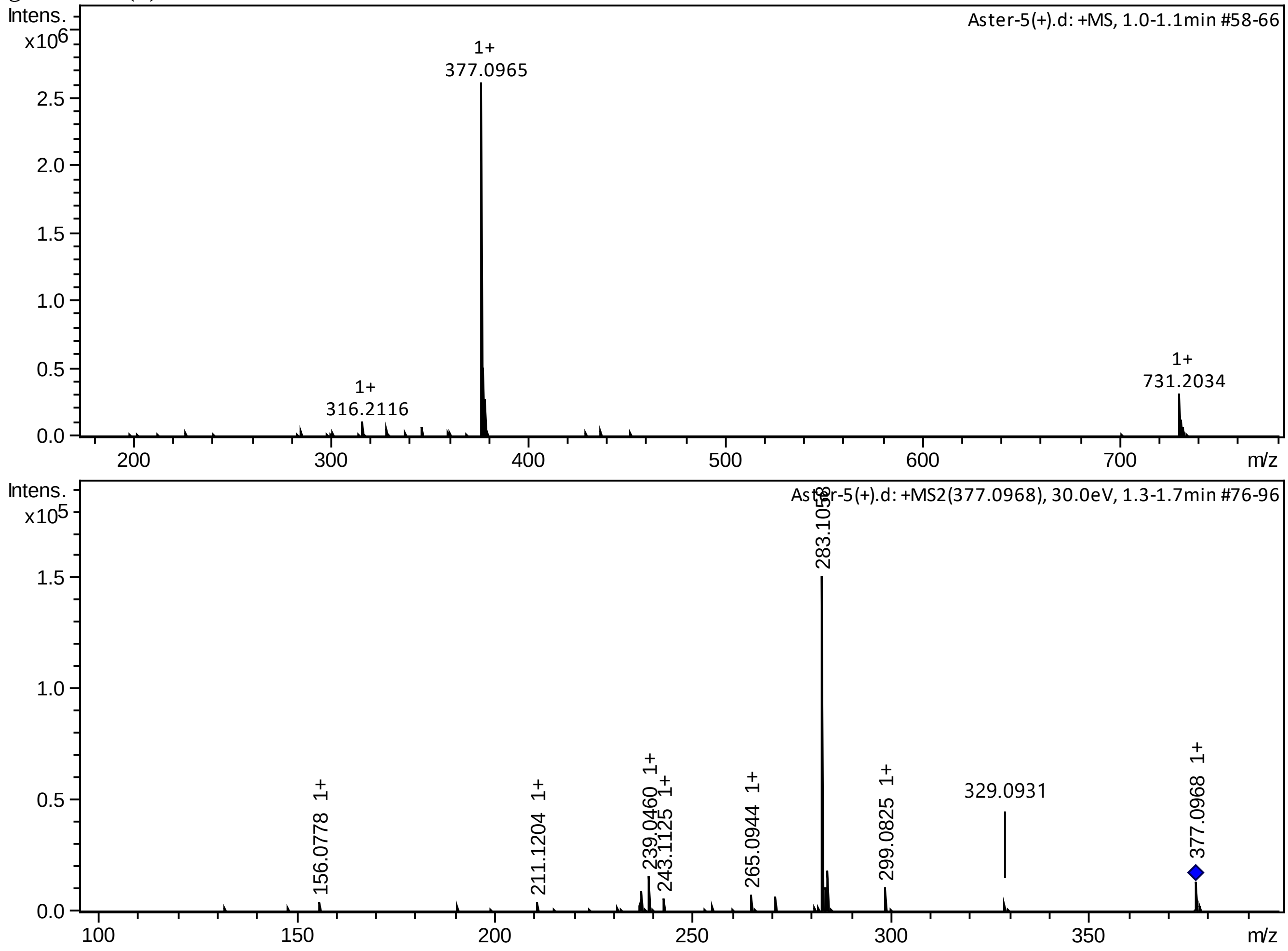

Supplement: Supplementary file 1 [file marinedrugs-21-00431-s001.zip › marinedrugs-2492140-supplementary.pdf]
